# A Convenient Route to New (Radio)Fluorinated and (Radio)Iodinated Cyclic Tyrosine Analogs

**DOI:** 10.3390/ph15020162

**Published:** 2022-01-28

**Authors:** Maria Noelia Chao, Jean-Michel Chezal, Eric Debiton, Damien Canitrot, Tiffany Witkowski, Sophie Levesque, Françoise Degoul, Sébastien Tarrit, Barbara Wenzel, Elisabeth Miot-Noirault, Audrey Serre, Aurélie Maisonial-Besset

**Affiliations:** 1 Inserm, Imagerie Moléculaire et Stratégies Théranostiques, UMR 1240, Université Clermont Auvergne, F-63000 Clermont-Ferrand, France; noeliachao@gmail.com (M.N.C.); j-michel.chezal@uca.fr (J.-M.C.); eric.debiton@uca.fr (E.D.); damien.canitrot@uca.fr (D.C.); tiffany.witkowski@inserm.fr (T.W.); Sophie.LEVESQUE@clermont.unicancer.fr (S.L.); francoise.degoul@inserm.fr (F.D.); sebastien.tarrit@uca.fr (S.T.); elisabeth.noirault@uca.fr (E.M.-N.); audreyserre@live.fr (A.S.); 2 Department of Nuclear Medicine, Jean Perrin Comprehensive Cancer Centre, F-63000 Clermont-Ferrand, France; 3 Helmholtz-Zentrum Dresden-Rossendorf, Research Site Leipzig, Institute of Radiopharmaceutical Cancer Research, 04318 Leipzig, Germany; b.wenzel@hzdr.de

**Keywords:** radioiodination, radiofluorination, tyrosine analogs, TIC(OH), PET/SPECT imaging

## Abstract

The use of radiolabeled non-natural amino acids can provide high contrast SPECT/PET metabolic imaging of solid tumors. Among them, radiohalogenated tyrosine analogs (i.e., [^123^I]IMT, [^18^F]FET, [^18^F]FDOPA, [^123^I]8-iodo-L-TIC(OH), etc.) are of particular interest. While radioiodinated derivatives, such as [^123^I]IMT, are easily available via electrophilic aromatic substitutions, the production of radiofluorinated aryl tyrosine analogs was a long-standing challenge for radiochemists before the development of innovative radiofluorination processes using arylboronate, arylstannane or iodoniums salts as precursors. Surprisingly, despite these methodological advances, no radiofluorinated analogs have been reported for [^123^I]8-iodo-L-TIC(OH), a very promising radiotracer for SPECT imaging of prostatic tumors. This work describes a convenient synthetic pathway to obtain new radioiodinated and radiofluorinated derivatives of TIC(OH), as well as their non-radiolabeled counterparts. Using organotin compounds as key intermediates, [^125^I]5-iodo-L-TIC(OH), [^125^I]6-iodo-L-TIC(OH) and [^125^I]8-iodo-L-TIC(OH) were efficiently prepared with good radiochemical yield (RCY, 51–78%), high radiochemical purity (RCP, >98%), molar activity (Am, >1.5–2.9 GBq/µmol) and enantiomeric excess (e.e. >99%). The corresponding [^18^F]fluoro-L-TIC(OH) derivatives were also successfully obtained by radiofluorination of the organotin precursors in the presence of tetrakis(pyridine)copper(II) triflate and nucleophilic [^18^F]F^−^ with 19–28% RCY d.c., high RCP (>98.9%), Am (20–107 GBq/µmol) and e.e. (>99%).

## 1. Introduction

**Figure 1:**
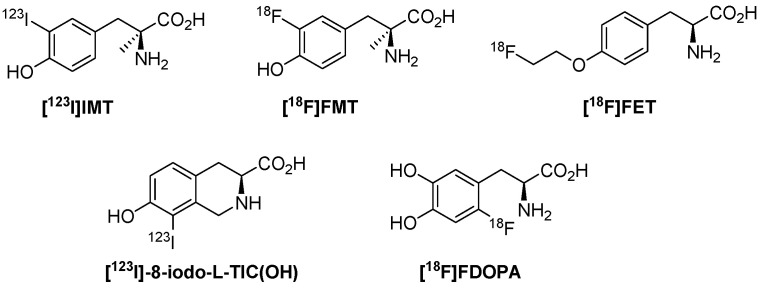
Examples of radioiodinated or radiofluorinated tyrosine analogs.

Amino acid transporters (AATs) are membrane proteins that supply cells with amino acids (AAs), and particularly essential AAs, to support their metabolism, growth and survival [1]. Christensen et al.’s pioneering work in the 1960s described and classified more than 20 AAT systems according to substrate specificity and transport mechanism [2,3,4,5,6]. More recently, AATs have been included in the Solute Carrier (SLC) superfamily of transporter systems [7,8,9]. AATs are currently included in 11 SLC families, with at least 66 different members. Because of the key functional role played by AATs in biological processes, expression alterations or dysfunctions have been linked with several human pathologies, such as neurodegenerative disorders, chronic kidney disease and cancer [10,11]. Tumor cells are highly dependent on nutrients, as they often present uncontrolled growth and a high proliferation rate, supported by increased AATs expression and/or activity [12,13]. This characteristic, leading to an intensive accumulation of AAs in cancer cells compared with normal cells, has been largely exploited in diagnostic applications [14]. Thus, the use of natural or non-natural radiolabeled AAs can provide high contrast Single Photon Emission Computed Tomography (SPECT) or Positron Emission Tomography (PET) imaging of primary cancer lesions and distant metastases in numerous applications, such as staging, treatment follow-up and early detection of recurrence [15]. One of the most upregulated AATs in cancer is the LAT1 system (L system, *SLC7A5*), which transports large neutral AAs, such as branched-chain and aromatic amino acids (leucine, tryptophan, phenylalanine or tyrosine, for example) [16,17,18]. In this context, many non-natural radiohalogenated tyrosine analogs (i.e., [^123^I]IMT, [^18^F]FET, [^18^F]FDOPA, [^123^I]8-iodo-L-TIC(OH), Figure 1) have been developed and thoroughly investigated over the past few decades, mostly for imaging neuroendocrine, prostatic and brain tumors [19,20,21,22,23,24,25,26,27,28]. Indeed, compared with their natural counterparts, non-natural AAs are often less metabolized in vivo. This reduces the production of radiometabolites, which could significantly affect image analysis.

Among all existing halogen radionuclides, fluorine-18 is currently the most attractive positron-emitting radionuclide for PET imaging due to its highly suitable physical and nuclear characteristics. This PET radionuclide displays simple decay and emission properties with high positron abundance (97%). Fluorine-18 has relatively low positron energy (maximum 635 keV) and a short positron linear range in tissue (2.3 mm), which results in high-resolution PET images. Its half-life (109.8 min) is long enough to allow relatively long imaging protocols, therefore facilitating kinetic studies and high-quality metabolite analyses. Moreover, from a radiochemical point of view, fluorine-18 allows multi-step synthetic approaches lasting several hours. Finally, fluorine-18 can be reliably and routinely produced at the multi-Curie level on widely implemented biomedical cyclotrons, using the well-characterized (p, n) nuclear reaction on an oxygen-18-enriched water target with a relatively low-energy proton beam (e.g., 18 MeV). In addition to fluorine-18, the multiple radioisotopes of iodine offer a wide range of applications in nuclear medicine and provide a convenient bridge between animal models and human clinical trials. Indeed, iodine-125 (half-life: 59.41 d; γ emitter) is suitable for pre-clinical in vitro experiments and SPECT imaging on small animal models, while iodine-123 (half-life: 13.22 h; γ emitter) and iodine-124 (half-life: 4.17 d; β^+^ emitter) are well suited for clinical SPECT and PET imaging purposes, respectively. The β^−^-emitting radioisotope, iodine-131 (half-life: 8.03 d), can be used for targeted radionuclide therapeutic approaches. 

While radioiodinated derivatives of some tyrosine analogs ([^123^I]IMT or [^123^I]8-iodo-L-TIC(OH), for example, Figure 1) can be easily produced under mild conditions via well-known electrophilic aromatic substitution reactions, radiofluorinated aryl tyrosine analogs are particularly difficult to obtain. Indeed, direct radiofluorination of electron-rich aromatic structures from a [^18^F]F^−^ source is still a challenge for the scientific community of radiochemists, as evidenced by the number of new methods, starting from promising arylboronate, aryl organotin, aryl sulfonium or iodonium salt precursors that have been published in recent years [29,30,31,32]. The significant progress in methodology recently reported in the radiofluorination of [^18^F]FDOPA, for example, is a perfect illustration of the new opportunities that are now available for producing radiofluorinated arenes that could not be routinely obtained even a few years ago [33,34,35,36,37,38,39,40,41].

**Figure 2:**
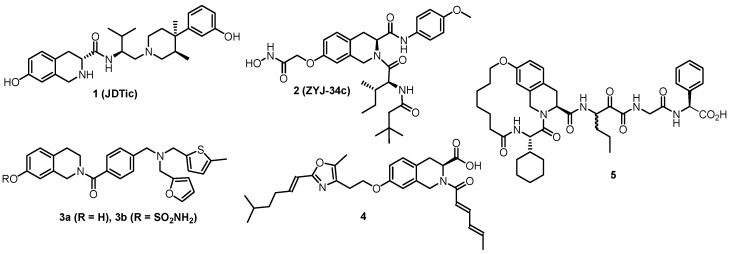
Examples of TIC(OH)-containing compounds used in medicinal chemistry.

Surprisingly, these methodological advances have not been applied to the synthesis of radiofluorinated derivatives of TIC(OH), a cyclic analog of tyrosine, which can be found in several compounds with a wide variety of biological activities (Figure 2). JDTic (**1**) is a potent and selective κ opioid receptor antagonist [42], which displays antidepressant and anxiolytic effects and reduces the signs related to substance abuse in a rodent model [43,44]. ZYJ-34c (**2**) is a histone deacetylase inhibitor, which exhibits higher in vivo antitumor potency in human breast carcinoma and pulmonary metastasis mouse models than the FDA-approved drug, suberoylanilide hydroxamic acid [45]. Compounds **3a** and **3b**, possessing decarboxylated TIC(OH) cores, have also demonstrated anticancer effects in oestrogen-sensitive breast cancers. These compounds present in vitro dual action as selective oestrogen receptor modulators. Moreover, compound **3a** inhibits steroidal sulfatase, while compound **3b** targets the alkaline phosphatase enzyme [46]. Compound **4** has shown hypoglycemic and hypolipidemic effects in a mouse model via peroxisome proliferator-activated receptor γ agonism and protein-tyrosine phosphatase 1B inhibition. These results highlighted that compound **4** can be considered as a candidate drug for the treatment of diabetes [47]. The TIC(OH) scaffold is also found in several peptides, such as non-natural conformation-constrained tyrosine analogs. These include the macrocyclic pentapeptide **5** [48], a potent inhibitor of hepatitis C virus NS3 protease, and Skite and Htc-tide peptides, which have been described as peptidic reporters of protein tyrosine kinase activity due to their efficient phosphorylation and slower dephosphorylation than conventional tyrosine reporters [49,50].

Samnick et al. also obtained very promising results with the [^123^I]8-iodo-L-TIC(OH) radiotracer for SPECT imaging of prostatic tumors in preclinical mouse models [51,52,53]. This compound presented high, rapid and long-lasting tumor uptake (>15% injected dose per gram, ID/g, at 15 min p.i.) associated with fast wash-out from non-target organs. However, the impact in terms of structure–activity relationship of the position of iodine substitution on this radiolabeled tyrosine derivative has never been explored. Furthermore, no radiofluorinated analogs of [^123^I]8-iodo-L-TIC(OH) have been reported so far.

Based on these observations, it would be of great interest to develop radioiodinated isomers at positions 5 and 6 of the TIC(OH) scaffold and to adapt TIC(OH) for ^18^F PET imaging. To this end, we have designed and developed a new synthetic pathway, using common organotin intermediates, to enable easy production of radioiodinated or radiofluorinated 5, 6 and 8 aryl-substituted TIC(OH) analogs. Since radiolabeling techniques are common in all iodine radioisotopes and considering the relatively low cost of iodine-125 and its favorable half-life and dosimetry, we decided to use this radionuclide as a model for our radioiodination protocols.

## 2. Results and Discussion

### 2.1. Chemistry

**Scheme 1:**
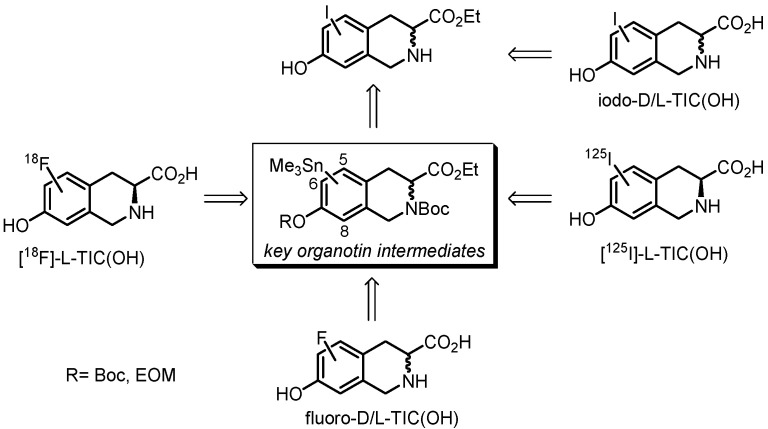
Retrosynthetic pathway to produce [^19^/^18^F]fluoro-D/L-TIC(OH) and [^125^I]iodo-L-TIC(OH) from common key organotin intermediates.

**Scheme 2:**
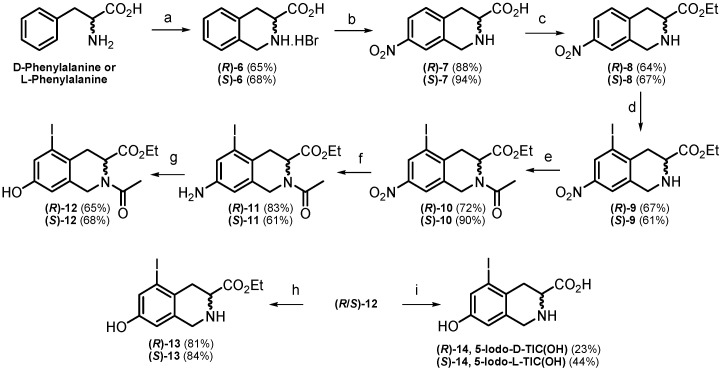
Preparation of iodinated compounds **(*R*/*S*)-14** (5-iodo-D/L-TIC(OH)) and their ester analogs **(*R*/*S*)-13.** Reagents and conditions: (**a**) (i) HBr conc., 40 °C, 10 min, (ii) aq. formaldehyde (37–41%), 75 °C, 4 h; (**b**) (i) NaNO_3_, H_2_SO_4_ conc., 0 °C, 5 min, (ii) NH_4_OH conc., pH = 8; (**c**) SOCl_2_, EtOH, reflux, 20 h; (**d**) NIS, TfOH, r.t., 7.5 h; (**e**) AcCl, Et_3_N, CHCl_3_, r.t., 4.5 h; (**f**) SnCl_2_, EtOH, reflux, 1 h; (**g**) (i) aq. H_2_SO_4_ 0.5 M, H_2_O, 100 °C, 10 min, (ii) NaNO_2_, 0 °C, 30 min, (iii) urea, reflux, 30 min; (**h**) (i) HCl conc., reflux, 7 h, (ii) HCl conc., EtOH, 15 h, (iii) NaHCO_3_ sat., pH = 8; (**i**) (i) HCl 6M, reflux, 4–4.5 h, (ii) aq. KHCO_3_ 5%, pH = 6–7, 4 °C, 16 h.

A convergent synthetic pathway (Scheme 1) was designed to easily produce the [^125^I]iodo-L-TIC(OH) radioiodinated tracers, fluoro-L-TIC(OH) reference fluorinated derivatives and [^18^F]fluoro-L-TIC(OH) radiofluorinated compounds from common key organotin intermediates. These organotin precursors were synthesized from iodinated analogs via a palladium catalyzed I/SnMe_3_ exchange reaction. The non-radiolabeled iodo-L/D-TIC(OH) compounds used as references were prepared by hydrolysis of iodinated ethyl ester intermediates. The reference fluorinated derivatives were obtained by regioselective silver-catalyzed electrophilic aromatic fluorination of the key organotin compounds using the mild fluorinating reagent F-TEDA-PF_6_, while their radiofluorinated analogs, [^18^F]fluoro-L-TIC(OH), were easily produced by copper-mediated nucleophilic aromatic radiofluorination from the organotin intermediates with [^18^F]F^−^. Finally, the [^125^I]iodo-L-TIC(OH) radiotracers were obtained by conventional electrophilic radioiododestannylation under mild conditions. For comparison and control of the enantiomeric excess, the corresponding non-radiolabeled iodinated and fluorinated derivatives from the D series were also synthesized.

(*R*) and (*S*)-ethyl 7-hydroxy-5-iodo-1,2,3,4-tetrahydroisoquinoline-3-carboxylates (**(*R/S*)-13**) were successfully obtained in eight steps starting from commercially available D- and L-phenylalanines, respectively (Scheme 2). Briefly, phenylalanines were treated with an aqueous solution of formaldehyde in the presence of hydrobromic acid, according to the Pictet–Spengler cyclization [54], to obtain tetrahydroisoquinoline derivatives **(*R*/*S*)-6**. Regioselective nitration of the **(*R/S*)-6** compounds at position 7 with sodium nitrate in concentrated sulfuric acid [55] provided the **(*R/S*)-7** compounds. It is worth mentioning that this reaction also led to the formation of minor by-products: the corresponding saturated isoquinoline compound and the 6-nitro derivative, which were removed during the purification step. After esterification of the **(*R/S*)-7** acids with thionyl chloride in ethanol, the ethyl esters **(*R/S*)-8** were regioselectively iodinated at position 5 with iodine(I) trifluoromethanesulfonate generated in situ from N-iodosuccinimide and trifluoromethanesulfonic acid at room temperature [56]. The **(*R/S*)-9** compounds then reacted with acetyl chloride [57] to obtain acetamides **(*R/S*)-10** in good yields (72% and 90%, respectively). Reduction of the nitro group of **(*R/S*)-10** using tin(II) chloride in refluxing ethanol [58] afforded amines **(*R/S*)-11**, which were diazotized with sodium nitrite in sulfuric acid [59] to produce phenols **(*R/S*)-12**. Finally, prolonged heating in hydrochloric acid [60] provided the fully deprotected compounds 5-iodo-TIC(OH) (**(*R/S*)-14**) or esters **(*R/S*)-13** after an additional Fischer esterification step.

**Scheme 3:**
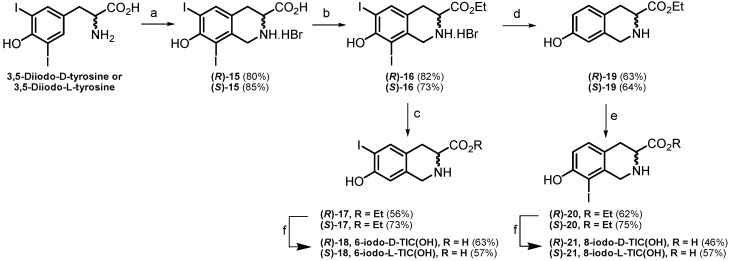
Preparation of iodinated compounds **(*R*/*S*)-18** (6-iodo-D/L-TIC(OH)) and **(*R*/*S*)-21** (8-iodo-D/L-TIC(OH)). Reagents and conditions: (**a**) (i) paraformaldehyde, HBr 33% in AcOH, TFA, 80 °C, 1 h; (ii) paraformaldehyde, 80 °C, 15 h; (**b**) aq. HBr conc., EtOH, reflux, 16 h; (**c**) Zn, aq. HBr conc., EtOH, r.t., 2 h; (**d**) Pd/C 10%, H_2_, Et_3_N, EtOH, r.t., 28 h; (**e**) NIS, CH_2_Cl_2_, ultrasound, r.t., 30 s; (**f**) (i) aq. 1 M LiOH, THF, MeOH, r.t., 1 h; (ii) aq. HCl 1M, pH = 6/7, 4 °C, 2 h.

The preparation of the TIC(OH) derivatives iodinated at positions 6 and 8 (**(*R*/*S*)**-**18** and **(*R*/*S*)**-**21**, respectively) is outlined in Scheme 3. The starting materials (commercial 3,5-diiodo-D/L-tyrosines) were converted into (*R*) and (*S*)-7-hydroxy-6,8-diiodo-1,2,3,4-tetrahydroisoquinoline-3-carboxylic acid hydrobromide salts (**(*R*/*S*)-15**) by paraformaldehyde (PFA) treatment in the presence of hydrobromic and trifluoroacetic acids, according to a modified Pictet–Spengler procedure described by Berrang et al. [61].

The Fischer esterification of **(*R*/*S*)-15** then produced ethyl esters **(*R*/*S*)-16** in good yields (82% and 73%, respectively). Monoiodinated compounds at position 6 (**(*R*/*S*)-17**) were obtained from **(*R*/*S*)-16** by regioselective reduction with zinc/hydrobromic acid in ethanol [62]. The common intermediates **(*R*/*S*)-16** were also hydrogenated using palladium on charcoal as a catalyst in the presence of trimethylamine [63] to afford the derivatives **(*R*/*S*)-19**. Finally, regioselective iodination at position 8 of **(*R*/*S*)-19** with *N*-iodosuccinimide in dichloromethane under ultrasound activation [64] provided the **(*R*/*S*)-20** compounds. It is worth mentioning that this regioselective monoiodination reaction has to be performed with 0.33 equivalents of *N*-iodosuccinimide at a concentration not exceeding 1.5 × 10^−3^ M and with a short reaction time to prevent the formation of the unwanted 6,8-diiodinated by-product. The final amino acids **(*R*/*S*)-18** (6-iodo-D/L-TIC(OH)) and **(*R*/*S*)-21** (8-iodo-D/L-TIC(OH)) were obtained by saponification of esters **(*R*/*S*)-17** and **(*R*/*S*)-20** using 1 M aqueous lithium hydroxide solution in THF, followed by precipitation at pH 6–7.

### 2.2. Radiochemistry

#### 2.2.1. Synthesis of [^125^I]iodo-L-TIC(OH) Compounds

**Scheme 4:**
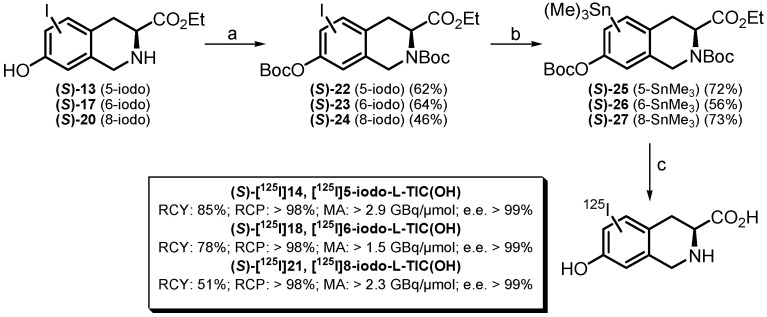
Preparation of [^125^I]iodo-L-TIC(OH) compounds (**(*S*)-[^125^I]14, (*S*)-[^125^I]18** and **(*S*)-[^125^I]21**). Reagents and conditions: (**a**) Boc_2_O, Et_3_N, DMAP, CH_2_Cl_2_, r.t., 16–20 h; (**b**) Sn_2_Me_6_, Pd(PPh_3_)_4_, 1,4-dioxane, reflux, 1.5 h for **(*S*)-25** and **(*S*)-26** or (i) 1.3 M iPrMgCl.LiCl, THF, −40 °C, 20 min; (ii) Me_3_SnCl, THF, −40 °C, 3 h then r.t. for **(*S*)-27**; (**c**) (i) [^125^I]NaI, CAT, EtOH, 1M PBS buffer, r.t., 5 min; (ii) aq. NaOH 10 M, 0 °C then r.t., 1 h; (iii) TFA, 0 °C then 60 °C, 30 min.

The synthetic approach for producing radioiodinated [^125^I]iodo-L-TIC(OH) compounds (**(*S*)-[^125^I]14**, **(*S*)-[^125^I]18** and **(*S*)-[^125^I]21**) is outlined in Scheme 4. Synthesis of the corresponding key organotin intermediates requires prior protection of both amine and phenol functions of the iodinated esters **(*S*)-13**, **(*S*)-17** and **(*S*)-20**. The protecting groups must be stable under the conditions used for the synthesis of organotin compounds, as well as in the oxidative medium required for the successful production of radioiodinated derivatives. Furthermore, they must be cleaved at low temperature after the radioiodination step to avoid thermal deiodination. Finally, removal of the protecting groups should prevent the formation of by-products to facilitate the final purification steps of the [^125^I]iodo-L-TIC(OH) radiotracers.

Based on these considerations, we chose *tert*-butoxycarbonyl (Boc) protecting groups, which can be easily introduced in a single step on both amine and phenol functions and cleaved by mild acidic hydrolysis with concomitant liberation of lowly reactive and volatile by-products (CO_2_ and isobutene). As depicted in Scheme 4, the protected compounds **(*S*)-22**, **(*S*)-23** and **(*S*)-24** were synthesized from iodinated ethyl esters **(*S*)-13**, **(*S*)-17** and **(*S*)-20** by Boc_2_O treatment in the presence of triethylamine and *N*,*N*-dimethylaminopyridine (DMAP). To obtain the corresponding organotin precursors, the fully protected compounds **(*S*)-22**, **(*S*)-23** and **(*S*)-24** were then treated with hexamethylditin and tetrakis(triphenylphosphine)palladium(0) in refluxing 1,4-dioxane [33]. Under these conditions, organotin derivatives **(*S*)-25** and **(*S*)-26** were obtained in good yields (72% and 56%, respectively), while only small amounts of the 8-substituted organotin derivative **(*S*)-27** were isolated (yield < 20%). For this compound, TLC monitoring of the reaction highlighted the formation of several by-products, probably due to thermal decomposition of the organotin product. We therefore tried to synthesize this compound using a slightly modified protocol of Mentzel et al. [65] involving treatment of the iodinated starting material **(*S*)-24** with an isopropylmagnesium chloride lithium chloride complex to generate an organomagnesium intermediate in situ, to enable a low temperature (−40 °C) reaction with trimethyltin chloride. Under these conditions, the organotin compound **(*S*)-27** was successfully produced with a good yield (73%).

**Figure 3:**
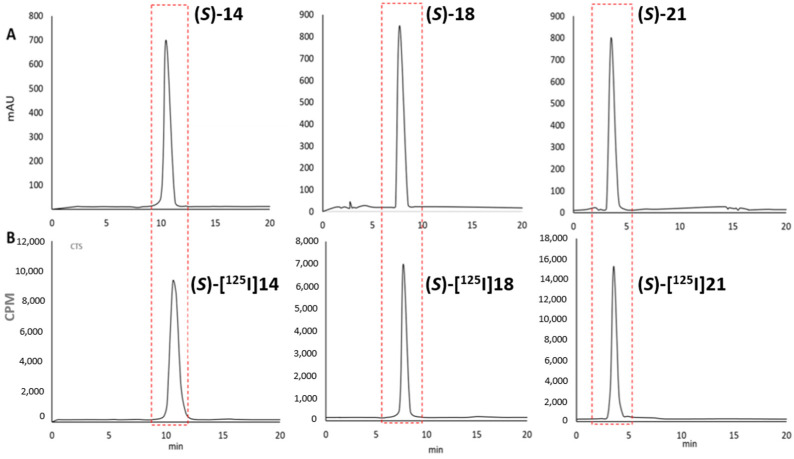
(**A**): UV-HPLC chromatograms (288 nm) of reference iodinated compounds **(*S*)-14**, **(*S*)-18** and **(*S*)-21**; (**B**): Radioactivity-HPLC chromatograms of the corresponding radiotracers **(*S*)-[^125^I]14** ([^125^I]5-iodo-L-TIC(OH)), **(*S*)-[^125^I]18** ([^125^I]6-iodo-L-TIC(OH)) and **(*S*)-[^125^I]21** ([^125^I]8-iodo-L-TIC(OH)) obtained after semi-preparative RP-HPLC purification and formulation. The radioactivity detector was connected in series after the UV detector accounting for a slight difference in retention times (≈0.3 min) observed between ^125^I and ^127^I products.

The organotin compounds **(*S*)-25**, **(*S*)-26** and **(*S*)-27** were then radiolabeled with high molar activity using a radioiodo-demetallation reaction with no-carrier-added [^125^I]NaI in the presence of Chloramine-T as a mild oxidative agent. To prevent the *tert*-butoxycarbonyl protecting groups from hydrolyzing under the acidic conditions that are normally used in these radioiodination processes, the pH of the reaction mixtures was maintained at 7 using a phosphate buffer. After optimization of the reaction parameters (i.e., Chloramine-T concentration, temperature, etc.), the corresponding radioiodinated intermediates were successfully produced in 5 min with high labeling efficiencies (>95%). We then turned our attention to the deprotection of these compounds to obtain the desired [^125^I]iodo-L-TIC(OH) derivatives. The removal of all protecting groups was performed under mild conditions using a two-step deprotection, consisting of saponification of the ethyl ester function, followed by acidic cleavage of the Boc groups. Under these conditions, hydrolysis of the ester function was achieved after 1 h using 10 M aqueous sodium hydroxide solution with limited deiodination (<5%, determined by radio-TLC and analytical radio-HPLC monitoring). The *N*- and *O*-Boc protecting groups were then removed by adding an excess of trifluoroacetic acid at 0 °C, followed by moderate heating at 60 °C for 30 min. After purification by anion-exchange solid-phase extraction and semi-preparative RP-HPLC and subsequent formulation in a sterile saline solution, **(*S*)-[^125^I]14** ([^125^I]5-iodo-L-TIC(OH)), **(*S*)-[^125^I]18** ([^125^I]6-iodo-L-TIC(OH)) and **(*S*)-[^125^I]21** ([^125^I]8-iodo-L-TIC(OH)) were efficiently isolated with good radiochemical yields (RCY, 51–78%), high radiochemical purities (RCP, >98%, Figure 3), molar activities (>1.5–2.9 GBq/µmol) and enantiomeric excess (>99%, Figure S1 in ESI). Interestingly, these three radioiodinated derivatives were found to be stable in the formulation medium for up to 7 days after production.

#### 2.2.2. Synthesis of [^19^F]fluoro-D/L-TIC(OH) References and [^18^F]fluoro-L-TIC(OH) Radiotracers

**Scheme 5:**
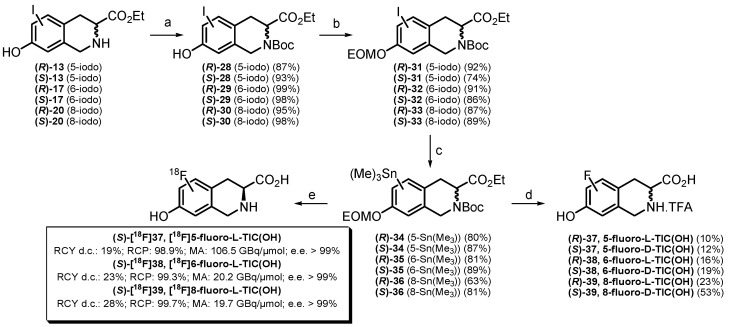
Preparation of [^18^F]fluoro-L-TIC(OH) compounds (**(*S*)-[^18^F]37**, **(*S*)-[^18^F]38** and **(*S*)-[^18^F]39**) and [^19^F]fluoro-D/L-TIC(OH) references (**(*R*/*S*)-37**, **(*R*/*S*)-38** and **(*R*/*S*)-39**). Reagents and conditions: (**a**) Boc_2_O, THF, aq. NaHCO_3_, r.t., 2–6 h; (**b**) EtOCH_2_Cl, DIPEA, THF, 40 °C, 16–22 h; (**c**) Sn_2_Me_6_, Pd(PPh_3_)_4_, 1,4-dioxane, reflux, 0.5–3 h for **(*R*/*S*)-34** and **(*R*/*S*)-35** or (i) 1.3 M *i*PrMgCl.LiCl, THF, −40 °C, 30 min; (ii) Me_3_SnCl, THF, −40 °C, 1 h then r.t., 2 h for **(*R*/*S*)-36**; (**d**) (i) Ag_2_O, NaHCO_3_, NaOTf, F-TEDA-PF_6_, acetone, 40–80 °C, 2–4 h; (ii) 7.2 M HCl in dioxane, r.t., 5 min; (iii) aq. 1M LiOH, MeOH, THF, r.t., 1–15 h; (**e**) (i) [^18^F]NaF, Cu(OTf)_2_(py)_4_, *N*,*N*-dimethylacetamide, 110 °C, 10 min, (ii) aq. HBr conc. 110 °C, 10 min.

The synthetic pathway to obtain the [^19^F]fluoro-D/L-TIC(OH) references and radiofluorinated compounds [^18^F]fluoro-L-TIC(OH) is depicted in Scheme 5. The amine function of the **(*R*/*S*)-13**, **(*R*/*S*)-17** and **(*R*/*S*)-20** derivatives was selectively protected with a Boc group by treatment with Boc_2_O and sodium bicarbonate in THF to afford derivatives **(*R*/*S*)-28**, **(*R*/*S*)-29** and **(*R*/*S*)-30**, respectively. The **(*R*/*S*)-28**, **(*R*/*S*)-29** and **(*R*/*S*)-30** compounds were then treated with chloromethyl ethyl ether and *N*,*N*-diisopropylethylamine in THF to produce ethoxymethyl (EOM) ethers **(*R*/*S*)-31**, **(*R*/*S*)-32** and **(*R*/*S*)-33**. The EOM group was selected to protect the phenol function, based on previous studies [33,38] demonstrating its excellent stability under the conditions commonly used for nucleophilic radiofluorination.

Organotin derivatives **(*R*/*S*)-34** and **(*R*/*S*)-35** were obtained in very good yields (80–89%) from **(*R*/*S*)-31** and **(*R*/*S*)-32** by a reaction with hexamethylditin and tetrakis(triphenylphosphine)palladium(0) as catalyst in refluxing 1,4-dioxane. However, this reaction failed to produce the 8-substituted tin derivative **(*R*/*S*)-36**, as previously observed for **(*S*)-27**. Therefore, the optimized reaction conditions for treating the iodinated starting material **(*R*/*S*)-33** with an isopropylmagnesium chloride lithium chloride complex and trimethyltin chloride at low temperature were used to produce **(*R*/*S*)-36**. Finally, the **[^19^F]fluoro-D/L-TIC(OH)** references were prepared by silver-catalyzed electrophilic aromatic fluorination of organotin compounds followed by a two-step deprotection. Electrophilic fluorination was carried out by treating **(*R*/*S*)-34**, **(*R*/*S*)-35**, and **(*R*/*S*)-36** with silver(I) oxide, sodium hydrogen carbonate, sodium triflate and 1-chloromethyl-4-fluoro-1,4-diazoniabicyclo[2.2.2]octane bis(hexafluorophosphate) (F-TEDA-PF_6_), easily obtained from commercial Selectfluor [66], according to the procedure described by Tang et al. [67]. This reaction was chosen for its broad substrate scope and large functional group tolerance, which allows late-stage fluorination. However, the silver-catalyzed electrophilic fluorination reaction turned out to be very moisture sensitive and, as a result, the hydrodestannylated by-product was generally formed. When applied to **(*R*/S)-34**, **(*R*/*S*)-35** and **(*R*/*S*)-36**, these reaction conditions led to a mixture of F/H derivatives in a ratio ranging from 38/62 to 93/7, determined by ^1^H NMR. Moreover, despite several attempts, the desired fluorinated products could not be separated from this by-product due to their similar structural properties. We therefore used a mixture of fully protected **[^19^F]fluoro-D/L-TIC(OH)** and the hydrodestannylated by-product in the next deprotection step without further purification. All our attempts to deprotect the phenolic and amino acid functions in a single step with concentrated aqueous acids, such as hydrogen chloride or hydrogen bromide, and at high temperatures, unfortunately led to by-product formation. Deprotection was therefore performed in two successive steps, involving cleavage of the EOM and *N*-Boc groups with hydrogen chloride in dioxane, followed by ester saponification with lithium hydroxide, both at room temperature. Finally, **(*R*/*S*)-37**, **(*R*/*S*)-38** and **(*R*/*S*)-39** references were isolated as trifluoroacetate salts after purification by preparative RP-HPLC.

**(*S*)-[^18^F]37**, **(*S*)-[^18^F]38** and **(*S*)-[^18^F]39** were obtained using copper-mediated radiofluorination of organotin compounds **(*S*)-34**, **(*S*)-35** and **(*S*)-36** with [^18^F]NaF in the presence of tetrakis(pyridine)copper(II) triflate. The [^18^F]NaF was prepared from an aqueous solution of [^18^F]F^−^ in [^18^O]H_2_O via a procedure slightly modified from Makaravage et al. [35]. Briefly, radioactive fluorides were trapped on a QMA carbonate cartridge, previously washed with an aqueous solution of sodium triflate, and efficiently eluted to the reaction vessel using an aqueous solution of sodium triflate containing a small amount (50 µg) of potassium carbonate (recovery: >96.3%, *n* = 17). After azeotropic drying using acetonitrile, the [^18^F]NaF was diluted in anhydrous *N*,*N*-dimethylacetamide (DMA) for use as a solvent in the subsequent radiofluorination step. This process enabled copper-mediated nucleophilic radiofluorination of the organotin compounds **(*S*)-34**, **(*S*)-35** and **(*S*)-36** in the presence of small amounts of base, preventing any degradation of the precursors in the reaction mixture. Aliquots of the resulting solution (200–300 µL) were used to optimize the reaction parameters. As stated by Bowden et al., the amount of organotin precursor used, the nature of copper complex introduced and the molar ratios of reagents are often critical parameters for the copper-mediated radiofluorination [68].

A screening of reaction parameters was therefore conducted with the precursor **(*S*)-35** to determine the optimal conditions of radiofluorination for the ^18^F-TIC(OH) analogs. First, we tested Cu(OTf)_2_ (2 or 4 molar equivalents (eq.) relative to the precursor) and anhydrous pyridine as an additive (2.5 to 25 molar eq. relative to the precursor) (Table 1) while maintaining constant the total volume of DMA (550 µL) and the temperature (110 °C, 10 min). The most promising results were obtained with 20 µmol of the organotin precursor **(*S*)-35** in the presence of 2 molar eq. of copper complex and 4 or 5 molar eq. of pyridine (entries 4 and 5). Under these conditions, RCY of around 30% could be achieved (determined by radio-TLC analyses of the reaction mixture). Interestingly, higher amounts of pyridine were always detrimental for this radiofluorination step and resulted in very low RCY (entries 6–8). Increasing the amount of copper complex and/or precursor (entries 9–14) or extending the reaction time (data not shown) did not have a significant effect on radiolabeling efficiency. At this step, no radiochemical impurities were identified during the TLC monitoring of the reaction mixture, and only the desired radiofluorinated intermediate and the remaining [^18^F]fluoride were visible.

A second set of experiments investigated the influence of the nature of the copper complex (i.e., Cu(OTf)_2_/pyridine vs. tetrakis(pyridine)copper(II) triflate, Cu(OTf)_2_(py)_4_) (Table 1, entries 15–18). Different amounts of Cu(OTf)_2_(py)_4_ were used while keeping constant the quantity of precursor **(*S*)-35** (20 µmol), the total volume of DMA (550 µL), the temperature (110 °C) and reaction time (10 min). Interestingly, higher RCY yields were obtained compared with the in situ combination of Cu(OTf)_2_ and pyridine described above (i.e., 37, 54, 41 or 36% when using 1, 1.5, 2 or 2.5 eq. of Cu(OTf)_2_(py)_4_, respectively). Longer heating at 110 °C did not induce a significant increase in labeling efficiency, proving that optimal RCY can be obtained in a very short time. With a view to facilitate further purification steps of the reaction mixture, the condition using small amounts of Cu(OTf)_2_(py)_4_ (1.5 eq.) (Entry 16) was selected and successfully applied to the two other organotin precursors **(*S*)-34** and **(*S*)-36**. Under these optimized and reproducible conditions, high RCY were obtained (93 ± 3%, *n* = 5 for **(*S*)-34**; 54 ± 8%, *n* = 5 for **(*S*)-35**; and 59 ± 17%, *n* = 5 for **(*S*)-36**) (see Figures S2–S4 in ESI for representative radio-TLC). The reaction mixtures were diluted with water and loaded on a SepPak C18 cartridge to remove the [^18^F]fluoride. The radiofluorinated intermediates trapped on the cartridge were then efficiently eluted using methanol. After evaporation under argon flow at 70 °C, all the protective groups were removed by heating in concentrated hydrobromic acid. Complete deprotection was achieved after 10 min at 110 °C. Then, final semi-preparative RP-HPLC purification and formulation in sterile saline solution were carried out.

**Figure 4:**
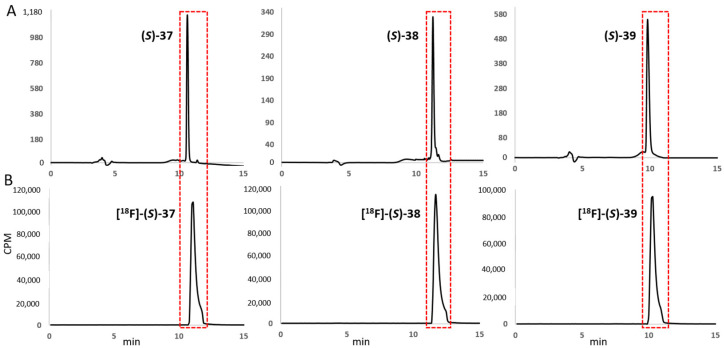
(**A**): UV-HPLC chromatograms (288 nm) of reference fluorinated compounds **(*S*)-37**, **(*S*)-38** and **(*S*)-39**; (**B**): Radioactivity-HPLC chromatograms of the corresponding radiotracers **(*S*)-[^18^F]37** (**[^18^F]5-fluoro-L-TIC(OH)**), **(*S*)-[^18^F]38** (**[^18^F]6-fluoro-L-TIC(OH)**) and **(*S*)-[^18^F]39** (**[^18^F]8-fluoro-L-TIC(OH)**) obtained after semi-preparative RP-HPLC purification and formulation. The radioactivity detector was connected in series after the UV detector accounting for the slight difference in retention times (≈0.3 min) observed between ^18^F and ^19^F products.

The desired radiotracers **(*S*)-[^18^F]37** ([^18^F]5-fluoro-L-TIC(OH)), **(*S*)-[^18^F]38** ([^18^F]6-fluoro-L-TIC(OH)) and **(*S*)-[^18^F]39** ([^18^F]8-fluoro-L-TIC(OH)) were isolated with high radiochemical purities (RCP > 98.9%, Figure 4), radiochemical yields (RCY: 19–28%), molar activities (MA: 20–107 GBq/µmol) and enantiomeric excess (e.e. > 99%, Figure S5 in ESI) in a total radiosynthesis time of generally less than 2 h.

## 3. Materials and Methods

### 3.1. Radiochemistry with Iodine-125

#### 3.1.1. General Information

[^125^I]NaI (3.97 GBq/mL, 643.8 MBq/mg) was purchased from PerkinElmer Life and Analytical Sciences (331 Treble Cove Road, Billerica, MA, USA) as a no-carrier-added solution in reductant-free 1.0 × 10^−5^ M aqueous sodium hydroxide solution (pH 8–11). The radio TLC strips (Merck neutral aluminum oxide 60F_254_ plates) were developed with dichloromethane/ethanol (97/3, *v*/*v*) and measured on an AMBIS 400 (Scanalytics, CSPI, San Diego, CA, USA). Semi-preparative RP-HPLC purifications were performed on a Perkin Elmer system equipped with a Flexar LC autosampler, a Series 200 pump, a Peltier column oven, a vacuum degasser, a Photodiode Array Detector (PDA) and a GabiStar detector (Raytest). The separation was carried out on a Nucleodur C-18 H-Tec column (Macherey-Nagel, 5 µm, 10 × 250 mm) using the following conditions: isocratic elution, flow rate = 1.2 mL/min, ammonium formate 20 mM/EtOH (80/20, *v*/*v*), λ = 254 and 288 nm. Analytical HPLC measurements were performed on a system consisting of a HP1100 (Hewlett Packard, Les Ulis, France) and a Flo-one A_500_ Radiomatic detector (Packard, Canberra, Australia). The separation was carried out on a C-18 column (Agilent Zorbax, 5 μm, 4.6 × 150 mm) using the following conditions: isocratic elution, flow rate = 0.7 mL/min, ammonium formate 20 mM/EtOH (95/5, *v*/*v*), λ = 254 and 288 nm. For the determination of the enantiomeric excess of each radiolabeled compound, the analytical HPLC measurements were performed on a Reprosil Chiral-AA 8 µm column (250 × 4.6 mm; 8 µm; CIL Cluzeau; Sainte-Foy-la-Grande, France) using the following conditions: water/ACN (30/70, *v*/*v*) as isocratic eluent mixture and a flow rate of 1 mL/min. Oasis MCX Plus extraction short cartridges (225 mg, 60 µm) were purchased from Waters. All radiolabeled compounds were compared by TLC or analytical HPLC to the authentic non-radioactive material and to be free of significant UV-absorbing chemical and radiochemical impurities.

#### 3.1.2. General Method for the Syntheses of **(*S*)-[^125^I]14** ([^125^I]5-iodo-L-TIC(OH)), **(*S*)-[^125^I]18** ([^125^I]6-iodo-L-TIC(OH)) and **(*S*)-[^125^I]21** ([^125^I]8-iodo-L-TIC(OH))

To a solution of organotin precursors **(*S*)-25**, **(*S*)-26** or **(*S*)-27** (0.25 µmol) in ethanol (100 µL) were successively added an aqueous 1 M phosphate buffer solution (pH 7, 100 µL), an aqueous chloramine-T solution (1 mg/mL, 50 µL) and [^125^I]NaI (5–12 µL, 15.9–19.6 MBq). The reaction mixture was stirred at room temperature for 5 min and cooled to 0 °C in an ice bath before addition of a cold aqueous sodium hydroxide solution (10 M, 50 µL). The reaction mixture was then stirred at room temperature for 1 h. After cooling to 0 °C, trifluoroacetic acid (500 µL) was added to the reaction mixture, which was further stirred at 60 °C in a sealed vial for 30 min. After cooling to 0 °C, the solution was neutralized by careful addition of an aqueous sodium hydroxide solution (10 M, 660 µL) and diluted with an aqueous citrate buffer solution (0.1 M, pH 5, 18 mL) before passing through an anionic cartridge (MCX Plus extraction short cartridges, Waters). The latter was successively washed with an aqueous formic acid solution (2% vol., 500 µL) and methanol (500 µL). Then, the recovery of the radioactive compound was performed by elution of the cartridge with a solution of ammonia in methanol (5/95, *v*/*v*, 2 mL). The eluate was concentrated under reduced pressure to obtain a final volume of 200 µL and purified by semi-preparative RP-HPLC to give **(*S*)-[^125^I]14** (**[^125^I]5-iodo-L-TIC(OH))**, **(*S*)-[^125^I]18** (**[^125^I]6-iodo-L-TIC(OH)**) and **(*S*)-[^125^I]21** (**[^125^I]8-iodo-L-TIC(OH)**) after evaporation under reduced pressure.

(3*S*)-7-hydroxy-5-[^125^I]iodo-1,2,3,4-tetrahydroisoquinoline-3-carboxylic acid **(*S*)-[^125^I]14** ([^125^I]5-iodo-L-TIC(OH)).

**(*S*)-[^125^I]14** (16.7 MBq) was synthesized according to the general protocol described above, starting from stannane compound **(*S*)-25** (150 µg, 0.25 µmol) and [^125^I]NaI (12 µL, 19.6 MBq). RCY: 85%; RCP: >98%; MA: >2.9 GBq/µmol; e.e. > 99%.

(3*S*)-7-hydroxy-6-[^125^I]iodo-1,2,3,4-tetrahydroisoquinoline-3-carboxylic acid **(*S*)-[^125^I]18** ([^125^I]6-iodo-L-TIC(OH)).

**(*S*)-[^125^I]18** (14.3 MBq) was synthesized according to the general protocol described above, starting from stannane compound **(*S*)-26** (150 µg, 0.25 µmol) and [^125^I]NaI (12 µL, 18.3 MBq). RCY: 78%; RCP: >98%; MA: >1.5 GBq/µmol; e.e. > 99%.

(3*S*)-7-hydroxy-8-[^125^I]iodo-1,2,3,4-tetrahydroisoquinoline-3-carboxylic acid **(*S*)-[^125^I]21** ([^125^I]8-iodo-L-TIC(OH)).

**(*S*)-[^125^I]21** (8.1 MBq) was synthesized according to the general protocol described above, starting from stannane compound **(*S*)-27** (150 µg, 0.25 µmol) and [^125^I]NaI (5 µL, 15.9 MBq). RCY: 51%; RCP: >98%; MA: >2.3 GBq/µmol; e.e. > 99%.

### 3.2. Radiochemistry with Fluorine-18

#### 3.2.1. General Information

No-carrier-added fluorine-18 was produced by Curium Pharma, via the [^18^O(p, n)^18^F] nuclear reaction by irradiation of a 2.8 mL > 97%-enriched [^18^O]H_2_O target on a PETtrace cyclotron (16 MeV proton beam, GE healthcare). Radio thin layer chromatography (radio-TLC) was performed on silica pre-coated TLC sheets (Alugram^®^ Xtra Sil G/UV_254_, Macherey-Nagel, Hoerdt, France), eluted with a mixture of ethyl acetate/cyclohexane (3/7, *v*/*v*) and measured on a miniGITA Dual radio-TLC instrument (Elysia-Raytest, Liège, Belgium). Analytical HPLC measurements were performed on a system consisting of an Agilent HP series 1100 (Hewlett Packard, Les Ulis, France) combined with a Flo-one A500 Radiomatic detector (Packard, Canberra, Australia). A Reprosil Chiral-AA 8 µm column (250 × 4.6 mm; 8 µm; CIL Cluzeau; Sainte-Foy-la-Grande; France) was employed for the determination of the enantiomeric excess of the produced radiotracers using the following solvent conditions: isocratic elution with a mixture of water/ACN (70/30, *v*/*v*) and a flow rate of 1 mL/min. For the determination of the radiochemical purities (RCP), a Zorbax Extend-C18 analytical column (4.6 × 150 mm, 5 µm, Agilent, Les Ulis, France) was employed using the following solvent conditions: water containing 0.1% of trifluoroacetic acid (solvent A) and methanol containing 0.1% of trifluoroacetic acid (solvent B); 0 to 3 min: isocratic elution 95% A; 3 to 15 min: gradient elution 95% → 5% A; 15 to 25 min: isocratic elution 5% A; 25 to 30 min: gradient elution 5% → 95% A with a flow rate of 1 mL/min. Unless otherwise indicated, all HPLC purifications and analyses were performed at λ = 254 nm. Preparation of anhydrous [^18^F]NaF and semi-preparative HPLC purifications were performed using a SynChrom R&D EVOI synthesis module (Raytest). A semi-preparative Waters Symmetryprep C18 column (300 × 7.8 mm; 7 µm; Waters) was used with 20 mM aq. ammonium bicarbonate/ethanol (99/1, *v*/*v*) as isocratic eluent at a flow rate of 2 mL/min. Sep-Pak^®^ Light Accell Plus QMA carbonate cartridges (130 mg, 37–55 µm) and Sep-Pak^®^ light C18 Plus cartridges (130 mg, 55–105 µm) were purchased from Waters. All radiolabeled compounds were compared by TLC or analytical HPLC to the authentic non-radioactive material and to be free of significant UV-absorbing chemical and radiochemical impurities.

#### 3.2.2. Preparation of Anhydrous [^18^F]NaF in DMA

The aqueous solution of [^18^F]F^−^ in [^18^O]H_2_O obtained from Curium Pharma was passed through an anion exchange resin (Sep-Pak^®^ Light Accell Plus QMA carbonate cartridge, Waters) preconditioned with 10 mL of ethanol, 10 mL of an aqueous solution of sodium trifluoromethanesulfonate (90 mg/mL) and 10 mL of water (without drying) following the protocol of Makaravage et al. [35]. The radioactivity was eluted to the reactor using a solution of potassium carbonate (50 µg) and sodium trifluoromethanesulfonate (10 mg) in water (550 µL) before the addition of anhydrous acetonitrile (1 mL). The resulting solution was dried by azeotropic distillation under reduced pressure and He flow at 100 °C for 12 min. After cooling to 30 °C, anhydrous *N*,*N*-dimethylacetamide (DMA, 2–2.5 mL) were added to the reactor.

#### 3.2.3. General Method for the Syntheses of **(*S*)-[^18^F]37** ([^18^F]5-fluoro-L-TIC(OH)), **(*S*)-[^18^F]38** ([^18^F]6-fluoro-L-TIC(OH)) and **(*S*)-[^18^F]39** ([^18^F]8-fluoro-L-TIC(OH))

To a solution of anhydrous [^18^F]NaF in anhydrous *N*,*N*-dimethylacetamide (200 µL, 36–1380 MBq, *n* = 22) were successively added a solution of organotin precursors **(*S*)-34**, **(*S*)-35** or **(*S*)-36** (10.8 mg, 20 µmol) in anhydrous *N*,*N*-dimethylacetamide (100 µL) and a solution of tetrakis(pyridine)copper(II) triflate (Cu(OTf)_2_(py)_4_, 20.3 mg, 30 µmol) in anhydrous *N*,*N*-dimethylacetamide (250 µL). The reaction mixture was stirred at 110 °C for 10 min. After cooling to 0 °C and addition of water (3 mL), the reaction mixture was passed through a Sep-Pak light C18 cartridge, preconditioned with 10 mL of water, 10 mL of methanol and 10 mL of water. The cartridge was washed with water (1 mL) and flushed with air before elution of the desired ^18^F-labeled intermediate with methanol (750 µL). After gentle evaporation of the solvent under argon flow at 70 °C, a concentrated hydrobromic acid solution (48 wt%, 200 µL) was added. The reaction mixture was heated at 110 °C for 10 min. After cooling to 0 °C, the mixture was diluted with 2 mL of a mixture of 20 mM aq. ammonium bicarbonate/ethanol (99/1, *v*/*v*) and purified by RP-HPLC. The collected fractions were diluted in saline and analyzed by analytical radio-HPLC to determine the radiochemical purity, enantiomeric excess and specific activity.

(3*S*)-7-hydroxy-5-[^18^F]fluoro-1,2,3,4-tetrahydroisoquinoline-3-carboxylic acid **(*S*)-[^18^F]37** ([^18^F]5-fluoro-L-TIC(OH)).

**(*S*)-[^18^F]37** (123 MBq) was synthesized according to the general protocol described above, starting from organotin compound **(*S*)-34** (10.8 mg, 20 µmol), tetrakis(pyridine)copper(II) triflate (20.3 mg, 30 µmol) and [^18^F]NaF (100 µL, 1.12 GBq). RCY d.c.: 19%; RCP: 98.9%; MA: 106.5 GBq/µmol; e.e. > 99%.

(3*S*)-7-hydroxy-6-[^18^F]fluoro-1,2,3,4-tetrahydroisoquinoline-3-carboxylic acid **(*S*)-[^18^F]38** ([^18^F]6-fluoro-L-TIC(OH)).

**(*S*)-[^18^F]38** (125 MBq) was synthesized according to the general protocol described above, starting from organotin compound **(*S*)-35** (10.8 mg, 20 µmol), tetrakis(pyridine)copper(II) triflate (20.3 mg, 30 µmol) and [^18^F]NaF (100 µL, 1.01 GBq). RCY d.c.: 23%; RCP: 99.3%; MA: 20.2 GBq/µmol; e.e. > 99%.

(3*S*)-7-hydroxy-8-[^18^F]fluoro-1,2,3,4-tetrahydroisoquinoline-3-carboxylic acid **(*S*)-[^18^F]39** ([^18^F]8-fluoro-L-TIC(OH)).

**(*S*)-[^18^F]39** (112 MBq) was synthesized according to the general protocol described above, starting from organotin compound **(*S*)-36** (10.8 mg, 20 µmol), tetrakis(pyridine)copper(II) triflate (20.3 mg, 30 µmol) and [^18^F]NaF (100 µL, 772 MBq). RCY d.c.: 28%; RCP: 99.7%; MA: 19.7 GBq/µmol; e.e. > 99%.

## 4. Conclusions

A novel and convenient pathway, involving common organotin intermediates, was successfully developed for the production of radioiodinated or radiofluorinated TIC(OH) analogs halogenated at positions 5, 6 or 8. This could pave the way for producing new radiohalogenated derivatives of well-known medicinal compounds containing the TIC(OH) scaffold, which could, in turn, lead to a broad set of applications not only in nuclear medicine but also in pharmaceutical and medicinal chemistry.

## Figures and Tables

**Table 1:** Optimization of the reaction parameters for copper-mediated radiofluorination of **(*S*)-35**.

Entry	(*S*)-35 (µmol)	Cu Complex	Eq. Cu Complex ^1^	Eq. of Pyrizdine ^1^	RCY ^2^ (%)
1	10	Cu(OTf)_2_	2	15	5
2	10	Cu(OTf)_2_	4	15	28
3	20	Cu(OTf)_2_	2	2.5	7
4	20	Cu(OTf)_2_	2	4	30
5	20	Cu(OTf)_2_	2	5	31
6	20	Cu(OTf)_2_	2	7.5	24
7	20	Cu(OTf)_2_	2	15	11
8	20	Cu(OTf)_2_	2	25	9
9	20	Cu(OTf)_2_	4	5	15
10	20	Cu(OTf)_2_	4	7.5	18
11	20	Cu(OTf)_2_	4	15	12
12	20	Cu(OTf)_2_	4	25	5
13	30	Cu(OTf)_2_	2	5	27
14	30	Cu(OTf)_2_	4	15	12
15	20	Cu(OTf)_2_(py)_4_	1	0	37
16	20	Cu(OTf)_2_(py)_4_	1.5	0	54
17	20	Cu(OTf)_2_(py)_4_	2	0	41
18	20	Cu(OTf)_2_(py)_4_	2.5	0	36

^1^ related to the molar amount of precursor. ^2^ determined by radio-TLC analyses of the crude reaction mixture after 10 min of reaction time at 110 °C.

## Data Availability

Data sharing is containing in this article.

## Supplementary Materials

The following are available online at https://www.mdpi.com/article/10.3390/ph15020162/s1, Experimental procedures for the syntheses of compounds **6**–**39**, Figure S1: Analytical HPLC measurements of the enantiomeric excess of the radioiodinated tracers, Figure S2: Representative radio-TLC (SiO_2_, cyclohexane/ethyl acetate, 7/3, *v*/*v*) of the crude reaction mixture after radiofluorination of precursor **(*S*)-34**, Figure S3: Representative radio-TLC (SiO_2_, cyclohexane/ethyl acetate, 7/3, *v*/*v*) of the crude reaction mixture after radiofluorination of precursor **(*S*)-35**, Figure S4: Representative radio-TLC (SiO_2_, cyclohexane/ethyl acetate, 7/3, *v*/*v*) of the crude reaction mixture after radiofluorination of precursor **(*S*)-36**, Figure S5: Analytical HPLC measurements of the enantiomeric excess of the radiofluorinated tracers, ^1^H NMR and ^13^C NMR spectra of compounds **6**–**39**.
