# Supplementary material for: A Convenient Route to New (Radio)Fluorinated and (Radio)Iodinated Cyclic Tyrosine Analogs
Source: Pharmaceuticals (Basel). 2022 Jan 28;15(2):162. doi: 10.3390/ph15020162 (PMC8877694; doi:10.3390/ph15020162)
Supplement: Supplementary file 1 [file pharmaceuticals-15-00162-s001.zip › pharmaceuticals-1545139-supplementary.pdf]

Supplementary information

A Convenient Route to New (Radio)Fluorinated and (Radio)Iodinated Cyclic Tyrosine Analogs

Maria Noelia Chao [1,†], Jean-Michel Chezal [1,†], Eric Debiton [1], Damien Canitrot [1], Tiffany Witkowski [1], Sophie Levesque [1,2], Françoise Degoul [1], Sébastien Tarrit [1], Barbara Wenzel [3], Elisabeth Miot-Noirault [1], Audrey Serre [1] and Aurélie Maisonial-Besset [1,*]

1 Inserm, Imagerie Moléculaire et Stratégies Théranostiques, UMR 1240, Université Clermont Auvergne, F-63000 Clermont-Ferrand, France; noeliachao@gmail.com (M.N.C.); j-michel.chezal@uca.fr (J.-M.C.); eric.debiton@uca.fr (E.D.); damien.canitrot@uca.fr (D.C.); tiffany.witkowski@inserm.fr (T.W.); Sophie.LEVESQUE@clermont.unicancer.fr (S.L.); francoise.degoul@inserm.fr (F.D.); sebastien.tarrit@uca.fr (S.T.); elisabeth.noirault@uca.fr (E.M.-N.); audreyserre@live.fr (A.S.)

2 Department of Nuclear Medicine, Jean Perrin Comprehensive Cancer Centre, F-63000 Clermont-Ferrand, France

3 Helmholtz-Zentrum Dresden-Rossendorf, Research Site Leipzig, Institute of Radiopharmaceutical Cancer Research, 04318 Leipzig, Germany; b.wenzel@hzdr.de

* Correspondence: aurelie.maisonial@uca.fr

† These authors contributed equally to this work.

Table of content

1. **Synthetic procedures** **S1**

General information **S1**

Experimental procedures for the syntheses of compounds **6**-**39** **S2**

2. **Radiochemistry** **S31**

Figure S1: Analytical HPLC measurements of the enantiomeric excess of the radioiodinated tracers **S31**

Figure S2: Representative radio-TLC (SiO_2, cyclohexane/ethyl acetate, 7/3, v/v) of the crude reaction mixture after radiofluorination of precursor **(*S*)-34** **S32**

Figure S3: Representative radio-TLC (SiO_2, cyclohexane/ethyl acetate, 7/3, v/v) of the crude reaction mixture after radiofluorination of precursor **(*S*)-35** **S32**

Figure S4: Representative radio-TLC (SiO_2, cyclohexane/ethyl acetate, 7/3, v/v) of the crude reaction mixture after radiofluorination of precursor **(*S*)-36** **S33**

Figure S5: Analytical HPLC measurements of the enantiomeric excess of the radiofluorinated tracers **S33**

3. **^{1}H NMR and ^{13}C NMR spectra of compounds 6-39** **S34**

4. **References** **S94**

1. Synthetic procedures

General information

All commercially available reagents and solvents were purchased at the following commercial suppliers: Sigma Aldrich, Alpha Aesar, ABX, Acros Organics, Fisher Scientific, Carlo Erba Reagents, ABCR or Bachem. All solvents were dried using common techniques.[1] Unless otherwise noted, moisture sensitive reactions were conducted under dry argon atmosphere. Analytical thin layer chromatography (TLC) was performed on precoated silica gel 60 F_{254} or neutral aluminium oxide 60 F_{254} plates (Merck or Macherey-Nagel) and visualized with UV light (254 nm) and/or developed with phosphomolybdic acid (8 wt.%) in ethanol, ninhydrin (0.2 wt.%) in ethanol, or iodine. Flash column chromatography was performed on silica gel 60A normal phase, 35–70 µm (Merck or SDS) or neutral aluminium oxide 90 standardized, 63-200 µm (Merck). Preparative RP-HPLC purifications were carried out on a CombiFlash EZ prep system (Teledyne Isco) equipped with a UV-visible detector. Separation was performed on a C18 column (Teledyne, Redisep Prep C18, 20 mm x 250 mm, 100 Å pore size, 5 µm) at room temperature using the following solvent conditions: 0.1% trifluoroacetic acid in water (solvent A) and 0.1% trifluoroacetic acid in acetonitrile (solvent B); 0-30 min: isocratic elution 95% A; 30-40 min: gradient elution 95%→0% A; 40-45 min: isocratic elution 100% B. The mobile phase flow rate was maintained at 15 $mL.min^{-1}$ and eluents were monitored at 280 and 220 nm. Uncorrected melting points (mp) were recorded on an electrothermal capillary Digital Melting Point Apparatus IA9300 (Bibby Scientific). NMR spectra (200.13, 400.13 or 500.13 MHz for ^{1}H and 50.32, 100.6 or 125.77 MHz for ^{13}C) were recorded on Bruker Avance 200, 400 or 500 instruments with chemical shift values (δ) expressed in parts per million (ppm) relative to residual solvent as standard and coupling constants (*J*) are given in Hz. ^{19}F NMR spectra (470.3 MHz) were recorded on a Bruker Avance 500 apparatus using trifluorotoluene as internal reference (-63.72 ppm). Infrared spectra (IR) were recorded in the range of 4000–440 cm^{-1} on a Nicolet IS10 (Fisher Scientific) with attenuated total reflectance (ATR) accessory. Compounds were analysed by High-Resolution Mass Spectrometry (HRMS) in positive mode (Waters® Micromass® Q-Tof micro™ Mass Spectrometer) and/or by electrospray ionization mass spectra (ESI-MS) recorded on a Esquire-LC ion trap mass spectrometer (Bruker Daltonics). For ESI-MS, the analysis of samples was performed at a final concentration between 1 and 10 pmol/µL. Each ESI-MS spectrum was recorded by averaging of 10 spectra.The analytical chiral chromatographic separations were performed on a JASCO LC-2000 system, incorporating a PU-2080*Plus* pump, AS-2055*Plus* auto injector (100 µL sample loop), and a MD-2010 diode-array-detector (monitoring from 195-600 nm) using a Reprosil Chiral-AA column 8 µm, 250 × 4.6 mm, Dr. Maisch GmbH, Germany (Method A: isocratic elution, water/ACN, 30/70, v/v, flow rate: 1.0 mL/min, λ = 292 nm; Method B: isocratic elution, aqueous 20 mM NH_4OAc/ACN, 35/65, v/v, flow rate: 1 mL/min, λ = 224 nm; Method C: isocratic elution, aqueous 20 mM NH_4OAc/ACN, 30/70, v/v, flow rate: 1 mL/min, λ = 224 nm). The polarimetric identification of the enantiomers was performed by using the Chiral Detector OR-2090 (JASCO Deutschland GmbH, Germany).

General method for the syntheses of 1,2,3,4-tetrahydroisoquinoline-3-carboxylic acids, hydrobromide salts ((*R*/*S*)-6)

These compounds were obtained according to the protocol described by Chelopo *et al.*[2]

A solution of commercial D/L-phenylalanines (0.12 mol) in concentrated hydrobromic acid solution (48 wt.%, 120 mL) was heated at 40 °C for 10 min before dropwise addition of an aqueous solution of formaldehyde (37-41 wt.%, 36.3 mL, 0.48 mol, 4 eq.). The reaction mixture was then heated at 75 °C for 4 h. After cooling to room temperature, the orange precipitate was filtered, washed with cold ethanol (3 × 50 mL) and dried under vacuum in a heating desiccator (35 °C) to yield the desired hydrobromide salts **(*R*/*S*)-6**.

(3*S*)-1,2,3,4-tetrahydroisoquinoline-3-carboxylic acid, hydrobromide salt ((*S*)-6)

Compound **(*S*)-6** (off-white solid, 21.1 g, 82.1 mmol) was synthesized according to the general protocol described above starting from commercial L-phenylalanine (20.0 g, 0.12 mol). Yield: 68%; mp 319-320 °C (Litt.[2] 295-300 °C decomposition); IR (ATR accessory) ν 3100-2900, 2800-2600, 1748, 1574, 1395, 1191, 1102 cm^{-1}; ^1H NMR (200.13 MHz, DMSO-d_6) δ 3.11 (dd, 1H, ^2J = 17.0 Hz, ^3J = 11.1 Hz), 3.31 (dd, 1H, ^2J = 17.0 Hz, ^3J = 5.1 Hz), 4.35 (s, 2H), 4.47 (dd, 1H, ^3J = 5.1, 11.1 Hz), 7.26 (s, 4H), 9.67 (brs, 1H); ^{13}C NMR (50.32 MHz, DMSO-d_6) δ 28.1, 43.9, 53.2, 126.6, 126.9, 127.6, 128.3, 128.8, 130.7, 170.0; HRMS: *m*/*z* calculated for $C_{10}H_{12}NO_2^+$ $[M+H]^+$: 178.0863, found: 178.0852.

(3*R*)-1,2,3,4-tetrahydroisoquinoline-3-carboxylic acid, hydrobromide salt ((*R*)-6)

Compound **(*R*)-6** (off-white solid, 25.4 g, 98.2 mmol) was synthesized according to the general protocol described above starting from commercial D-phenylalanine (25.0 g, 0.15 mol). Yield: 65%; mp 314-316 °C; IR (ATR accessory) ν 3250-2900, 2800-2600, 1748, 1574, 1394, 1194, 1101 cm^{-1}; ^1H NMR (400.13 MHz, DMSO-d_6) δ 3.12 (dd, 1H, ^2J = 17.0 Hz, ^3J = 11.3 Hz), 3.31 (dd, 1H, ^2J = 17.0 Hz, ^3J = 5.0 Hz), 4.35 (s, 2H), 4.47 (dd, 1H, ^3J = 5.0, 11.3 Hz), 7.26 (s, 4H), 9.67 (brs, 2H); ^{13}C NMR (100.6 MHz, DMSO-d_6) δ 28.1, 44.0, 53.3, 126.6, 126.9, 127.6, 128.3, 128.8, 130.7, 170.1; HRMS: *m*/*z* calculated for $C_{10}H_{12}NO_2^+$ $[M+H]^+$: 178.0863, found: 178.0848.

General method for the syntheses of 7-nitro-1,2,3,4-tetrahydroisoquinoline-3-carboxylic acids ((*R*/*S*)-7)

These compounds were obtained according to the protocol described by Flynn *et al.*[3]

To a solution of carboxylic acids **(*R*/*S*)-6** (38.7 mmol) in concentrated sulfuric acid (50 mL) was added dropwise, with a careful control of the temperature (0-5 °C), a solution of sodium nitrate (42.7 mmol, 1.1 eq.) in concentrated sulfuric acid (30 mL). The reaction mixture was then stirred for 5 min and slowly poured on ice (300 g). Then the pH was increased to 8 by slow addition, at 0 °C, of a concentrated ammonium hydroxide solution (28-30 wt.%, 200 mL) (**caution! highly exothermic process**). The resulting precipitate was further filtered, washed with water (5 × 50 mL) and dried overnight under vacuum in a heating desiccator (35 °C) to give the desired nitro compounds **(*R*/*S*)-7**.

(3*S*)-7-nitro-1,2,3,4-tetrahydroisoquinoline-3-carboxylic acid ((*S*)-7)

The derivative **(*S*)-7** (light pink solid, 8.11 g, 36.5 mmol) was synthesized according to the general protocol described above starting from hydrobromide salt **(*S*)-6** (10.0 g, 38.7 mmol). The structural characterisation of compound **(*S*)-7** was highly limited by its poor solubility in commonly used NMR solvents. Yield: 94%; mp 289-291 °C (decomposition); IR (ATR accessory) ν 3100-2900, 2800-

2500, 1526, 1629, 1398, 1351, 1316, 1083 cm^{-1}; HRMS: *m*/*z* calculated for $C_{10}H_{11}N_2O_4^+$ $[M+H]^+$: 223.0713, found: 223.0727.

(3*R*)-7-nitro-1,2,3,4-tetrahydroisoquinoline-3-carboxylic acid ((*R*)-7)

The derivative **(*R*)-7** (ochre solid, 15.18 g, 68.3 mmol) was synthesized according to the general protocol described above starting from hydrobromide salt **(*R*)-6** (20.0 g, 77.5 mmol). The structural characterisation of compound **(*R*)-7** was highly limited by its poor solubility in commonly used NMR solvents. Yield: 88%; mp 289-291 °C (decomposition); IR (ATR accessory) ν 3100-2900, 2800-2500, 1630, 1540, 1395, 1353, 1317, 1082 cm^{-1}.

General method for the syntheses of ethyl 7-nitro-1,2,3,4-tetrahydroisoquinoline-3-carboxylates ((*R*/*S*)-8)

To a solution of carboxylic acids **(*R*/*S*)-7** (29.2 mmol) in anhydrous ethanol (150 mL) was slowly added, under argon, thionyl chloride (0.146 mmol, 5 eq.). The reaction mixture was then refluxed for 20 h under stirring. After cooling to room temperature, the solvent was evaporated under reduced pressure. The residue was further dissolved in an aqueous 5% sodium bisulfite solution (30 mL) and pH was adjusted to 9 by addition of an aqueous saturated sodium carbonate solution. The mixture was extracted with ethyl acetate (3 x 80 mL) and the combined organic layers were dried over anhydrous magnesium sulfate, filtered and evaporated under reduced pressure. The residue was purified by column chromatography (SiO_2, ethyl acetate/cyclohexane, 8/2, v/v) to yield the desired esters **(*R*/*S*)-8**. The latter was unstable and had to be quickly engaged in the next step.

Ethyl (3*S*)-7-nitro-1,2,3,4-tetrahydroisoquinoline-3-carboxylate ((*S*)-8)

Ester **(*S*)-8** (pink solid, 4.86 g, 19.4 mmol) was synthesized according to the general protocol described above, starting from carboxylic acid compound **(*S*)-7** (6.48 g, 29.2 mmol). Yield: 67%; R_f (SiO_2, ethyl acetate/cyclohexane, 8/2, v/v): 0.34; mp 95-97 °C (litt.[4] 94-95 °C); IR (ATR accessory) ν 3358, 1735, 1721, 1518, 1347, 1295, 1236, 1216, 1032 cm^{-1}; ^1H NMR (400.13 MHz, DMSO-d_6) δ 1.22 (t, 3H, ^3J = 7.1 Hz), 2.91 (dd, 1H, ^2J = 17.0 Hz, ^3J = 8.5 Hz), 3.07 (dd, 1H, ^2J = 17.0 Hz, ^3J = 4.8 Hz), 3.71 (dd, 1H, ^3J = 4.8, 8.5 Hz), 3.95 (d, 1H, ^2J = 16.7 Hz), 4.07 (d, 1H, ^2J = 16.7 Hz), 4.13 (q, 2H, ^3J = 7.1 Hz), 7.41 (m, 1H), 7.97 (m, 2H); ^{13}C NMR (100.6 MHz, DMSO-d_6) δ 14.1, 31.1, 46.2, 54.4, 60.4, 120.6, 120.9, 130.3, 137.5, 142.1, 145.6, 172.4; HRMS: *m*/*z* calculated for $C_{12}H_{15}N_2O_4^+$ $[M+H]^+$: 251.1026, found: 251.1029.

Ethyl (3*R*)-7-nitro-1,2,3,4-tetrahydroisoquinoline-3-carboxylate ((*R*)-8)

Ester **(*R*)-8** (pink solid, 8.31 g, 33.2 mmol) was synthesized according to the general protocol described above, starting from carboxylic acid compound **(*R*)-7** (11.61 g, 52.3 mmol). Yield: 64%; R_f (SiO_2, ethyl acetate/cyclohexane, 8/2, v/v): 0.34; mp 93-95 °C; IR (ATR accessory) ν 3358, 1735, 1720, 1519, 1346, 1295, 1237, 1216, 1032 cm^{-1}; ^1H NMR (400.13 MHz, DMSO-d_6) δ 1.21 (t, 3H, ^3J = 7.1 Hz), 2.91 (dd, 1H, ^2J = 17.2 Hz, ^3J = 8.8 Hz), 3.08 (dd, 1H, ^2J = 17.2 Hz, ^3J = 4.8 Hz), 3.72 (dd, 1H, ^3J = 4.8, 8.8 Hz), 3.96 (d, 1H, ^2J = 16.8 Hz), 4.08 (d, 1H, ^2J = 16.8 Hz), 4.13 (q, 2H, ^3J = 7.1 Hz), 7.41 (m, 1H), 7.97 (m, 2H); ^{13}C NMR (125.0 MHz, DMSO-d_6) δ 14.3, 31.8, 47.1, 55.3, 61.5, 121.4, 121.5, 130.2, 136.4, 141.2, 146.5, 172.4; HRMS: *m*/*z* calculated for $C_{12}H_{15}N_2O_4^+$ $[M+H]^+$: 251.1026, found: 251.1014.

Ethyl 7-nitroisoquinoline-3-carboxylate was observed as by-product of this reaction:

R_f (SiO_2, ethyl acetate): 0.38; mp 232-234 °C; IR (ATR accessory) ν 3000-2800, 1703, 1341, 1283, 1229 cm^{-1}; ^1H NMR (200.13 MHz, $CDCl_3$) δ 1.50 (t, 3H, ^3J = 7.1 Hz), 4.56 (q, 2H, ^3J = 7.1 Hz), 8.16 (d, 1H, ^3J = 9.0 Hz), 8.56 (dd, 1H, ^3J = 9.0 Hz, ^4J = 2.1 Hz), 8.68 (s, 1H), 9.02 (d, ^4J = 2.1 Hz), 9.56 (s, 1H); ^{13}C NMR (50.32 MHz, $CDCl_3$) δ 14.5, 62.6, 123.4, 124.3, 124.7, 128.8, 130.2, 138.3, 145.1, 147.7, 154.5, 165.1; ESI-MS: *m*/*z* calculated for $C_{12}H_{11}N_2O_4^+$ $[M+H]^+$: 247.07, found: 247.07.

General method for the syntheses of ethyl 5-iodo-7-nitro-1,2,3,4-tetrahydroisoquinoline-3-carboxylates ((*R*/*S*)-9)

These compounds were obtained according to the protocol described by Harling *et al.*[5]
To a solution of nitro compounds **(*R*/*S*)-8** (14.0 mmol) in trifluoromethanesulfonic acid (19 mL) was added *N*-iodosuccinimide (18.2 mmol, 1.3 eq.). The reaction mixture was stirred at room temperature for 7.5 h. The resulting solution was then poured into an aqueous 5% sodium bisulfite solution (50 mL), before addition of an aqueous saturated sodium bicarbonate solution (180 mL). The pH was then adjusted to 8 by addition of solid sodium bicarbonate. The solution was then extracted with ethyl acetate (3 x 100 mL) and the combined organic layers were dried over anhydrous magnesium sulfate, filtered and evaporated under vacuum. The residue was purified by column chromatography (SiO_2, ethyl acetate/cyclohexane, 8/2, v/v) to give iodinated esters **(*R*/*S*)-9**, which was quickly engaged in the next step.

Ethyl (3*S*)-5-iodo-7-nitro-1,2,3,4-tetrahydroisoquinoline-3-carboxylate ((*S*)-9)

Iodinated compound **(*S*)-9** (pink solid, 6.37 g, 16.9 mmol) was synthesized according to the general protocol described above, starting from compound **(*S*)-8** (7.00 g, 28.0 mmol). Yield: 61%; R_f (SiO_2, ethyl acetate/cyclohexane, 8/2, v/v): 0.48; mp 107-109 °C; IR (ATR accessory) ν 3363, 3200-2900, 1737, 1718, 1688, 1515, 1395, 1341, 1217, 1187 cm^{-1}; ^1H NMR (400.13 MHz, DMSO-d_6) δ 1.23 (t, 3H, ^3J = 7.1 Hz), 2.69 (dd, 1H, ^2J = 17.5 Hz, ^3J = 8.6 Hz), 2.86 (dd, 1H, ^2J = 17.5 Hz, ^3J = 5.0 Hz), 3.74 (dd, ^3J = 5.0, 8.6 Hz), 3.97 (m, 2H), 4.16 (q, 2H, ^3J = 7.1 Hz), 8.00 (d, 1H, ^4J = 2.2 Hz), 8.40 (d, 1H, 2.2 Hz), 11.1 (brs, 1H); ^{13}C NMR (100.6 MHz, DMSO-d_6) δ 14.1, 37.3, 46.9, 55.4, 60.6, 102.6, 120.8, 130.3, 139.6, 144.2, 145.7, 172.1; HRMS: *m*/*z* calculated for $C_{12}H_{14}IN_2O_4^+$ $[M+H]^+$: 376.9993, found: 376.9966.

Ethyl (3*R*)-5-iodo-7-nitro-1,2,3,4-tetrahydroisoquinoline-3-carboxylate ((*R*)-9)

Iodinated compound **(*R*)-9** (pink solid, 8.33 g, 22.1 mmol) was synthesized according to the general protocol described above, starting from compound **(*R*)-8** (8.31 g, 33.2 mmol). Yield: 67%; R_f (SiO_2, ethyl acetate/cyclohexane, 8/2, v/v): 0.48; mp 106-108 °C; IR (ATR accessory) ν 3362, 3100-2900, 1736, 1720, 1688, 1518, 1344, 1295, 1237, 1217, 1197, 1031 cm^{-1}; ^1H NMR (200.13 MHz, $CDCl_3$) δ 1.32 (t, 3H, ^3J = 7.1 Hz), 2.34 (m, 1H), 2.83 (dd, 1H, ^2J = 17.7 Hz, ^3J = 9.2 Hz), 3.07 (dd, 1H, ^2J = 17.7 Hz, ^3J = 5.0 Hz), 3.74 (dd, 1H, ^3J = 5.0, 9.2 Hz), 4.12 (m, 2H), 4.26 (q, 2H, ^3J = 7.1 Hz), 7.92 (d, 1H, ^3J = 2.2 Hz), 8.54 (d, 1H, ^3J = 2.2 Hz); ^{13}C NMR (50.32 MHz, $CDCl_3$) δ 14.3, 37.8, 47.7, 56.2, 61.7, 101.8, 121.1, 131.8, 138.3, 143.9, 146.2, 172.0; HRMS: *m*/*z* calculated for $C_{12}H_{14}IN_2O_4^+$ $[M+H]^+$: 376.9993, found: 376.9968.

General method for the syntheses of ethyl 2-acetyl-5-iodo-7-nitro-1,2,3,4-tetrahydroisoquinoline-3-carboxylates (*R*/*S*)-10

These compounds were obtained according to the protocol described by Ohta *et al.*[6]

To a solution of amines **(*R*/*S*)-9** (13.4 mmol) in anhydrous chloroform (150 mL) were successively added, under argon, anhydrous triethylamine (21.5 mmol, 1.6 eq.) and acetyl chloride (20.1 mmol, 1.5 eq.). The reaction mixture was stirred at room temperature for 4.5 h before addition of water (150 mL). After decantation, the aqueous layer was extracted with dichloromethane (100 mL) and the combined organic layers were dried over anhydrous magnesium sulfate, filtered and evaporated under reduced pressure. The residue was purified by column chromatography (SiO_2, ethyl acetate/cyclohexane, 8/2, v/v) to yield compounds **(*R*/*S*)-10**.

Ethyl (3*S*)-2-acetyl-5-iodo-7-nitro-1,2,3,4-tetrahydroisoquinoline-3-carboxylate ((*S*)-10)

Acetamide **(*S*)-10** (yellow oil, 4.86 g, 11.6 mmol) was synthesized according to the general protocol described above, starting from amine **(*S*)-9** (4.87 g, 12.9 mmol). Yield: 90%; R_f (SiO_2, ethyl acetate/cyclohexane, 8/2, v/v): 0.51; IR (ATR accessory) ν 3100-2900, 1709, 1651, 1523, 1407, 1344, 1181, 1023 cm^{-1}; ^1H NMR (400.13 MHz, $CDCl_3$) δ 1.12 (t, 3H, ^3J = 7.1 Hz), 1.19 (t, 3H*, ^3J = 7.1 Hz), 2.15 (s, 3H*), 2.23 (s, 3H), 2.96 (dd, 1H, ^2J = 17.2 Hz, ^3J = 6.6 Hz), 3.05 (dd, 1H*, ^2J = 16.9 Hz, ^3J = 6.2 Hz), 3.49 (dd, 1H, ^2J = 17.2 Hz, ^3J = 2.5 Hz), 3.58 (dd, 1H*, ^2J = 16.9 Hz, ^3J = 2.3 Hz), 4.04 (m, 4H), 4.42 (d, 1H*, ^2J = 18.0 Hz), 4.76 (m, 2H), 4.84 (dd, 1H*, ^3J = 2.3, 6.2 Hz), 5.03 (d, 1H*, ^2J = 18.0 Hz), 5.57 (dd, 1H, ^3J = 2.5, 6.6 Hz), 7.97 (m, 2H), 8.47 (d, 1H*, ^4J = 2.2 Hz), 8.49 (d, 1H, ^4J = 2.2 Hz); ^{13}C NMR (100.6 MHz, $CDCl_3$) δ 14.2 (2C), 21.7*, 22.0, 36.8, 37.4*, 43.2*, 46.2, 50.2, 55.6*, 61.9, 62.4*, 100.1*, 100.8, 121.3, 121.8*, 132.1*, 132.5, 134.8, 135.5*, 142.0*, 143.0, 146.7, 146.9*, 169.5*, 170.0, 170.4 (2C); HRMS: *m*/*z* calculated for $C_{14}H_{16}IN_2O_5^+$ $[M+H]^+$: 419.0098, found: 419.0095.
*On the NMR spectra, two rotamers were evidenced in 70% vs 30% proportions. The symbol * refers to the minor rotamer.*

Ethyl (3*R*)-2-acetyl-5-iodo-7-nitro-1,2,3,4-tetrahydroisoquinoline-3-carboxylate ((*R*)-10)

Acetamide **(*R*)-10** (yellow oil, 2.28 g, 5.45 mmol) was synthesized according to the general protocol described above, starting from amine **(*R*)-9** (2.85 g, 7.58 mmol). Yield: 72%; R_f (SiO_2, ethyl acetate/cyclohexane, 8/2, v/v): 0.51; IR (ATR accessory) ν 3050-2900, 1733, 1712, 1651, 1523, 1407, 1344, 1196, 1023 cm^{-1}; ^1H NMR (200.13 MHz, $CDCl_3$) δ 1.16 (t, 6H, ^3J = 7.1 Hz), 2.18 (s, 3H*), 2.25 (s, 3H), 3.03 (m, 2H), 3.58 (m, 2H), 4.06 (m, 4H), 4.45 (d, 1H*, ^2J = 18.0 Hz), 4.78 (m, 3H), 5.08 (d, 1H*, ^2J = 18.0 Hz), 5.62 (dd, 1H, ^3J = 2.5, 6.6 Hz), 8.00 (s, 2H), 8.57 (s, 1H*), 8.60 (s,

1H); ^{13}C NMR (100.6 MHz, $CDCl_3$) δ 14.2 (2C), 21.7*, 22.0, 36.7, 37.4*, 43.2*, 46.2, 50.8, 55.5*, 61.9, 62.3*, 100.1, 100.8, 121.3, 121.7*, 132.1*, 132.4, 134.8, 135.5*, 142.0*, 143.0, 146.7, 146.8*, 169.4*, 169.9, 170.3 (2C); HRMS: *m/z* calculated for $C_{14}H_{16}IN_2O_5^+$ $[M+H]^+$: 419.0098, found: 419.0141.
*On the NMR spectra, two rotamers were evidenced in 70% vs 30% proportions. The symbol * refers to the minor rotamer.*

General method for the syntheses of ethyl 2-acetyl-7-amino-5-iodo-1,2,3,4-tetrahydroisoquinoline-3-carboxylates ((*R*/*S*)-11)

These compounds were synthesized following a protocol adapted from Brown *et al.*[7]

To a solution of esters **(*R*/*S*)-10** (4.70 mmol) in anhydrous ethanol (130 mL) was added, under argon and stirring, tin(II) chloride (39.4 mmol, 3.5 eq.). The reaction mixture was refluxed for 1 h. After cooling to room temperature, the volume of the solution was reduced by 75% under reduced pressure and then diluted with dichloromethane (150 mL). The resulting solution was washed successively with an aqueous 2 N sodium hydroxide solution (2 × 50 mL) and brine (100 mL). The organic layers were combined, dried over anhydrous magnesium sulfate, filtered and evaporated under reduced pressure. The residue was suspended in anhydrous diethyl ether (15 mL), triturated and filtered to give the desired amino compounds **(*R*/*S*)-11**.

Ethyl (3*S*)-2-acetyl-7-amino-5-iodo-1,2,3,4-tetrahydroisoquinoline-3-carboxylate ((*S*)-11)

Amine **(*S*)-11** (light yellow solid, 128 mg, 0.33 mmol) was synthesized according to the general protocol described above, starting from nitro compound **(*S*)-10** (226 mg, 0.54 mmol). Compound **(*S*)-11** was additionally purified by column chromatography (Al_2O_3, dichloromethane/ethanol, 99/1, v/v). Yield: 61%; R_f (Al_2O_3, dichloromethane/ethanol, 99/1, v/v): 0.25; mp 152-153 °C; IR (ATR accessory) ν 3429, 3378, 3342, 3232, 3000-2800, 1722, 1648, 1627, 1606, 1405, 1371, 1335, 1291, 1195, 1023 cm^{-1}; ^{1}H NMR (400.13 MHz, DMSO-d_6) δ 1.07 (t, 3H, ^3J = 7.1 Hz), 1.08 (t, 3H*, ^3J = 7.1 Hz), 2.03 (s, 3H*), 2.13 (s, 3H), 2.81 (dd, 1H, ^2J = 15.6 Hz, ^3J = 6.0 Hz), 2.90 (dd, 1H*, ^2J = 15.7 Hz, ^3J = 5.7 Hz), 3.14 (dd, 1H, ^2J = 15.6 Hz, ^3J = 3.3 Hz), 3.22 (dd, 1H*, ^2J = 15.7 Hz, ^3J = 2.6 Hz), 4.00 (m, 4H), 4.14 (d, 1H*, ^2J = 17.2 Hz), 4.43 (d, 1H, ^2J = 15.6 Hz), 4.54 (d, 1H*, ^2J = 17.2 Hz), 4.57 (d, 1H, ^2J = 15.6 Hz), 5.03 (dd, 1H*, ^3J = 2.6, 5.6 Hz), 5.10 (dd, 1H, ^3J = 3.3, 6.0 Hz), 5.25 (brs, 4H), 6.39 (d, 1H*, ^4J = 2.0 Hz), 6.43 (d, 1H, ^4J = 2.0 Hz), 6.99 (m, 2H); ^{13}C NMR (100.6 MHz, DMSO-d_6) δ 14.1 (2C), 21.6*, 21.9, 34.7, 35.5*, 43.2*, 46.1, 52.1, 55.8*, 60.5, 61.0*, 100.8, 101.0*, 111.7, 111.8*, 121.5*, 121.8, 122.3 (2C), 134.4*, 134.8, 148.6 (2C), 169.8, 169.9*, 170.5 (2C); HRMS: *m/z* calculated for $C_{14}H_{18}IN_2O_3^+$ $[M+H]^+$: 389.0357, found: 389.0347.
*On the NMR spectra, two rotamers were evidenced in 60% vs 40% proportions. The symbol * refers to the minor rotamer.*

Ethyl (3*R*)-2-acetyl-7-amino-5-iodo-1,2,3,4-tetrahydroisoquinoline-3-carboxylate ((*R*)-11)

Amine **(*R*)-11** (yellow solid, 4.04 g, 10.4 mmol) was synthesized according to the general protocol described above, starting from nitro compound **(*R*)-10** (5.24 g, 12.5 mmol). Yield: 83%; R_f (Al_2O_3, dichloromethane/ethanol, 99/1, v/v): 0.25; mp 153-154 °C; IR (ATR accessory) ν 3429, 3342, 3230, 2979, 1721, 1627, 1606, 1404, 1371, 1335, 1292, 1195, 1022 cm^{-1}; ^{1}H NMR (200.13 MHz, $CDCl_3$) δ 1.16 (t, 3H*, ^3J = 7.1 Hz), 1.17 (t, 3H, ^3J = 7.1 Hz), 2.14 (s, 3H*), 2.22 (s, 3H), 2.92 (m, 2H), 3.34 (dd, 1H, ^2J = 16.1 Hz, ^3J = 3.1 Hz), 3.46 (dd, 1H*, ^2J = 16.1 Hz, ^3J = 3.1 Hz), 3.65 (m, 4H), 4.40 (d, 1H*, ^2J = 17.1 Hz), 4.55 (m, 2H), 4.70 (m, 1H*), 4.76 (d, 1H*, ^2J = 17.1 Hz), 4.57 (m, 2H), 5.44 (dd, 1H, ^3J = 3.2, 6.0 Hz), 6.42 (m, 1H), 6.45 (m, 1H*), 7.07 (d, 1H*, ^4J = 2.2 Hz), 7.10 (d, 1H, ^4J = 2.2 Hz); ^{13}C NMR (50.32 MHz, $CDCl_3$) δ 14.3 (2C), 22.0*, 22.2, 35.4, 36.4*, 43.9*, 46.7, 52.0, 56.6*,

61.4, 61.9*, 100.8*, 101.5, 112.7, 113.2*, 124.0*, 124.5, 125.1 (2C), 134.3, 135.0*, 146.3, 146.5, 170.5 (2C), 170.8 (2C); HRMS: *m*/*z* calculated for $C_{14}H_{18}IN_2O_3^+$ [M+H]$^+$: 389.0357, found: 389.0332.
*On the NMR spectra, two rotamers were evidenced in 60% vs 40% proportions. The symbol * refers to the minor rotamer.*

General method for the syntheses of ethyl 2-acetyl-7-hydroxy-5-iodo-1,2,3,4-tetrahydroisoquinoline-3-carboxylates ((*R*/*S*)-12)

These compounds were synthesized following a protocol adapted from Jiang *et al.*[8]

To a solution of amines **(*R*/*S*)-11** (2.14 mmol) in water (20 mL) was added an aqueous 0.5 M sulfuric acid solution (20 mL). The reaction mixture was stirred at 100 °C (external temperature) until complete dissolution of the precipitate (around 10 min). After cooling to 0 °C, sodium nitrite (3.20 mmol, 1.5 eq.) was added. The reaction mixture was stirred at 0 °C for 30 min until total consumption of starting compounds **(*R*/*S*)-11** (TLC monitoring with ninhydrin revelation: Al_2O_3, dichloromethane/ethanol, 99/1, v/v). Then urea (1.07 mmol, 0.5 eq.) was added at 0 °C and the reaction mixture was refluxed for 30 min until no more gas evolution was observed. The resulting solution was then cooled to room temperature and extracted with ethyl acetate (3 × 50 mL). The organic layers were combined, dried over anhydrous magnesium sulfate, filtered and evaporated under reduced pressure. The residue was purified by column chromatography (SiO_2, ethyl acetate) to give compounds **(*R*/*S*)-12**.

Ethyl (3*S*)-2-acetyl-7-hydroxy-5-iodo-1,2,3,4-tetrahydroisoquinoline-3-carboxylate ((*S*)-12)

Compound **(*S*)-12** (yellow solid, 2.06 g, 5.29 mmol) was synthesized according to the general protocol described above, starting from amine **(*S*)-11** (3.00 g, 7.73 mmol). Yield: 68%; R_f (SiO_2, ethyl acetate): 0.43; mp 67-69 °C; IR (ATR accessory) ν 3450-2800, 1733, 1600, 1568, 1420, 1368, 1326, 1283, 1187, 1122, 1190, 1023 cm^{-1}; ^{1}H NMR (200.13 MHz, $CDCl_3$) δ 1.17 (t, 6H, ^3J = 7.1 Hz), 2.16 (s, 3H*), 2.24 (s, 3H), 2.96 (m, 2H), 3.44 (m, 2H), 4.08 (m, 4H), 4.43 (d, 1H*, ^2J = 17.3 Hz), 4.57 (m, 2H), 4.72 (m, 1H*), 4.78 (d, 1H*, ^2J = 17.3 Hz), 5.39 (dd, 1H, ^3J = 3.3, 6.0 Hz), 6.61 (m, 2H), 7.27 (m, 2H), 8.02 (brs, 2H); ^{13}C NMR (50.32 MHz, CD_3OD) δ 14.5 (2C), 21.7*, 21.9, 36.3, 37.1*, 45.1*, 47.9, 54.2, 57.9*, 62.5, 62.9*, 100.5*, 100.8, 114.6 (2C), 125.7*, 125.8, 127.0*, 127.4, 135.8*, 136.1, 158.2 (2C), 171.7*, 172.1, 173.3, 173.5*; HRMS: *m*/*z* calculated for $C_{14}H_{17}INO_4^+$ [M+H]$^+$: 390.0197, found: 390.0206.
*On the NMR spectra, two rotamers were evidenced in 60% vs 40% proportions. The symbol * refers to the minor rotamer.*

Ethyl (3*R*)-2-acetyl-7-hydroxy-5-iodo-1,2,3,4-tetrahydroisoquinoline-3-carboxylate ((*R*)-12)

Compound **(*R*)-12** (yellow solid, 1.83 g, 4.70 mmol) was synthesized according to the general protocol described above, starting from amine **(*R*)-11** (2.83 g, 7.29 mmol). Yield: 65%; R_f (SiO_2, ethyl acetate): 0.43; mp 65-66 °C; IR (ATR accessory) ν 3450-2800, 1734, 1608, 1564, 1472, 1423, 1368, 1281, 1212, 1190, 1122, 1024 cm^{-1}; ^{1}H NMR (200.13 MHz, $CDCl_3$) δ 1.18 (t, 3H*, ^3J = 7.1 Hz), 1.19 (t, 3H, ^3J = 7.1 Hz), 2.18 (s, 3H*), 2.25 (s, 3H), 2.98 (m, 2H), 3.46 (m, 2H), 4.10 (m, 4H), 4.43 (d, 1H*, ^2J = 17.4 Hz), 4.59 (m, 2H), 4.73 (m, 1H*), 5.25 (d, 1H*, ^2J = 17.4 Hz), 5.43 (dd, 1H, ^3J = 3.1, 6.0 Hz), 6.62 (m, 2H), 7.28 (m, 2H); ^{13}C NMR (50.32 MHz, $CDCl_3$) δ 14.2 (2C), 21.8*, 22.0, 35.4, 36.2*, 44.2*, 46.9, 52.6, 56.7*, 61.9, 62.2*, 100.2*, 100.5, 114.6 (2C), 125.7*, 125.8, 127.0*, 127.4, 135.8*, 136.1, 158.2 (2C), 171.7*, 172.1, 173.3, 173.5*; HRMS: *m*/*z* calculated for $C_{14}H_{17}INO_4^+$ [M+H]$^+$: 390.0197, found: 390.0169.
*On the NMR spectra, two rotamers were evidenced in 67% vs 33% proportions. The symbol * refers to the minor rotamer.*

General method for the syntheses of ethyl 7-hydroxy-5-iodo-1,2,3,4-tetrahydroisoquinoline-3-carboxylates ((*R*/*S*)-13)

A solution of derivatives **(*R*/*S*)-12** (1.05 mmol) in concentrated hydrochloric acid solution (37 wt.%, 12 mL) was refluxed for 7 h. After cooling to room temperature, the reaction mixture was concentrated under reduced pressure. The residue was co-evaporated with anhydrous ethanol (10 mL) and then diluted in anhydrous ethanol (20 mL) before addition of a concentrated hydrochloric acid solution (37 wt.%, 0.55 mL). The reaction mixture was then refluxed for 15 h. After cooling to room temperature, the solvents were evaporated under reduced pressure and the residue was dissolved in water (25 mL). The pH of the solution was adjusted to 8 by addition of an aqueous saturated sodium bicarbonate solution. The resulting solution was then extracted with ethyl acetate (4 x 25 mL). The organic layers were combined, dried over anhydrous magnesium sulfate, filtered and evaporated under reduced pressure. The residue was purified by column chromatography (SiO_2, ethyl acetate) to give esters **(*R*/*S*)-13**.

Ethyl (3*S*)-7-hydroxy-5-iodo-1,2,3,4-tetrahydroisoquinoline-3-carboxylate ((*S*)-13)

Compound **(*S*)-13** (off-white solid, 1.54 g, 4.44 mmol) was synthesized according to the general protocol described above, starting from acetamide **(*S*)-12** (2.06 g, 5.29 mmol). Yield: 84%; R_f (SiO_2, ethyl acetate): 0.24; mp 148-150 °C; IR (ATR accessory) ν 3260, 1733, 1602, 1557, 1454, 1425, 1274, 1205, 1178, 1106, 1024 cm^{-1}; ^1H NMR (200.13 MHz, CD_3OD) δ 1.31 (t, 3H, ^3J = 7.2 Hz), 2.65 (dd, 1H, ^2J = 16.5 Hz, ^3J = 10.3 Hz), 2.94 (dd, 1H, ^2J = 16.5 Hz, ^3J = 5.2 Hz), 3.67 (dd, 1H, ^3J = 5.2, 10.3 Hz), 3.92 (s, 2H), 4.25 (q, 2H, ^3J = 7.2 Hz), 6.54 (d, 1H, ^4J = 2.3 Hz), 7.20 (d, 1H, ^4J = 2.3 Hz); ^{13}C NMR (50.32 MHz, CD_3OD) δ 14.5, 37.2, 48.2, 57.6, 62.1, 102.4, 113.8, 125.4, 127.1, 138.0, 157.0, 173.5; HRMS: *m*/*z* calculated for $C_{12}H_{15}INO_3^+$ $[M+H]^+$: 348.0091, found: 348.0076.

Ethyl (3*R*)-7-hydroxy-5-iodo-1,2,3,4-tetrahydroisoquinoline-3-carboxylate ((*R*)-13)

Compound **(*R*)-13** (off-white solid, 1.24 g, 3.57 mmol) was synthesized according to the general protocol described above, starting from acetamide **(*R*)-12** (1.71 g, 4.39 mmol). Yield: 81%; R_f (SiO_2, ethyl acetate): 0.24; mp 154-156 °C; IR (ATR accessory) ν 3251, 2980, 2895, 2700-2400, 1734, 1593, 1432, 1233, 1189, 1106, 1029 cm^{-1}; ^1H NMR (500.13 MHz, CD_3OD) δ 1.32 (t, 3H, ^3J = 7.2 Hz), 2.69 (dd, 1H, ^2J = 16.5 Hz, ^3J = 10.2 Hz), 2.96 (dd, 1H, ^2J = 16.5 Hz, ^3J = 4.9 Hz), 3.68 (dd, 1H, ^3J = 10.2, 4.9 Hz), 3.96 (m, 2H), 4.25 (m, 2H), 6.48 (brs, 1H), 7.22 (d, 1H, ^4J = 1.8 Hz); ^{13}C NMR (50.32 MHz, CD_3OD) δ 14.5, 37.5, 48.5, 57.9, 62.3, 102.5, 114.1, 125.5, 127.4, 138.4, 157.5, 173.8; HRMS: *m*/*z* calculated for $C_{12}H_{15}INO_3^+$ $[M+H]^+$: 348.0091, found: 348.0076.

General method for the syntheses of 7-hydroxy-5-iodo-1,2,3,4-tetrahydroisoquinoline-3-carboxylic acids ((*S*)-14, 5-iodo-L-TIC(OH)) and ((*R*)-14, 5-iodo-D-TIC(OH))

These compounds were synthesized following the protocol of Moreno-Manas *et al.*[9]

A solution of esters **(*R*/*S*)-12** (0.36 mmol) in a 6 M hydrochloric acid solution (5 mL) was refluxed under stirring for 4.5 h. After cooling to room temperature, an aqueous 5% potassium bicarbonate solution was added to reach pH 6-7. The reaction mixture was stored at 4 °C for 16 h. The precipitate formed was then filtered and washed with anhydrous diethyl ether (3 x 1.5 mL) to yield **(*R*/*S*)-14**.

(3*S*)-7-hydroxy-5-iodo-1,2,3,4-tetrahydroisoquinoline-3-carboxylic acid ((*S*)-14, 5-iodo-L-TIC(OH))

Compound **(*S*)-14** (yellow solid, 50 mg, 0.16 mmol) was synthesized according to the general protocol described above, starting from compound **(*S*)-12** (140 mg, 0.36 mmol). Yield: 44%; mp 255-257 °C; IR (ATR accessory) ν 3348, 3000-2700, 1733, 1602, 1403, 1217, 1198 cm^{-1}; ^1H NMR (400.13 MHz, DMSO-d_6) δ 2.80 (dd, 1H, ^2J = 16.6 Hz, ^3J = 11.3 Hz), 3.07 (dd, 1H, ^2J = 16.6 Hz, ^3J = 5.1 Hz), 4.23 (s, 2H), 4.38 (m, 1H), 6.72 (d, 1H, ^4J = 2.0 Hz), 7.31 (d, 1H, ^4J = 2.0 Hz), 9.89 (brs, 2H), 10.0 (brs, 1H); ^{13}C NMR (100.6 MHz, DMSO-d_6) δ 33.5, 44.2, 54.1, 101.6, 113.4, 123.3, 125.1, 131.1, 156.8, 169.7; HRMS: *m*/*z* calculated for $C_{10}H_{11}INO_3^+$ $[M+H]^+$: 319.9778, found: 319.9782; e.e.(Method C): t_R = 12.8 min, 98.8%.

(3*R*)-7-hydroxy-5-iodo-1,2,3,4-tetrahydroisoquinoline-3-carboxylic acid ((*R*)-14, 5-iodo-D-TIC(OH))

Compound **(*R*)-14** (yellow solid, 58 mg, 0.18 mmol) was synthesized according to the general protocol described above, starting from compound **(*R*)-12** (300 mg, 0.77 mmol) with a reaction time of 4 h. Yield: 23%; mp 252-254 °C; IR (ATR accessory) ν 3400-2700, 1607, 1434, 1404 cm^{-1}; ^1H NMR (200.13 MHz, DMSO-d_6) δ 2.64 (dd, 1H, ^2J = 16.7 Hz, ^3J = 10.9 Hz), 3.00 (dd, 1H, ^2J = 16.7 Hz, ^3J = 5.7 Hz), 4.09 (m, 2H), 6.68 (s, 1H), 7.30 (s, 1H), 10.10 (brs, 1H); ^{13}C NMR (50.32 MHz, DMSO-d_6) δ 35.0, 44.7, 56.8, 102.1, 113.7, 125.1, 125.8, 132.1, 156.6, 169.6; HRMS: *m*/*z* calculated for $C_{10}H_{11}INO_3^+$ $[M+H]^+$: 319.9778, found: 319.9788; e.e.(Method C): t_R = 23.4 min, 100%.

General method for the syntheses of 7-hydroxy-6,8-diiodo-1,2,3,4-tetrahydroisoquinoline-3-carboxylic acids, hydrobromide salts ((*R*/*S*)-15)

To a solution of commercial hydrated 3,5-D/L-diiodotyrosines (max. of water 10 wt.%, 10.0 g, 23.1 mmol) in trifluoroacetic acid (130 mL) were successively added a solution of hydrobromic acid in acetic acid (33 wt.%, 30 mL) and paraformaldehyde (0.90 g). The reaction mixture was heated at 80 °C with vigorous stirring for 1 h. After cooling to room temperature, a second addition of paraformaldehyde (0.14 g) was performed. Then, the mixture was heated at 80 °C for 15 h. After cooling to room temperature, the solution was placed in an ice bath for 30 min. The resulting precipitate was filtered under reduced pressure, washed with anhydrous diethyl ether (4 × 20 mL) and dried under vacuum to give tetrahydroquinolines **(*R*/*S*)-15**.

(3*S*)-7-Hydroxy-6,8-diiodo-1,2,3,4-tetrahydroisoquinoline-3-carboxylic acid, hydrobromide salt ((*S*)-15)

Compound **(*S*)-15** (grey solid, 10.3 g, 19.6 mmol) was synthesized according to the general protocol described above, starting from hydrated 3,5-diiodo-L-tyrosine. Yield: 85%; mp 321-323 °C; IR (ATR accessory) ν 3200-2400, 1751, 1173 cm^{-1}; ^1H NMR (400.13 MHz, DMSO-d_6) δ 3.03 (dd, 1H, ^2J = 16.9 Hz, ^3J = 11.5 Hz), 3.21 (dd, 1H, ^2J = 16.9 Hz, ^3J = 4.7 Hz), 4.03 (d, 1H, ^2J = 16.2 Hz), 4.15 (d, 1H, ^2J = 16.2 Hz), 4.34 (dd, 1H, ^3J = 4.7, 11.5 Hz), 7.74 (s, 1H), 9.69 (brs, 3H); ^{13}C NMR (100.6 MHz, DMSO-d_6) δ 27.1, 50.4, 52.7, 86.4, 90.9, 126.9, 131.9, 138.9, 154.5, 169.8; HRMS: *m*/*z* calculated for $C_{10}H_{10}I_2NO_3^+$ $[M+H]^+$: 445.8745, found: 445.8748.

(3*R*)-7-Hydroxy-6,8-diiodo-1,2,3,4-tetrahydroisoquinoline-3-carboxylic acid, hydrobromide salt ((*R*)-15)

Compound **(*R*)-15** (pale brownish solid, 9.7 g, 18.5 mmol) was synthesized according to the general protocol described above, starting from hydrated 3,5-diiodo-D-tyrosine. Yield: 80%; mp 317-319 °C; IR (ATR accessory) ν 3000-2500, 2800-2300, 1750, 1174 cm^{-1}; ^{1}H NMR (400.13 MHz, DMSO-d_6) δ 3.04 (dd, 1H, ^{2}J = 16.6 Hz, ^{3}J = 11.7 Hz), 3.20 (dd, 1H, ^{2}J = 16.6 Hz, ^{3}J = 4.6 Hz), 4.04 (d, 1H, ^{2}J = 16.2 Hz), 4.14 (d, 1H, ^{2}J = 16.2 Hz), 4.35 (dd, 1H, ^{3}J = 4.6, 11.3 Hz), 7.73 (s, 1H), 9.68 (brs, 3H); ^{13}C NMR (100.6 MHz, DMSO-d_6) δ 27.1, 50.4, 52.7, 86.5, 91.0, 126.9, 131.9, 138.8, 154.5, 169.8; HRMS: *m*/*z* calculated for $C_{10}H_{10}I_2NO_3^+$ $[M+H]^+$: 445.8745, found: 445.8723.

General method for the syntheses of ethyl 7-hydroxy-6,8-diiodo-1,2,3,4-tetrahydroisoquinoline-3-carboxylates, hydrobromide salts ((*R*/*S*)-16)

To a solution of carboxylic acids **(*R*/*S*)-15** (10.0 g, 19.0 mmol) in ethanol (800 mL) was added a concentrated hydrobromic acid solution (48 wt.%, 20 mL). The mixture was refluxed for 16 h. After cooling to room temperature, the solvent was evaporated under reduced pressure and the residue was suspended in ethyl acetate (15 mL). The resulting precipitate was filtered, washed with ethyl acetate (5 mL) and dried under reduced pressure to give esters **(*R*/*S*)-16**.

Ethyl (3*S*)-7-hydroxy-6,8-diiodo-1,2,3,4-tetrahydroisoquinoline-3-carboxylate, hydrobromide salt ((*S*)-16)

Compound **(*S*)-16** (white solid, 7.67 g, 13.8 mmol) was synthesized according to the general protocol described above, starting from carboxylic acid **(*S*)-15**. Yield: 73%; mp 214-216 °C (decomposition); IR (ATR accessory) ν 3375, 2901, 2753, 2619, 2497, 1721, 1261 cm^{-1}; ^{1}H NMR (400.13 MHz, DMSO-d_6) δ 1.26 (t, 3H, ^{3}J = 7.1 Hz), 3.05 (dd, 1H, ^{2}J = 16.8 Hz, ^{3}J = 11.4 Hz), 3.20 (dd, 1H, ^{2}J = 16.8 Hz, ^{3}J = 4.8 Hz), 4.03 (d, 1H, ^{2}J = 16.2 Hz), 4.17 (d, 1H, ^{2}J = 16.2 Hz), 4.28 (q, 2H, ^{3}J = 7.1 Hz), 4.46 (dd, 1H, ^{3}J = 4.8, 11.4 Hz), 7.73 (s, 1H), 9.72 (brs, 3H); ^{13}C NMR (100.6 MHz, DMSO-d_6) δ 14.0, 27.1, 50.4, 52.8, 62.3, 86.4, 90.9, 126.6, 132.0, 138.8, 154.5, 168.3; HRMS: *m*/*z* calculated for $C_{12}H_{14}I_2NO_3^+$ $[M+H]^+$: 473.9058, found: 473.9068.

Ethyl (3*R*)-7-hydroxy-6,8-diiodo-1,2,3,4-tetrahydroisoquinoline-3-carboxylate, hydrobromide salt ((*R*)-16)

Compound **(*R*)-16** (ochre solid, 8.6 g, 15.6 mmol) was synthesized according to the general protocol described above, starting from carboxylic acid **(*R*)-15**. Yield: 82%; mp 220-222 °C; IR (ATR accessory) ν 3000-2400, 2250-2700, 1722, 1261 cm^{-1}; ^{1}H NMR (400.13 MHz, DMSO-d_6) δ 1.26 (t, 3H, ^{3}J = 7.1 Hz), 3.04 (dd, 1H, ^{2}J = 16.8 Hz, ^{3}J = 11.5 Hz), 3.20 (dd, 1H, ^{2}J = 16.8 Hz, ^{3}J = 4.8 Hz), 4.03 (d, 1H, ^{2}J = 16.2 Hz), 4.17 (d, 1H, ^{2}J = 16.2 Hz), 4.28 (q, 2H, ^{3}J = 7.1 Hz), 4.45 (dd, 1H, ^{3}J = 4.8, 11.5 Hz), 7.73 (s, 1H), 9.69 (brs, 2H); ^{13}C NMR (100.6 MHz, DMSO-d_6) δ 14.0, 27.1, 50.4, 52.7, 62.3, 86.6, 91.1, 126.6, 131.9, 138.8, 154.5, 168.2; HRMS: *m*/*z* calculated for $C_{12}H_{14}I_2NO_3^+$ $[M+H]^+$: 473.9058, found: 473.9021.

General method for the syntheses of ethyl 7-hydroxy-6-iodo-1,2,3,4-tetrahydroisoquinoline-3-carboxylates ((*R*/*S*)-17)

To a solution of **(*R*/*S*)-16** (5.42 mmol) in ethanol (55 mL) were successively added a concentrated hydrobromic acid solution (48 wt.%, 5.0 mL) dropwise and zinc dust (1.42 g, 21.7 mmol, 4 eq.) in small portions over 1.5 h and under vigorous stirring. After stirring 2 h at room temperature, water (100 mL) and brine (100 mL) were successively added. The mixture was extracted with ethyl acetate (3 × 200 mL) and the combined organic layers were washed with an aqueous saturated sodium bicarbonate solution (150 mL). Then, the organic layer was dried over anhydrous magnesium sulfate, filtered and evaporated under reduced pressure. The solid was suspended in ethyl acetate (5 mL), filtered and dried under vacuum to give compounds **(*R*/*S*)-17**.

Ethyl (3*S*)-7-hydroxy-6-iodo-1,2,3,4-tetrahydroisoquinoline-3-carboxylate ((*S*)-17)

Compound **(*S*)-17** (pale yellow solid, 1.37 g, 3.95 mmol) was synthesized according to the general protocol described above, starting from diiodinated compound **(*S*)-16** (3.00 g, 5.42 mmol). Yield: 73%; mp 191-193 °C; R_f (SiO_2, dichloromethane/ethanol, 98/2, v/v): 0.20; IR (ATR accessory) ν 3252, 1729, 1271 cm^{-1}; ^1H NMR (400.13 MHz, DMSO-d_6) δ 1.20 (t, 3H, ^3J = 7.0 Hz), 2.66 (dd, 1H, ^2J = 15.8 Hz, ^3J = 9.4 Hz), 2.81 (dd, 1H, ^2J = 15.8 Hz, ^3J = 4.6 Hz), 3.55 (dd, 1H, ^3J = 4.6, 9.4 Hz), 3.74 (d, 1H, ^2J = 16.4 Hz), 3.83 (d, 1H, ^2J = 16.4 Hz), 4.11 (q, 2H, ^3J = 7.0 Hz), 6.53 (s, 1H), 7.40 (s, 1H), 10.02 (brs, 1H); ^{13}C NMR (100.6 MHz, DMSO-d_6) δ 14.1, 29.8, 46.3, 55.1, 60.2, 81.7, 111.9, 126.3, 136.9, 138.6, 154.4, 172.8; HRMS: *m*/*z* calculated for $C_{12}H_{15}INO_3^+$ [M+H]$^+$: 348.0091, found: 348.0094.

Ethyl (3*R*)-7-hydroxy-6-iodo-1,2,3,4-tetrahydroisoquinoline-3-carboxylate ((*R*)-17)

Compound **(*R*)-17** (pale pinkish solid, 1.22 g, 3.54 mmol) was synthesized according to the general protocol described above, starting from diiodinated compound **(*S*)-16** (3.50 g, 6.32 mmol). Yield: 56%; mp 189-191 °C; R_f (SiO_2, dichloromethane/ethanol, 98/2, v/v): 0.20; IR (ATR accessory) ν 3251, 1727, 1271 cm^{-1}; ^1H NMR (400.13 MHz, DMSO-d_6) δ 1.20 (t, 3H, ^3J = 7.0 Hz), 2.66 (dd, 1H, ^2J = 15.5 Hz, ^3J = 9.4 Hz), 2.81 (dd, 1H, ^2J = 15.8 Hz, ^3J = 4.6 Hz), 3.55 (dd, 1H, ^3J = 4.6, 9.4 Hz), 3.75 (d, 1H, ^2J = 16.3 Hz), 3.83 (d, 1H, ^2J = 16.3 Hz), 4.11 (q, 2H, ^3J = 7.0 Hz), 6.53 (s, 1H), 7.40 (s, 1H), 10.00 (brs, 1H); ^{13}C NMR (125.77 MHz, DMSO-d_6) δ 14.1, 29.7, 46.3, 55.1, 60.2, 81.6, 111.9, 126.3, 136.9, 138.6, 154.4, 172.7; HRMS: *m*/*z* calculated for $C_{12}H_{15}INO_3^+$ [M+H]$^+$: 348.0091, found: 348.0101.

General method for the syntheses of ethyl 7-hydroxy-1,2,3,4-tetrahydroisoquinoline-3-carboxylates ((*R*/*S*)-19)

To a solution of diiodinated compounds **(*R*/*S*)-16** (1.99 mmol) in ethanol (65 mL) were successively added triethylamine (0.64 mL, 4.57 mmol, 2.3 eq.) and palladium on activated charcoal 10% (72 mg). After stirring under hydrogen atmosphere for 28 h, the mixture was filtered on a pad of celite 545®, washed with ethanol (2 × 5 mL). The filtrate was evaporated under reduced pressure and the residue was dissolved in dichloromethane (27 mL). The resulting solution was washed successively with brine (5 mL) and a saturated aqueous solution of sodium bicarbonate (2 × 10 mL). The organic layer was dried over anhydrous magnesium sulfate, filtered and evaporated under reduced pressure to give compounds **(*R*/*S*)-19**.

Ethyl (3*S*)-7-hydroxy-1,2,3,4-tetrahydroisoquinoline-3-carboxylate ((*S*)-19)

Compound **(*S*)-19** (pale yellow solid, 0.28 g, 1.27 mmol) was synthesized according to the general protocol described above, starting from diiodinated compound **(*S*)-16** (1.10 g, 1.99 mmol). Yield: 64%; mp 225-227 °C, IR (ATR accessory) ν 3128, 3000-2400, 1746, 1219 cm^{-1}; ^{1}H NMR (400.13 MHz, CD_3OD) δ 1.20 (t, 3H, ^3J = 7.1 Hz), 2.72 (dd, 1H, ^2J = 15.8 Hz, ^3J = 10.3 Hz), 2.88 (dd, 1H, ^2J = 15.8 Hz, ^3J = 4.6 Hz), 3.55 (dd, 1H, ^3J = 4.6, 10.3 Hz), 3.83 (d, 1H, ^2J = 16.0 Hz), 3.89 (d, 1H, ^2J = 16.0 Hz), 4.13 (q, 2H, ^3J = 7.1 Hz), 6.40 (d, 1H, ^4J = 2.5 Hz), 6.52 (dd, 1H, ^3J = 8.3 Hz, ^4J = 2.5 Hz), 6.84 (d, 1H, ^3J = 8.3 Hz); ^{13}C NMR (100.6 MHz, CD_3OD) δ 14.4, 28.8, 45.6, 55.8, 63.9, 113.6, 117.0, 121.6, 129.5, 131.2, 158.0, 169.7; HRMS: *m*/*z* calculated for $C_{12}H_{16}NO_3^+$ [M+H]$^+$: 222.1125, found: 222.1120.

Ethyl (3*R*)-7-hydroxy-1,2,3,4-tetrahydroisoquinoline-3-carboxylate ((*R*)-19)

Compound **(*R*)-19** (pale yellow solid, 1.26 g, 5.68 mmol) was synthesized according to the general protocol described above, starting from diiodinated compound **(*R*)-16** (5.00 g, 9.02 mmol). **(*R*)-19** was purified by column chromatography (SiO_2, dichloromethane/ethanol, 97/3, v/v). Yield: 63%; mp 152-154 °C; R_f (SiO_2, dichloromethane/ethanol, 97/3, v/v): 0.05; IR (ATR accessory) ν 3282, 3000-2400, 1730, 1467, 1199 cm^{-1}; ^{1}H NMR (400.13 MHz, CD_3OD) δ 1.29 (t, 3H, ^3J = 7.1 Hz), 2.83 (dd, 1H, ^2J = 15.9 Hz, ^3J = 10.3 Hz), 2.98 (dd, 1H, ^2J = 15.9 Hz, ^3J = 4.6 Hz), 3.66 (dd, 1H, ^3J = 4.6, 10.3 Hz), 3.93 (d, 1H, ^2J = 16.0 Hz), 3.99 (d, 1H, ^2J = 16.0 Hz), 4.22 (q, 2H, ^3J = 7.1 Hz), 6.48 (d, 1H, ^4J = 2.2 Hz), 6.60 (dd, 1H, ^3J = 8.3, ^4J = 2.2 Hz), 6.93 (d, 1H, ^3J = 8.3 Hz); ^{13}C NMR (100.6 MHz, CD_3OD) δ 14.5, 31.6, 47.9, 57.1, 62.1, 113.3, 115.0, 124.8, 131.0, 136.3, 156.8, 174.2; HRMS: *m*/*z* calculated for $C_{12}H_{16}NO_3^+$ [M+H]$^+$: 222.1125, found: 222.1121.

General method for the syntheses of ethyl 7-hydroxy-8-iodo-1,2,3,4-tetrahydroisoquinoline-3-carboxylates ((*R*/*S*)-20)

To a solution of compounds **(*R*/*S*)-19** (300 mg, 1.36 mmol) in dichloromethane (300 mL) placed in an ultrasound bath was added *N*-iodosuccinimide (101 mg, 0.45 mmol, 0.33 eq.). The mixture was sonicated for 30 s and immediately placed in an ice bath before addition of cold water (30 mL). After decantation, the organic layer was washed with water (30 mL), dried over anhydrous magnesium sulfate, filtered and evaporated under reduced pressure. The residue was purified by column chromatography (SiO_2, dichloromethane/ethanol, 99/1, v/v). Then, the collected fractions, containing traces of *N*-hydroxysuccinimide, were evaporated and dissolved in ethyl acetate (200 mL) before extraction with a 5% hydrochloric acid solution (4 × 25 mL). The pH of the solution was adjusted to 7 by addition of an aqueous saturated solution of sodium hydrogen carbonate. After extraction with ethyl acetate (4 × 25 mL), the combined organic layers were dried over anhydrous magnesium sulfate, filtered and evaporated under reduced pressure to give compounds **(*R*/*S*)-20**.

Ethyl (3*S*)-7-hydroxy-8-iodo-1,2,3,4-tetrahydroisoquinoline-3-carboxylate ((*S*)-20)

Compound **(*S*)-20** (pale yellow solid, 117 mg, 0.34 mmol) was synthesized according to the general protocol described above, starting from compound **(*S*)-19**. Yield: 75%; R_f (SiO_2, dichloromethane/ethanol, 99/1, v/v): 0.29; mp 134-136 °C; IR (ATR accessory) ν 2976, 1726, 1293, 1020 cm^{-1}; ^{1}H NMR (500.13 MHz, CD_3OD) δ 1.29 (t, 3H, ^3J = 7.1 Hz), 2.88 (dd, 1H, ^2J = 15.8 Hz, ^3J = 10.0 Hz), 2.97 (dd, 1H, ^2J = 15.8 Hz, ^3J = 4.6 Hz), 3.63 (dd, 1H, ^3J = 4.6, 10.0 Hz), 3.77 (d, 1H, ^2J =

16.6 Hz), 4.06 (d, 1H, ^2J = 16.6 Hz), 4.22 (q, 2H, ^3J = 7.1 Hz), 6.69 (d, 1H, ^3J = 8.2 Hz), 6.97 (d, 1H, ^3J = 8.2 Hz); ^{13}C NMR (125.77 MHz, CD_3OD) δ 14.5, 31.8, 54.6, 56.5, 62.2, 88.3, 113.8, 126.9, 131.0, 138.4, 156.3, 173.9; HRMS: *m*/*z* calculated for $C_{12}H_{15}INO_3^+$ $[M+H]^+$: 348.0091, found: 348.0095.

Ethyl (3*R*)-7-hydroxy-8-iodo-1,2,3,4-tetrahydroisoquinoline-3-carboxylate ((*R*)-20)

Compound **(*R*)-20** (pale yellow solid, 98 mg, 0.28 mmol) was synthesized according to the general protocol described above, starting from compound **(*R*)-19**. Yield: 62%; R_f (SiO_2, dichloromethane/ethanol, 99/1, v/v) 0.32; mp 131-133 °C; IR (ATR accessory) ν 2980, 1718, 1309, 1024 cm^{-1}; ^{1}H NMR (500.13 MHz, CD_3OD) δ 1.29 (t, 3H, ^3J = 7.1 Hz), 2.87 (dd, 1H, ^2J = 15.7 Hz, ^3J = 10.0 Hz), 2.97 (dd, 1H, ^2J = 15.8 Hz, ^3J = 4.6 Hz), 3.63 (dd, 1H, ^3J = 4.6, 10.0 Hz), 3.77 (d, 1H, ^2J = 16.6 Hz), 4.06 (d, 1H, ^2J = 16.6 Hz), 4.22 (q, 2H, ^3J = 7.1 Hz), 6.69 (d, 1H, ^3J = 8.2 Hz), 6.97 (d, 1H, ^3J = 8.2 Hz); ^{13}C NMR (125.77 MHz, CD_3OD) δ 14.5, 31.8, 54.7, 56.5, 62.2, 88.3, 113.8, 127.0, 131.0, 138.4, 156.3, 173.9; HRMS: *m*/*z* calculated for $C_{12}H_{15}INO_3^+$ $[M+H]^+$: 348.0091, found: 348.0089.

General method for the syntheses of 1,2,3,4-tetrahydro-7-hydroxy-iodoisoquinoline-3-carboxylic acids ((*R*/*S*)-18, 6-iodo-TIC(OH)), and ((*R*/*S*)-21, 8-iodo-TIC(OH))

These compounds were synthesized following the protocol of Azukizawa *et al.*[10]

To a solution of esters **(*R*/*S*)-17** or **(*R*/*S*)-20** (0.42 mmol) in a mixture of tetrahydrofuran and methanol (3/1, v/v, 3 mL) was added an aqueous 1 M lithium hydroxide solution (1 mL). The reaction mixture was stirred at room temperature for 1 h before evaporation of the methanol under reduced pressure. Then a 1 M hydrochloric acid solution (0.8 mL) was added to adjust the pH to 6/7. The mixture was stored at 4 °C for 2 h. The precipitate formed was filtered, dried overnight in a vacuum desiccator to yield the corresponding carboxylic acids **(*R*/*S*)-18** or **(*R*/*S*)-21**.

(3*S*)-1,2,3,4-tetrahydro-7-hydroxy-6-iodoisoquinoline-3-carboxylic acid ((*S*)-18, 6-iodo-L-TIC(OH))

Compound **(*S*)-18** (pale yellow solid, 389 mg, 1.22 mmol) was synthesized according to the general protocol described above, starting from ester **(*S*)-17** (738 mg, 2.13 mmol). Yield: 57%; mp 261-263 °C; IR (ATR accessory) ν 3000-2400, 2250-2700, 1641, 1558, 1403, 1286 cm^{-1}; ^{1}H NMR (400.13 MHz, DMSO-*d*6) δ 2.79 (dd, 1H, ^2J = 16.6 Hz, ^3J = 11.0 Hz), 3.05 (dd, 1H, ^2J = 16.6 Hz, ^3J = 4.8 Hz), 3.54 (dd, 1H, ^3J = 4.8, 11.0 Hz), 4.06 (s, 2H), 6.68 (s, 1H), 7.53 (s, 1H), 10.49 (brs, 1H); ^{13}C NMR (100.6 MHz, DMSO-*d*6) δ 27.7, 43.7, 54.8, 83.9, 112.1, 124.8, 130.5, 138.6, 155.2, 169.7; HRMS: *m*/*z* calculated for $C_{10}H_{11}INO_3^+$ $[M+H]^+$: 319.9778, found: 319.9762; e.e. (Method A): t_R = 6.83 min, 100%.

(3*R*)-1,2,3,4-tetrahydro-7-hydroxy-6-iodoisoquinoline-3-carboxylic acid ((*R*)-18, 6-iodo-D-TIC(OH))

Compound **(*R*)-18** (yellow solid, 98 mg, 0.31 mmol) was synthesized according to the general protocol described above, starting from ester **(*R*)-17** (170 mg, 0.49 mmol). Yield: 63%; mp 275-277 °C; IR (ATR accessory) ν 2800-3300, 2500-2700, 1595, 1397 cm^{-1}; ^{1}H NMR (400.13 MHz, DMSO-*d*6) δ 2.78 (dd, 1H, ^2J = 16.7 Hz, ^3J = 11.1 Hz), 3.04 (dd, 1H, ^2J = 16.7 Hz, ^3J = 4.7 Hz), 3.50 (dd, 1H,

^3J = 4.7, 11.1 Hz), 4.05 (s, 2H), 6.72 (s, 1H), 7.52 (s, 1H), 10.73 (brs, 1H); ^{13}C NMR (100.6 MHz, DMSO-d_6) δ 28.2, 43.8, 55.6, 83.8, 112.3, 125.5, 130.9, 138.5, 155.2, 169.5; HRMS: *m/z* calculated for $C_{10}H_{11}INO_3^+$ [M+H]$^+$: 319.9778, found: 319.9785; e.e. (Method A): t_R = 11.56 min, 100%.

(3*S*)-1,2,3,4-tetrahydro-7-hydroxy-8-iodoisoquinoline-3-carboxylic acid ((*S*)-21, 8-iodo-L-TIC(OH))

Compound **(*S*)-21** (pale yellow solid, 75 mg, 0.24 mmol) was synthesized according to the general protocol described above, starting from ester **(*S*)-20** (145 mg, 0.42 mmol). Yield: 57%; mp 216-218 °C; IR (ATR accessory) ν 2400-3000, 2250-2700, 1598, 1386, 1296 cm^{-1}; ^{1}H NMR (400.13 MHz, CD_3OD) δ 3.06 (dd, 1H, ^2J = 16.3 Hz, ^3J = 11.7 Hz), 3.30 (m, 1H), 3.80 (dd, 1H, ^3J = 4.8, 11.7 Hz), 4.14 (d, 1H, ^2J = 16.1 Hz), 4.35 (d, 1H, ^2J = 16.1 Hz), 6.81 (d, 1H, ^3J = 8.3 Hz), 7.10 (d, 1H, ^3J = 8.3 Hz); ^{13}C NMR (50.32 MHz, DMSO-d_6) δ 28.2, 50.6, 53.8, 87.7, 114.8, 123.8, 130.6, 131.6, 156.1, 170.4; HRMS: *m/z* calculated for $C_{10}H_{11}INO_3^+$ [M+H]$^+$: 319.9778, found: 319.9770; e.e. (Method A): t_R = 7.23 min, 100%.

(3*R*)-1,2,3,4-tetrahydro-7-hydroxy-8-iodoisoquinoline-3-carboxylique ((*R*)-21, 8-iodo-D-TIC(OH))

Compound **(*R*)-21** (yellow solid, 57 mg, 0.18 mmol) was synthesized according to the general protocol described above, starting from ester **(*R*)-20** (137 mg, 0.39 mmol). Yield: 46%; mp 214-216 °C; IR (ATR accessory) ν 3000-3800, 2938, 1621, 1427 cm^{-1}; ^{1}H NMR (200.13 MHz, D_2O) δ 3.07 (dd, 1H, ^2J = 17.0 Hz, ^3J = 10.9 Hz), 3.24 (dd, 1H, ^2J = 17.0 Hz, ^3J = 5.2 Hz), 4.11 (d, 1H, ^2J = 16.3 Hz), 4.22 (dd, 1H, ^3J = 5.2, 10.9 Hz), 4.37 (d, 1H, ^2J = 16.3 Hz), 6.75 (d, 1H, ^3J = 8.4 Hz), 7.03 (d, 1H, ^3J = 8.4 Hz); ^{13}C NMR (50.32 MHz, DMSO-d_6) δ 28.1, 50.3, 53.2, 87.8, 114.5, 123.5, 130.2, 131.6, 156.2, 170.1; HRMS: *m/z* calculated for $C_{10}H_{11}INO_3^+$ [M+H]$^+$: 319.9778, found: 319.9777; e.e. (Method A): t_R = 13.3 min, 95.4%.

General method for the syntheses of ethyl 2-(*tert*-butoxycarbonyl)-7-(*tert*-butoxycarbonyloxy)-iodo-1,2,3,4-tetrahydroisoquinoline-3-carboxylates ((*S*)-22), ((*S*)-23), and ((*S*)-24).

To a solution of compound **(*S*)-13**, **(*S*)-17** or **(*S*)-20** (1.44 mmol) in anhydrous dichloromethane (12.5 mL) were successively added 4-dimethylaminopyridine (53 mg, 0.43 mmol, 0.3 eq.), anhydrous triethylamine (399 µL, 2.88 mmol, 2 eq.) and a solution of di-*tert*-butyl dicarbonate (629 mg, 2.88 mmol, 2 eq.) in anhydrous dichloromethane (0.5 mL). The reaction mixture was stirred at room temperature for 16-20 h before addition of a 0.1 M hydrochloric acid solution (25 mL). After decantation, the aqueous layer was extracted with dichloromethane (2 × 50 mL). The organic layers were combined, dried over anhydrous magnesium sulfate, filtered and evaporated under reduced pressure. The residue was purified by column chromatography (SiO_2, cyclohexane/ethyl acetate, 8/2, v/v) to give compound **(*S*)-22**, **(*S*)-23** or **(*S*)-24**.

Ethyl (3*S*)-2-(*tert*-butoxycarbonyl)-7-(*tert*-butoxycarbonyloxy)-5-iodo-1,2,3,4-tetrahydroisoquinoline-3-carboxylate ((*S*)-22)

Compound **(*S*)-22** (pale yellow oil, 98 mg, 0.18 mmol) was synthesized according to the general protocol described above, starting from compound **(*S*)-13** (100 mg, 0.29 mmol) with a reaction time of 16 h. Yield: 62%; R_f (SiO_2, cyclohexane/ethyl acetate, 8/2, v/v): 0.31; IR (ATR accessory) ν 2978, 1758, 1699, 1244, 1147 cm^{-1}; ^{1}H NMR (400.13 MHz, $CDCl_3$) δ 1.16 (t, 3H, ^3J = 7.1 Hz), 1.17 (t, 3H*, ^3J = 7.1 Hz), 1.45 (s, 9H), 1.50 (s, 9H), 1.54 (s, 18H), 2.96 (dd, 1H, ^2J = 16.2 Hz, ^3J = 6.2 Hz), 3.07 (dd, 1H*, ^2J = 16.1 Hz, ^3J = 6.3 Hz), 3.32 (dd, 1H, ^2J = 16.2 Hz, ^3J = 4.3 Hz), 3.42 (dd, 1H*, ^2J = 16.1

Hz, ^3J = 2.1 Hz), 4.09 (m, 4H), 4.44 (m, 2H), 4.69 (m, 2H), 4.79 (dd, 1H, ^3J = 4.3, 6.2 Hz), 5.14 (dd, 1H*, ^3J = 2.1, 6.3 Hz), 6.96 (s, 1H), 6.97 (s, 1H*), 7.54 (s, 2H); ^{13}C NMR (50.32 MHz, $CDCl_3$) δ 14.2 (2C), 27.6 (6C), 27.8 (6C), 35.8, 36.2*, 44.9*, 45.1, 53.8, 54.8*, 61.9, 62.1*, 84.3 (2C), 85.6, 85.7, 99.8*, 100.3, 119.3, 119.6*, 130.8 (2C), 132.1 (2C), 133.8, 134.2*, 146.8 (2C), 149.8 (2C), 149.8 (2C), 169.5, 169.6*; HRMS: *m/z* calculated for $C_{22}H_{30}INNaO_7^+$ [M+Na]$^+$: 570.0959, found: 570.0962.

*On the NMR spectra, two rotamers were evidenced in 50% vs 50% proportions. The symbol * refers to the minor rotamer.*

Ethyl (3*S*)-2-(*tert*-butoxycarbonyl)-7-(*tert*-butoxycarbonyloxy)-6-iodo-1,2,3,4-tetrahydroisoquinoline-3-carboxylate ((*S*)-23)

Compound **(*S*)-23** (pale yellow oil, 502 mg, 0.92 mmol) was synthesized according to the general protocol described above, starting from compound **(*S*)-17** (500 mg, 1.44 mmol) with a reaction time of 16 h. Yield: 64%; R_f (SiO_2, cyclohexane/ethyl acetate, 8/2, v/v): 0.31; IR (ATR accessory) ν 2979, 1760, 1705, 1236, 1140 cm^{-1}; ^{1}H NMR (400.13 MHz, $CDCl_3$) δ 1.16 (t, 3H, ^3J = 7.1 Hz), 1.17 (t, 3H*, ^3J = 7.1 Hz), 1.45 (s, 9H*), 1.51 (s, 9H), 1.57 (s, 18H), 3.09 (m, 3H), 3.23 (dd, 1H*, ^2J = 16.1 Hz, ^3J = 2.6 Hz), 4.07 (m, 4H), 4.42 (m, 2H), 4.68 (d, 1H, ^2J = 17.1 Hz), 4.69 (d, 1H*, ^2J = 16.6 Hz), 4.77 (m, 1H*), 5.13 (dd, 1H, ^3J = 2.6, 6.2 Hz), 6.93 (s, 1H), 6.97 (s, 1H*), 7.60 (s, 1H), 7.61 (s, 1H*); ^{13}C NMR (100.6 MHz, $CDCl_3$) δ 14.1, 14.2*, 27.8 (6C), 28.4* (3C), 28.5 (3C), 30.1, 30.6*, 43.6*, 44.1, 52.1, 54.0*, 61.4 (2C), 80.9 (2C), 84.3 (2C), 88.0, 88.3*, 120.3, 120.6*, 131.8, 132.2*, 134.7, 135.8*, 138.6*, 139.2, 149.9, 150.1*, 151.0, 151.1*, 154.7*, 155.3, 170.9, 171.4*; HRMS: *m/z* calculated for $C_{22}H_{30}INNaO_7^+$ [M+Na]$^+$: 570.0959, found: 570.1011.

*On the NMR spectra, two rotamers were evidenced in 52% vs 48% proportions. The symbol * refers to the minor rotamer.*

Ethyl (3*S*)-2-(*tert*-butoxycarbonyl)-7-(*tert*-butoxycarbonyloxy)-8-iodo-1,2,3,4-tetrahydroisoquinoline-3-carboxylate ((*S*)-24)

Compound **(*S*)-24** (pale yellow oil, 610 mg, 1.11 mmol) was synthesized according to the general protocol described above, starting from compound **(*S*)-20** (840 mg, 2.42 mmol) with a reaction time of 20 h. Yield: 46%; R_f (SiO_2, cyclohexane/ethyl acetate, 8/2, v/v): 0.30; IR (ATR accessory) ν 2979, 1760, 1698, 1143 cm^{-1}; ^{1}H NMR (400.13 MHz, $CDCl_3$) δ 1.16 (6H, t, ^3J = 7.1 Hz), 1.48 (s, 9H*), 1.53 (s, 9H), 1.57 (s, 18H), 3.14 (m, 3H), 3.25 (dd, 1H*, ^2J = 16.1 Hz, ^3J = 2.6 Hz), 4.08 (m, 4H), 4.32 (m, 2H), 4.77 (m, 2H), 4.85 (m, 1H), 5.13 (dd, 1H, ^3J = 2.6, 6.3 Hz), 7.00 (d, 2H, ^3J = 8.2 Hz), 7.15 (d, 2H, ^3J = 8.2 Hz); ^{13}C NMR (100.6 MHz, $CDCl_3$) δ 14.2, 14.3*, 27.8 (6C), 28.5 (6C), 30.8, 31.4*, 50.3*, 51.1, 52.0, 53.7*, 61.5 (2C), 81.1 (2C), 84.3 (2C), 94.9, 95.1*, 120.7, 120.8*, 129.3*, 129.9, 131.4*, 131.6, 137.0, 137.8*, 150.2, 150.3*, 151.2, 154.7*, 155.3 (2C), 170.9, 171.3*; HRMS: *m/z* calculated for $C_{22}H_{30}INNaO_7^+$ [M+Na]$^+$: 570.0959, found: 570.0983.

*On the NMR spectra, two rotamers were evidenced in 56% vs 44% proportions. The symbol * refers to the minor rotamer.*

General method for the syntheses of ethyl (3*S*)-2-(*tert*-butoxycarbonyl)-7-(*tert*-butoxycarbonyloxy)-trimethylstannyl-1,2,3,4-tetrahydroisoquinoline-3-carboxylates ((*S*)-25) and ((*S*)-26).

A solution of iodinated compound **(*S*)-22** or **(*S*)-23** (0.97 mmol) in anhydrous 1,4-dioxane (13 mL) was degazed for 20 min under argon. then were successively added, under argon and stirring, tetrakis(triphenylphosphine)palladium(0) (56 mg, 48.6 µmol, 5% mol) and hexamethylditin (400 µL, 1.93 mmol, 2 eq.). The reaction mixture was refluxed for 1.5 h. After cooling to room temperature,

the solution was filtered through a pad of celite® 545, washed with ethyl acetate (3 × 10 mL). The filtrate was evaporated under reduced pressure and the residue was purified by column chromatography (SiO_2, cyclohexane/ethyl acetate, 8/2, v/v) to give stannane **(*S*)-25** or **(*S*)-26**.

Ethyl (3*S*)-2-(*tert*-butoxycarbonyl)-7-(*tert*-butoxycarbonyloxy)-5-trimethylstannyl-1,2,3,4-tetrahydroisoquinoline-3-carboxylate ((*S*)-25)

Stannane **(*S*)-25** (yellow oil, 78 mg, 0.13 mmol) was synthesized according to the general protocol described above, starting from iodinated compound **(*S*)-22** (100 mg, 0.18 mmol). Yield: 72%; R_f (SiO_2, cyclohexane/ethyl acetate, 8/2, v/v): 0.50; IR (ATR accessory) ν 2979, 1756, 1701, 1241, 1151 cm^{-1}; ^1H NMR (200.13 MHz, $CDCl_3$) δ 0.33 (s, 18H, $^2J_{^{117/119}Sn\text{-}H}$ = 53.9 Hz), 1.14 (t, 6H, ^3J = 7.1 Hz), 1.45 (s, 9H*), 1.51 (s, 9H), 1.55 (s, 18H), 3.15 (m, 4H), 4.08 (m, 4H), 4.40 (d, 1H, ^2J = 16.6 Hz), 4.47 (d, 1H*, ^2J = 16.6 Hz), 4.73 (m, 3H), 5.11 (dd, 1H*, ^3J = 5.7, 2.9 Hz), 6.91 (m, 2H), 7.04 (m, 2H); ^{13}C NMR (50.32 MHz, $CDCl_3$) δ -8.5 (6C), 14.2 (2C), 27.9 (6C), 28.5 (6C), 33.5, 34.0*,44.5*, 45.0, 52.9, 54.8*, 61.4 (2C), 80.8 (2C), 83.7 (2C), 119.5, 119.7*, 126.6, 126.9*, 133.7, 134.8*, 135.4, 136.0*, 143.7, 144.5*, 149.4, 152.2*, 154.8 (2C), 155.5 (2C), 171.2, 171.7*; HRMS: *m*/*z* calculated for $C_{25}H_{39}NNaO_7{}^{120}Sn^+$ $[M+Na]^+$: 608.1641, found: 608.1638.

*On the NMR spectra, two rotamers were evidenced in 51% vs 49% proportions. The symbol * refers to the minor rotamer.*

Ethyl (3*S*)-2-(*tert*-butoxycarbonyl)-7-(*tert*-butoxycarbonyloxy)-6-trimethylstannyl-1,2,3,4-tetrahydroisoquinoline-3-carboxylate ((*S*)-26)

Stannane **(*S*)-26** (yellow oil, 1.80 g, 3.08 mmol) was synthesized according to the general protocol described above, starting from iodinated compound **(*S*)-23** (3.00 g, 5.48 mmol). Yield: 56%; R_f (SiO_2, cyclohexane/ethyl acetate, 8/2, v/v): 0.46; IR (ATR accessory) ν 2978, 1755, 1699, 1249, 1137 cm^{-1}; ^1H NMR (400.13 MHz, $CDCl_3$) δ 0.30 (s, 18H, $^2J_{^{117}Sn\text{-}H}$ = 54.1 Hz, $^2J_{^{119}Sn\text{-}H}$ = 56.6 Hz), 1.13 (t, 3H, ^3J = 7.1 Hz), 1.14 (t, 3H*, ^3J = 7.1 Hz), 1.44 (s, 9H*), 1.50 (s, 9H), 1.53 (s, 18H), 3.10 (m, 3H), 3.24 (dd, 1H*, ^2J = 15.8 Hz, ^3J = 2.9 Hz), 4.08 (m, 4H), 4.42 (d, 1H*, ^2J = 16.5 Hz), 4.48 (d, 1H, ^2J = 16.5 Hz), 4.73 (m, 3H), 5.10 (dd, 1H*, ^3J = 6.3, 2.9 Hz), 6.92 (s, 1H), 6.95 (s, 1H*), 7.17 (s, 1H), 7.18 (s, 1H*); ^{13}C NMR (100.6 MHz, $CDCl_3$) δ -9.0 (6C, $^1J_{^{119}Sn\text{-}C}$ = 364 Hz, $^1J_{^{117}Sn\text{-}C}$ = 348 Hz), 14.1, 14.2*, 27.9 (6C), 28.4* (3C), 28.5 (3C), 30.6, 31.2*, 44.1*, 44.6, 52.7, 54.7*, 61.3 (2C), 80.7 (2C), 83.3 (2C), 118.7, 119.0*, 129.3, 129.9*, 132.3, 132.6*, 134.8, 135.8*, 136.1*, 136.6, 152.5 (2C), 154.9 (2C), 155.0*, 155.5, 171.5, 172.1*; HRMS: *m*/*z* calculated for $C_{26}H_{41}NO_9{}^{120}Sn^+$ $[M+HCOOH]^+$: 631.1798, found: 631.2399.

*On the NMR spectra, two rotamers were evidenced in 53% vs 47% proportions. The symbol * refers to the minor rotamer.*

Ethyl (3*S*)-2-(*tert*-butoxycarbonyl)-7-(*tert*-butoxycarbonyloxy)-5-trimethylstannyl-1,2,3,4-tetrahydroisoquinoline-3-carboxylate ((*S*)-27)

A solution of 1.3 M isopropylmagnesium chloride lithium chloride complex solution in tetrahydrofuran (715 µL, 0.94 mmol, 1.2 eq.) was added dropwise, under argon and at -40 °C, to a solution of iodinated compound **(*S*)-24** (430 mg, 0.79 mmol) in anhydrous tetrahydrofuran (3 mL). After stirring at -40 °C for 20 min, a solution of trimethyltin chloride (200 µL, 0.94 mmol, 1.2 eq.) in anhydrous tetrahydrofuran (1.5 mL) was added. The reaction mixture was then stirred at -40 °C for 3 h. After heating to room temperature, the reaction mixture was evaporated under reduced pressure and the residue was purified by column chromatography (SiO_2, cyclohexane/ethyl acetate, 8/2, v/v) to yield stannane **(*S*)-27** (338 mg, 0.58 mmol) as a yellow oil. Yield: 73%; R_f (SiO_2, cyclohexane/ethyl acetate, 8/2, v/v): 0.60; IR (ATR accessory) ν 2978, 1754, 1699, 1142 cm^{-1}; ^1H NMR (200.13 MHz,

CDCl3) δ 0,27 (s, 9H, $^2J_{^{119}Sn\text{-}H}$ = 56.2 Hz, $^2J_{^{117}Sn\text{-}H}$= 54.0 Hz), 0,39 (s, 9H*, $^2J_{^{119}Sn\text{-}H}$ = 56.1 Hz, $^2J_{^{117}Sn\text{-}H}$ = 54.2 Hz), 1.15 (m, 6H), 1.44 (s, 9H), 1.52 (s, 9H), 1.54 (s, 18 H), 3.15 (m, 4H), 4.10 (m, 4H), 4.42-4.76 (m, 5H), 5.02 (dd, 1H*, ^3J = 3.8, 5.9 Hz), 6.95 (d, 1H, ^3J = 8.1 Hz), 6.96 (d, 1H*, ^3J = 8.1 Hz), 7.11 (d, 1H, ^3J = 8.1 Hz), 7.13 (d, 1H*, ^3J = 8.1 Hz); ^{13}C NMR (50.32 MHz, CDCl3) δ -6.4 (6C, $^1J_{^{117/119}Sn\text{-}C}$ = 353 Hz), 14.2 (2C), 27.9 (6C), 28.4 (3C), 28.6 (3C), 31.3, 32.0*, 46.5*, 47.7, 52.5, 54.6*, 61.3 (2C), 80.6*, 80.9, 83.5 (2C), 119.8, 120.0*, 129.1 (2C), 129.5*, 130.1*, 130,4, 131.5, 140.8, 142.5*, 152.4, 154.7*, 155.0 (2C), 155.5 (2C), 171.5, 172.2*; HRMS: *m/z* calculated for $C_{25}H_{39}NNaO_7{}^{120}Sn^+$ [M+Na]$^+$: 608.1641, found: 608.1636.

*On the NMR spectra, two rotamers were evidenced in 57% vs 43% proportions. The symbol * refers to the minor rotamer.*

General method for the syntheses of 2-*tert*-butyl 3-ethyl 7-hydroxy-iodo-3,4-dihydroisoquinoline-2,3(1*H*)-dicarboxylates ((*R*/*S*)-28, (*R*/*S*)-29 and (*R*/*S*)-30)

To a solution of compounds **(*R*/*S*)-13**, **(*R*/*S*)-17** or **(*R*/*S*)-20** (2.88 mmol) in a mixture of tetrahydrofuran/aqueous saturated sodium bicarbonate solution (8 mL, 1/1, v/v) was added under stirring di-*tert*-butyl dicarbonate (0.70 g, 3.20 mmol, 1.11 eq.). The mixture was stirred at room temperature for 2-6 h, before extraction with ethyl acetate (2 × 50 mL). The organic layers were combined, dried over anhydrous magnesium sulfate, filtered and evaporated under reduced pressure. The residue was purified by column chromatography (SiO_2, cyclohexane/ethyl acetate, 7/3, v/v) to give compounds **(*R*/*S*)-28**, **(*R*/*S*)-29** or **(*R*/*S*)-30**.

(3*S*)-2-*tert*-butyl 3-ethyl 7-hydroxy-5-iodo-3,4-dihydroisoquinoline-2,3(1*H*)-dicarboxylate ((*S*)-28)

Compound **(*S*)-28** (colorless oil, 574 mg, 1.28 mmol) was synthesized according to the general protocol described above, starting from amine **(*S*)-13** (478 mg, 1.38 mmol) with a reaction time of 6 h. Yield: 93%; R_f (SiO_2, cyclohexane/ethyl acetate, 7/3, v/v): 0.39; IR (ATR accessory) ν 3000-3500, 2925, 1739, 1664, 1366, 1160 cm^{-1}; ^{1}H NMR (500.13 MHz, CDCl3) δ 1.18 (t, 3H*, ^3J = 7.2 Hz), 1.21 (t, 3H, ^3J = 7.1 Hz), 1.47 (s, 9H), 1.52 (s, 9H*), 2.94 (dd, 1H*, ^2J = 16.0 Hz, ^3J = 6.1 Hz), 3.05 (dd, 1H, ^2J = 15.8 Hz, ^3J = 6.1 Hz), 3.29 (dd, 1H, ^2J = 15.8 Hz, ^3J = 4.3 Hz), 3.41 (dd, 1H*, ^2J = 16.0 Hz, ^3J = 2.4 Hz), 4.10 (m, 4H), 4.42 (d, 1H, ^2J = 16.0 Hz), 4.45 (d, 1H*, ^2J = 16.2 Hz), 4.55 (d, 1H*, ^2J = 12.5 Hz), 4.57 (d, 1H, ^2J = 15.9 Hz), 4.75 (dd, 1H, ^3J = 4.4, 5.9 Hz), 5.09 (d, 1H*, ^3J = 4.1 Hz), 6.61 (s, 1H*), 6.62 (s, 1H), 7.26 (s, 2H); ^{13}C NMR (125.77 MHz, CDCl3) δ 14.2*, 14.3, 28.4 (3C), 28.5* (3C), 35.8 (2C), 44.8, 45.2*, 53.6*, 55.1, 61.7, 61.8*, 81.3*, 81.7, 99.9, 100.7*, 113.6 (2C), 125.1 (2C), 126.6 (2C), 135.2*, 135.4, 155.2, 155.5*, 155.7*, 156.0, 171.8, 171.9*; HRMS: *m/z* calculated for $C_{17}H_{23}INO_5^+$ [M+H]$^+$: 448.0615, found: 448.0604.

*On the NMR spectra, two rotamers were evidenced in 63% vs 37% proportions. The symbol * refers to the minor rotamer.*

(3*R*)-2-*tert*-butyl 3-ethyl 7-hydroxy-5-iodo-3,4-dihydroisoquinoline-2,3(1*H*)-dicarboxylate ((*R*)-28)

Compound **(*R*)-28** (colorless oil, 552 mg, 1.23 mmol) was synthesized according to the general protocol described above, starting from amine **(*R*)-13** (492 mg, 1.42 mmol) with a reaction time of 5 h. Yield: 87%; R_f (SiO_2, cyclohexane/ethyl acetate, 7/3, v/v): 0.39; IR (ATR accessory) ν 3000-3500, 2927, 1699, 1366, 1159 cm^{-1}; ^{1}H NMR (500.13 MHz, $CDCl_3$) δ 1.17 (t, 3H*, ^3J = 7.1 Hz), 1.21 (t, 3H, ^3J = 7.1 Hz), 1.46 (s, 9H), 1.51 (s, 9H*), 2.93 (dd, 1H*, ^2J = 15.9 Hz, ^3J = 6.0 Hz), 3.05 (dd, 1H, ^2J = 15.8 Hz, ^3J = 6.1 Hz), 3.28 (dd, 1H, ^2J = 15.8 Hz, ^3J = 4.2 Hz), 3.40 (dd, 1H*, ^2J = 15.9 Hz, ^3J = 1.8 Hz), 4.09 (m, 4H), 4.42 (d, 1H, ^2J = 16.0 Hz), 4.45 (d, 1H*, ^2J = 16.2 Hz), 4.54 (d, 1H*, ^2J = 16.3 Hz), 4.56 (d, 1H, ^2J = 16.0 Hz), 4.75 (dd, 1H, ^3J = 4.6, 5.7 Hz), 5.07 (d, 1H*, ^3J = 3.6 Hz), 6.62 (s, 2H), 7.25 (s, 2H); ^{13}C NMR (125.77 MHz, $CDCl_3$) δ 14.2*, 14.3, 28.4 (3C), 28.5* (3C), 35.8 (2C), 44.8, 45.2*, 53.6*, 55.1, 61.7, 61.8*, 81.3*, 81.7, 99.9, 100.7*, 113.7 (2C), 125.0*, 125.1, 126.5 (2C), 135.1*, 135.4, 155.2, 155.5*, 155.8*, 156.1, 171.8 (2C); HRMS: *m*/*z* calculated for $C_{17}H_{23}INO_5^+$ $[M+H]^+$: 448.0615, found: 448.0608.

*On the NMR spectra, two rotamers were evidenced in 64% vs 36% proportions. The symbol * refers to the minor rotamer.*

(3*S*)-2-*tert*-butyl 3-ethyl 7-hydroxy-6-iodo-3,4-dihydroisoquinoline-2,3(1*H*)-dicarboxylate ((*S*)-29)

Compound **(*S*)-29** (colorless oil, 1.26 g, 2.82 mmol) was synthesized according to the general protocol described above, starting from amine **(*S*)-17** (1.00 g, 2.88 mmol) with a reaction time of 2 h. Yield: 98%; R_f (SiO_2, cyclohexane/ethyl acetate, 7/3, v/v): 0.34; IR (ATR accessory) ν 3000-3400, 2971, 1748, 1667, 1414, 1186 cm^{-1}; ^{1}H NMR (500.13 MHz, $CDCl_3$) δ 1.14 (t, 3H*, ^3J = 7.0 Hz), 1.16 (t, 3H, ^3J = 7.0 Hz), 1.45 (s, 9H), 1.51 (s, 9H*), 3.03 (m, 3H), 3.14 (dd, 1H*, ^2J = 15.7 Hz, ^3J = 2.7 Hz), 4.07 (m, 4H), 4.39 (d, 1H*, ^2J = 16.1 Hz), 4.42 (d, 1H, ^2J = 15.9 Hz), 4.58 (d, 1H*, ^2J = 16.8 Hz), 4.59 (d, 1H, ^2J = 16.4 Hz), 4.74 (t, 1H, ^3J = 5.1 Hz), 5.08 (dd, 1H*, ^3J = 2.7, 6.0 Hz), 6.73 (s, 1H*), 6.75 (s, 1H), 7.43 (s, 1H*), 7.45 (s, 1H); ^{13}C NMR (125.77 MHz, $CDCl_3$) δ 14.2*, 14.3, 28.4 (3C), 28.5* (3C), 30.2*, 30.5, 44.1, 44.5*, 52.7*, 54.4, 61.5 (2C), 81.0, 81.3*, 82.9, 83.3*, 112.5*, 112.7, 126.0 (2C), 135.2, 135.7*, 137.7*, 138.1, 154.0, 154.4*, 155.2*, 155.6, 171.6*, 171.9; HRMS: *m*/*z* calculated for $C_{17}H_{23}INO_5^+$ $[M+H]^+$: 448.0615, found: 448.0620.

*On the NMR spectra, two rotamers were evidenced in 56% vs 44% proportions. The symbol * refers to the minor rotamer.*

(3*R*)-2-*tert*-butyl 3-ethyl 7-hydroxy-6-iodo-3,4-dihydroisoquinoline-2,3(1*H*)-dicarboxylate ((*R*)-29)

Compound **(*R*)-29** (colorless oil, 637 mg, 1.42 mmol) was synthesized according to the general protocol described above, starting from amine **(*R*)-17** (0.50 g, 1.44 mmol) with a reaction time of 2 h. Yield: 99%; R_f (SiO_2, cyclohexane/ethyl acetate, 7/3, v/v): 0.34; IR (ATR accessory) ν 3100-3400, 2973, 1743, 1663, 1413, 1188 cm^{-1}; ^{1}H NMR (500.13 MHz, $CDCl_3$) δ 1.15 (t, 3H*, ^3J = 7.2 Hz), 1.17 (t, 3H, ^3J = 7.1 Hz), 1.46 (s, 9H), 1.51 (s, 9H*), 3.04 (m, 3H), 3.15 (dd, 1H*, ^2J = 15.5 Hz, ^3J = 2.7 Hz), 4.09 (m, 4H), 4.39 (d, 1H*, ^2J = 16.5 Hz), 4.43 (d, 1H, ^2J = 15.9 Hz), 4.59 (d, 1H*, ^2J = 16.8 Hz), 4.60 (d, 1H, ^2J = 16.4 Hz), 4.75 (t, 1H, ^3J = 5.2 Hz), 5.09 (dd, 1H*, ^3J = 2.7, 6.0 Hz), 6.74 (s, 1H*), 6.77 (s, 1H), 7.43 (s, 1H*), 7.45 (s, 1H); ^{13}C NMR (125.77 MHz, $CDCl_3$) δ 14.2*, 14.3, 28.5 (3C), 28.5* (3C), 30.2*, 30.5, 44.1, 44.5*, 52.6*, 54.4, 61.4, 61.5*, 81.0*, 81.2, 83.1, 83.5*, 112.5*, 112.7,

126.1 (2C), 135.3*, 135.9, 137.6, 138.0*, 153.8*, 154.2, 155.1, 155.5*, 171.5*, 171.8; HRMS: *m*/*z* calculated for $C_{18}H_{24}INO_7^+$ [M+H]$^+$: 448.0615, found: 448.0604.

*On the NMR spectra, two rotamers were evidenced in 55% vs 45% proportions. The symbol * refers to the minor rotamer.*

(3*S*)-2-*tert*-butyl 3-ethyl 7-hydroxy-8-iodo-3,4-dihydroisoquinoline-2,3(1*H*)-dicarboxylate ((*S*)-30)

Compound **(*S*)-30** (colorless oil, 631 mg, 1.41 mmol) was synthesized according to the general protocol described above, starting from amine **(*S*)-20** (500 mg, 1.44 mmol) with a reaction time of 4 h. Yield: 98%; R_f (SiO_2, cyclohexane/ethyl acetate, 7/3, v/v): 0.35; IR (ATR accessory) ν 3000-3500, 2926, 1737, 1665, 1366, 1159 cm^{-1}; ^{1}H NMR (500.13 MHz, $CDCl_3$) δ 1.15 (t, 6H, ^3J = 7.1 Hz), 1.49 (s, 9H*), 1.55 (s, 9H), 3.10 (m, 3H), 3.18 (dd, 1H*, ^2J = 15.7 Hz, ^3J = 2.5 Hz), 4.07 (m, 4H), 4.31 (d, 2H, ^2J = 17.2 Hz), 4.62 (d, 1H, ^2J = 17.2 Hz), 4.69 (d, 1H*, ^2J = 17.2 Hz), 4.87 (m, 1H*), 5.11 (dd, 1H, ^3J = 2.8, 6.1 Hz), 6.84 (d, 2H, ^3J = 8.2 Hz), 7.01 (d, 2H, ^3J = 8.2 Hz); ^{13}C NMR (125.77 MHz, $CDCl_3$) δ 14.2, 14.3*, 28.5 (6C), 30.6, 31.1*, 50.4*, 51.1, 52.3, 53.9*, 61.4 (2C), 81.1 (2C), 89.5, 89.8*, 113.3 (2C), 125.6*, 125.8, 129.8*, 130.2, 135.5, 136.1*, 153.8, 153.9*, 155.0*, 155.5, 171.2, 171.5*; HRMS: *m*/*z* calculated for $C_{17}H_{23}INO_5^+$ [M+H]$^+$: 448.0615, found: 448.0605.

*On the NMR spectra, two rotamers were evidenced in 51% vs 49% proportions. The symbol * refers to the minor rotamer.*

(3*R*)-2-*tert*-butyl 3-ethyl 7-hydroxy-8-iodo-3,4-dihydroisoquinoline-2,3(1*H*)-dicarboxylate ((*R*)-30)

Compound **(*R*)-30** (colorless oil, 613 mg, 1.37 mmol) was synthesized according to the general protocol described above, starting from amine **(*R*)-20** (500 mg, 1.44 mmol) with a reaction time of 4 h. Yield: 95%; R_f (SiO_2, cyclohexane/ethyl acetate, 7/3, v/v): 0.35; IR (ATR accessory) ν 3000-3500, 2978, 1736, 1663, 1366, 1159 cm^{-1}; ^{1}H NMR (500.13 MHz, $CDCl_3$) δ 1.15 (t, 6H, ^3J = 7.1 Hz), 1.49 (s, 9H*), 1.55 (s, 9H), 3.10 (m, 3H), 3.17 (dd, 1H*, ^2J = 15.7 Hz, ^3J = 2.2 Hz), 4.07 (m, 4H), 4.31 (d, 2H, ^2J = 17.0 Hz), 4.62 (d, 1H, ^2J = 17.2 Hz), 4.69 (d, 1H*, ^2J = 17.2 Hz), 4.87 (m, 1H*), 5.11 (m, 1H), 6.84 (d, 2H, ^3J = 8.2 Hz), 7.01 (d, 2H, ^3J = 8.2 Hz); ^{13}C NMR (125.77 MHz, $CDCl_3$) δ 14.2, 14.3*, 28.5 (6C), 30.6, 31.1*, 50.4*, 51.2, 52.4, 53.9*, 61.4 (2C), 81.1 (2C), 89.5, 89.8*, 113.3 (2C), 125.6*, 125.8, 129.8*, 130.2, 135.5, 136.1*, 153.8, 153.9*, 155.0*, 155.5, 171.2, 171.5*; HRMS: *m*/*z* calculated for $C_{17}H_{22}INNaO_5^+$ [M+Na]$^+$: 470.0435, found: 470.0425.

*On the NMR spectra, two rotamers were evidenced in 51% vs 49% proportions. The symbol * refers to the minor rotamer.*

General method for the syntheses of 2-*tert*-butyl 3-ethyl 7-(ethoxymethoxy)-iodo-3,4-dihydroisoquinoline-2,3(1*H*)-dicarboxylates ((*R*/*S*)-31, (*R*/*S*)-32 and (*R*/*S*)-33).

To a solution of **(*R*/*S*)-28**, **(*R*/*S*)-29** or **(*R*/*S*)-30** (1.12 mmol) in anhydrous tetrahydrofuran (4 mL) were successively added anhydrous *N*,*N*-diisopropylethylamine (430 µL, 2.53 mmol, 2.3 eq.) and chloromethyl ethyl ether (218 µL, 2.35 mmol, 2.1 eq.). The mixture was stirred in a sealed tube under argon at 40 °C for 16-22 h. After cooling to room temperature, the mixture was diluted with methyl *tert*-butyl ether (5 mL) and water (4 mL). The organic layer was washed successively with an aqueous 5% acetic acid solution (2 × 25 mL) and an aqueous 10% sodium carbonate solution (2 ×

5 mL), dried over anhydrous magnesium sulfate, filtered and evaporated under reduced pressure. The residue was purified by column chromatography (SiO_2, cyclohexane/ethyl acetate, 8/2, v/v) to give compounds **(*R*/*S*)-31**, **(*R*/*S*)-32** or **(*R*/*S*)-33.**

(3*S*)-2-*tert*-butyl 3-ethyl 7-(ethoxymethoxy)-5-iodo-3,4-dihydroisoquinoline-2,3(1*H*)-dicarboxylate ((*S*)-31)

Compound **(*S*)-31** (colorless oil, 535 mg, 1.06 mmol) was synthesized according to the general protocol described above, starting from compound **(*S*)-28** (640 mg, 1.43 mmol) with a reaction time of 22 h. Yield: 74%; R_f (SiO_2, cyclohexane/ethyl acetate, 8/2, v/v): 0.44; IR (ATR accessory) ν 2975, 1739, 1697, 1391, 1365, 1161, 1005 cm^{-1}; ^1H NMR (500.13 MHz, $CDCl_3$) δ 1.20 (m, 12H), 1.45 (s, 9H*), 1.51 (s, 9H), 2.94 (dd, 1H*, ^2J = 16.2 Hz, ^3J = 6.2 Hz), 3.05 (dd, 1H, ^2J = 15.9 Hz, ^3J = 6.2 Hz), 3.28 (dd, 1H*, ^2J = 15.9 Hz, ^3J = 4.1 Hz), 3.39 (dd, 1H, ^2J = 16.2 Hz, ^3J = 2.3 Hz), 3.70 (m, 4H), 4.09 (m, 4H); 4.42 (d, 1H, ^2J = 16.5 Hz), 4.46 (d, 1H*, ^2J = 17.1 Hz), 4.62 (d, 1H, ^2J = 16.1 Hz), 4.65 (d, 1H*, ^2J = 16.0 Hz), 4.76 (dd, 1H, ^3J = 4.4, 5.9 Hz), 5.11 (dd, 1H*, ^3J = 2.4, 6.2 Hz), 5.16 (m, 4H), 6.80 (s, 1H), 6.84 (s, 1H*), 7.41 (s, 2H); ^{13}C NMR (125.77 MHz, $CDCl_3$) δ 14.3 (2C), 15.2 (2C), 28.4* (3C), 28.5 (3C), 35.9, 36.0*, 44.5*, 45.1, 53.2, 54.8*, 61.4 (2C), 64.6 (2C), 80.9 (2C), 93.3*, 93.4, 99.9*, 101.0, 113.9, 114.6*, 125.6*, 125.8, 128.4, 128.8*, 135.3, 136.0*, 154.7, 155.3*, 156.4, 156.5*, 171.1, 171.5*; HRMS: *m*/*z* calculated for $C_{20}H_{29}INO_6^+$ $[M+H]^+$: 506.1034, found: 506.1022.

*On the NMR spectra, two rotamers were evidenced in 53% vs 47% proportions. The symbol * refers to the minor rotamer.*

(3*R*)-2-*tert*-butyl 3-ethyl 7-(ethoxymethoxy)-5-iodo-3,4-dihydroisoquinoline-2,3(1*H*)-dicarboxylate ((*R*)-31)

Compound **(*R*)-31** (colorless oil, 539 mg, 1.07 mmol) was synthesized according to the general protocol described above, starting from compound **(*R*)-28** (518 mg, 1.16 mmol) with a reaction time of 22 h. Yield: 92%; R_f (SiO_2, cyclohexane/ethyl acetate, 8/2, v/v): 0.44; IR (ATR accessory) ν 2976, 2931, 1698, 1391, 1366, 1159, 1005 cm^{-1}; ^1H NMR (500.13 MHz, $CDCl_3$) δ 1.20 (m, 12H), 1.46 (s, 9H*), 1.52 (s, 9H), 2.94 (dd, 1H*, ^2J = 16.1 Hz, ^3J = 6.3 Hz), 3.05 (dd, 1H, ^2J = 15.9 Hz, ^3J = 6.1 Hz), 3.29 (dd, 1H*, ^2J = 15.9 Hz, ^3J = 4.1 Hz), 3.39 (dd, 1H, ^2J = 16.1 Hz, ^3J = 1.9 Hz), 3.70 (m, 4H), 4.09 (m, 4H); 4.42 (d, 1H, ^2J = 16.6 Hz), 4.46 (d, 1H*, ^2J = 17.6 Hz), 4.62 (d, 1H, ^2J = 16.6 Hz), 4.65 (d, 1H*, ^2J = 16.2 Hz), 4.77 (m, 1H), 5.11 (dd, 1H*, ^3J = 2.2, 6.0 Hz), 5.16 (m, 4H), 6.80 (s, 1H*), 6.84 (s, 1H), 7.41 (s, 2H); ^{13}C NMR (125.77 MHz, $CDCl_3$) δ 14.3 (2C), 15.2 (2C), 28.5* (3C), 28.6 (3C), 35.9, 36.1*, 44.5*, 45.1, 53.2, 54.8*, 61.4 (2C), 64.6 (2C), 80.9 (2C), 93.4 (2C), 99.9, 101.0*, 114.0, 114.6*, 125.6*, 125.8, 128.4*, 128.8, 135.3, 136.1*, 154.7, 155.3*, 156.4, 156.5*, 171.1, 171.5*; HRMS: *m*/*z* calculated for $C_{20}H_{29}INO_6^+$ $[M+H]^+$: 506.1034, found: 506.1025.

*On the NMR spectra, two rotamers were evidenced in 52% vs 48% proportions. The symbol * refers to the minor rotamer.*

(3*S*)-2-*tert*-butyl 3-ethyl 7-(ethoxymethoxy)-6-iodo-3,4-dihydroisoquinoline-2,3(1*H*)-dicarboxylate ((*S*)-32)

Compound **(*S*)-32** (colorless oil, 487 mg, 0.96 mmol) was synthesized according to the general protocol described above, starting from compound **(*S*)-29** (0.50 g, 1.12 mmol) with a reaction time of 16 h. Yield: 86%; R_f (SiO_2, cyclohexane/ethyl acetate, 8/2, v/v): 0.34; IR (ATR accessory) ν 2973,

2930, 1738, 1698, 1390, 1366, 1165, 1150 cm^{-1}; ^{1}H NMR (500.13 MHz, $CDCl_3$) δ 1.15 (t, 3H, ^{3}J = 6.9 Hz), 1.16 (t, 3H*, ^{3}J = 7.0 Hz), 1.22 (t, 3H, ^{3}J = 6.9 Hz), 1.24 (t, 3H*, ^{3}J = 7.0 Hz), 1.45 (s, 9H*), 1.52 (s, 9H), 3.04 (m, 3H), 3.16 (dd, 1H*, ^{2}J = 15.8 Hz, ^{3}J = 2.2 Hz), 3.76 (m, 4H, ^{3}J = 6.9 Hz), 4.08 (m, 4H), 4.41 (d, 1H*, ^{2}J = 16.4 Hz), 4.45 (d, 1H, ^{2}J = 16.9 Hz), 4.63 (d, 1H, ^{2}J = 16.9 Hz), 4.65 (d, 1H*, ^{2}J = 16.4 Hz), 4.75 (t, 1H, ^{3}J = 5.2 Hz), 5.11 (dd, 1H*, ^{3}J = 2.4, 6.0 Hz), 5.25 (m, 4H), 6.82 (s, 1H*), 6.89 (s, 1H), 7.54 (s, 1H), 7.55 (s, 1H*); ^{13}C NMR (125.8 MHz, $CDCl_3$) δ 14.2, 14.3*, 15.2 (2C), 28.4* (3C), 28.6 (3C), 30.1, 30.5*, 44.1*, 44.7, 52.4, 54.3*, 61.3*, 61.4, 64.9 (2C), 80.8*, 80.9, 84.9, 85.1*, 93.9, 94.1*, 112.4, 113.0*, 127.3, 127.7*, 134.5, 135.6*, 138.5*, 139.1, 154.9*, 155.0, 155.2*, 155.5, 171.3, 171.8*; HRMS: *m/z* calculated for $C_{20}H_{29}INO_6^+$ $[M+H]^+$: 506.1034, found: 506.1044.

*On the NMR spectra, two rotamers were evidenced in 55% vs 45% proportions. The symbol * refers to the minor rotamer.*

(3*R*)-2-*tert*-butyl 3-ethyl 7-(ethoxymethoxy)-6-iodo-3,4-dihydroisoquinoline-2,3(1*H*)-dicarboxylate ((*R*)-32)

Compound **(*R*)-32** (colorless oil, 651 mg, 1.29 mmol) was synthesized according to the general protocol described above, starting from compound **(*R*)-29** (637 mg, 1.42 mmol) with a reaction time of 16 h; Yield: 91%; R_f (SiO_2, cyclohexane/ethyl acetate, 8/2, v/v): 0.34; IR (ATR accessory) ν 2976, 2915, 1737, 1695, 1389, 1365, 1148 cm^{-1}; ^{1}H NMR (500.13 MHz, $CDCl_3$) δ 1.15 (t, 3H, ^{3}J = 7.1 Hz), 1.16 (t, 3H*, ^{3}J = 7.0 Hz), 1.22 (t, 3H, ^{3}J = 7.1 Hz), 1.24 (t, 3H*, ^{3}J = 7.0 Hz), 1.45 (s, 9H*), 1.52 (s, 9H), 3.04 (m, 3H), 3.16 (dd, 1H*, ^{2}J = 15.8 Hz, ^{3}J = 2.5 Hz), 3.76 (m, 4H, ^{3}J = 6.9 Hz), 4.08 (m, 4H), 4.41 (d, 1H*, ^{2}J = 16.5 Hz), 4.45 (d, 1H, ^{2}J = 16.9 Hz), 4.63 (d, 1H, ^{2}J = 16.9 Hz), 4.65 (d, 1H*, ^{2}J = 16.5 Hz), 4.75 (t, 1H, ^{3}J = 5.2 Hz), 5.11 (dd, 1H*, ^{3}J = 2.5, 6.1 Hz), 5.25 (m, 4H), 6.82 (s, 1H*), 6.89 (s, 1H), 7.54 (s, 1H), 7.55 (s, 1H*); ^{13}C NMR (125.8 MHz, $CDCl_3$) δ 14.2, 14.3*, 15.2 (2C), 28.5* (3C), 28.6 (3C), 30.1, 30.5*, 44.1*, 44.7, 52.4, 54.3*, 61.4 (2C), 64.9 (2C), 80.8*, 80.9, 84.9, 85.1*, 93.9, 94.1*, 112.4, 113.0*, 127.3, 127.7*, 134.5, 135.6*, 138.5*, 139.2*, 154.9*, 155.1, 155.2*, 155.6, 171.3, 171.8*; HRMS: m/z calculated for $C_{20}H_{29}INO_6^+$ $[M+H]^+$: 506.1034, found: 506.1020.
*On the NMR spectra, two rotamers were evidenced in 55% vs 45% proportions. The symbol * refers to the minor rotamer.*

(3*S*)-2-*tert*-butyl 3-ethyl 7-(ethoxymethoxy)-8-iodo-3,4-dihydroisoquinoline-2,3(1*H*)-dicarboxylate ((*S*)-33)

Compound **(*S*)-33** (colorless oil, 626 mg, 1.24 mmol) was synthesized according to the general protocol described above, starting from compound **(*S*)-30** (623 mg, 1.39 mmol) with a reaction time of 16 h. Yield: 89%; R_f (SiO_2, cyclohexane/ethyl acetate, 8/2, v/v): 0.35; IR (ATR accessory) ν 2975, 2905, 1737, 1697, 1390, 1366, 1162, 1014 cm^{-1}; ^{1}H NMR (500.13 MHz, $CDCl_3$) δ 1.15 (t, 6H, ^{3}J = 7.1 Hz), 1.22 (t, 6H, ^{3}J = 7.0 Hz), 1.48 (s, 9H*), 1.54 (s, 9H), 3.11 (m, 3H), 3.18 (dd, 1H*, ^{2}J = 15.8 Hz, ^{3}J = 2.7 Hz), 3.76 (q, 4H, ^{3}J = 7.1 Hz), 4.09 (m, 4H), 4.34 (d, 2H, ^{2}J = 17.4 Hz), 4.70 (d, 1H, ^{2}J = 17.4 Hz), 4.77 (d, 1H*, ^{2}J = 17.3 Hz), 4.86 (t, 1H*, ^{3}J = 4.8 Hz), 5.10 (dd, 1H, ^{3}J = 2.9, 6.0 Hz), 5.27 (m, 4H), 6.92 (d, 2H, ^{3}J = 8.4 Hz), 7.05 (d, 1H*, ^{3}J = 8.2 Hz) 7.06 (d, 1H, ^{3}J = 8.3 Hz); ^{13}C NMR (125.77 MHz, $CDCl_3$) δ 14.2, 14.3*, 15.2 (2C), 28.5 (6C), 30.7, 31.2*, 50.7*, 51.5, 52.4, 54.0*, 61.3 (2C), 64.9 (2C), 80.9 (2C), 91.3, 91.5*, 94.0 (2C), 113.3 (2C), 127.1*, 127.3, 129.1*, 129.6, 136.7, 137.4*, 154.9*, 155.1, 155.2*, 155.5, 171.2, 171.5*; HRMS: *m/z* calculated for $C_{20}H_{29}INO_6^+$ $[M+H]^+$: 506.1034, found: 506.1021.

*On the NMR spectra, two rotamers were evidenced in 57% vs 43% proportions. The symbol * refers to the minor rotamer.*

(3*R*)-2-*tert*-butyl 3-ethyl 7-(ethoxymethoxy)-8-iodo-3,4-dihydroisoquinoline-2,3(1*H*)-dicarboxylate ((*R*)-33)

Compound **(*R*)-33** (colorless oil, 571 mg, 1.13 mmol) was synthesized according to the general protocol described above, starting from compound **(*R*)-30** (582 mg, 1.30 mmol) with a reaction time of 16 h. Yield: 87%; R_f (SiO_2, cyclohexane/ethyl acetate, 8/2, v/v): 0.35; IR (ATR accessory) ν 2975, 1736, 1695, 1390, 1366, 1161, 1014 cm^{-1}; ^1H NMR (500.13 MHz, $CDCl_3$) δ 1.15 (t, 6H, ^3J = 7.1 Hz), 1.22 (t, 6H, ^3J = 7.1 Hz), 1.48 (s, 9H*), 1.54 (s, 9H), 3.11 (m, 3H), 3.18 (dd, 1H*, ^2J = 15.7 Hz, ^3J = 2.7 Hz), 3.76 (q, 4H, ^3J = 7.1 Hz), 4.08 (m, 4H), 4.34 (d, 2H, ^2J = 17.4 Hz), 4.70 (d, 1H, ^2J = 17.4 Hz), 4.77 (d, 1H*, ^2J = 17.3 Hz), 4.86 (t, 1H*, ^3J = 4.8 Hz), 5.10 (dd, 1H, ^3J = 2.9, 6.1 Hz), 5.26 (m, 4H), 6.92 (d, 2H, ^3J = 8.4 Hz), 7.05 (d, 1H*, ^3J = 8.3 Hz), 7.06 (d, 1H, ^3J = 8.4 Hz); ^{13}C NMR (125.77 MHz, $CDCl_3$) δ 14.2, 14.3*, 15.2 (2C), 28.5 (6C), 30.7, 31.2*, 50.7*, 51.5, 52.4, 54.0*, 61.3 (2C), 64.8 (2C), 80.9 (2C), 91.3, 91.5*, 94.0 (2C), 113.3 (2C), 127.1*, 127.3, 129.1*, 129.6, 136.7, 137.4*, 154.9*, 155.1, 155.2*, 155.5, 171.2, 171.5*; HRMS: *m/z* calculated for $C_{20}H_{29}INO_6^+$ $[M+H]^+$: 506.1034, found: 506.1032.

*On the NMR spectra, two rotamers were evidenced in 56% vs 44% proportions. The symbol * refers to the minor rotamer.*

General method for the syntheses of 2-*tert*-butyl 3-ethyl 7-(ethoxymethoxy)-(trimethylstannyl)-3,4-dihydroisoquinoline-2,3(1*H*)-dicarboxylates (*R*/*S*)-34 and (*R*/*S*)-35.

To a solution of iodinated compounds **(*R*/*S*)-31** or **(*R*/*S*)-32** (0.35 mmol) in degassed anhydrous 1,4-dioxane (6 mL) were successively added under stirring and argon flow, tetrakis(triphenylphosphine)palladium (0) (20 mg, 17.3 µmol, 5% mol) and hexamethylditin (145 µL, 0.70 mmol, 2 eq.). The mixture was refluxed for 0.5-3 h. After cooling to room temperature, the mixture was filtered on celite 545®, washed with ethyl acetate (3 × 10 mL) and the filtrate was evaporated under reduced pressure. The residue was purified by column chromatography (SiO_2, cyclohexane/ethyl acetate, 9/1, v/v) to give compounds **(*R*/*S*)-34** or **(*R*/*S*)-35.**

(3*S*)-2-*tert*-butyl 3-ethyl 7-(ethoxymethoxy)-5-(trimethylstannyl)-3,4-dihydroisoquinoline-2,3(1*H*)-dicarboxylate ((*S*)-34)

Compound **(*S*)-34** (yellowish oil, 611 mg, 1.21 mmol) was synthesized according to the general protocol described above, starting from iodinated compound **(*S*)-31** (623 mg, 1.39 mmol) with a reaction time of 2 h. Yield: 87%; R_f (SiO_2, cyclohexane/ethyl acetate, 9/1, v/v, iodine revelation): 0.18; IR (ATR accessory) ν 2976, 2905, 1740, 1697, 1390, 1366, 1164, 1012 cm^{-1}; ^1H NMR (500.13 MHz, $CDCl_3$) δ 0.33 (s, 18H, $^2J_{H\text{-}^{119}Sn}$ = 54.7 Hz, $^2J_{H\text{-}^{117}Sn}$ = 52.4 Hz), 1.15 (t, 6H, ^3J = 7.1 Hz), 1.22 (t, 3H*, ^3J = 6.9 Hz), 1.24 (t, 3H, ^3J = 6.7 Hz), 1.46 (s, 9H*), 1.52 (s, 9H), 3.12 (m, 3H), 3.19 (dd, 1H*, ^2J = 15.4 Hz, ^3J = 2.9 Hz), 3.72 (m, 4H), 4.06 (m, 4H), 4.47 (d, 1H*, ^2J = 16.4 Hz), 4.51 (d, 1H, ^2J = 17.0 Hz), 4.65 (d, 2H, ^2J = 16.4 Hz), 4.77 (t, 1H*, ^3J = 5.1 Hz), 5.13 (dd, 1H, ^3J = 5.9, 2.9 Hz), 5.19 (m, 4H), 6.77 (d, 1H, ^4J = 2.1 Hz), 6.83 (d, 1H*, ^4J = 2.1 Hz), 6.96 (d, 1H, ^4J = 2.4 Hz, $^3J_{H\text{-}^{119}Sn}$ = 52.5 Hz, $^3J_{H\text{-}^{117}Sn}$ = 49.7 Hz), 6.98 (d, 1H*, ^4J = 2.4 Hz, $^3J_{H\text{-}^{119}Sn}$ = 52.3 Hz, $^3J_{H\text{-}^{117}Sn}$ = 49.0 Hz); ^{13}C NMR (125.77 MHz, $CDCl_3$) δ -8.6 (3C, $^1J_{C\text{-}^{119}Sn}$ = 351 Hz, $^1J_{C\text{-}^{117}Sn}$ = 336 Hz), -8.5 (3C*, $^1J_{C\text{-}^{119}Sn}$ = 351 Hz, $^1J_{C\text{-}^{117}Sn}$ = 338 Hz), 14.2, 14.3*, 15.2 (2C), 28.5* (3C), 28.6 (3C), 33.3, 33.7*, 44.8*, 45.3, 53.1, 55.0*, 61.2*, 61.3, 64.3 (2C), 80.6 (2C), 93.4, 93.5*, 113.4, 114.2*, 123.0*, 123.1, 131.2,

131.7*, 133.7, 134.7*, 143.2*, 144.0, 154.9*, 155.6, 155.7, 155.9*, 171.4, 171.9*; HRMS: *m*/*z* calculated for $C_{23}H_{38}NO_6Sn^+$ [M+H]$^+$: 544.1716, found: 544.1708.

*On the NMR spectra, two rotamers were evidenced in 53% vs 47% proportions. The symbol * refers to the minor rotamer.*

(3*R*)-2-*tert*-butyl 3-ethyl 7-(ethoxymethoxy)-5-(trimethylstannyl)-3,4-dihydroisoquinoline-2,3(1*H*)-dicarboxylate ((*R*)-34)

Compound **(*R*)-34** (yellowish oil, 477 mg, 0.88 mmol) was synthesized according to the general protocol described above, starting from iodinated compound **(*R*)-31** (556 mg, 1.10 mmol) with a reaction time of 3 h. Yield: 80%; R_f (SiO_2, cyclohexane/ethyl acetate, 9/1, v/v, iodine revelation): 0.18; IR (ATR accessory) ν 2976, 2906, 1738, 1698, 1391, 1366, 1163, 1010 cm^{-1}; ^{1}H NMR (500.13 MHz, $CDCl_3$) δ 0.33 (s, 18H, $^2J_{H\text{-}^{119}Sn}$ = 54.7 Hz, $^2J_{H\text{-}^{117}Sn}$ = 52.4 Hz), 1.15 (t, 6H, ^3J = 7.1 Hz), 1.22 (t, 3H*, ^3J = 6.9 Hz), 1.24 (t, 3H, ^3J = 7.0 Hz), 1.46 (s, 9H*), 1.52 (s, 9H), 3.12 (m, 3H), 3.19 (dd, 1H*, ^2J = 15.4 Hz, ^3J = 2.9 Hz), 3.72 (m, 4H), 4.06 (m, 4H), 4.47 (d, 1H*, ^2J = 16.4 Hz), 4.61 (d, 1H, ^2J = 17.0 Hz), 4.65 (d, 2H, ^2J = 16.4 Hz), 4.77 (t, 1H*, ^3J = 5.1 Hz), 5.13 (dd, 1H, ^3J = 2.9, 5.9 Hz), 5.19 (m, 4H), 6.77 (d, 1H, ^4J = 2.1 Hz), 6.83 (d, 1H*, ^4J = 2.1 Hz), 6.96 (d, 1H, ^4J = 2.4 Hz, $^3J_{H\text{-}^{119}Sn}$ = 53.1 Hz, $^3J_{H\text{-}^{117}Sn}$ = 49.9 Hz), 6.98 (d, 1H*, ^4J = 2.4 Hz, $^3J_{H\text{-}^{119}Sn}$ = 52.5 Hz, $^3J_{H\text{-}^{117}Sn}$ = 49.4 Hz); ^{13}C NMR (125.77 MHz, $CDCl_3$) δ -8.6 (3C, $^1J_{C\text{-}^{119}Sn}$ = 351 Hz, $^1J_{C\text{-}^{117}Sn}$ = 336 Hz), -8.5 (3C*, $^1J_{C\text{-}^{119}Sn}$ = 351 Hz, $^1J_{C\text{-}^{117}Sn}$ = 339 Hz), 14.2, 14.3*, 15.2 (2C), 28.5* (3C), 28.6 (3C), 33.3, 33.8*, 44.8*, 45.3, 53.1, 55.0*, 61.2*, 61.3, 64.3 (2C), 80.6*, 80.7, 93.5, 93.6*, 113.5, 114.3*, 123.0*, 123.1, 131.2, 131.8*, 133.7, 134.8*, 143.2*, 144.1, 154.9*, 155.6, 155.8, 155.9*, 171.4, 171.9*; HRMS: *m*/*z* calculated for $C_{23}H_{38}NO_6Sn^+$ [M+H]$^+$: 544.1716, found: 544.1714.

*On the NMR spectra, two rotamers were evidenced in 54% vs 46% proportions. The symbol * refers to the minor rotamer.*

(3*S*)-2-*tert*-butyl 3-ethyl 7-(ethoxymethoxy)-6-(trimethylstannyl)-3,4-dihydroisoquinoline-2,3(1*H*)-dicarboxylate ((*S*)-35)

Compound **(*S*)-35** (yellowish oil, 169 mg, 0.31 mmol) was synthesized according to the general protocol described above, starting from iodinated compound **(*S*)-32** (176 mg, 0.35 mmol) with a reaction time of 30 min. Yield: 89%; R_f (SiO_2, cyclohexane/ethyl acetate, 9/1, v/v, iodine revelation): 0.15; IR (ATR accessory) ν 2976, 2901, 1701, 1391, 1367, 1243, 1158, 1015 cm^{-1}; ^{1}H NMR (500.13 MHz, $CDCl_3$) δ 0.25 (s, 18H, $^2J_{H\text{-}^{119}Sn}$ = 56.2 Hz, $^2J_{H\text{-}^{117}Sn}$ = 53.9 Hz), 1.14 (t, 3H, ^3J = 7.0 Hz), 1.16 (t, 3H*, ^3J = 7.0 Hz), 1.21 (t, 3H*, ^3J = 7.3 Hz), 1.23 (t, 3H, ^3J = 7.1 Hz), 1.44 (s, 9H*), 1.51 (s, 9H), 3.07 (m, 3H), 3.19 (dd, 1H*, ^2J = 15.7 Hz, ^3J = 2.8 Hz), 3.68 (m, 4H), 4.08 (m, 4H), 4.41 (d, 1H*, ^2J = 16.2 Hz), 4.48 (d, 1H, ^2J = 16.7 Hz), 4.70 (m, 3H), 5.09 (dd, 1H*, ^3J = 6.2, 2.8 Hz), 5.17 (m, 4H), 6.80 (s, 1H*, $^4J_{H\text{-}^{117/119}Sn}$ = 17.1 Hz), 6.87 (s, 1H, $^4J_{H\text{-}^{117/119}Sn}$ = 17.2 Hz), 7.09 (s, 1H, $^3J_{H\text{-}^{117/119}Sn}$ = 48.2 Hz), 7.10 (s, 1H*, $^3J_{H\text{-}^{117/119}Sn}$ = 48.7 Hz); ^{13}C NMR (125.77 MHz, $CDCl_3$) δ -9.0 (6C, $^1J_{C\text{-}^{119}Sn}$ = 362 Hz, $^1J_{C\text{-}^{117}Sn}$ = 346 Hz), 14.2, 14.3*, 15.2 (2C), 28.5* (3C), 28.6 (3C), 30.5, 31.0*, 44.5*, 45.1, 52.9, 54.8*, 61.1 (2C), 64.3 (2C), 80.5*, 80.6, 92.9, 93.0*, 109.6, 110.2*, 125.1, 125.6*, 129.2, 129.3*, 134.9, 135.7*, 136.1*, 136.5, 155.0*, 155.7, 160.7, 160.9*, 171.7, 172.3*; HRMS: *m*/*z* calculated for $C_{23}H_{38}NO_6Sn^+$ [M+H]$^+$: 544.1716, found: 544.11704.

*On the NMR spectra, two rotamers were evidenced in 55% vs 45% proportions. The symbol * refers to the minor rotamer.*

(3*R*)-2-*tert*-butyl 3-ethyl 7-(ethoxymethoxy)-6-(trimethylstannyl)-3,4-dihydroisoquinoline-2,3(1*H*)-dicarboxylate ((*R*)-35)

Compound **(*R*)-35** (yellowish oil, 544 mg, 1.00 mmol) was synthesized according to the general protocol described above, starting from iodinated compound **(*R*)-32** (625 mg, 1.24 mmol) with a reaction time of 1 h. Yield: 81%; R_f (SiO_2, cyclohexane/ethyl acetate, 9/1, v/v): 0.15; IR (ATR accessory) 2976, 2938, 1698, 1389, 1321, 1163, 1012 ν cm^{-1}; ^{1}H NMR (500.13 MHz, $CDCl_3$) δ 0.25 (s, 18H, $^2J_{H\text{-}^{119}Sn}$ = 56.2 Hz, $^2J_{H\text{-}^{117}Sn}$ = 53.8 Hz), 1.15 (t, 3H, ^3J = 7.1 Hz), 1.16 (t, 3H*, ^3J = 7.0 Hz), 1.22 (t, 3H*, ^3J = 7.3 Hz), 1.23 (t, 3H, ^3J = 7.2 Hz), 1.45 (s, 9H*), 1.52 (s, 9H), 3.07 (m, 3H), 3.19 (dd, 1H*, ^2J = 15.7 Hz, ^3J = 2.8 Hz), 3.69 (m, 4H), 4.09 (m, 4H), 4.42 (d, 1H*, ^2J = 16.2 Hz), 4.49 (d, 1H, ^2J = 16.7 Hz), 4.70 (m, 3H), 5.10 (dd, 1H*, ^3J = 2.8, 6.2 Hz), 5.18 (m, 4H), 6.81 (s, 1H*, $^4J_{H\text{-}^{117/119}Sn}$ = 17.1 Hz), 6.88 (s, 1H, $^4J_{H\text{-}^{117/119}Sn}$ = 17.2 Hz), 7.10 (s, 1H, $^3J_{H\text{-}^{117/119}Sn}$ = 48.5 Hz), 7.11 (s, 1H*, $^3J_{H\text{-}^{117/119}Sn}$ = 48.3 Hz); ^{13}C NMR (125.8 MHz, $CDCl_3$) δ -9.0 (6C, $^1J_{C\text{-}^{119}Sn}$ = 362 Hz, $^1J_{C\text{-}^{117}Sn}$ = 346 Hz), 14.2, 14.3*, 15.2 (2C), 28.5* (3C), 28.6 (3C), 30.5, 31.0*, 44.5*, 45.1, 52.9, 54.8*, 61.1*, 61.2, 64.3 (2C), 80.6 (2C), 92.9, 93.0*, 109.6, 110.2*, 125.1, 125.6*, 129.2, 129.3*, 134.9, 135.7*, 136.1*, 136.5, 155.0*, 155.7, 160.7, 160.9*, 171.8, 172.3*; HRMS: m/z calculated for $C_{23}H_{38}NO_6Sn^+$ $[M+H]^+$: 544.1716, found: 544.1711.
*On the NMR spectra, two rotamers were evidenced in 56% vs 44% proportions. The symbol * refers to the minor rotamer.*

General method for the syntheses of 2-*tert*-butyl 3-ethyl 7-(ethoxymethoxy)-8-(trimethylstannyl)-3,4-dihydroisoquinoline-2,3(1*H*)-dicarboxylates ((*R*/*S*)-36)

A solution of 1.3 M isopropylmagnesium chloride lithium chloride complex solution in tetrahydrofuran (1.05 mL, 1.37 mmol, 1.2 eq.) was added dropwise, under argon and at -40 °C, to a solution of iodinated compounds **(*R*/*S*)-33** (577 mg, 1.14 mmol) in anhydrous tetrahydrofuran (0.9 mL). After stirring at -40 °C for 30 min, a solution of 1 M trimethyltin chloride in tetrahydrofuran (1.71 mL, 1.71 mmol, 1.5 eq.) was dropwise added. The reaction mixture was then stirred at -40 °C for 1 h. After return back to room temperature during 2 h, the reaction mixture was evaporated under reduced pressure and the residue was purified by column chromatography (SiO_2, cyclohexane/ethyl acetate, 8/2, v/v) to give stannanes **(*R*/*S*)-36**.

(3*S*)-2-*tert*-butyl 3-ethyl 7-(ethoxymethoxy)-8-(trimethylstannyl)-3,4-dihydroisoquinoline-2,3(1*H*)-dicarboxylate ((*S*)-36)

Compound **(*S*)-36** (colorless oil, 498 mg, 0.92 mmol) was synthesized according to the general protocol described above, starting from iodinated compound **(*S*)-33** (577 mg, 1.14 mmol). Yield: 81%; R_f (SiO_2, cyclohexane/ethyl acetate, 8/2, v/v, iodine revelation): 0.49; IR (ATR accessory) ν 2976, 2906, 1738, 1698, 1390, 1366, 1163, 1020 cm^{-1}; ^{1}H NMR (500.13 MHz, $CDCl_3$) δ 0.34 (s, 9H, $^2J_{H\text{-}^{119}Sn}$ = 55.8 Hz, $^2J_{H\text{-}^{117}Sn}$ = 53.8 Hz), 0.37 (s, 9H*, $^2J_{H\text{-}^{119}Sn}$ = 55.7 Hz, $^2J_{H\text{-}^{117}Sn}$ = 54.2 Hz), 1.15 (t, 3H, ^3J = 7.1 Hz), 1.18 (t, 3H*, ^3J = 7.1 Hz), 1.22 (t, 6H, ^3J = 7.1 Hz), 1.44 (s, 9H*), 1.53 (s, 9H), 3.02 (dd, 1H*, ^2J = 15.1 Hz, ^3J = 6.1 Hz), 3.12 (m, 3H), 3.69 (q, 4H, ^3J = 7.1 Hz), 4.09 (m, 4H), 4.37 (d, 1H*, ^2J = 15.9 Hz), 4.44 (d, 1H, ^2J = 16.3 Hz), 4.62 (t, 1H*, ^3J = 6.0 Hz), 4.70 (d, 1H, ^2J = 16.3 Hz), 4.80 (d, 1H*, ^2J = 15.9 Hz), 5.00 (dd, 1H, ^3J = 3.7, 6.1 Hz), 5.15 (m, 4H), 6.94 (d, 1H, ^3J = 8.4 Hz), 6.96 (d, 1H*, ^3J = 7.4 Hz), 7.05 (d, 1H, ^3J = 8.1 Hz), 7.06 (d, 1H*, ^3J = 7.8 Hz); ^{13}C NMR (125.77

MHz, $CDCl_3$) δ -6.1 (6C, $^1J_{C-^{119}Sn}$ = 360 Hz, $^1J_{C-^{117}Sn}$ = 344 Hz), 14.2, 14.3*, 15.2 (2C), 28.5* (3C), 28.7 (3C), 31.2, 31.8*, 46.4*, 47.7, 52.9, 54.9*, 61.1 (2C), 64.3 (2C), 80.4*, 80.8, 93.2 (2C), 111.2*, 111.3, 125.3, 126.1*, 127.9, 128.6*, 129.6*, 130.5, 140.9, 142.6*, 154.8*, 155.7, 160.9, 161.1*, 171.8, 172.5*; HRMS: *m/z* calculated for $C_{23}H_{38}NO_6Sn^+$ $[M+H]^+$: 544.1716, found: 544.1705.

*On the NMR spectra, two rotamers were evidenced in 66% vs 44% proportions. The symbol * refers to the minor rotamer.*

(3*R*)-2-*tert*-butyl 3-ethyl 7-(ethoxymethoxy)-8-(trimethylstannyl)-3,4-dihydroisoquinoline-2,3(1*H*)-dicarboxylate ((*R*)-36)

Compound **(*R*)-36** (colorless oil, 400 mg, 0.74 mmol) was synthesized according to the general protocol described above, starting from iodinated compound **(*R*)-33** (593 mg, 1.17 mmol). Yield: 63%; R_f (SiO_2, cyclohexane/ethyl acetate, 8/2, v/v, iodine revelation): 0.49; IR (ATR accessory) ν 2976, 2910, 1738, 1698, 1390, 1365, 1163, 1020 cm^{-1}; ^1H NMR (500.13 MHz, $CDCl_3$) δ 0.34 (s, 9H, $^2J_{H-^{119}Sn}$ = 55.8 Hz, $^2J_{H-^{117}Sn}$ = 53.7 Hz), 0.37 (s, 9H*, $^2J_{H-^{119}Sn}$ = 55.7 Hz, $^2J_{H-^{117}Sn}$ = 54.1 Hz), 1.15 (t, 3H, ^3J = 7.1 Hz), 1.18 (t, 3H*, ^3J = 7.1 Hz), 1.22 (t, 6H, ^3J = 7.1 Hz), 1.44 (s, 9H*), 1.53 (s, 9H), 3.02 (dd, 1H*, ^2J = 15.1 Hz, ^3J = 6.1 Hz), 3.12 (m, 3H), 3.69 (q, 4H, ^3J = 7.1 Hz), 4.08 (m, 4H), 4.37 (d, 1H*, ^2J = 15.9 Hz), 4.44 (d, 1H, ^2J = 16.3 Hz), 4.62 (t, 1H*, ^3J = 6.0 Hz), 4.70 (d, 1H, ^2J = 16.3 Hz), 4.80 (d, 1H*, ^2J = 15.9 Hz), 5.00 (dd, 1H, ^3J = 3.7, 6.1 Hz), 5.16 (m, 4H), 6.94 (d, 1H, ^3J = 8.2 Hz), 6.96 (d, 1H*, ^3J = 7.4 Hz), 7.04 (d, 1H, ^3J = 8.1 Hz), 7.06 (d, 1H*, ^3J = 7.7 Hz); ^{13}C NMR (125.77 MHz, $CDCl_3$) δ -6.1 (6C, $^1J_{C-^{119}Sn}$ = 360 Hz, $^1J_{C-^{117}Sn}$ = 344 Hz), 14.2, 14.3*, 15.2 (2C), 28.5* (3C), 28.7 (3C), 31.2, 31.8*, 46.4*, 47.7, 52.9, 54.9*, 61.1*, 61.2, 64.3 (2C), 80.4*, 80.8, 93.2 (2C), 111.2*, 111.3, 125.3, 126.1*, 127.9, 128.6*, 129.6*, 130.5, 140.9, 142.6*, 154.8*, 155.7, 160.9, 161.1*, 171.8, 172.5*; HRMS: *m/z* calculated for $C_{23}H_{38}NO_6Sn^+$ $[M+H]^+$: 544.1716, found: 544.1708.

*On the NMR spectra, two rotamers were evidenced in 65% vs 45% proportions. The symbol * refers to the minor rotamer.*

General method for the syntheses of fluoro-1,2,3,4-tetrahydro-7-hydroxyisoquinoline-3-carboxylic acids, trifluoroacetate salts (*R*/*S*)-37 (5-fluoro-L/D-TIC(OH)), (*R*/*S*)-38 (6-fluoro-L/D-TIC(OH)) and (*R*/*S*)-39 (8-fluoro-L/D-TIC(OH)).

To a solution of **(*R*/S)-34**, **(*R*/S)-35** or **(*R*/S)-36** (0.72 mmol) in anhydrous acetone (7 mL) were successively added in a vial, under argon, anhydrous silver(I) oxide (34.5 μmol, 0.05 eq.), anhydrous sodium hydrogen carbonate (1.44 mmol, 2 eq.), anhydrous sodium triflate (0.72 mmol, 1 eq.) and anhydrous 1-(chloromethyl)-4-fluoro-1,4-diazabicyclo[2.2.2]octane-1,4-diium bis(hexafluorophosphate) (F-TEDA-PF_6) (1.08 mmol, 1.5 eq.). The vial was sealed and the mixture was heated at 40-80 °C for 2-4 h. After cooling to room temperature, the reaction mixture was filtered over celite 545® and washed with acetone (3 × 5 mL). The filtrate was evaporated under vacuum and the residue was purified by column chromatography (SiO_2, cyclohexane/ethyl acetate, 8/2, v/v) to give mixtures of expected fluorinated compounds and the corresponding hydrodestannylated product ((*R/S*)-2-*tert*-butyl 3-ethyl 7-(ethoxymethoxy)-3,4-dihydroisoquinoline-2,3(1*H*)-dicarboxylate). A solution of the previous mixture in 7.2 M hydrogen chloride in dioxane (1 mL) was stirred at room temperature for 5 min. The solution was then evaporated under reduced pressure to dryness and the residue was dissolved in a mixture of tetrahydrofuran and methanol (3 mL, 3/1, v/v) before addition of a 1 M aqueous solution of lithium hydroxide (0.8 mL). After stirring at room temperature for 1-15 h, the organic solvents were evaporated under vacuum. The resulting aqueous phase was acidified until pH 1-2 by addition of an aqueous solution of trifluoroacetic acid (1/1, v/v) and purified by preparative RP-HPLC to give after freeze-drying compounds **(*R*/S)-37**, **(*R*/S)-38** or **(*R*/S)-39**.

(3*S*)-5-fluoro-1,2,3,4-tetrahydro-7-hydroxyisoquinoline-3-carboxylic acid, trifluoroacetate salt ((*S*)-37, 5-fluoro-L-TIC(OH))

Compound **(*S*)-37** (white solid, 31.9 mg, 0.098 mmol) was synthesized according to the general protocol described above, starting from stannane **(*S*)-34** (446 mg, 0.82 mmol) with a reaction time with F-TEDA-PF_6 of 2 h at 60 °C and a saponification time of 2 h. Yield: 12%; t_R (preparative RP-HPLC) = 13.5-17.5 min; mp 295-297 °C; IR (ATR accessory) ν 2200-3200, 1731, 1632, 1190, 1147 cm^{-1}; ^{1}H NMR (500.13 MHz, CD_3OD) δ 2.94 (dd, 1H, ^2J = 17.2 Hz, ^3J = 11.5 Hz), 3.37 (dd, 1H, ^2J = 17.1 Hz, ^3J = 5.4 Hz), 4.24 (dd, 1H, ^3J = 5.4, 11.5 Hz), 4.36 (m, 2H), 6.50 (d, 1H, ^4J = 2.0 Hz), 6.53 (d, 1H, $^3J_{H-F}$ = 11.1 Hz, ^4J = 2.2 Hz); ^{13}C NMR (125.8 MHz, CD_3OD) δ 23.2 (d, $^3J_{C-F}$ = 4.3 Hz), 45.3 (d, $^4J_{C-F}$ = 2.7 Hz), 55.6, 103.4 (d, $^2J_{C-F}$ = 23.8 Hz), 109.8 (d, 1H, $^4J_{C-F}$ = 2.9 Hz), 110.1 (d, $^2J_{C-F}$ = 19.8 Hz), 118.2 (q, $^1J_{C-F}$ = 292.8 Hz), 131.5 (d, $^3J_{C-F}$ = 6.8 Hz), 159.4 (d, $^3J_{C-F}$ = 12.2 Hz), 162.3 (d, $^1J_{C-F}$ = 244.6 Hz), 163.0 (q, $^2J_{C-F}$ = 34.3 Hz), 171.4; ^{19}F NMR (470.59 MHz, CD_3OD) δ -76.42 (3F), -118.31; HRMS: m/z calculated for $C_{10}H_{11}FNO_3^+$ $[M+H]^+$: 212.0717, found: 212.0714; e.e. (Method B): t_R = 7.6 min, 94.0%.

(3*R*)-5-fluoro-1,2,3,4-tetrahydro-7-hydroxyisoquinoline-3-carboxylic acid, trifluoroacetate salt ((*R*)-37, 5-fluoro-D-TIC(OH))

Compound **(*R*)-37** (white solid, 27.9 mg, 0.086 mmol) was synthesized according to the general protocol described above, starting from stannane **(*R*)-34** (481 mg, 0.89 mmol) with a reaction time with F-TEDA-PF_6 of 2 h at 40 °C and a saponification time of 2 h. Yield: 10%; t_R (preparative RP-HPLC) = 18-21 min; mp 274-276 °C; IR (ATR accessory) ν 2200-3200, 1726, 1670, 1189, 1150 cm^{-1}; ^{1}H NMR (500.13 MHz, CD_3OD) δ 2.95 (dd, 1H, ^2J = 17.3 Hz, ^3J = 11.6 Hz), 3.38 (dd, 1H, ^2J = 17.1 Hz, ^3J = 5.4 Hz), 4.31 (dd, 1H, ^3J = 5.5, 11.6 Hz), 4.76 (m, 2H), 6.51 (d, 1H, ^4J = 2.0 Hz), 6.54 (d, 1H, $^3J_{H-F}$ = 11.1 Hz, ^4J = 2.1 Hz); ^{13}C NMR (125.8 MHz, CD_3OD) δ 23.1 (d, $^3J_{C-F}$ = 4.4 Hz), 45.3 (d, $^4J_{C-F}$ = 2.6 Hz), 55.3, 103.4 (d, $^2J_{C-F}$ = 23.8 Hz), 109.8 (d, 1H, $^4J_{C-F}$ = 3.0 Hz), 109.9 (d, $^2J_{C-F}$ = 18.6 Hz), 118.1 (q, $^1J_{C-F}$ = 292.3 Hz), 131.5 (d, $^3J_{C-F}$ = 6.8 Hz), 159.4 (d, $^3J_{C-F}$ = 12.1 Hz), 162.3 (d, $^1J_{C-F}$ = 244.6 Hz), 162.8 (q, $^2J_{C-F}$ = 35.0 Hz), 171.1; ^{19}F NMR (470.59 MHz, CD_3OD) δ -76.41 (3F), -118.31; HRMS: m/z calculated for $C_{10}H_{11}FNO_3^+$ $[M+H]^+$: 212.0717, found: 212.0714; e.e. (Method B): t_R = 13.4 min, 97.2%.

(3*S*)-6-fluoro-1,2,3,4-tetrahydro-7-hydroxyisoquinoline-3-carboxylic acid, trifluoroacetate salt ((*S*)-38, 6-fluoro-L-TIC(OH))

Compound **(*S*)-38** (white solid, 43.6 mg, 0.13 mmol) was synthesized according to the general protocol described above, starting from stannane **(*S*)-35** (390 mg, 0.72 mmol) with a reaction time with F-TEDA-PF_6 of 3 h at 80 °C and a saponification time of 1 h. Yield: 19%; t_R (preparative RP-HPLC) = 9.8-12 min; mp 296-298 °C; IR (ATR accessory) ν 2200-3200, 1703, 1629, 1193, 1143 cm^{-1}; ^{1}H NMR (500.13 MHz, CD_3OD) δ 3.10 (dd, 1H, ^2J = 16.9 Hz, ^3J = 11.5 Hz), 3.34 (dd, 1H, ^2J = 16.9 Hz, ^3J = 5.2 Hz), 4.27 (dd, 1H, ^3J = 5.2, 11.5 Hz), 4.32 (m, 2H), 6.78 (d, 1H, $^4J_{H-F}$ = 8.5 Hz), 7.00 (d, 1H, $^3J_{H-F}$ = 11.4 Hz); ^{13}C NMR (125.8 MHz, CD_3OD) δ 28.9, 45.1, 55.7, 116.4 (d, $^3J_{C-F}$ = 3.1 Hz), 116.7 (q, $^1J_{C-F}$ = 292.6 Hz), 117.0 (d, 1H, $^2J_{C-F}$ = 19.5 Hz), 123.1 (d, $^3J_{C-F}$ = 6.6 Hz), 124.8 (d, $^4J_{C-F}$ = 3.3 Hz), 145.8 (d, $^2J_{C-F}$ = 13.6 Hz), 152.7 (d, $^1J_{C-F}$ = 242.5 Hz), 161.5 (q, $^2J_{C-F}$ = 36.0 Hz), 171.3; ^{19}F NMR (470.59 MHz, CD_3OD) δ -76.43 (3F), -138.60 ; HRMS: m/z calculated for $C_{10}H_{11}FNO_3^+$ $[M+H]^+$: 212.0717, found: 212.0714; e.e. (Method C): t_R = 9.2 min, 100%.

(3*R*)-6-fluoro-1,2,3,4-tetrahydro-7-hydroxyisoquinoline-3-carboxylic acid, trifluoroacetate salt ((*R*)-38, 6-fluoro-D-TIC(OH))

Compound **(*R*)-38** (white solid, 53.0 mg, 0.16 mmol) was synthesized according to the general protocol described above, starting from stannane **(*R*)-35** (524 mg, 0.97 mmol) with a reaction time with F-TEDA-PF_6 of 3 h at 80 °C and a saponification time of 1 h. Yield: 16%; t_R (preparative RP-HPLC) = 9.5-12.5 min; mp 293-295 °C; IR (ATR accessory) ν 2200-3200, 1726, 1637, 1183, 1140 cm^{-1}; ^1H NMR (500.13 MHz, CD_3OD) δ 3.10 (dd, 1H, ^2J = 16.9 Hz, ^3J = 11.5 Hz), 3.34 (dd, 1H, ^2J = 16.9 Hz, ^3J = 5.2 Hz), 4.27 (dd, 1H, ^3J = 5.2, 11.5 Hz), 4.32 (m, 2H), 6.78 (d, 1H, $^4J_{H-F}$ = 8.5 Hz), 7.00 (d, 1H, $^3J_{H-F}$ = 11.4 Hz); ^{13}C NMR (125.8 MHz, CD_3OD) δ 29.0, 45.1, 55.7, 116.4 (d, $^3J_{C-F}$ = 3.1 Hz), 119.3 (q, $^1J_{C-F}$ = 291.8 Hz), 117.0 (d, 1H, $^2J_{C-F}$ = 19.5 Hz), 123.1 (d, $^3J_{C-F}$ = 6.7 Hz); 124.8 (d, $^4J_{C-F}$ = 3.4 Hz), 145.8 (d, $^2J_{C-F}$ = 13.7 Hz), 152.7 (d, $^1J_{C-F}$ = 242.5 Hz), 163.0 (q, $^2J_{C-F}$ = 34.4 Hz), 171.4; ^{19}F NMR (470.59 MHz, CD_3OD) δ -76.44 (3F), -138.49 , HRMS: m/z calculated for $C_{10}H_{11}FNO_3^+$ $[M+H]^+$: 212.0717, found: 212.0713; e.e. (Method C): t_R = 15.4 min, 93.3%.

(3*S*)-8-fluoro-1,2,3,4-tetrahydro-7-hydroxyisoquinoline-3-carboxylic acid, trifluoroacetate salt ((*S*)-39, 8-fluoro-L-TIC(OH))

Compound **(*S*)-39** (white solid, 141.3 mg, 0.43 mmol) was synthesized according to the general protocol described above, starting from stannane **(*S*)-36** (446 mg, 0.82 mmol) with a reaction time with F-TEDA-PF_6 of 4 h at 70 °C and a saponification time of 15 h. Yield: 53%; t_R (preparative RP-HPLC) = 16-22 min; mp 181-183 °C; IR (ATR accessory) ν 2200-3200, 1720, 1661, 1189, 1139 cm^{-1}; ^1H NMR (500.13 MHz, CD_3OD) δ 3.10 (dd, 1H, ^2J = 16.9 Hz, ^3J = 11.6 Hz), 3.37 (dd, 1H, ^2J = 17.0 Hz, ^3J = 5.0 Hz), 4.20 (dd, 1H, ^3J = 5.0, 11.6 Hz), 4.31 (d, 1H, ^2J = 16.1 Hz), 4.50 (d, 1H, ^2J = 16.1 Hz), 6.92 (m, 2H); ^{13}C NMR (125.8 MHz, CD_3OD) δ 28.8 (d, $^4J_{C-F}$ = 1.3 Hz), 40.6 (d, $^3J_{C-F}$ = 7.5 Hz), 55.7, 117.5 (d, $^2J_{C-F}$ = 13.6 Hz), 118.1 (q, $^1J_{C-F}$ = 292.6 Hz), 118.9 (d, 1H, $^3J_{C-F}$ = 3.2 Hz), 123.3 (d, $^3J_{C-F}$ = 1.7 Hz), 125.6 (d, $^4J_{C-F}$ = 4.0 Hz), 144.9 (d, $^2J_{C-F}$ = 11.7 Hz), 149.4 (d, $^1J_{C-F}$ = 241.3 Hz), 162.9 (q, $^2J_{C-F}$ = 34.9 Hz), 171.4; ^{19}F NMR (470.59 MHz, CD_3OD) δ -76.47 (3F), -144.71; HRMS: m/z calculated for $C_{10}H_{11}FNO_3^+$ $[M+H]^+$: 212.0717, found: 212.0713; e.e. (Method C): t_R = 8.0 min, 100%.

(3*R*)-8-fluoro-1,2,3,4-tetrahydro-7-hydroxyisoquinoline-3-carboxylic acid, trifluoroacetate salt ((*R*)-39, 8-fluoro-D-TIC(OH))

Compound **(*R*)-39** (white solid, 48.9 mg, 0.15 mmol) was synthesized according to the general protocol described above, starting from stannane **(*R*)-36** (351 mg, 0.65 mmol) with a reaction time with F-TEDA-PF_6 of 3 h at 50 °C and a saponification time of 5 h. A purification by preparative RP-HPLC (20 mM ammonium formate in water (A) and ethanol (B); 0-17 min: isocratic elution 99% A; 17-25 min: gradient elution 99%-0% A; 40-45 min: isocratic elution 100% B; flow rate: 10 $mL.min^{-1}$) was performed to isolate **(*R*)-39** from the corresponding hydrodestannylated product, followed by a second purification according to the general protocol to obtain **(*R*)-39** as a trifluoroacetate salt. Yield: 23%; t_R (preparative RP-HPLC) = 10.5-14 min; mp 175-177 °C; IR (ATR accessory) ν 2200-3200, 1720, 1661, 1189, 1140 cm^{-1}; ^1H NMR (500.13 MHz, CD_3OD) δ 3.10 (dd, 1H, ^2J = 17.0 Hz, ^3J = 11.6 Hz), 3.36 (dd, 1H, ^2J = 17.0 Hz, ^3J = 5.0 Hz), 4.20 (dd, 1H, ^3J = 5.0, 11.5 Hz), 4.31 (d, 1H, ^2J = 16.2 Hz), 4.50 (d, 1H, ^2J = 16.0 Hz), 6.92 (m, 2H); ^{13}C NMR (125.8 MHz, CD_3OD) δ 28.8 (d, $^4J_{C-F}$ = 1.6 Hz), 40.6 (d, $^3J_{C-F}$ = 7.5 Hz), 55.7, 117.5 (d, $^2J_{C-F}$ = 13.7 Hz), 118.1 (q, $^1J_{C-F}$ = 292.6 Hz), 118.9 (d, 1H, $^3J_{C-F}$ = 3.2 Hz), 123.3 (d, $^3J_{C-F}$ = 1.9 Hz), 125.6 (d, $^4J_{C-F}$ = 4.0 Hz), 144.9 (d, $^2J_{C-F}$ = 11.8 Hz),

149.4 (d, $^{1}J_{C\text{-}F}$ = 241.3 Hz), 162.9 (q, $^{2}J_{C\text{-}F}$ = 34.7 Hz), 171.3; ^{19}F NMR (470.59 MHz, CD_3OD) δ -76.44 (3F), -144.80; HRMS: m/z calculated for $C_{10}H_{11}FNO_3^+$ $[M+H]^+$: 212.0717, found: 212.0712. e.e. (Method C): t_R = 12.5 min, 97.1%.

2. Radiochemistry

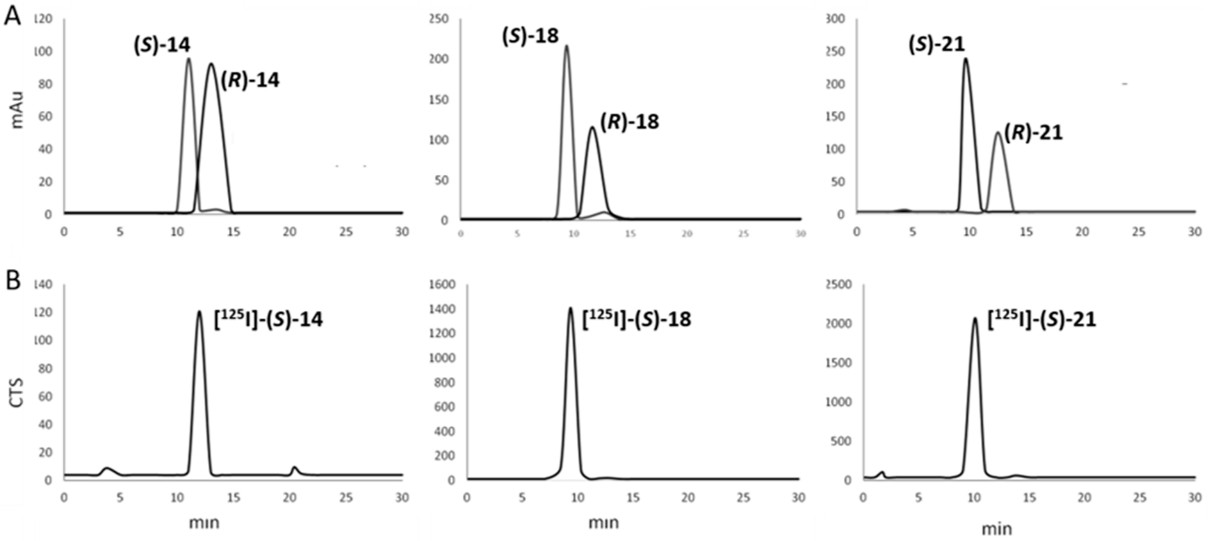

Figure S1: Analytical HPLC measurements of the enantiomeric excess of the radioiodinated tracers **[^{125}I](*S*)-14**, **[^{125}I](*S*)-18**, and **[^{125}I](*S*)-21** performed on a Reprosil Chiral-AA 8 µm column (250 x 4.6 mm; 8 µm; CIL Cluzeau; France) using the following conditions: water/ACN (30/70, v/v) as isocratic eluent mixture and a flow rate of 1 mL/min. **A:** UV chromatograms of the non-radioactive references from the L and D series; **B:** Radioactivity chromatograms of the corresponding radioiodinated tracers. The detector of radioactivity was connected in series after the UV detector accounting for the slight difference of retention times (≈ 0.5 min) observed between ^{125}I and ^{127}I products.

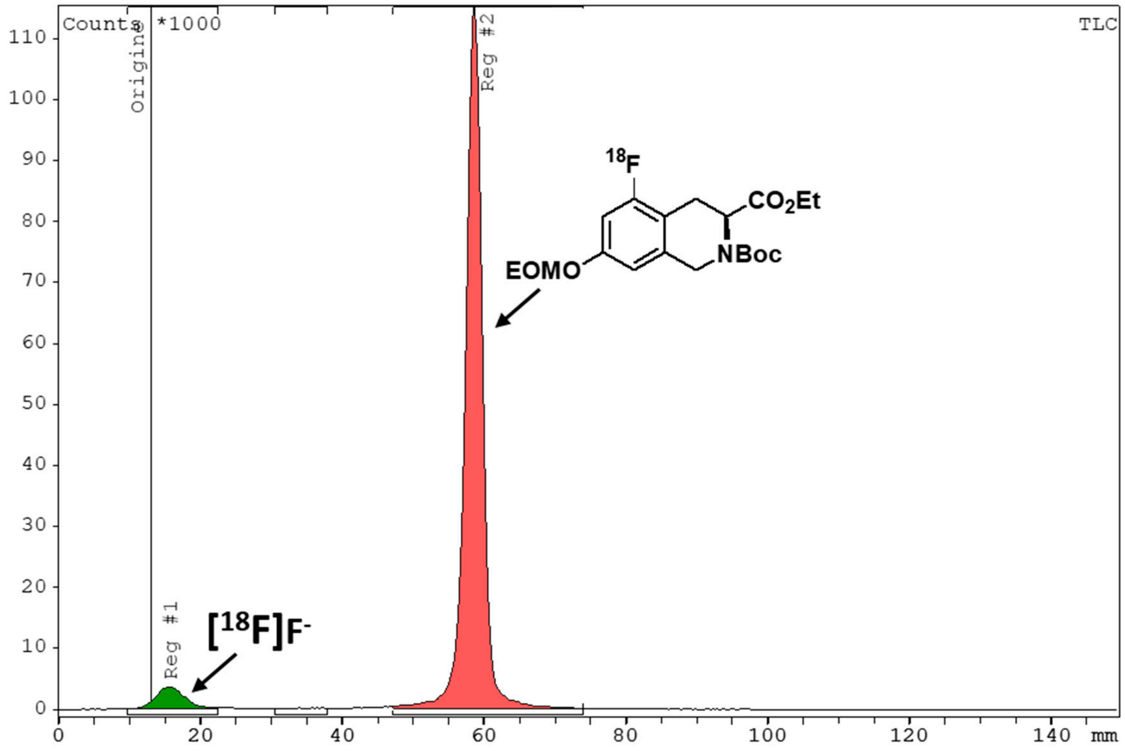

Figure S2: Representative radio-TLC (SiO_2, cyclohexane/ethyl acetate, 7/3, v/v) of the crude reaction mixture after radiofluorination of precursor **(*S*)-34** for 10 min at 110°C in DMA.

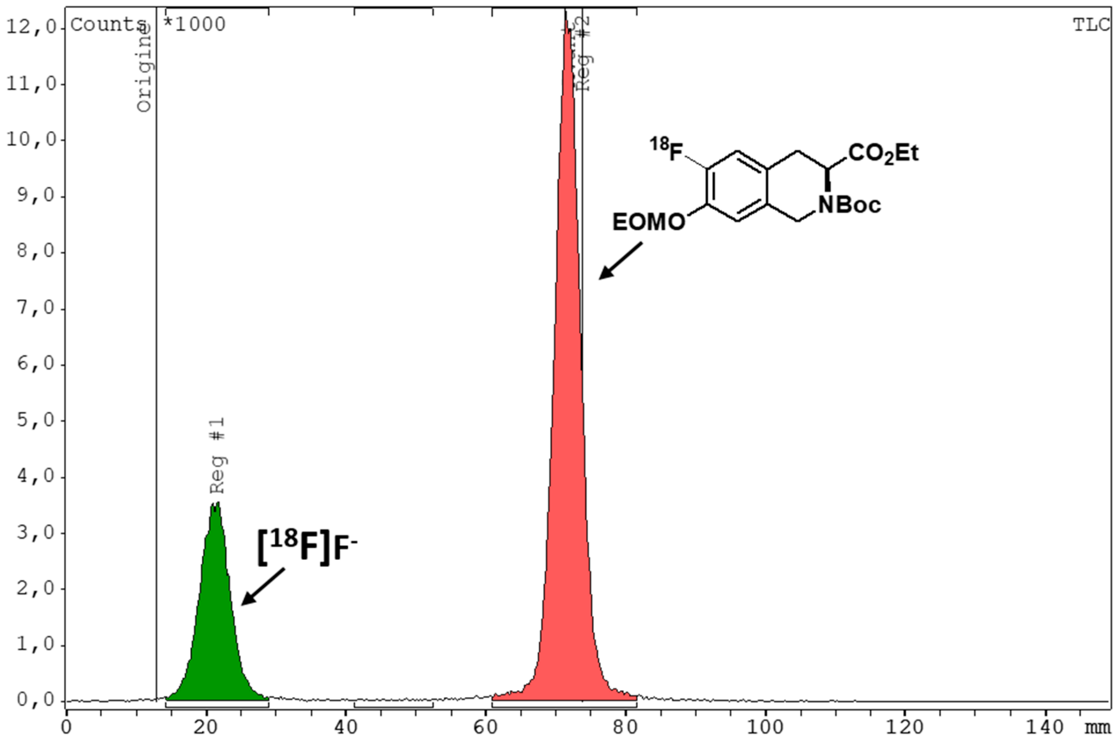

Figure S3: Representative radio-TLC of the crude reaction mixture (SiO_2, cyclohexane/ethyl acetate, 7/3, v/v) after radiofluorination of precursor **(*S*)-35** for 10 min at 110 °C in DMA.

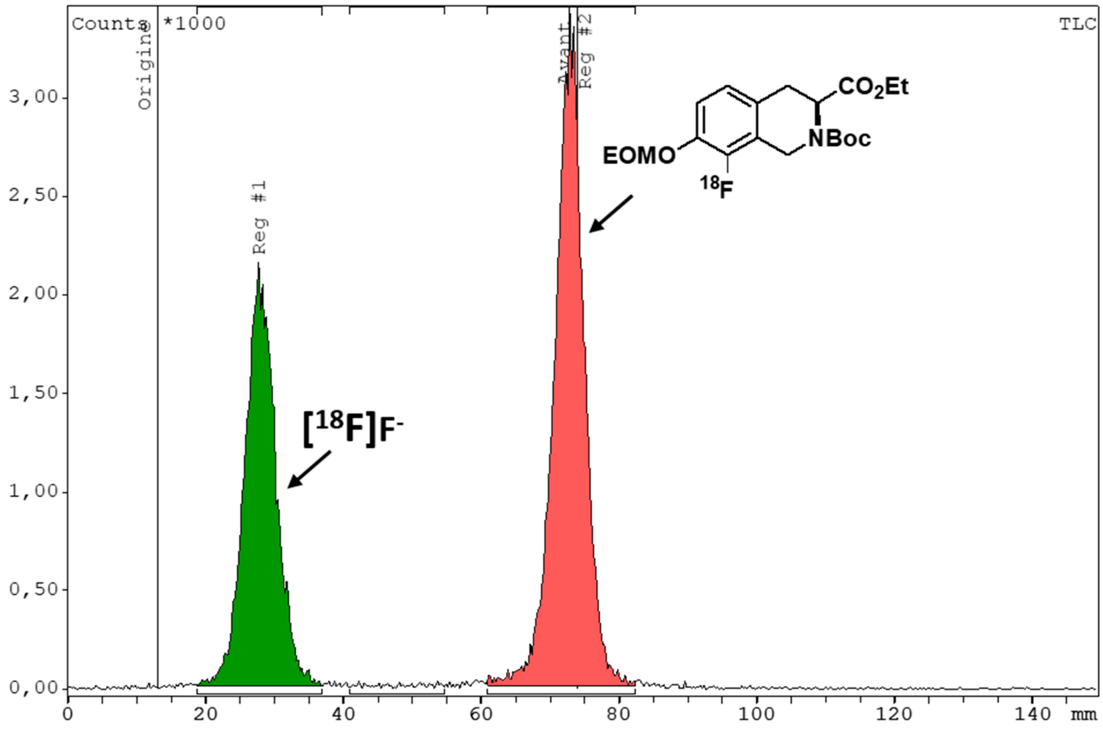

Figure S4: Representative radio-TLC (SiO_2, cyclohexane/ethyl acetate, 7/3, v/v) of the crude reaction mixture after radiofluorination of precursor **(*S*)-36** for 10 min at 110 °C in DMA.

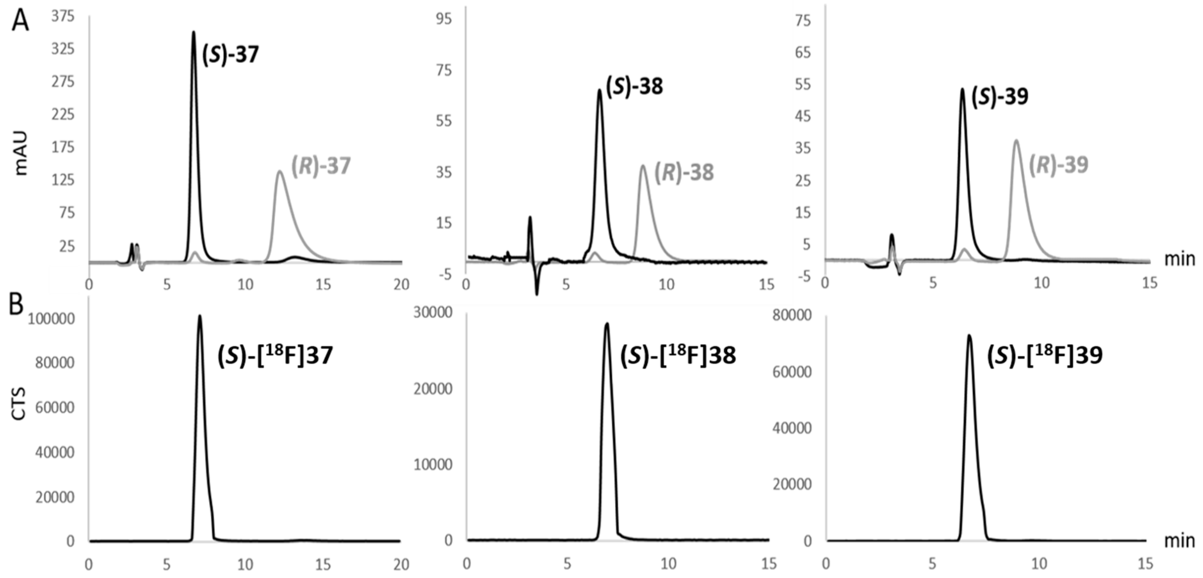

Figure S5: Analytical HPLC measurements of the enantiomeric excess of the radiofluorinated tracers **[^{18}F](*S*)-37**, **[^{18}F](*S*)-38**, and **[^{18}F](*S*)-39**, performed on a Reprosil Chiral-AA 8 µm column (250 x 4.6 mm; 8 µm; CIL Cluzeau; France) using the following conditions: water/ACN (70/30, v/v) as isocratic eluent mixture and a flow rate of 1 mL/min. **A:** UV chromatograms of the non-radioactive references from the L and D series; **B:** Radioactivity chromatograms of the corresponding radiofluorinated tracers. The detector of radioactivity was connected in series after the UV detector accounting for the slight difference of retention times (≈ 0.3 min) observed between ^{18}F and ^{19}F products.

3. ^{1}H NMR and ^{13}C NMR spectra of compounds 6-39

^{1}H (top) and ^{13}C (down) NMR spectra of compound ***(S)*-6** in DMSO-d_6

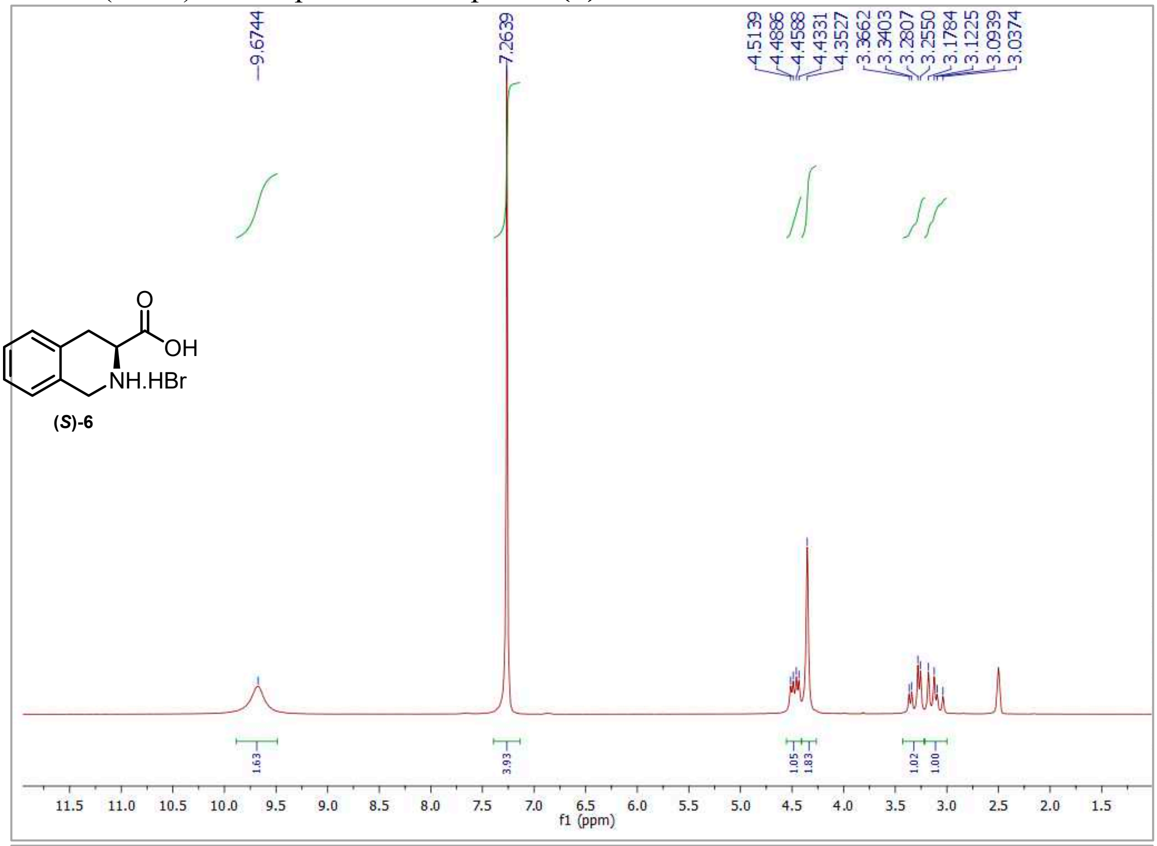

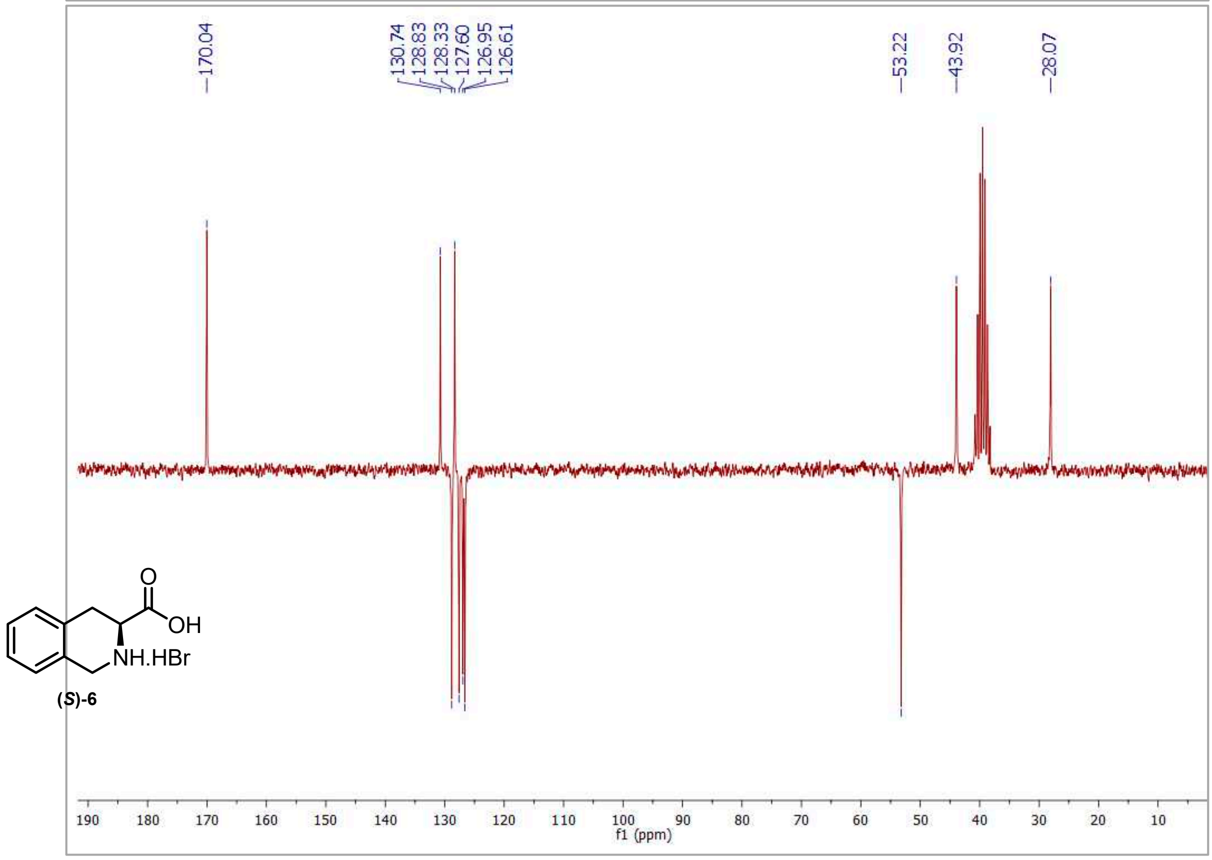

^{1}H (top) and ^{13}C (down) NMR spectra of compound ***(R)*-6** in DMSO-d_6

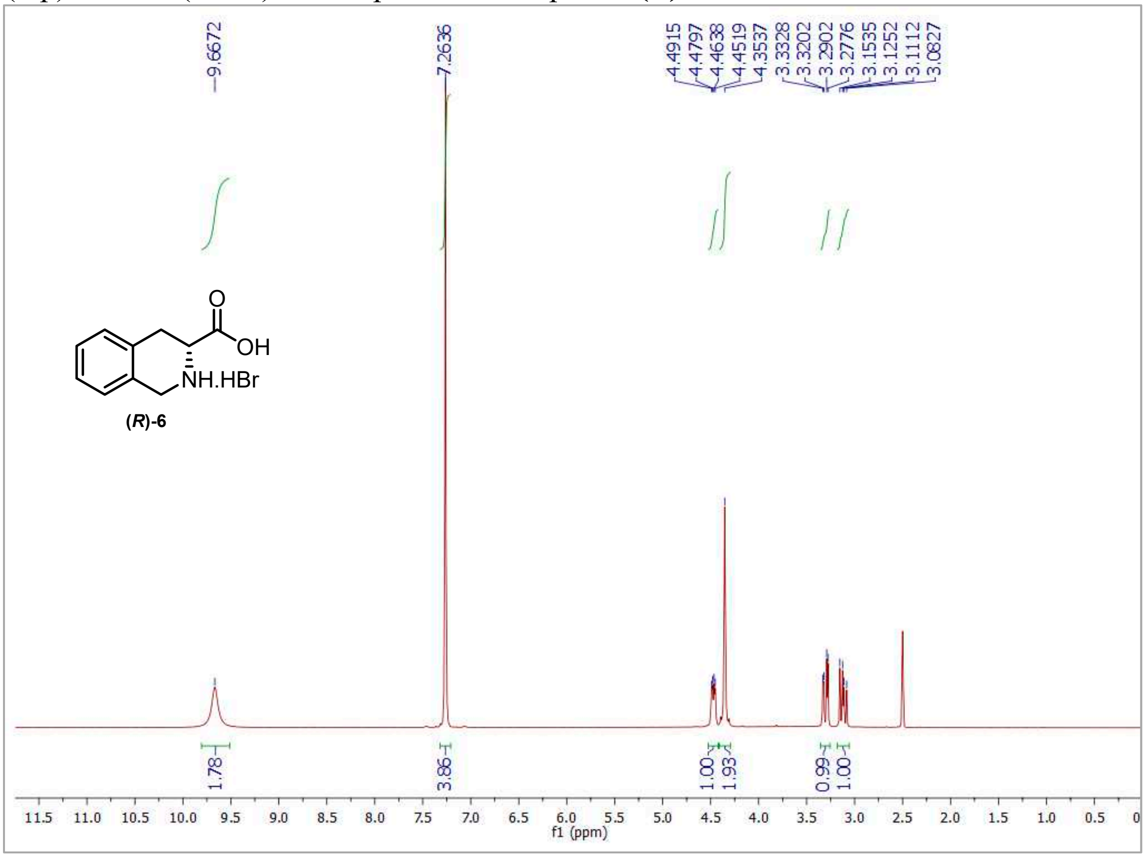

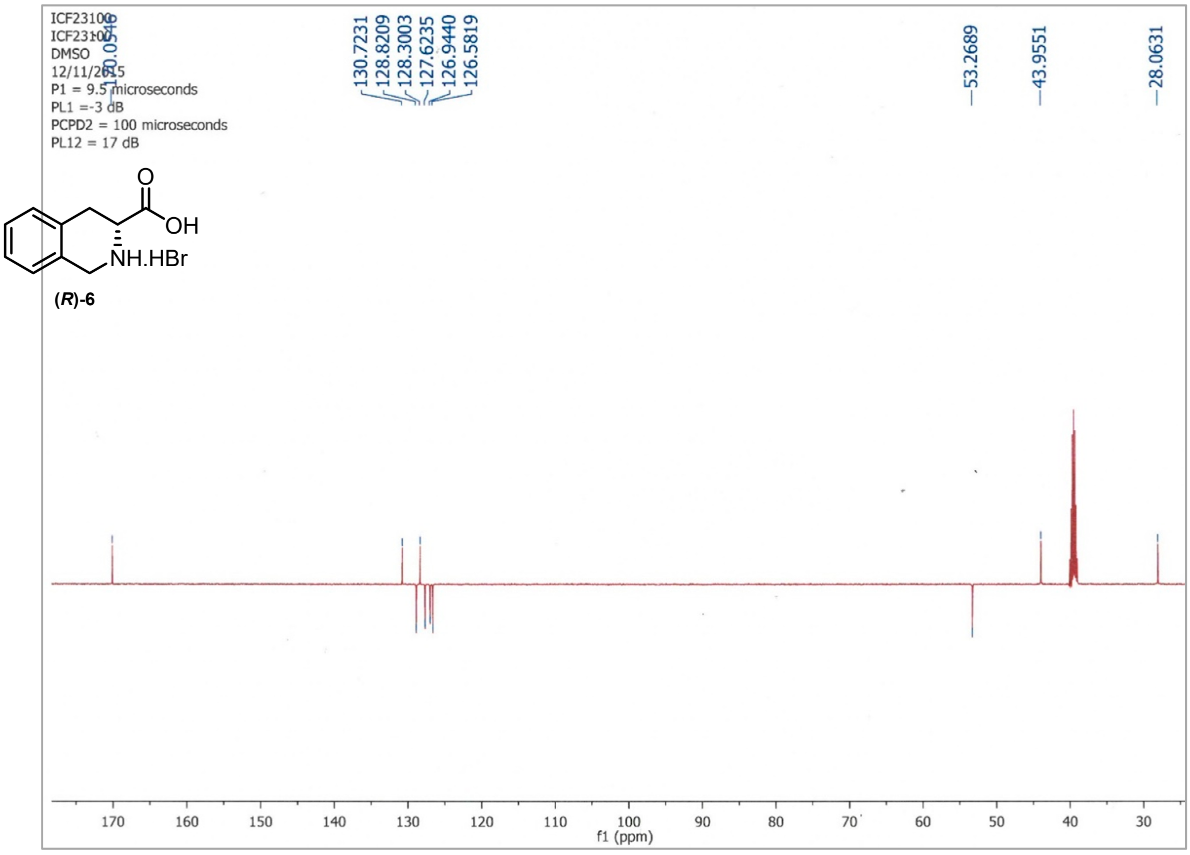

^1H (top) and ^{13}C (down) NMR spectra of compound ***(S)*-8** in DMSO-d_6

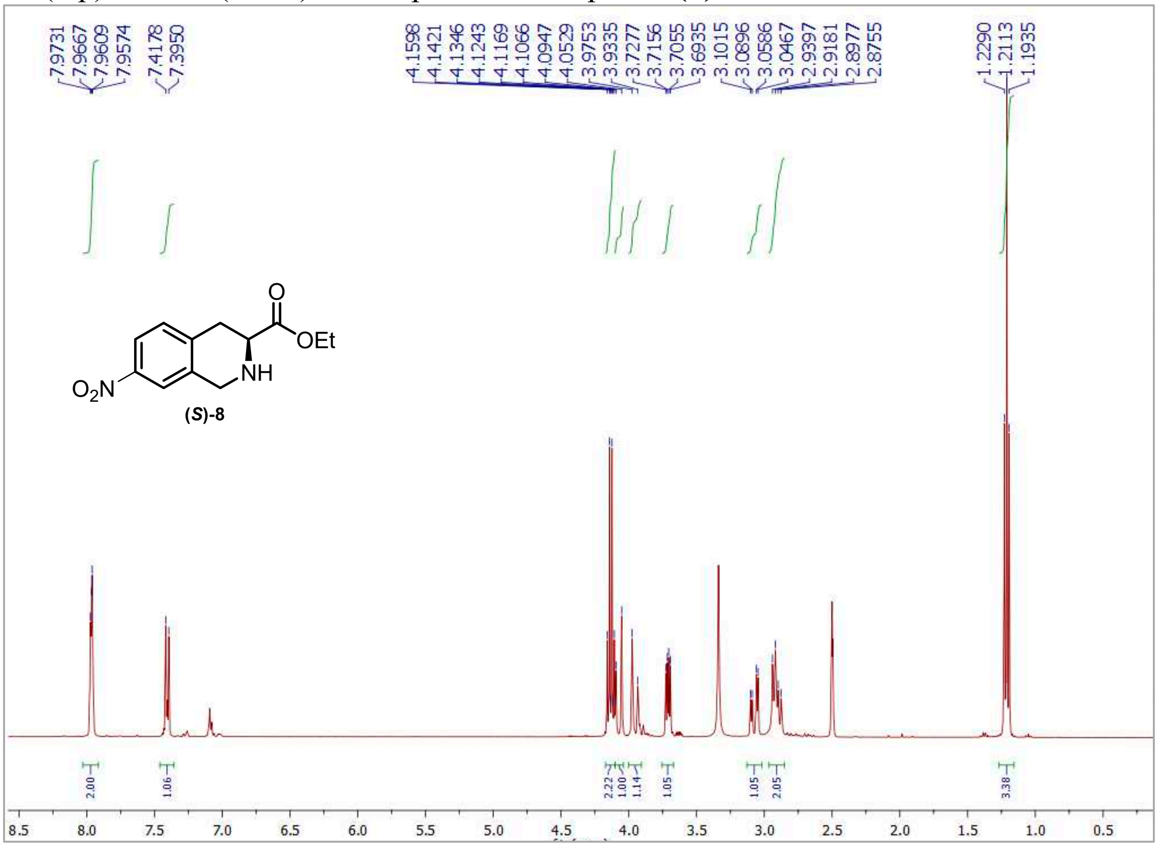

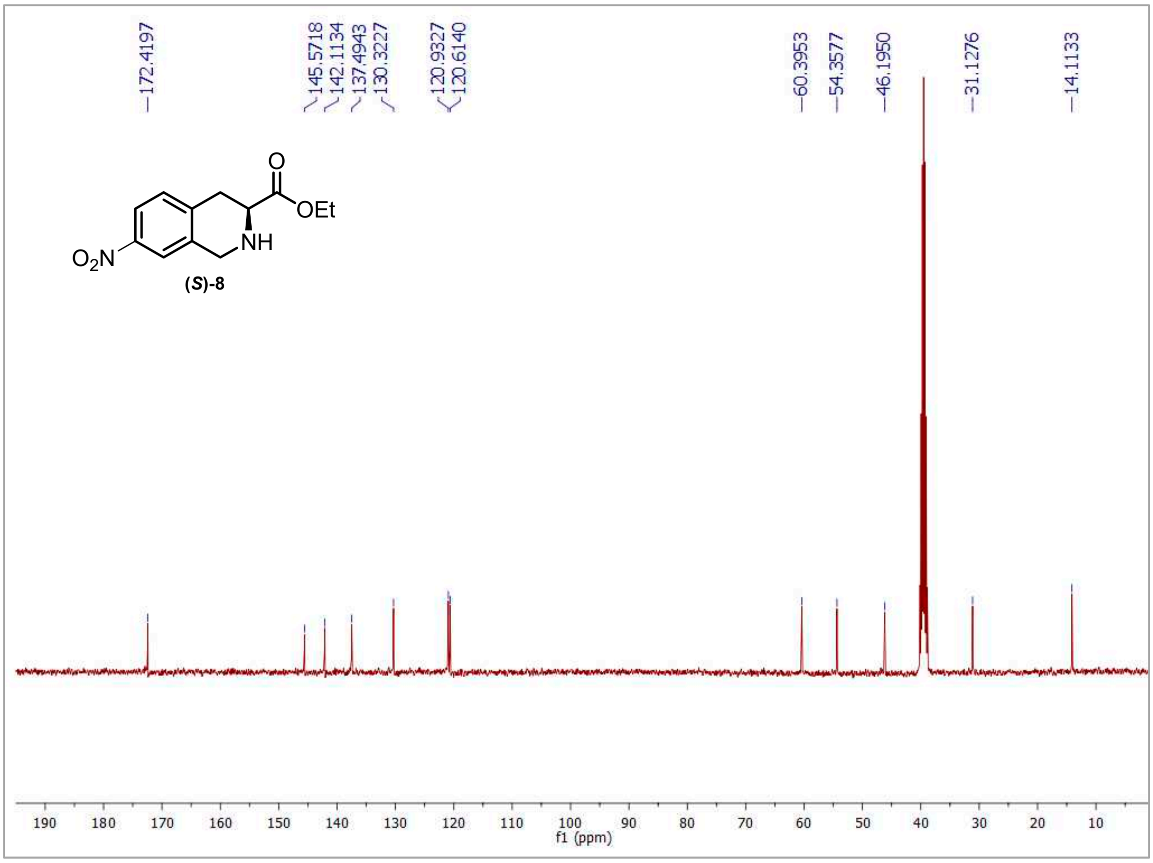

^{1}H (top) and ^{13}C (down) NMR spectra of compound ***(R)*-8**

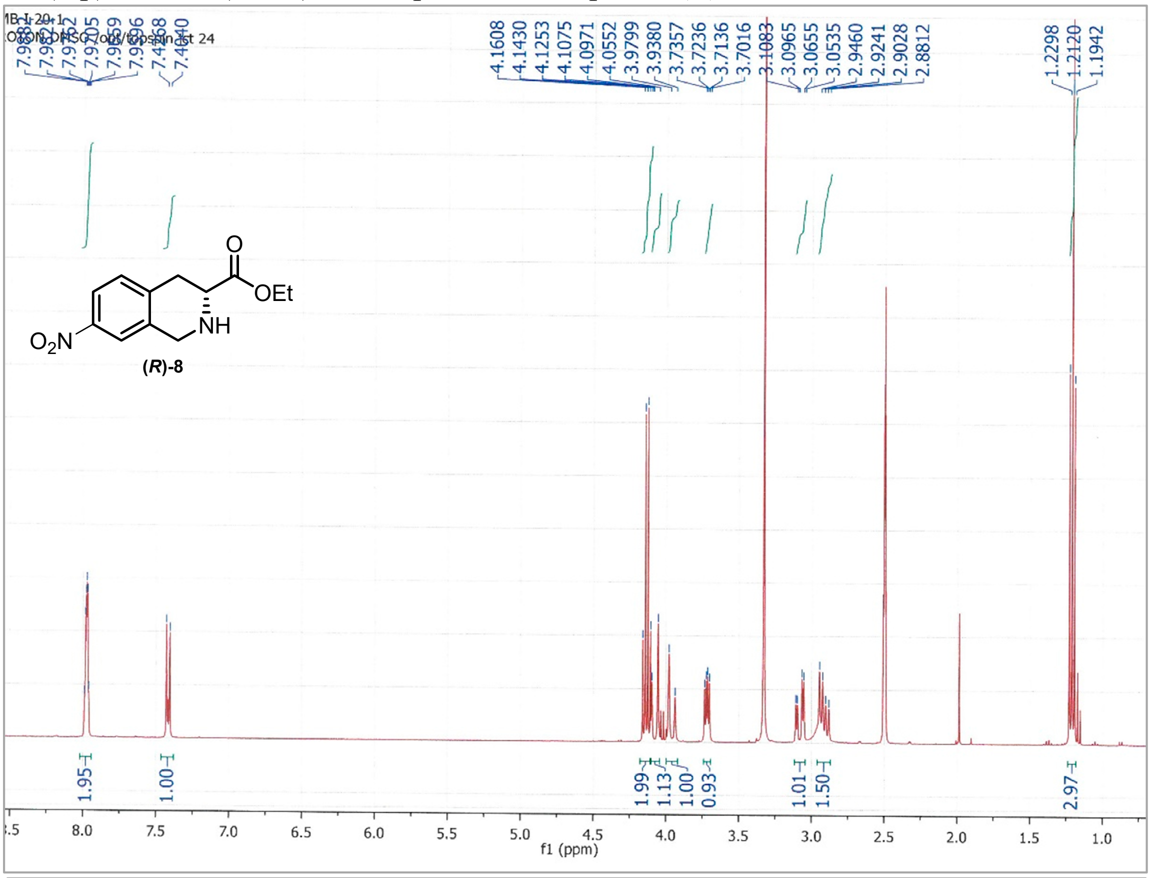

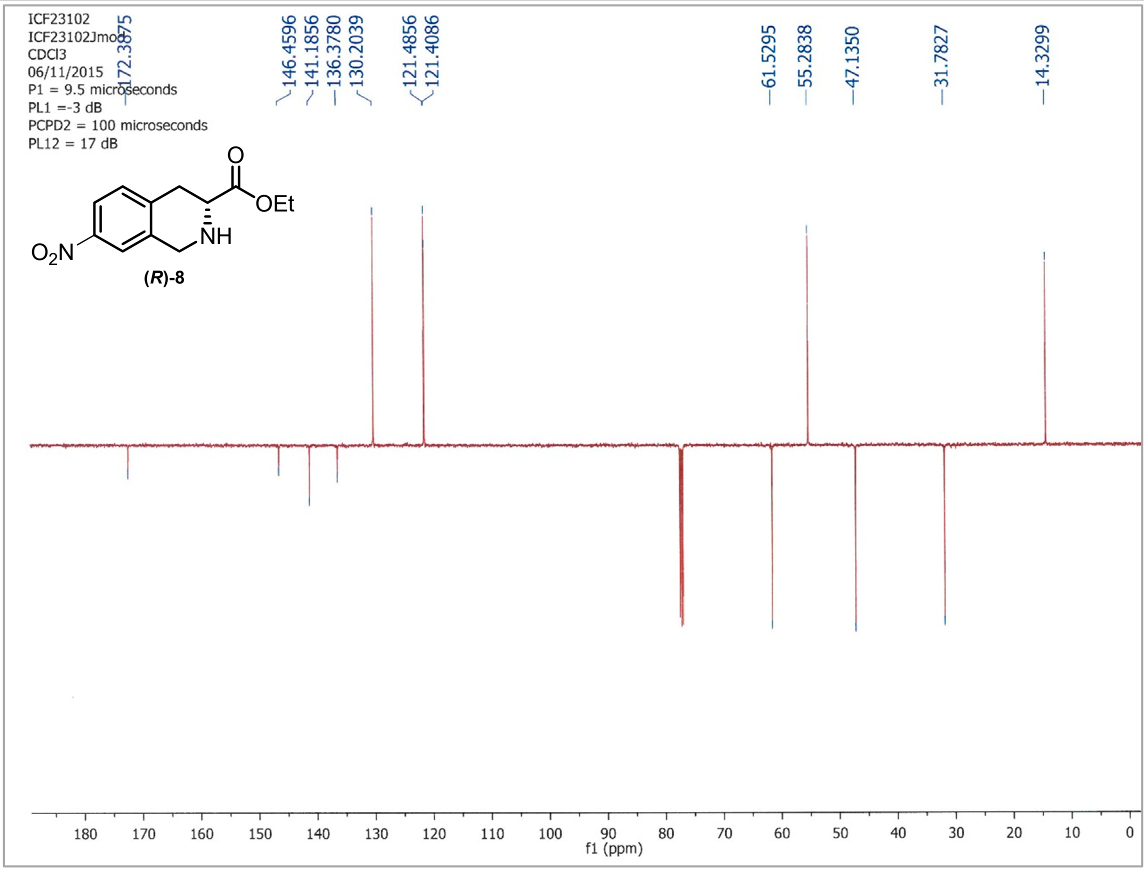

^{1}H (top) and ^{13}C (down) NMR spectra of compound **(*S*)-9** in DMSO-d_6

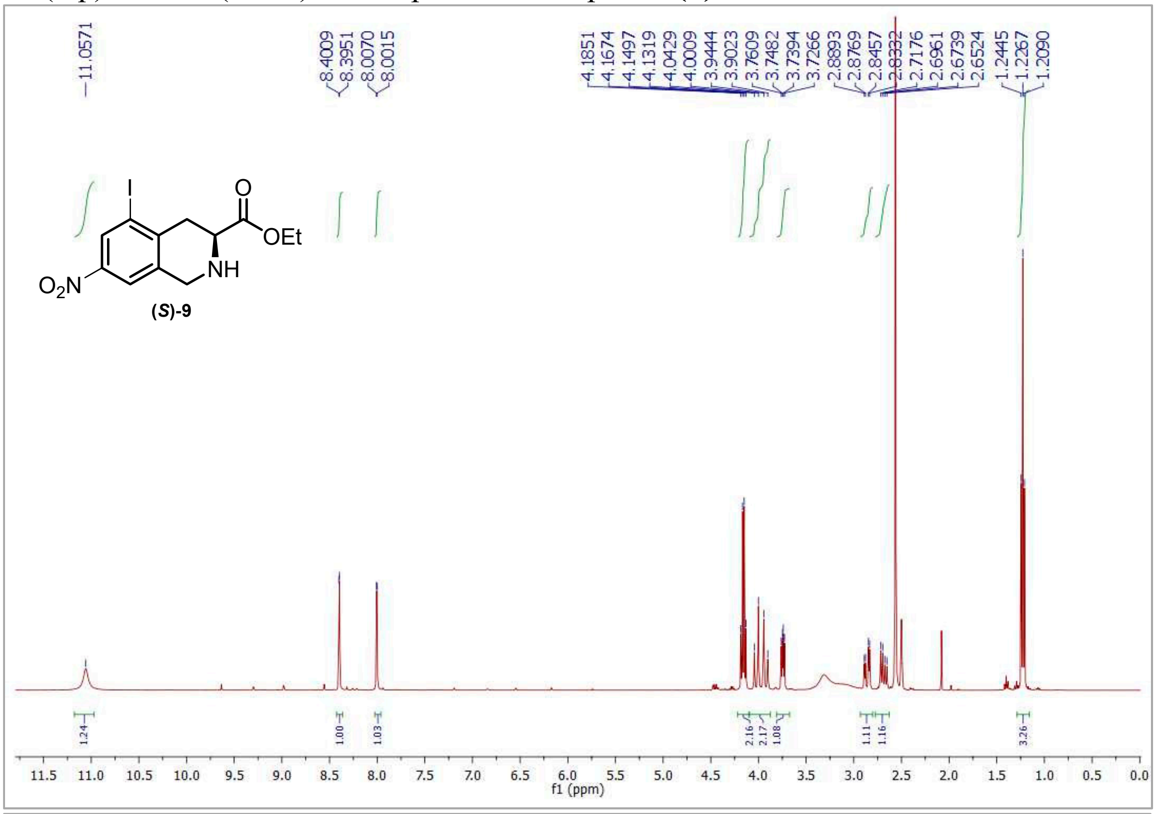

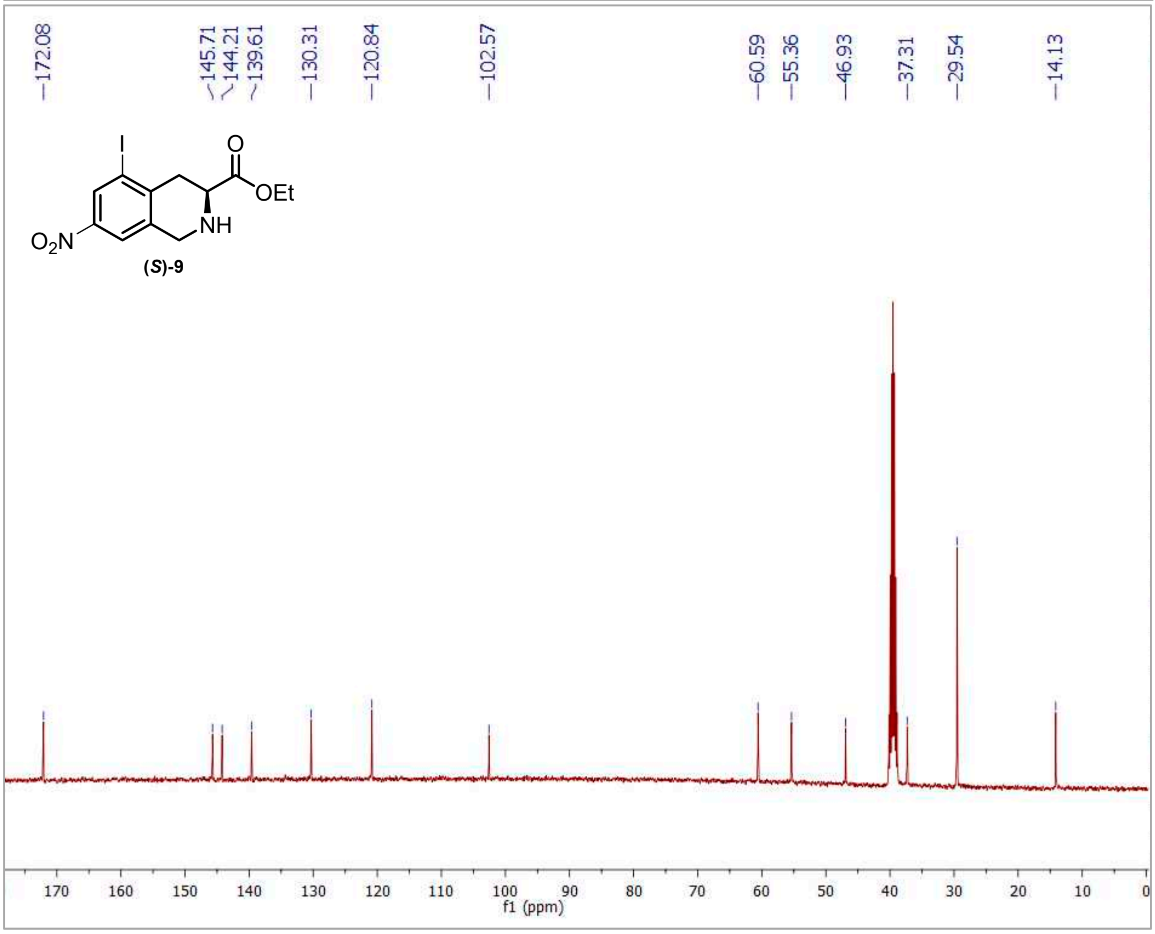

^{1}H (top) and ^{13}C (down) NMR spectra of compound **(*R*)-9** in $CDCl_3$

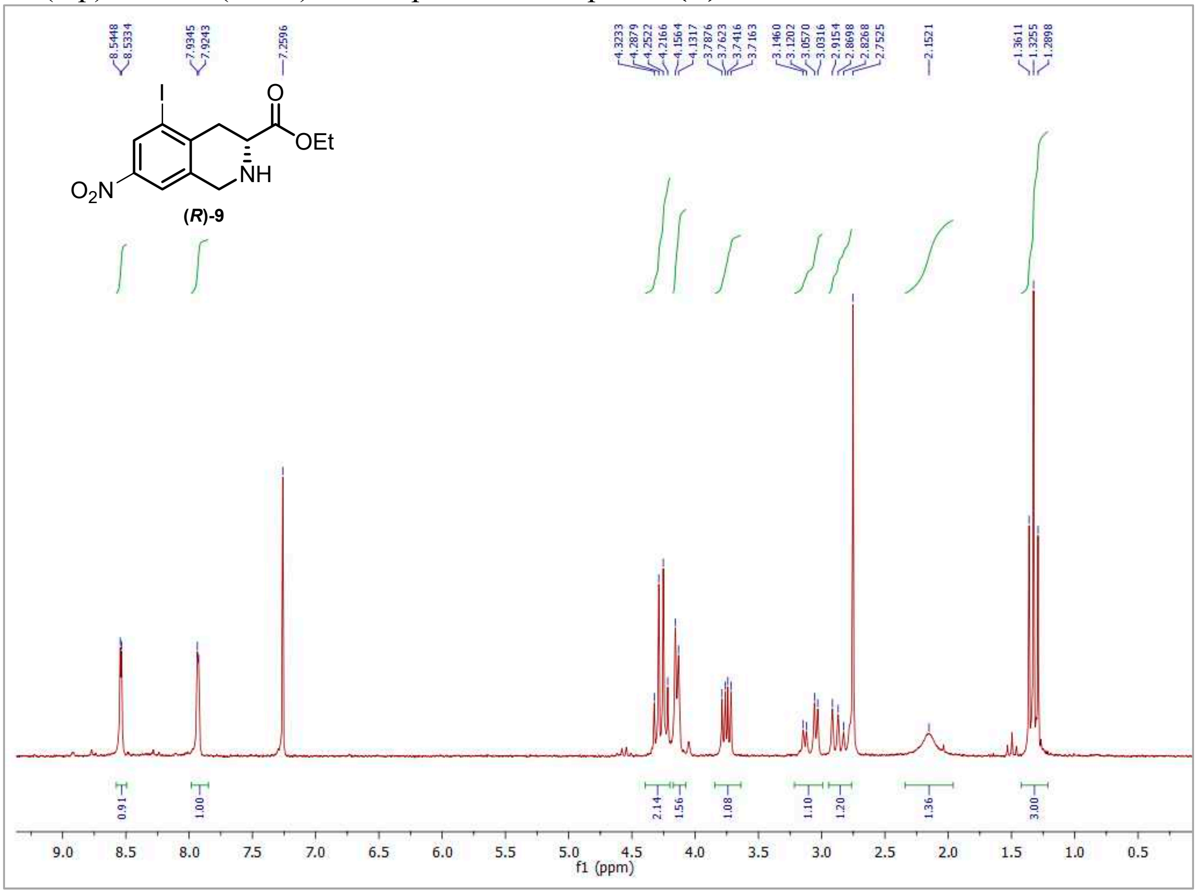

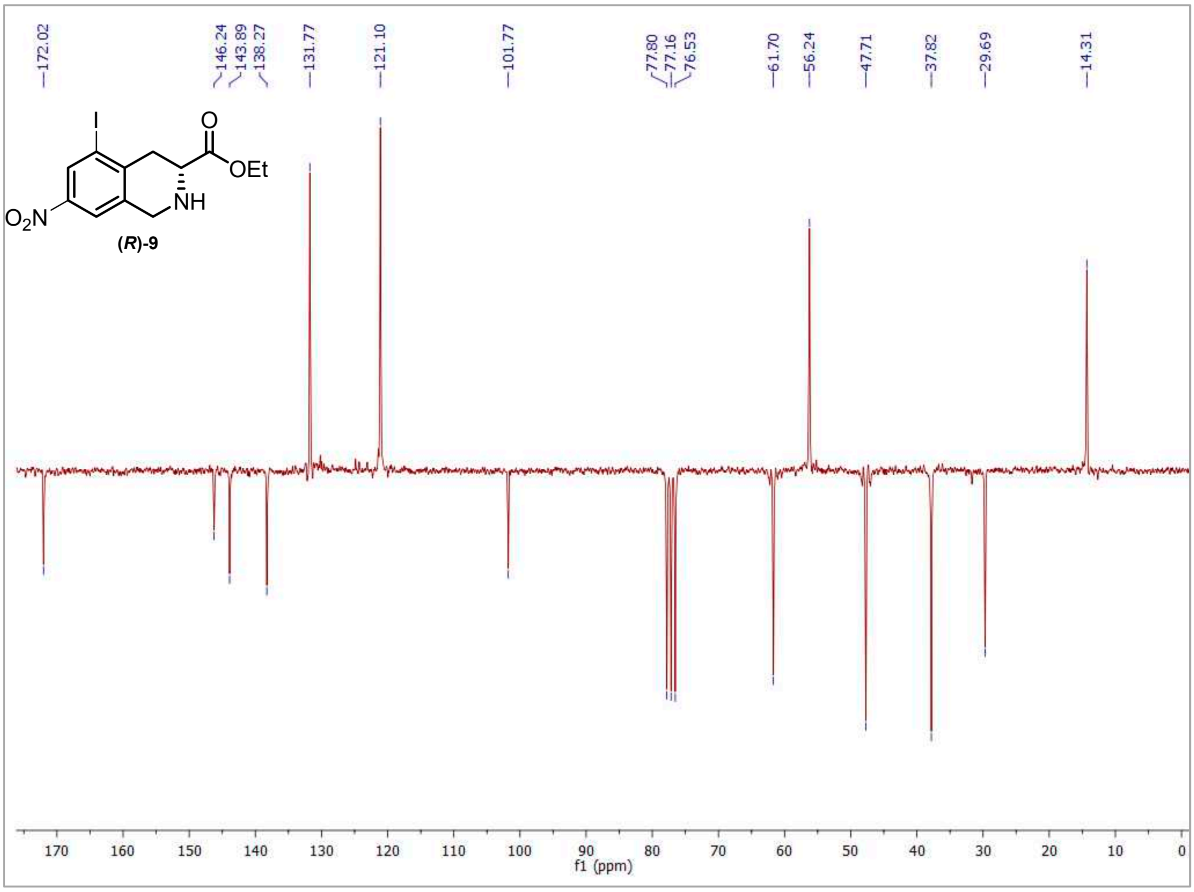

^{1}H (top) and ^{13}C (down) NMR spectra of compound ***(S)*-10** in $CDCl_3$

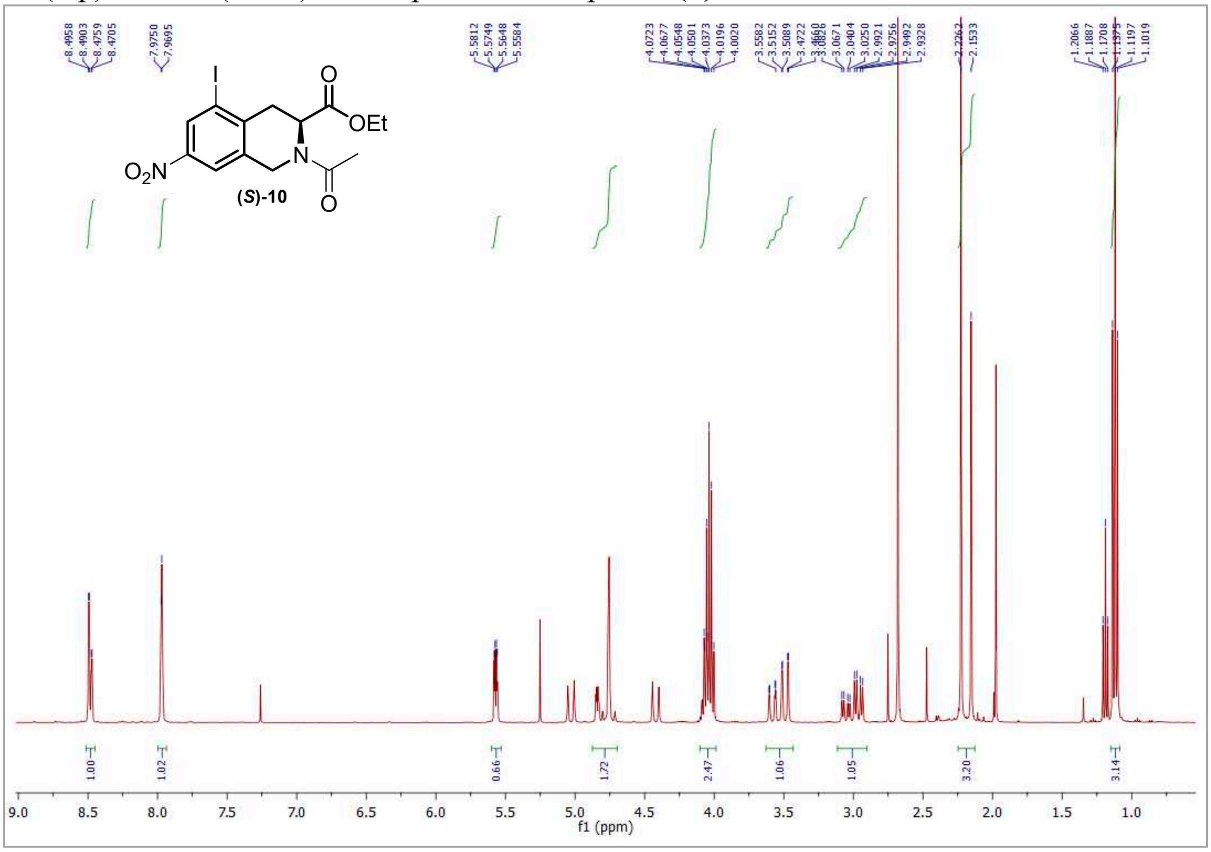

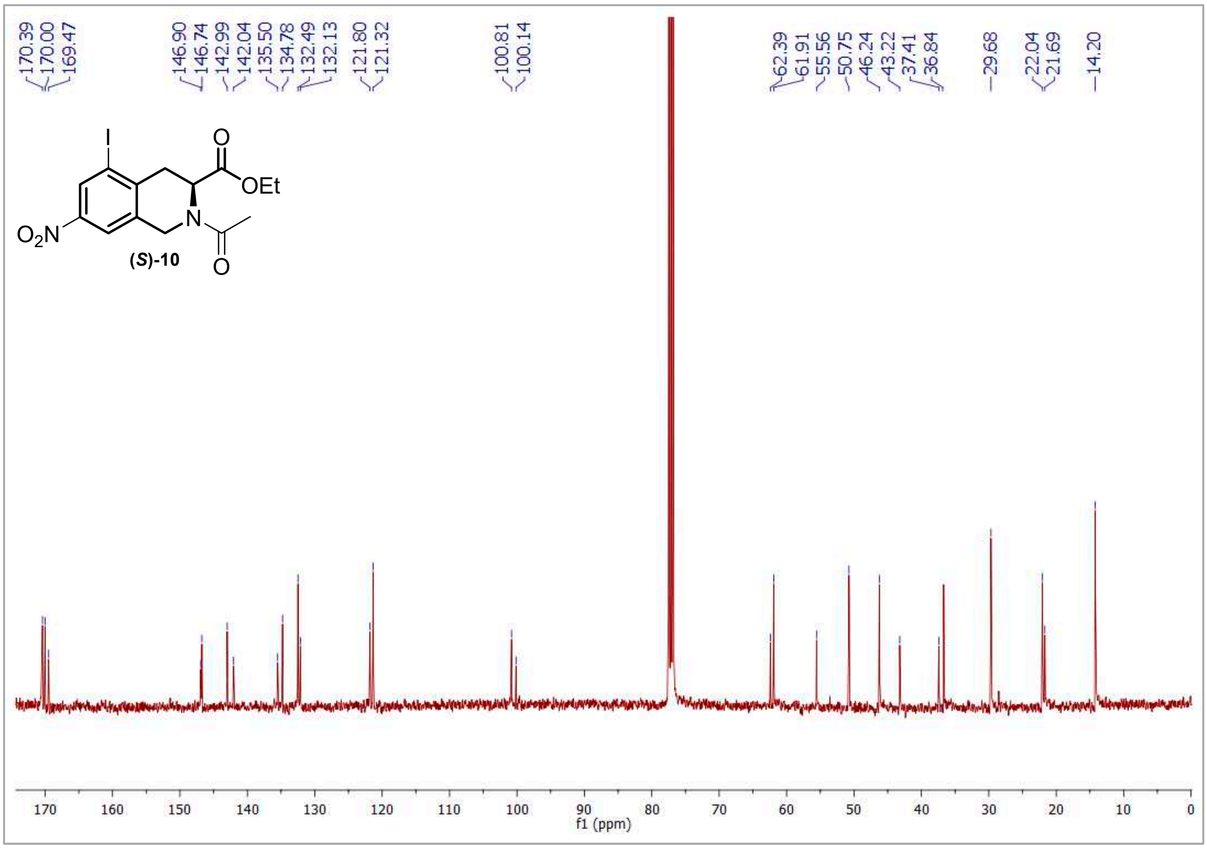

^{1}H (top) and ^{13}C (down) NMR spectra of compound **(*R*)-10** in $CDCl_3$

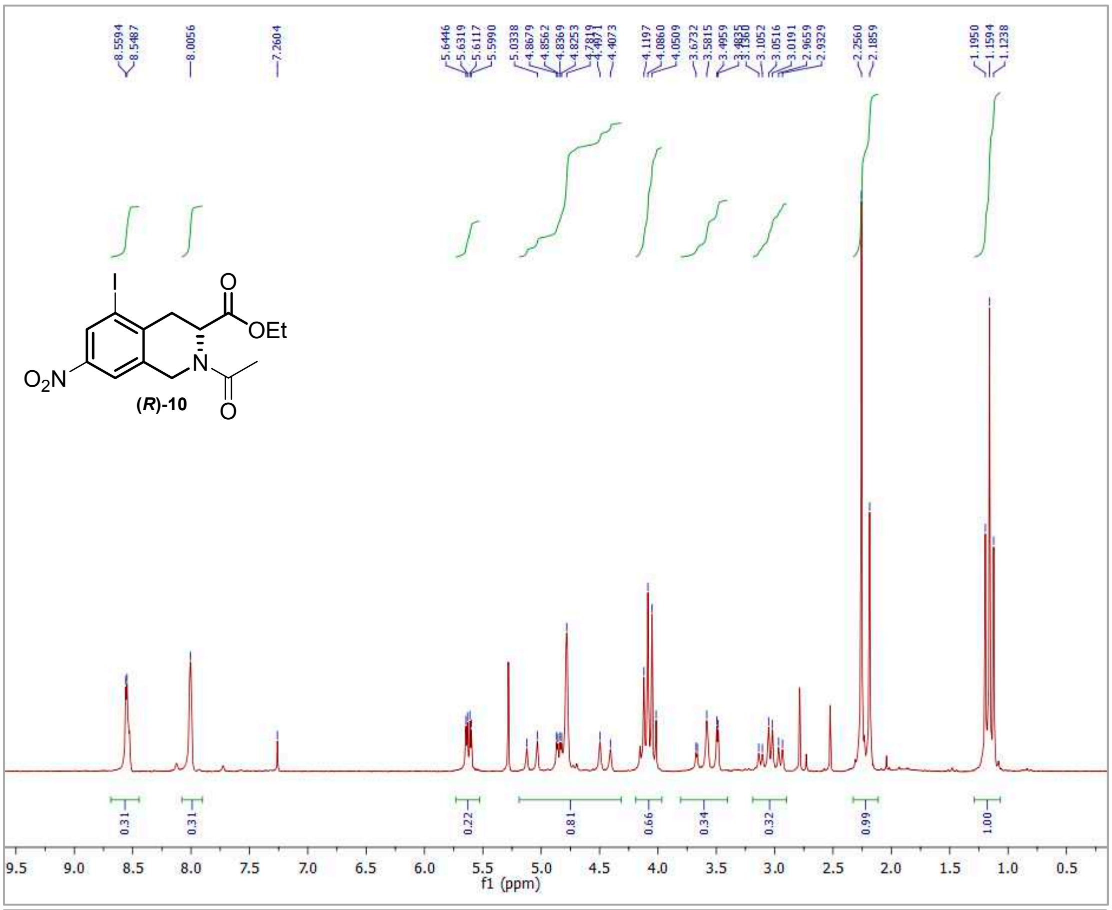

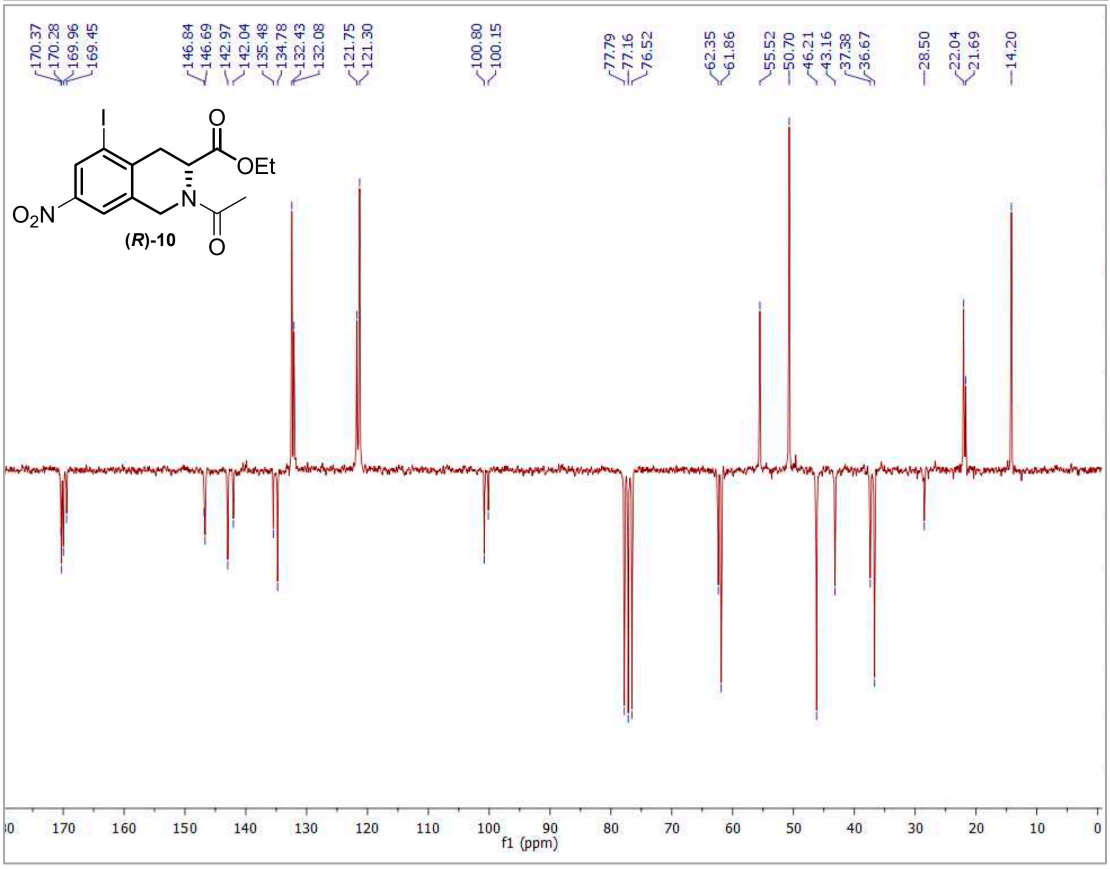

^{1}H (top) and ^{13}C (down) NMR spectra of compound ***(S)*-11** in DMSO-d_6

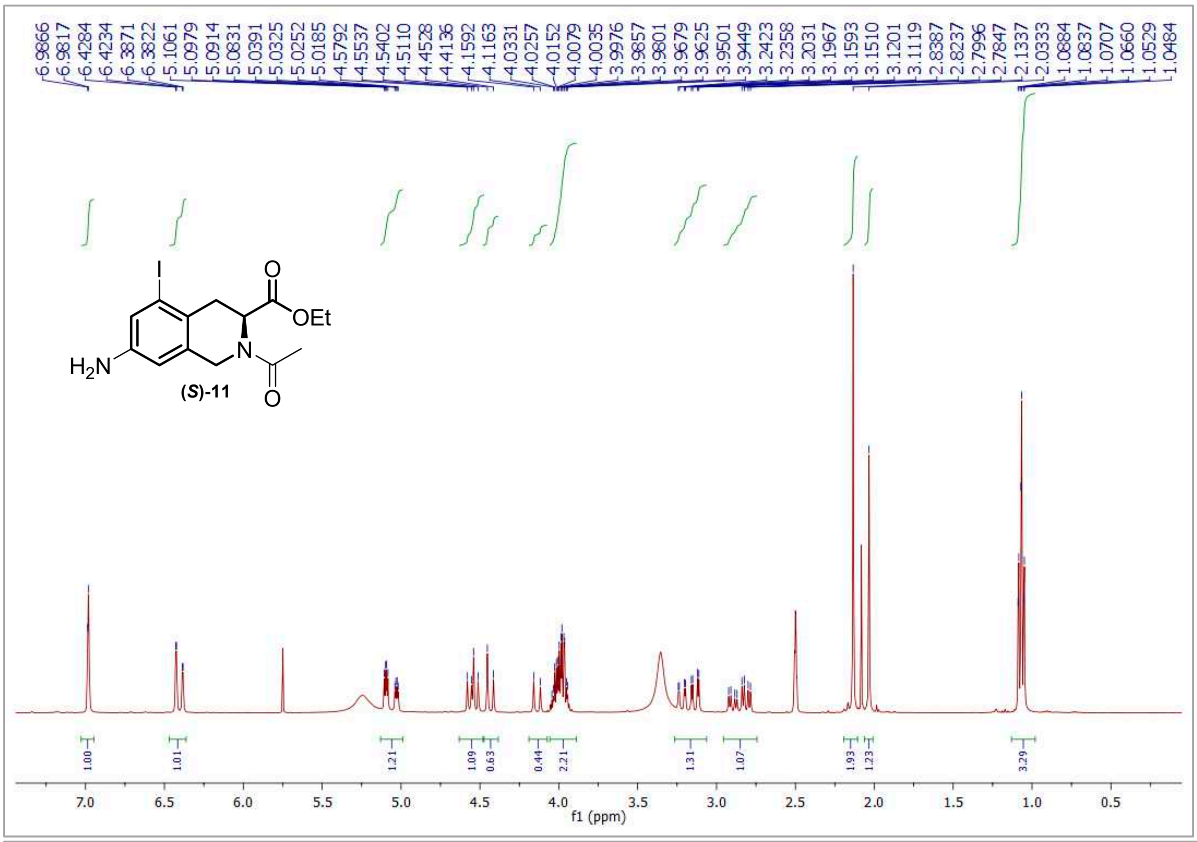

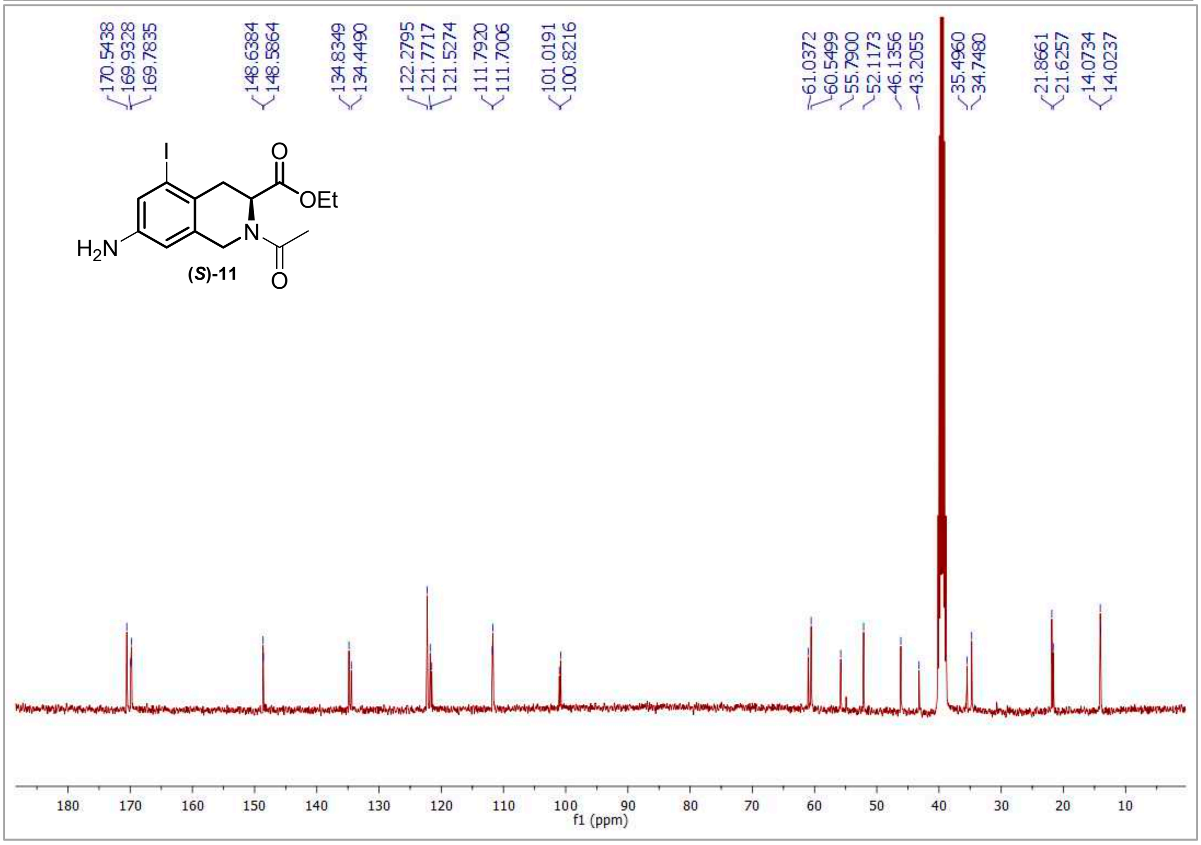

^{1}H (top) and ^{13}C (down) NMR spectra of compound **(*R*)-11** in $CDCl_3$

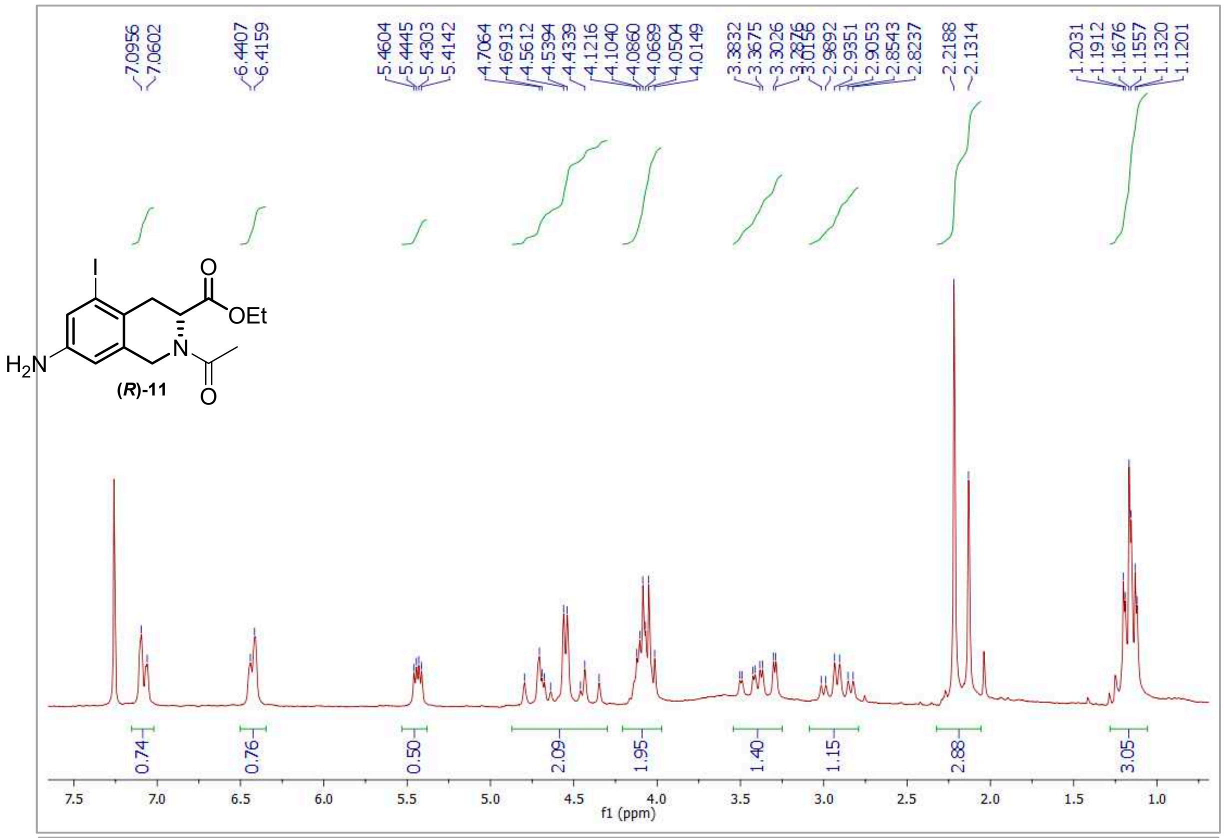

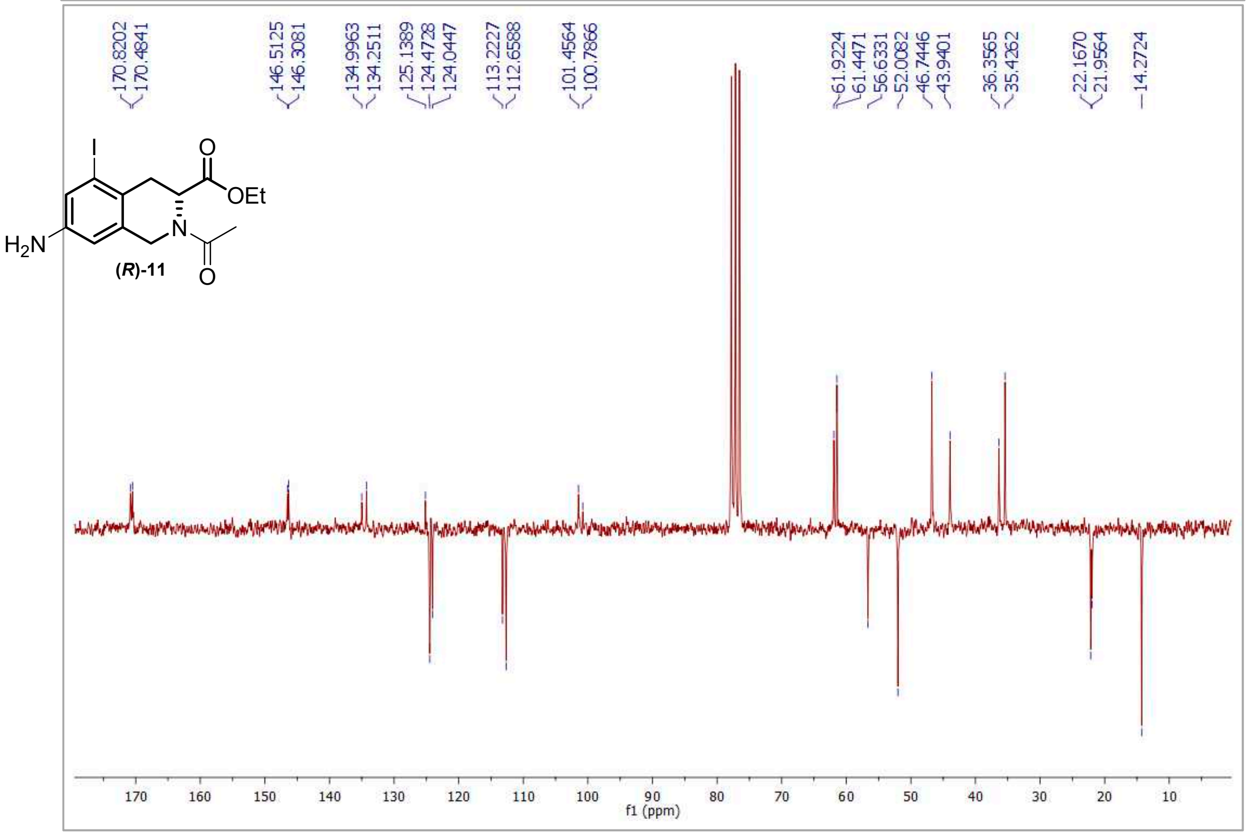

^{1}H (top) and ^{13}C (down) NMR spectra of compound **(*S*)-12** in $CDCl_3$

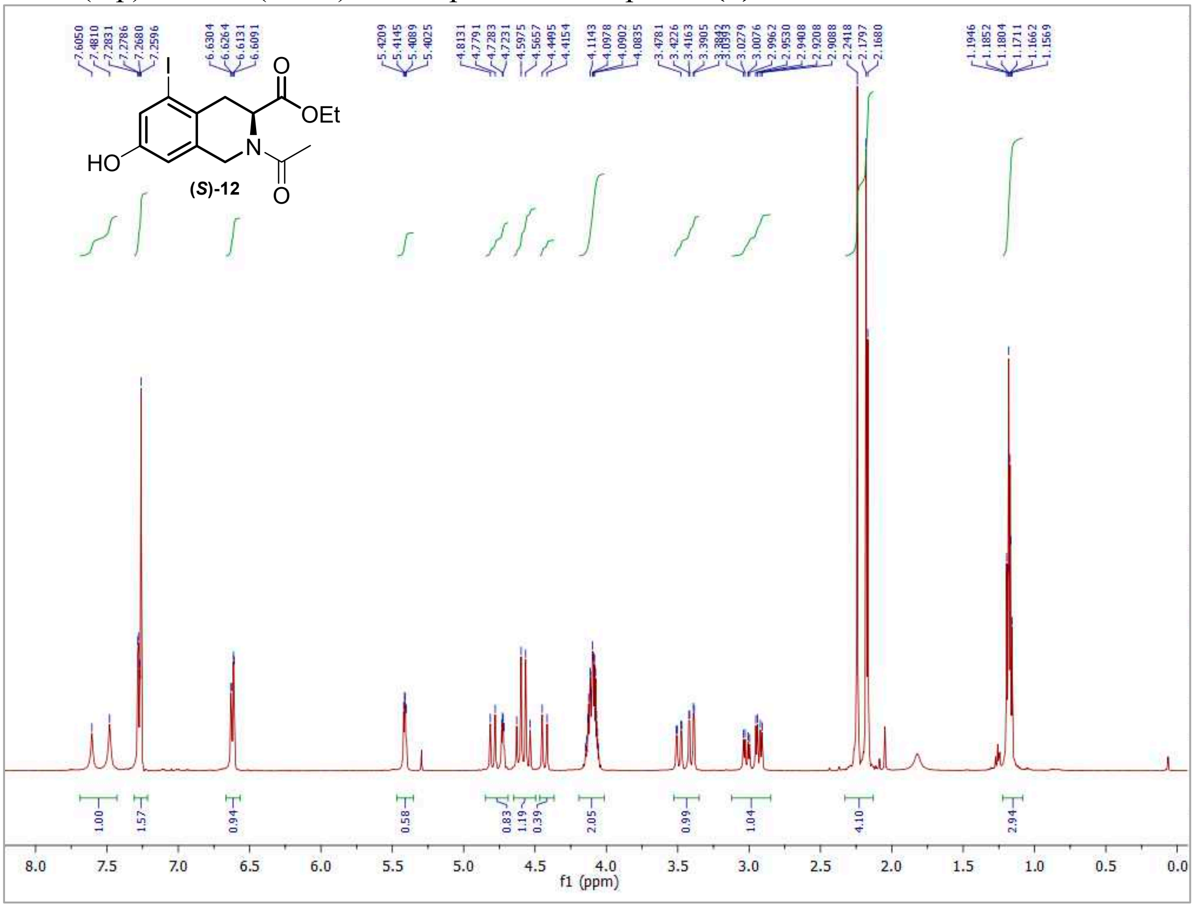

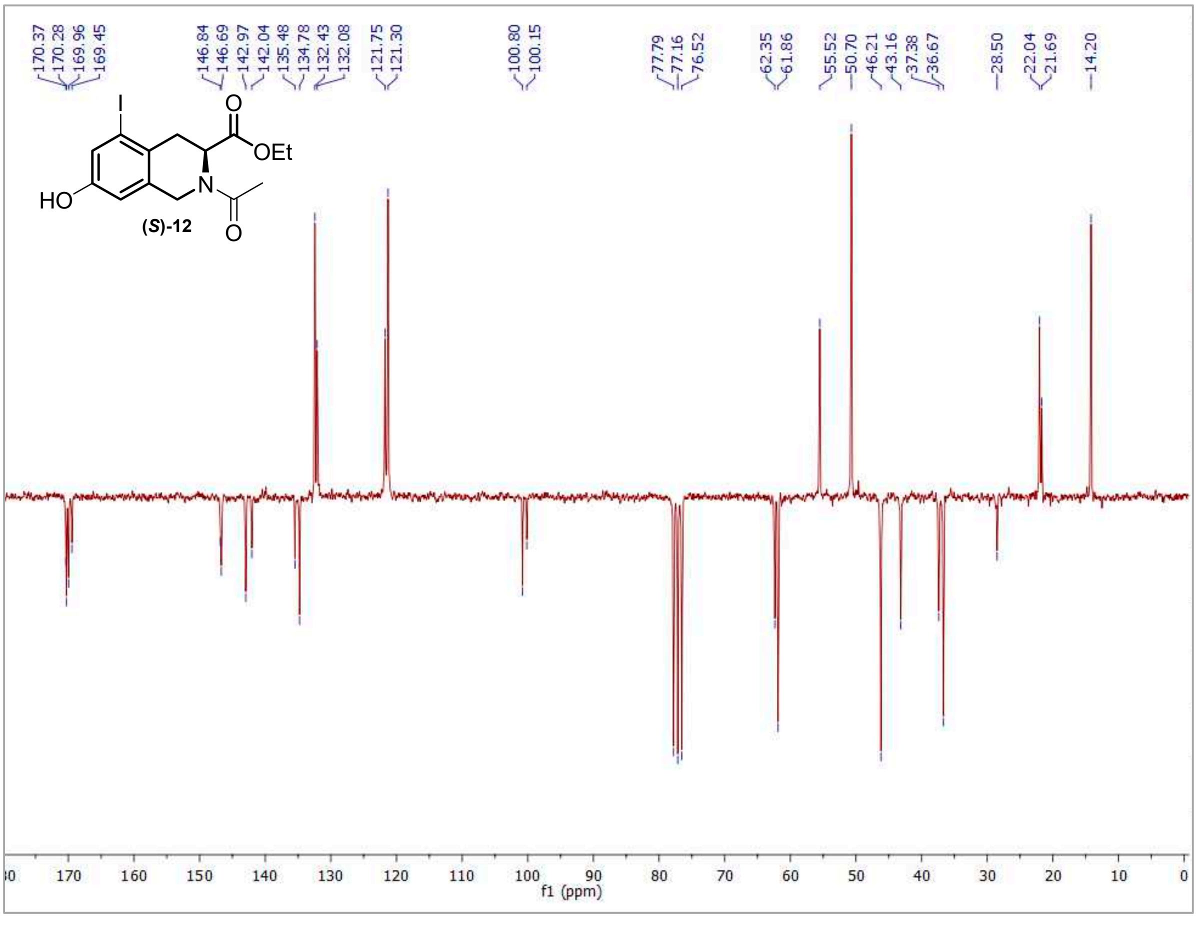

^{1}H (top) and ^{13}C (down) NMR spectra of compound ***(R)*-12** in $CDCl_3$

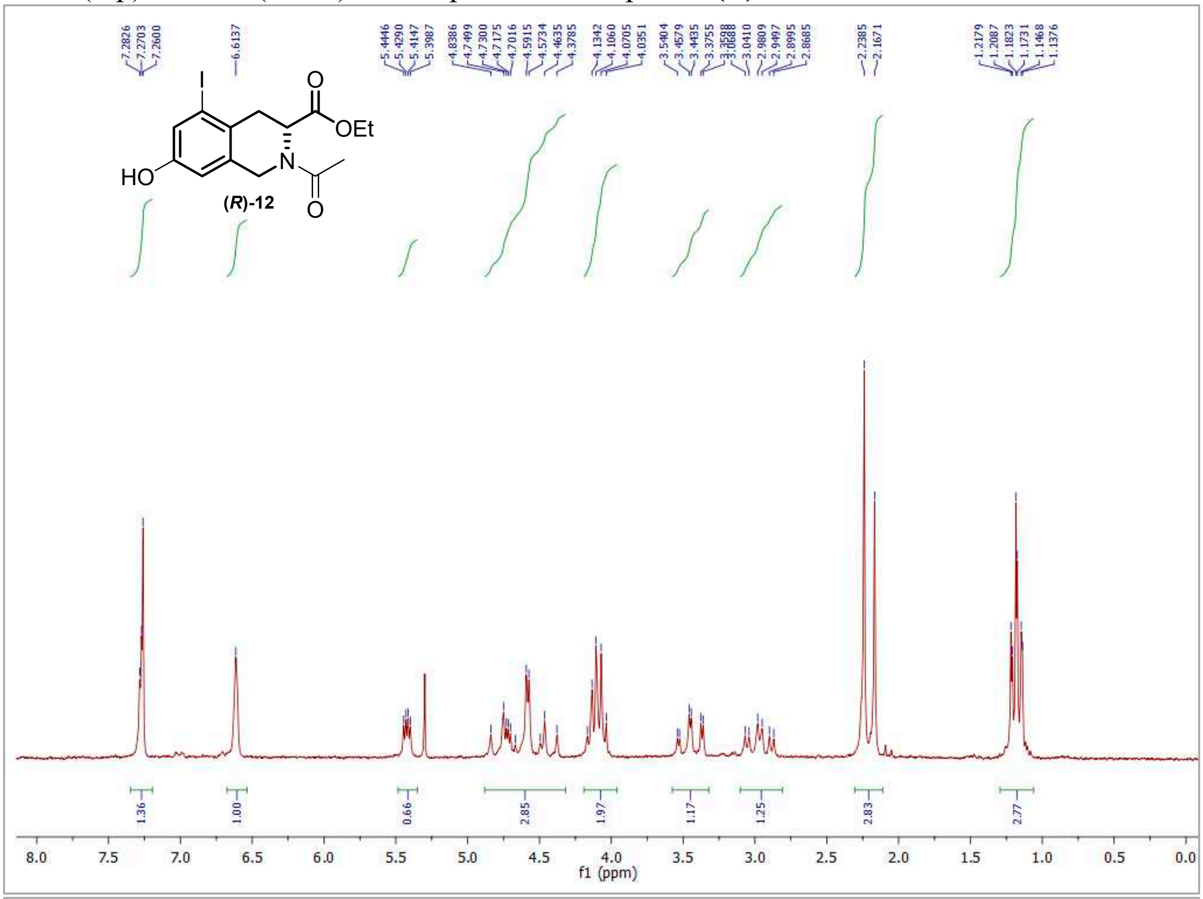

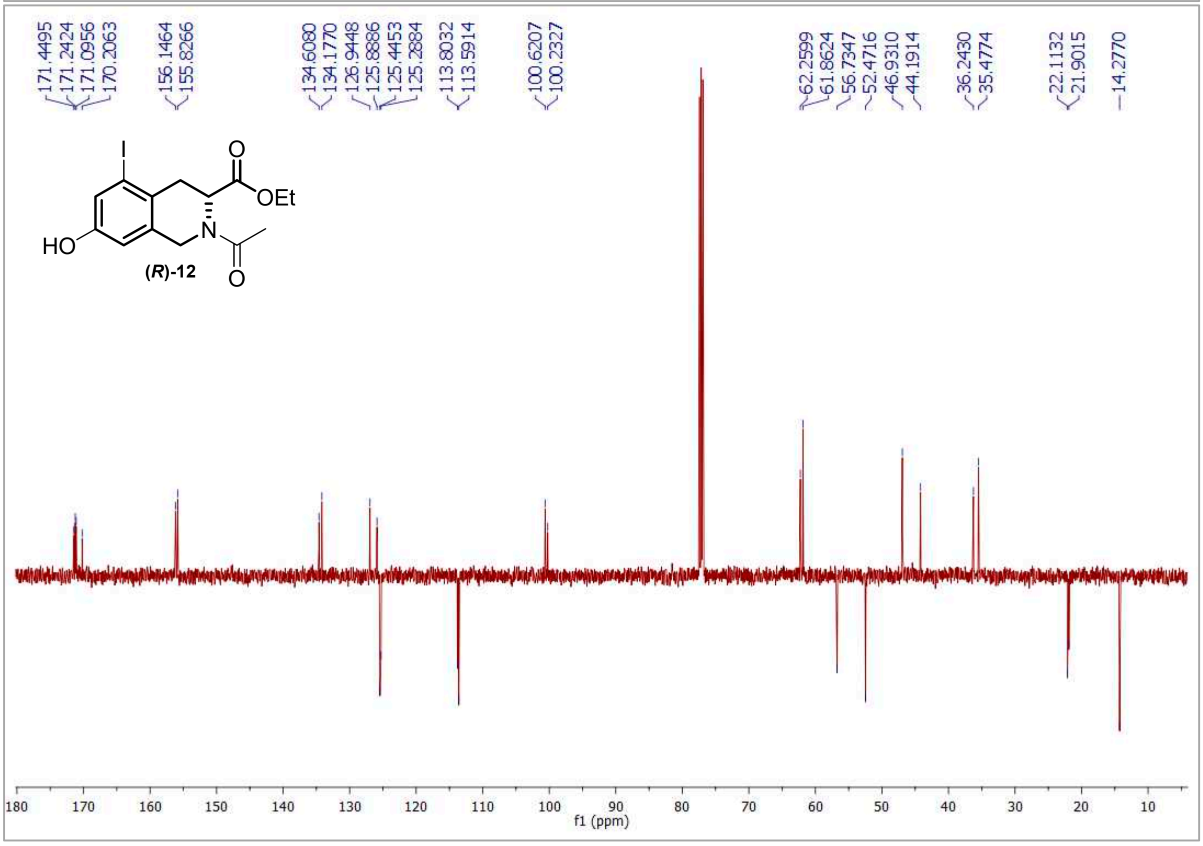

^{1}H (top) and ^{13}C (down) NMR spectra of compound **(*S*)-13** in CD_3OD

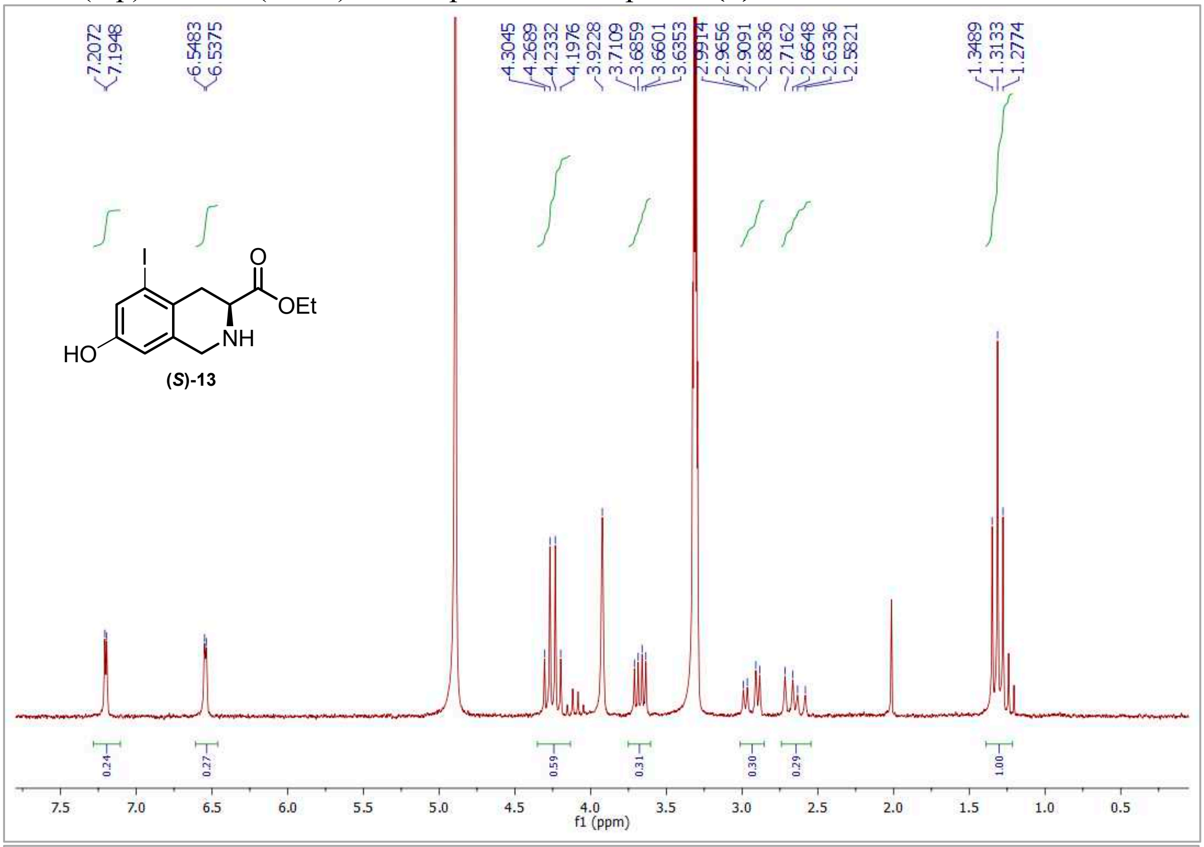

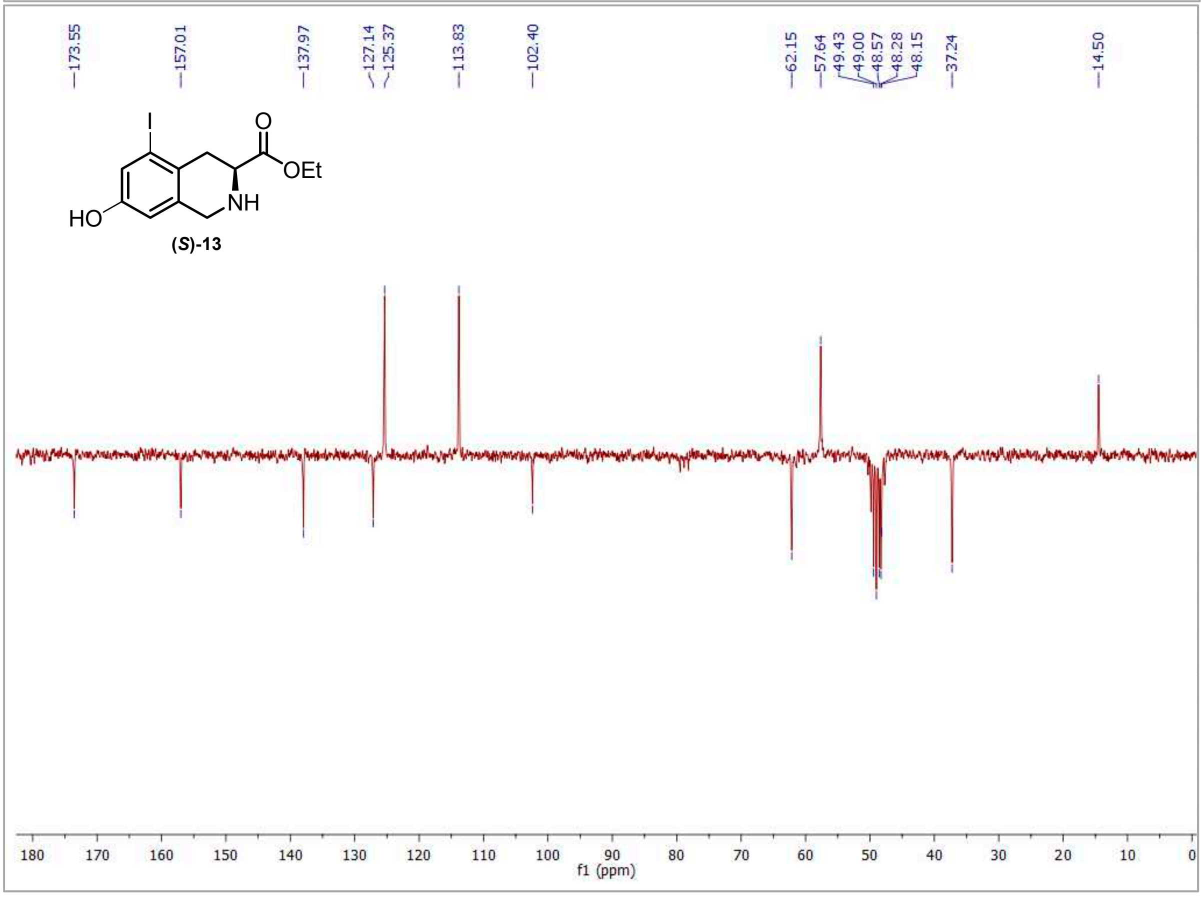

^{1}H (top) and ^{13}C (down) NMR spectra of compound **(*R*)-13** in $CDCl_3$ (^{1}H) or CD_3OD (^{13}C)

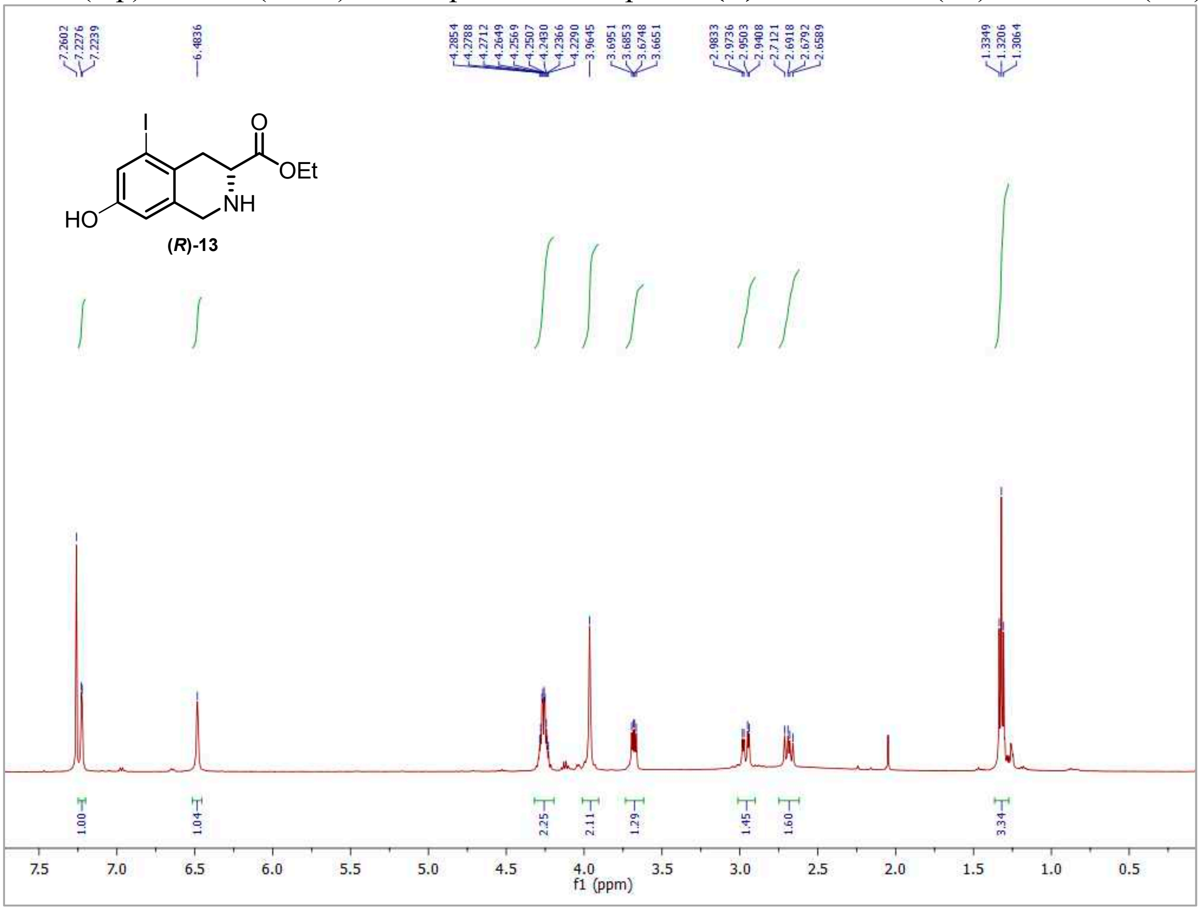

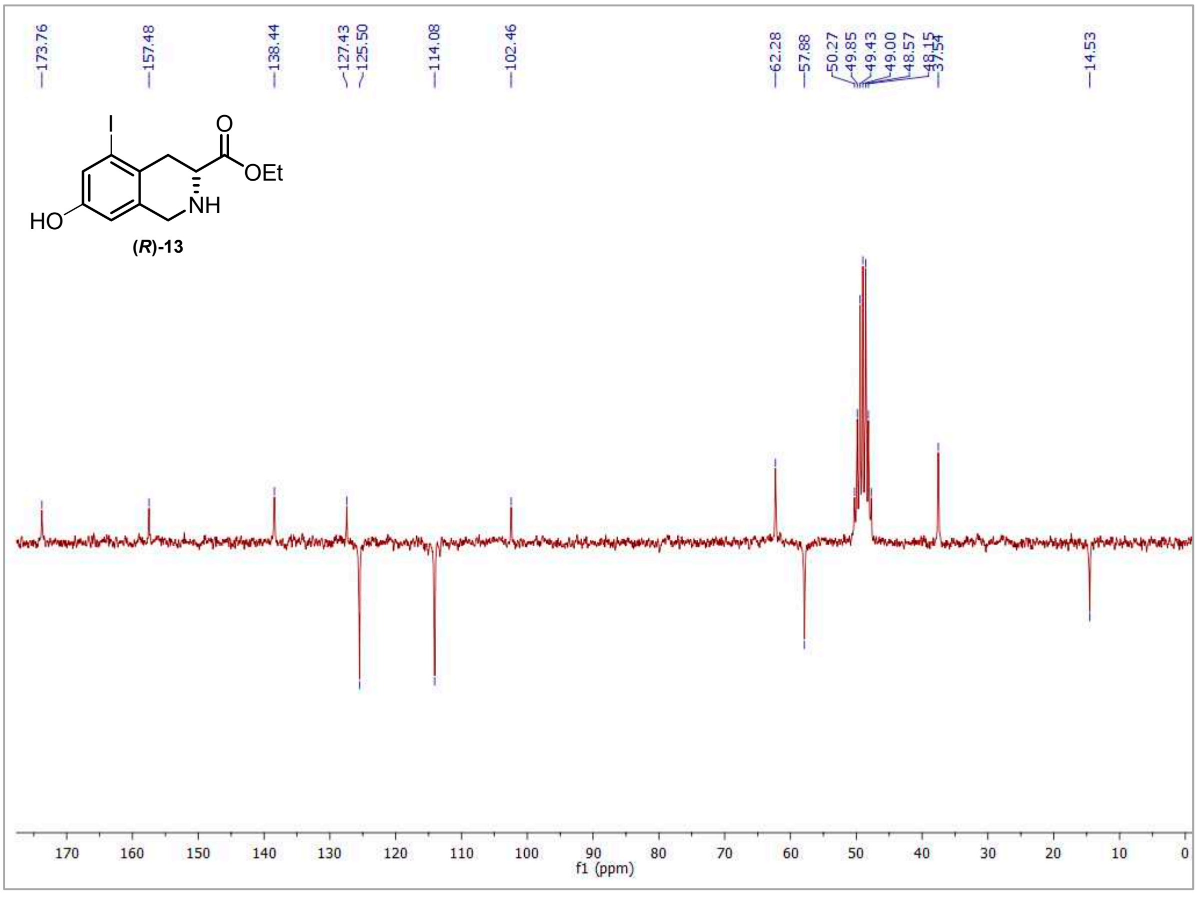

^{1}H (top) and ^{13}C (down) NMR spectra of compound **(*S*)-14** in DMSO-d_6

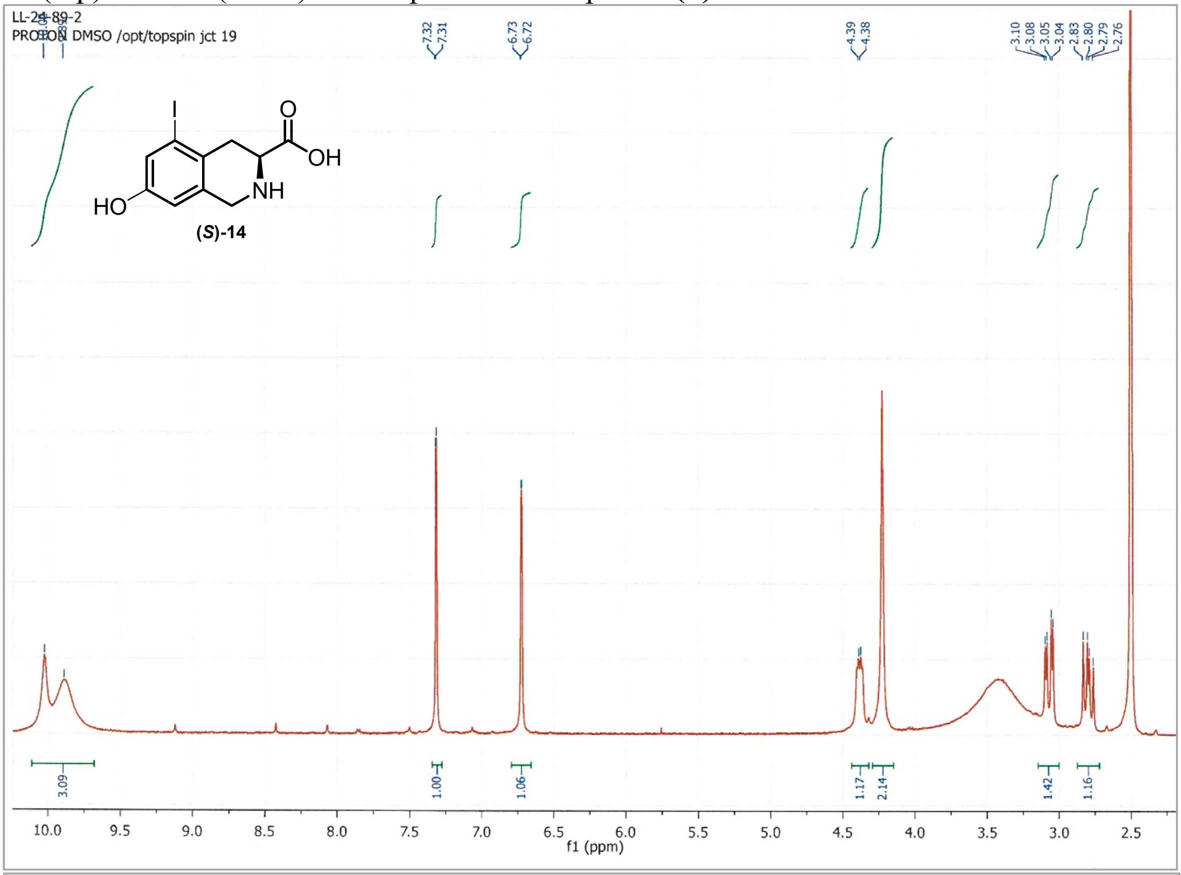

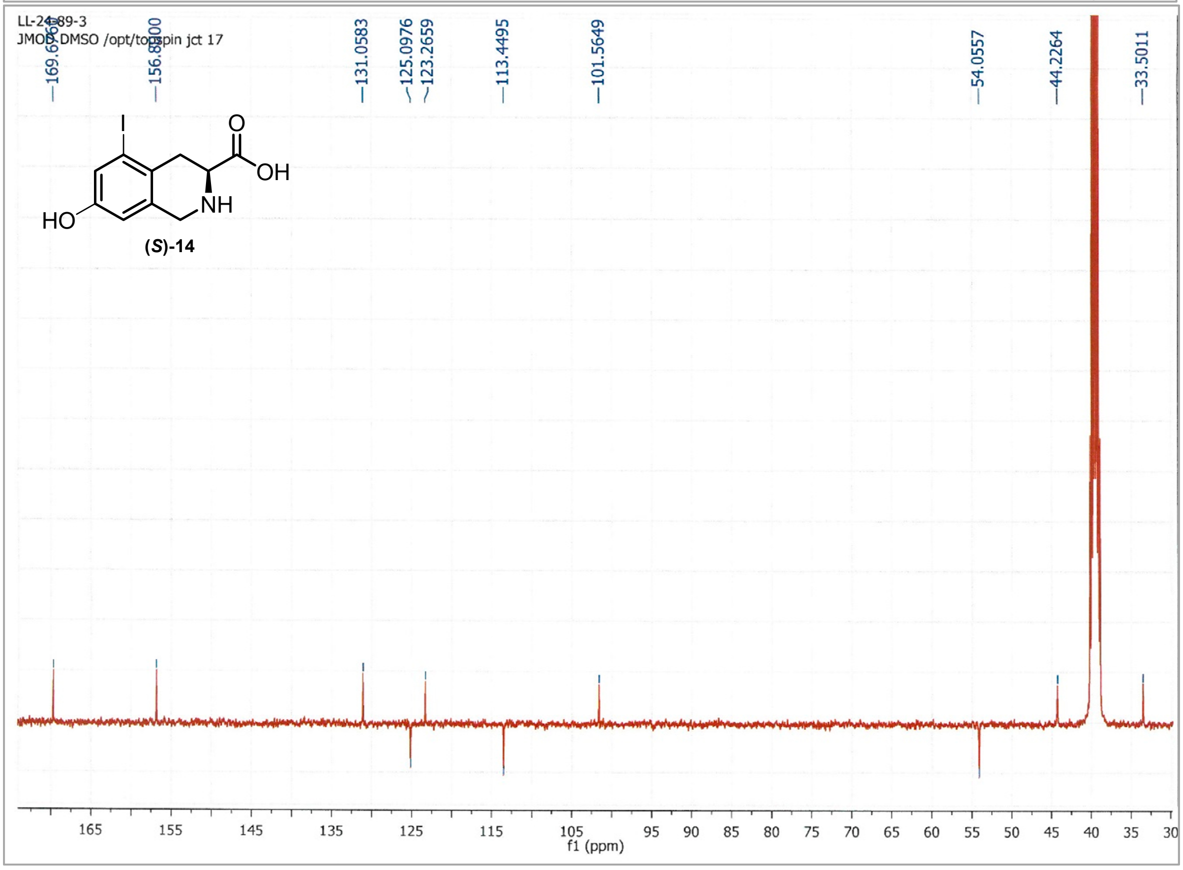

^{1}H (top) and ^{13}C (down) NMR spectra of compound ***(R)*-14** in DMSO-d_6

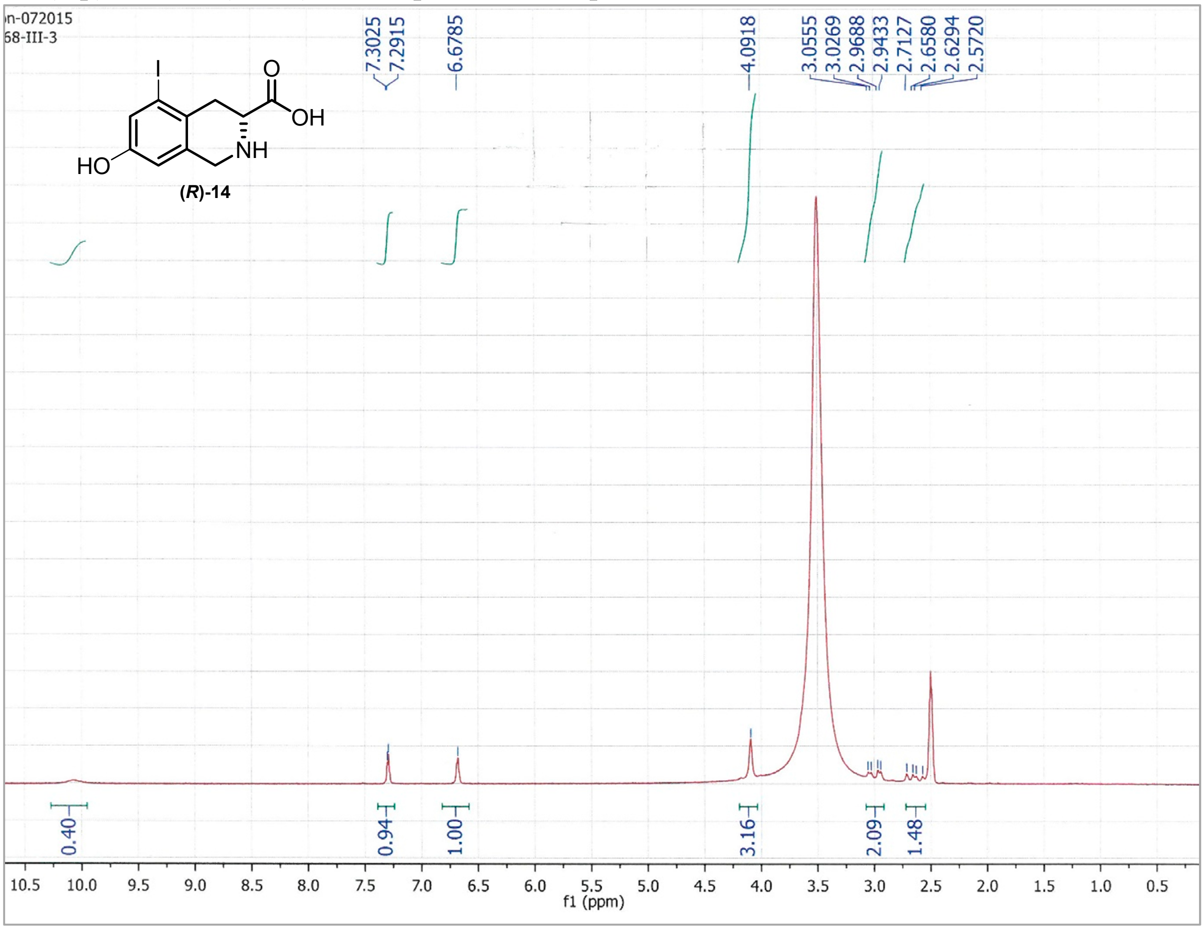

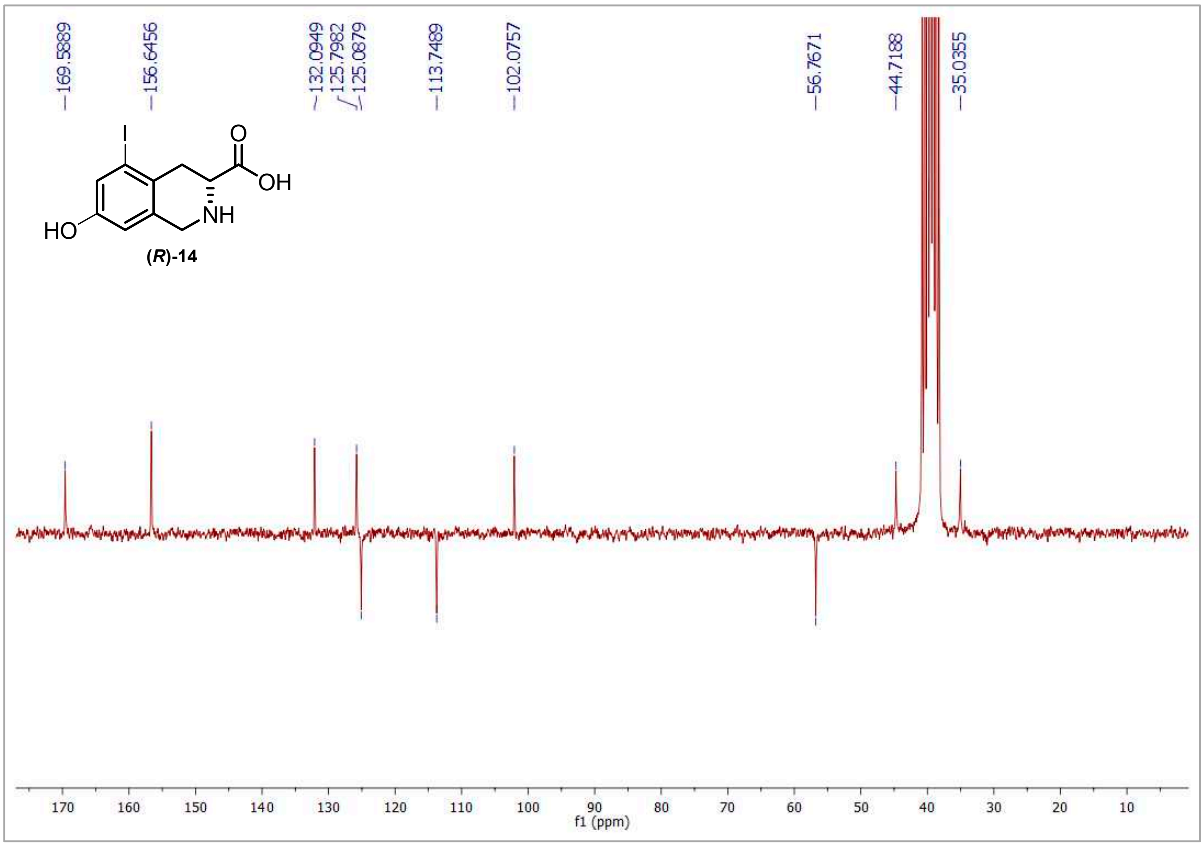

^{1}H (top) and ^{13}C (down) NMR spectra of compound **(*S*)-15** in DMSO-d_6.

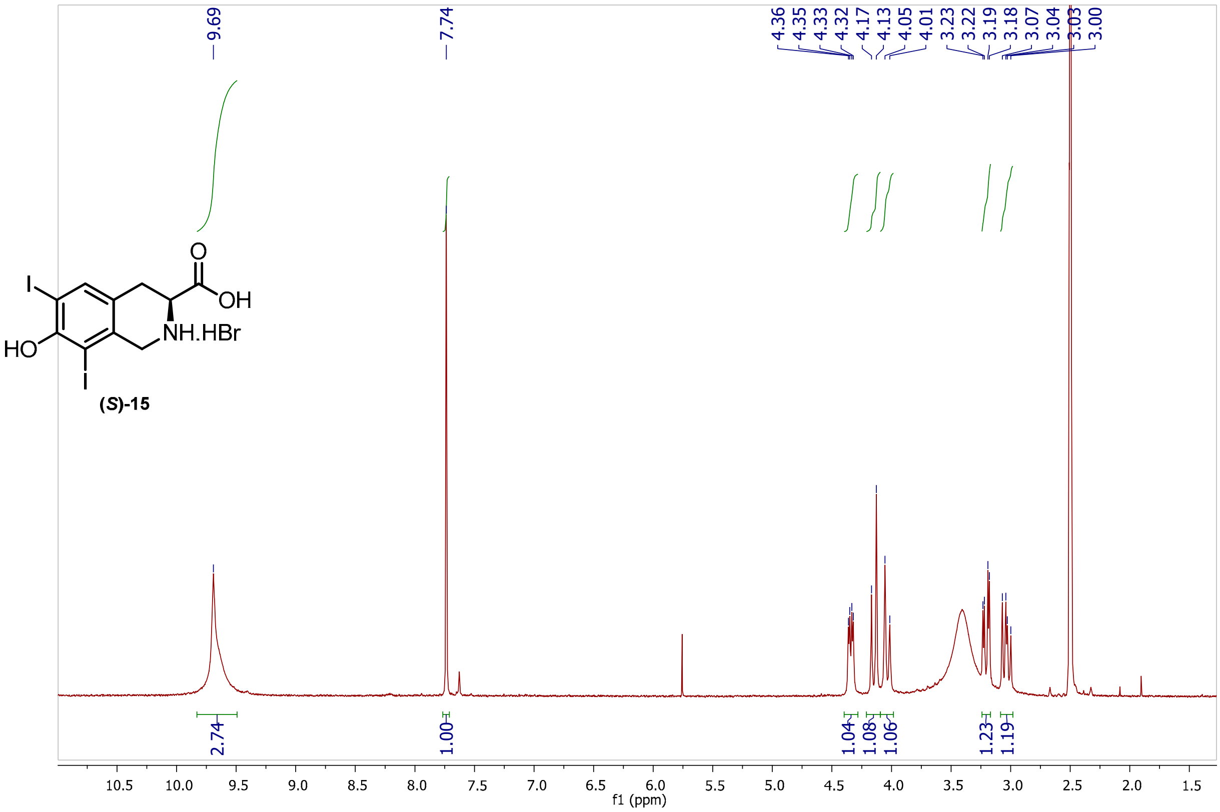

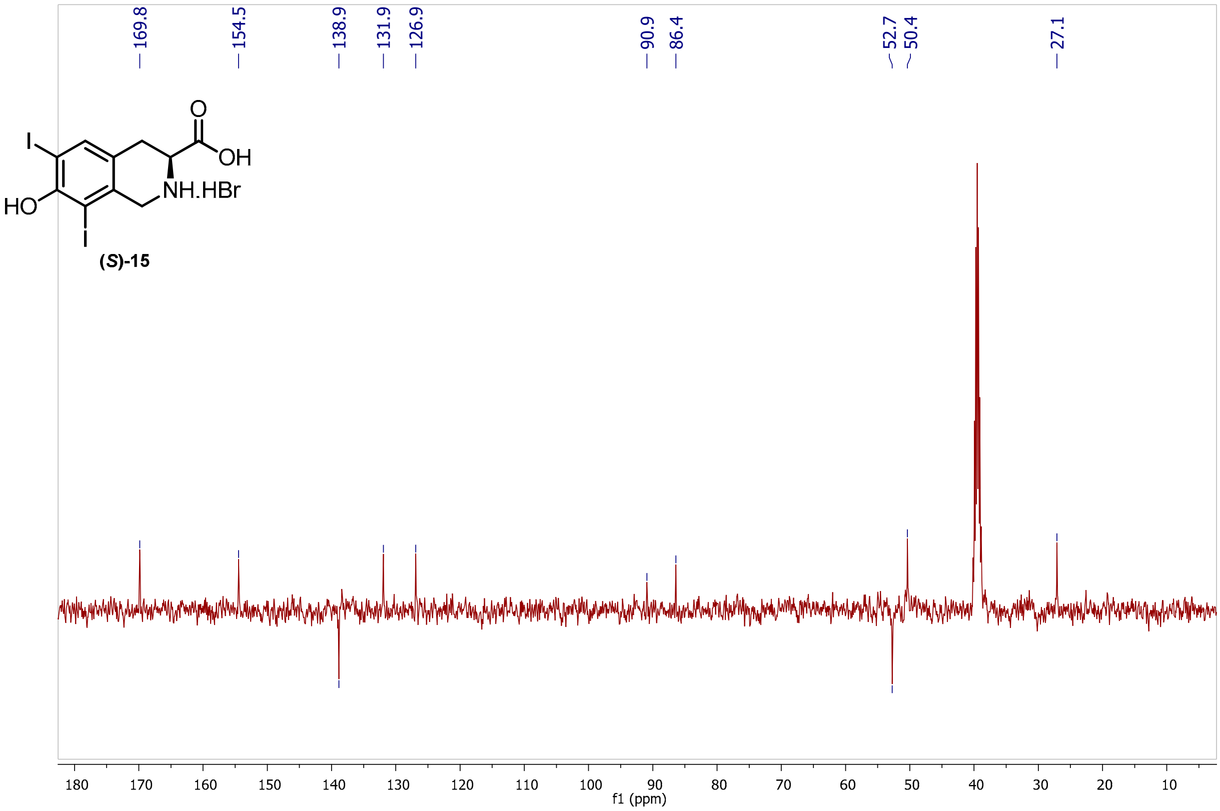

^{1}H (top) and ^{13}C (down) NMR spectra of compound **(*R*)-15** in DMSO-d_6.

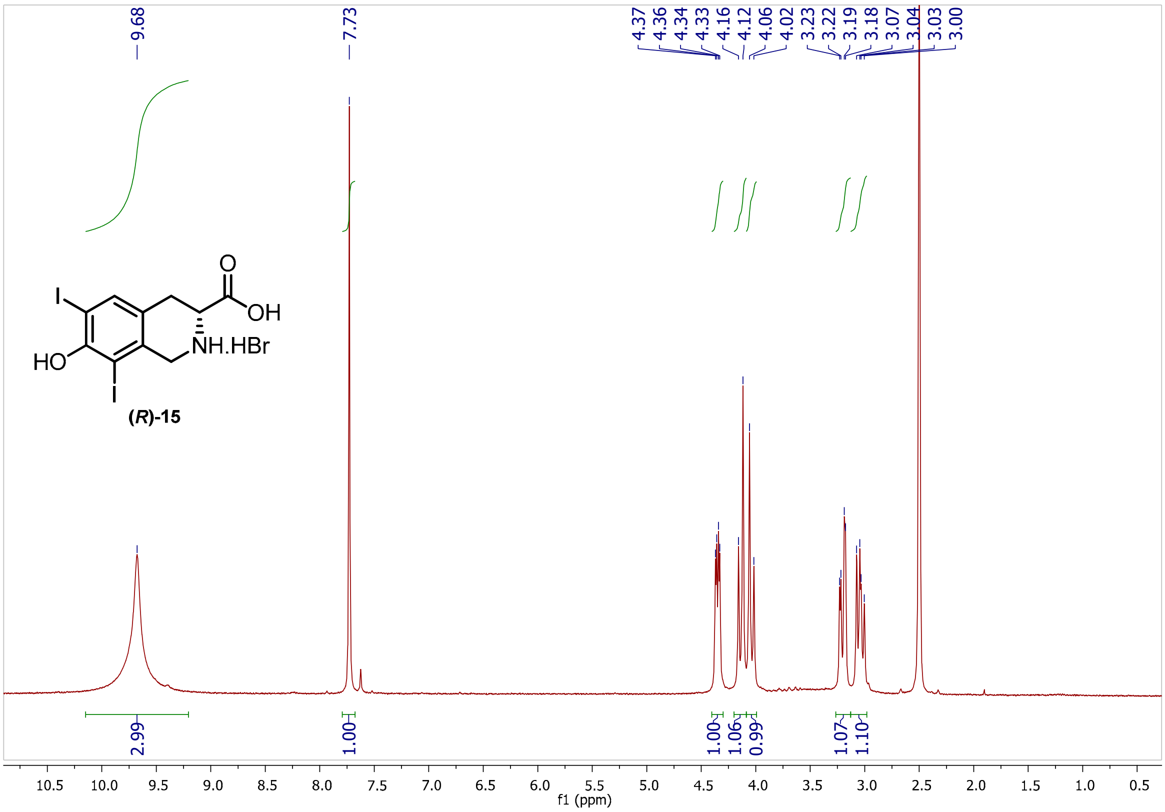

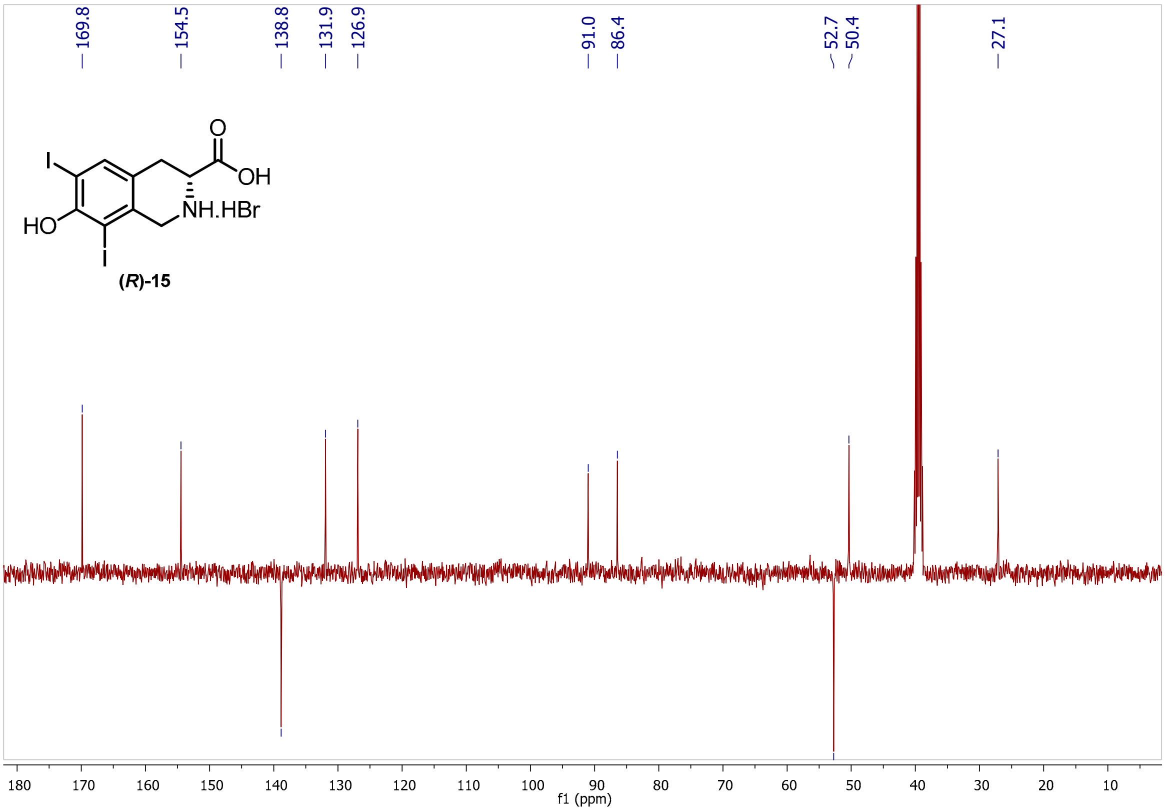

^{1}H (top) and ^{13}C (down) NMR spectra of compound **(*S*)-16** in DMSO-d_6.

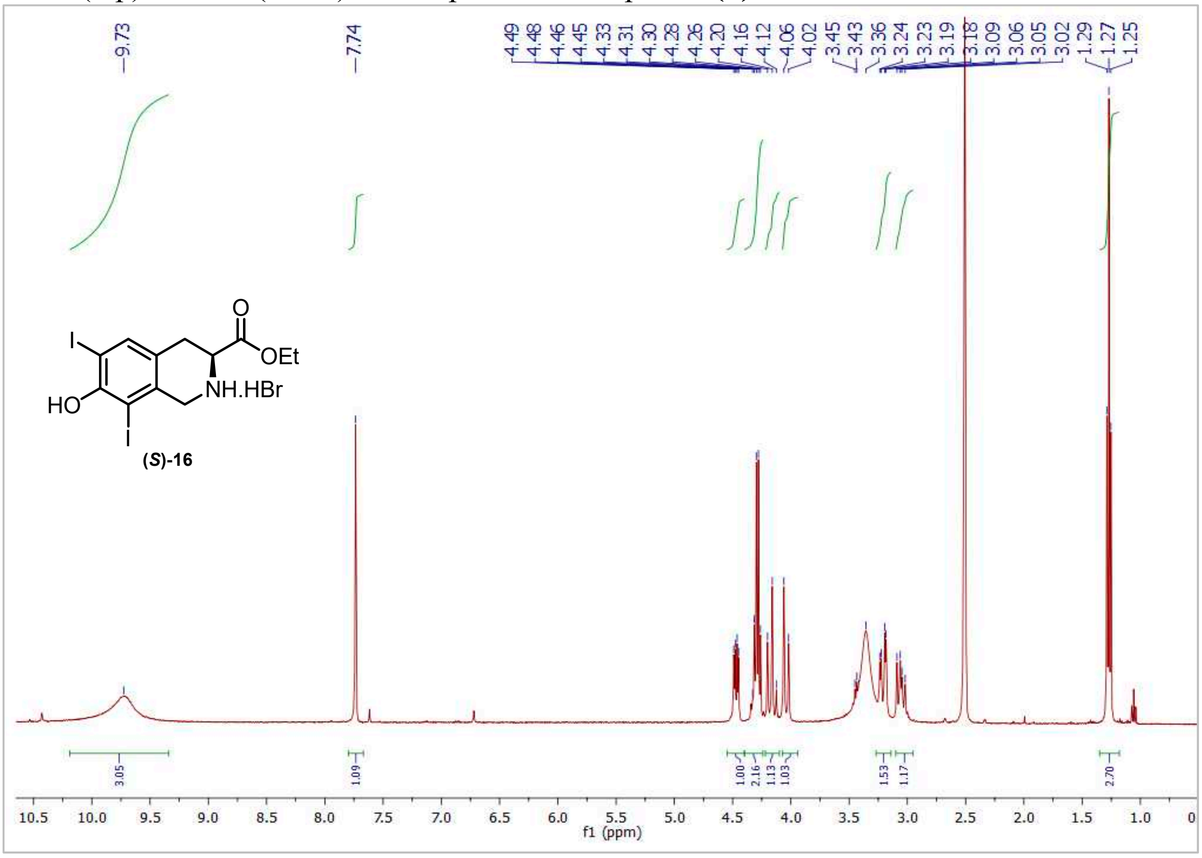

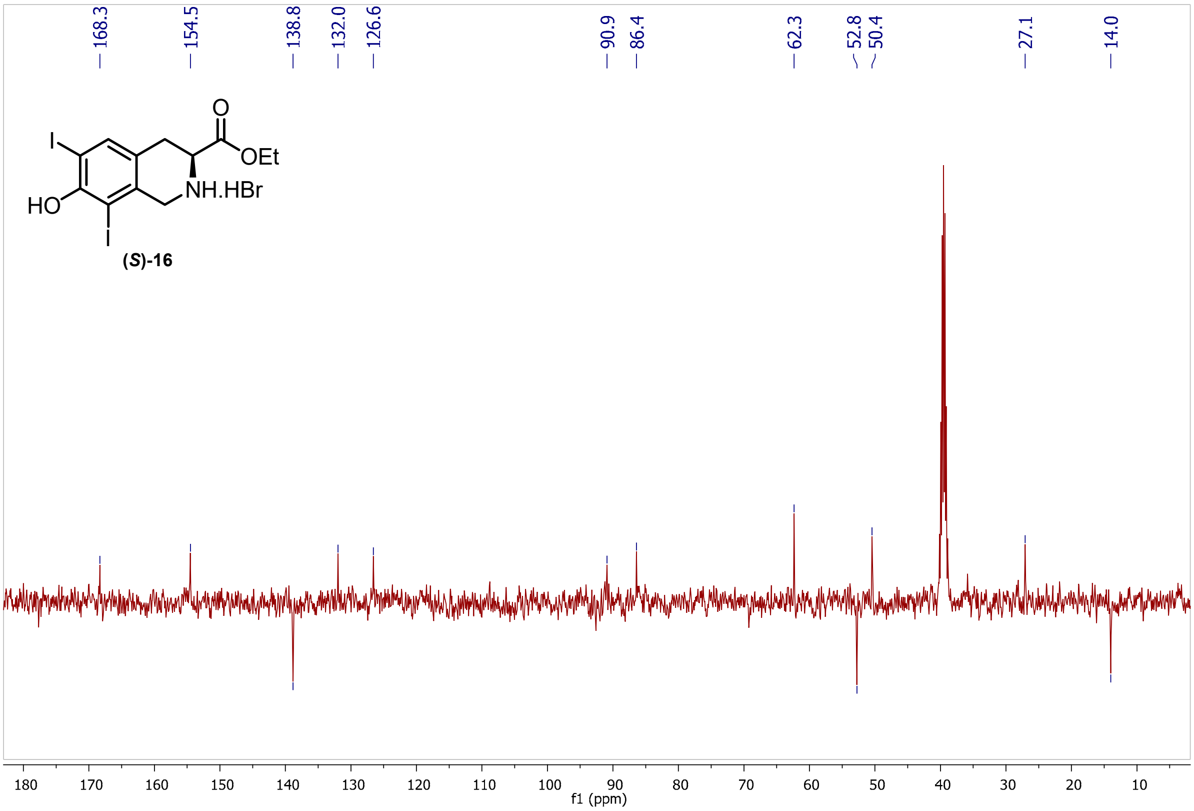

^{1}H (top) and ^{13}C (down) NMR spectra of compound **(*R*)-16** in DMSO-d_6.

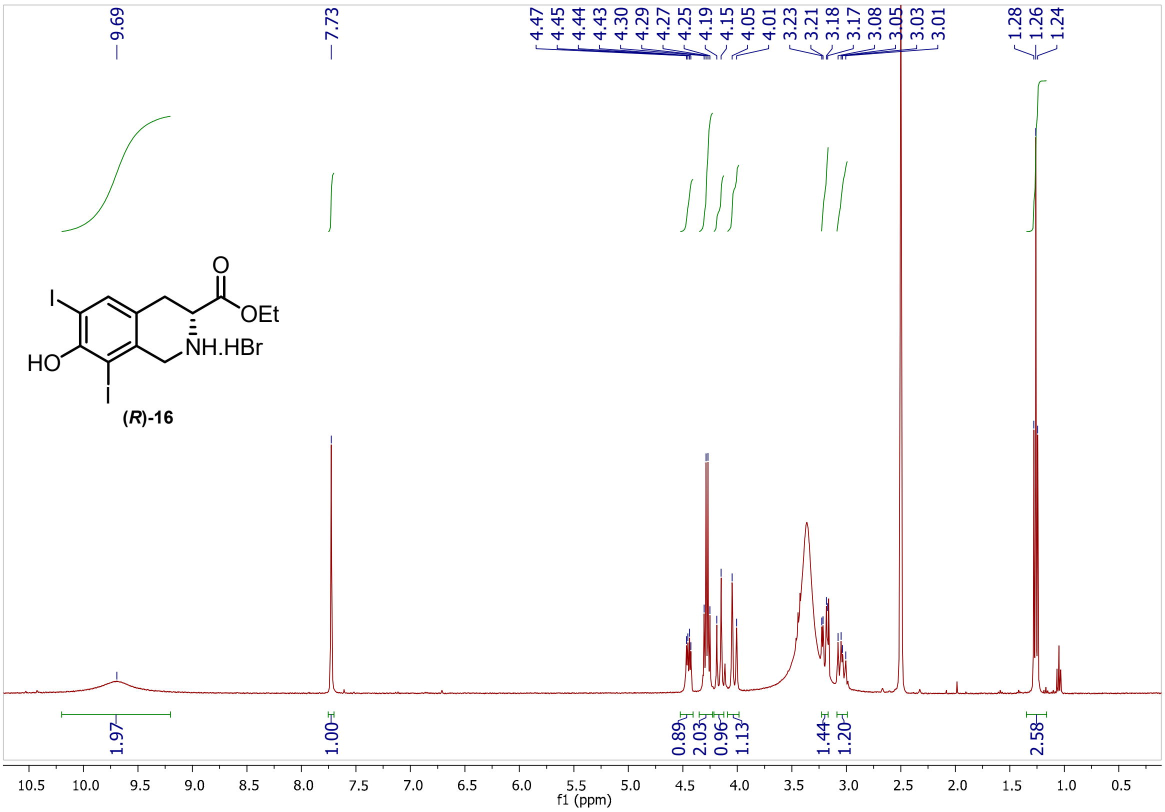

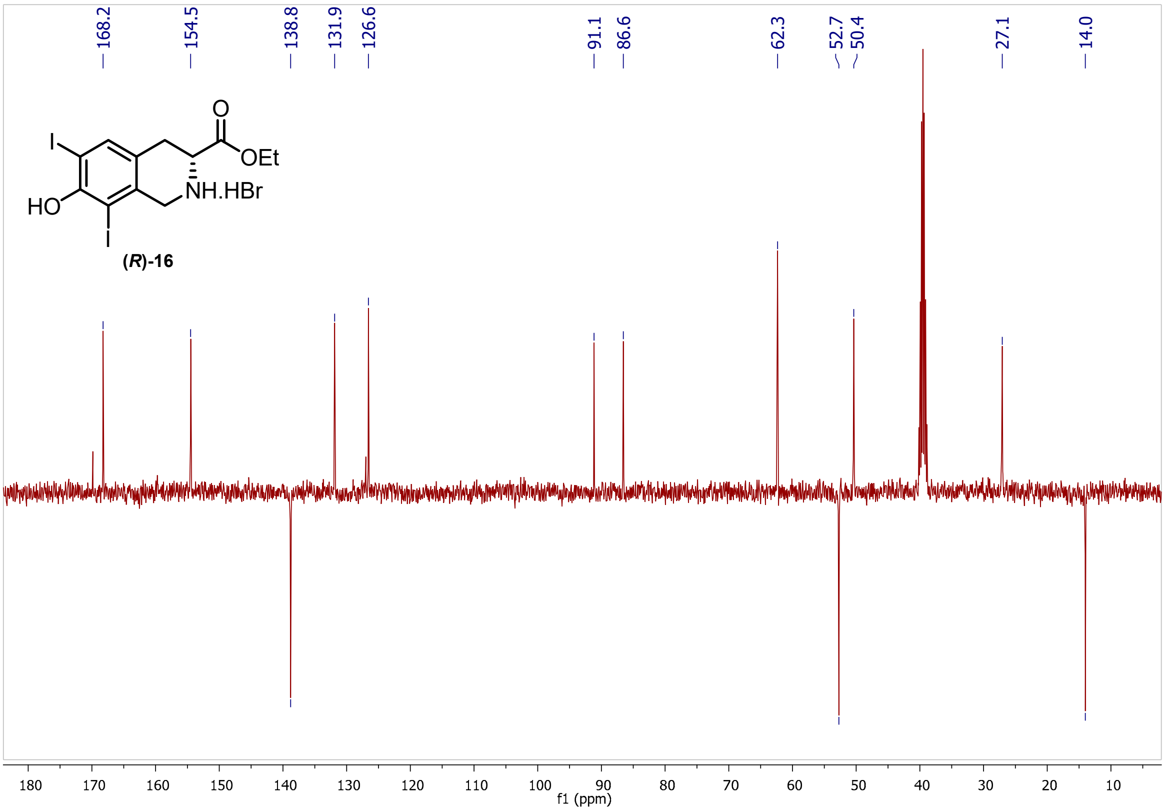

^{1}H (top) and ^{13}C (down) NMR spectra of compound **(*S*)-17** in DMSO-d_6.

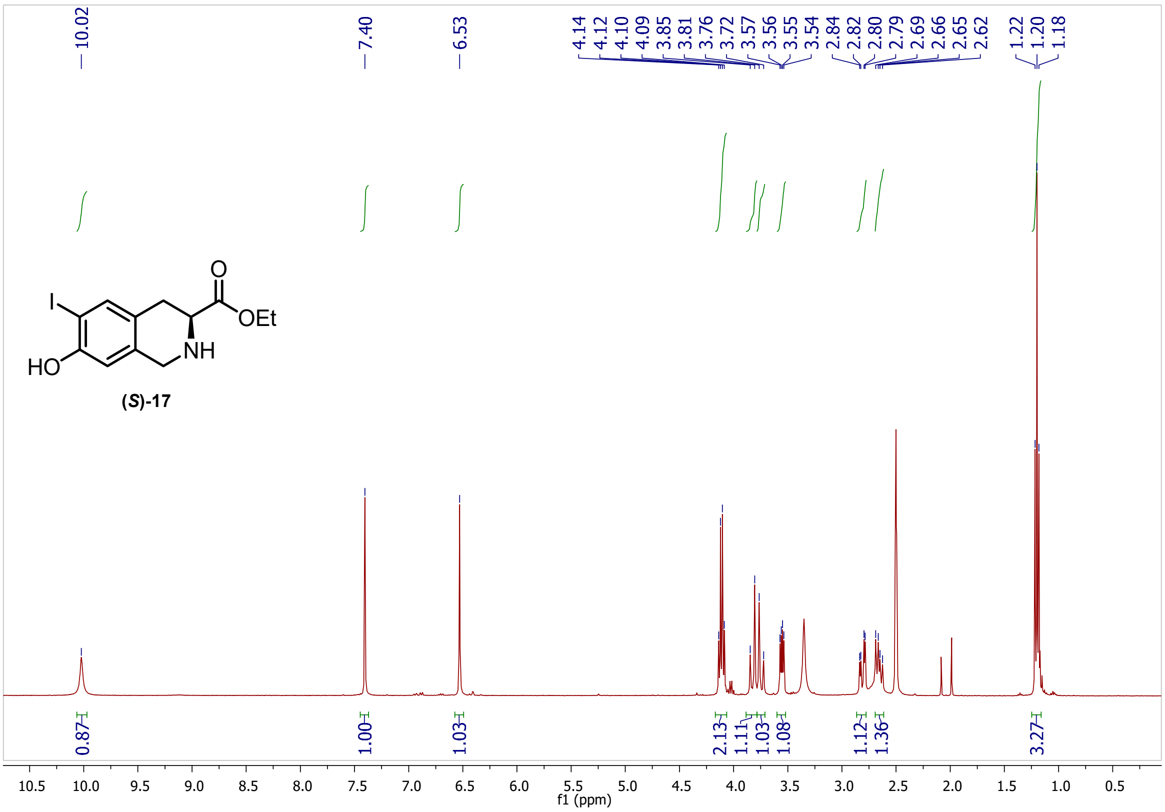

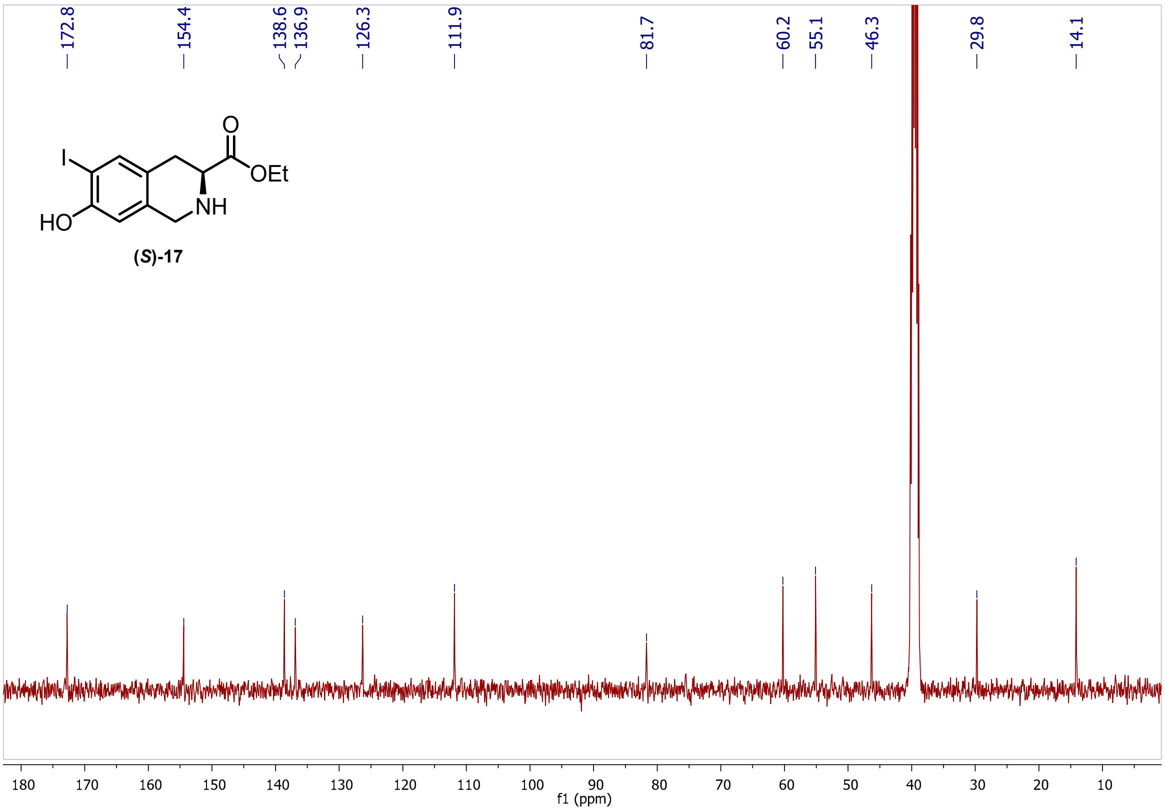

^{1}H (top) and ^{13}C (down) NMR spectra of compound **(*R*)-17** in DMSO-d_6.

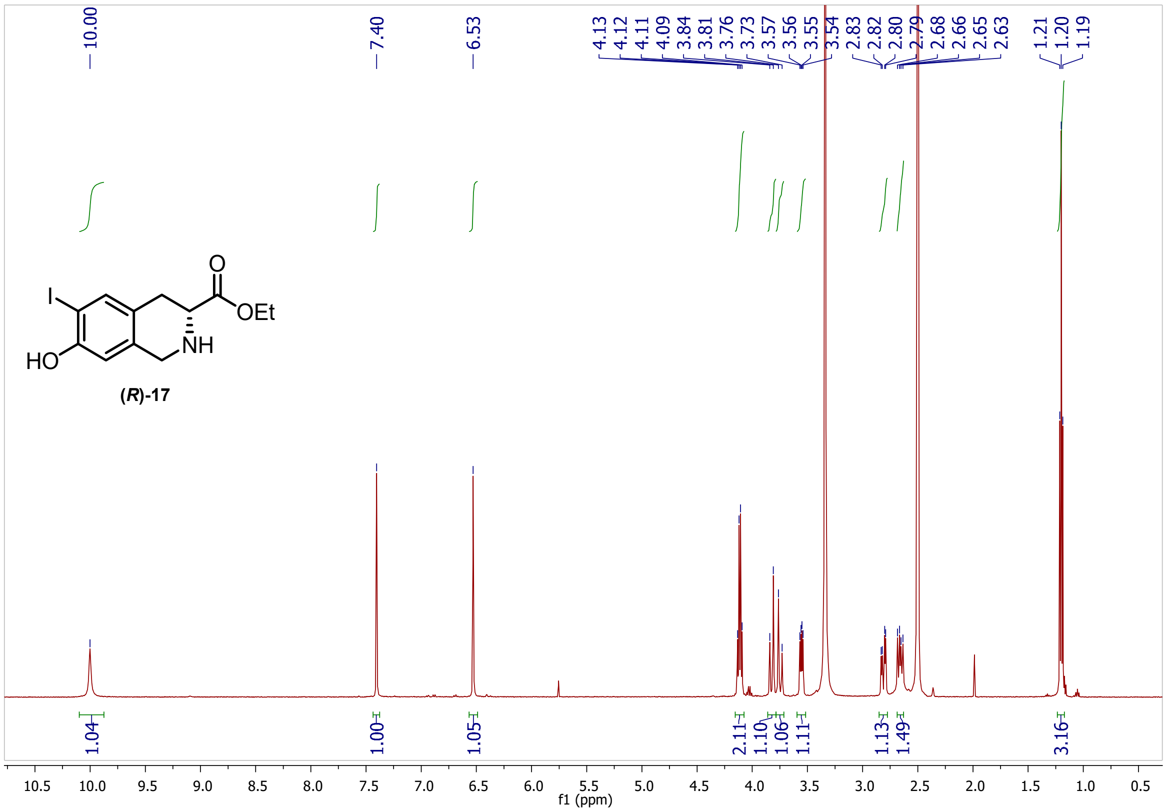

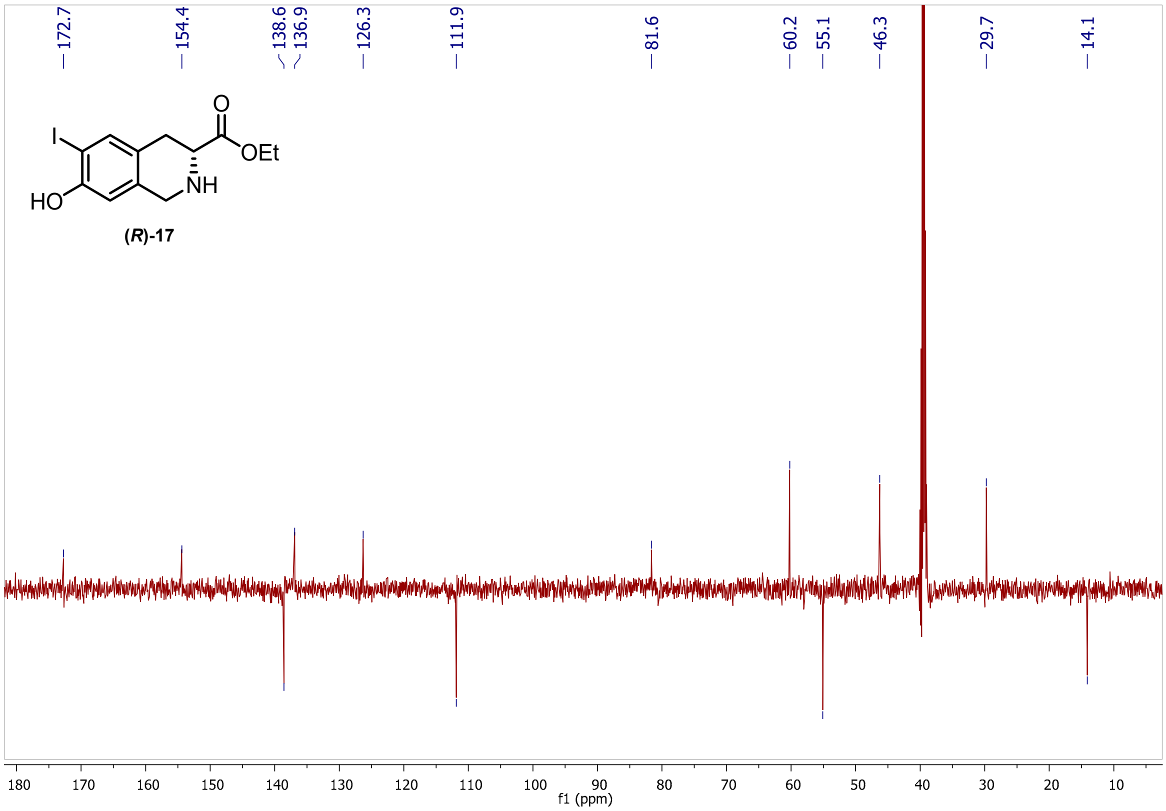

^1H (top) and ^{13}C (down) NMR spectra of compound **(*S*)-18 (6-iodo-L-TIC(OH))** in DMSO-d_6.

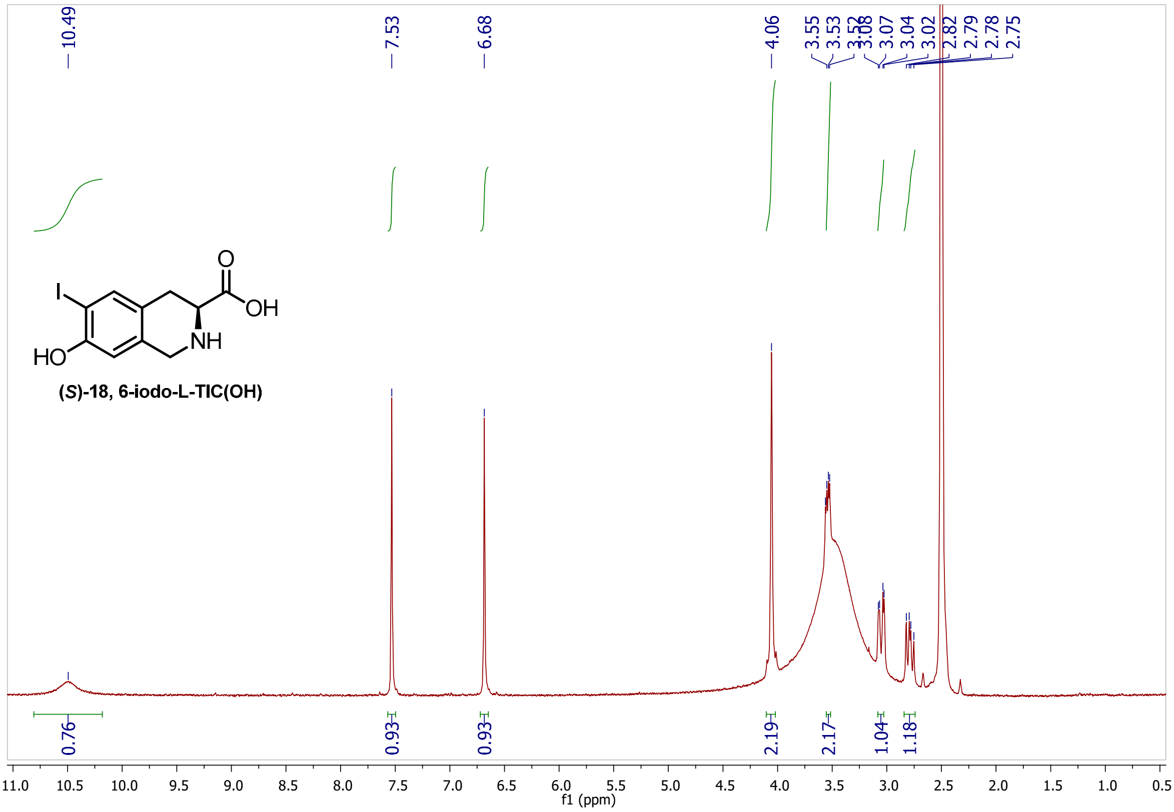

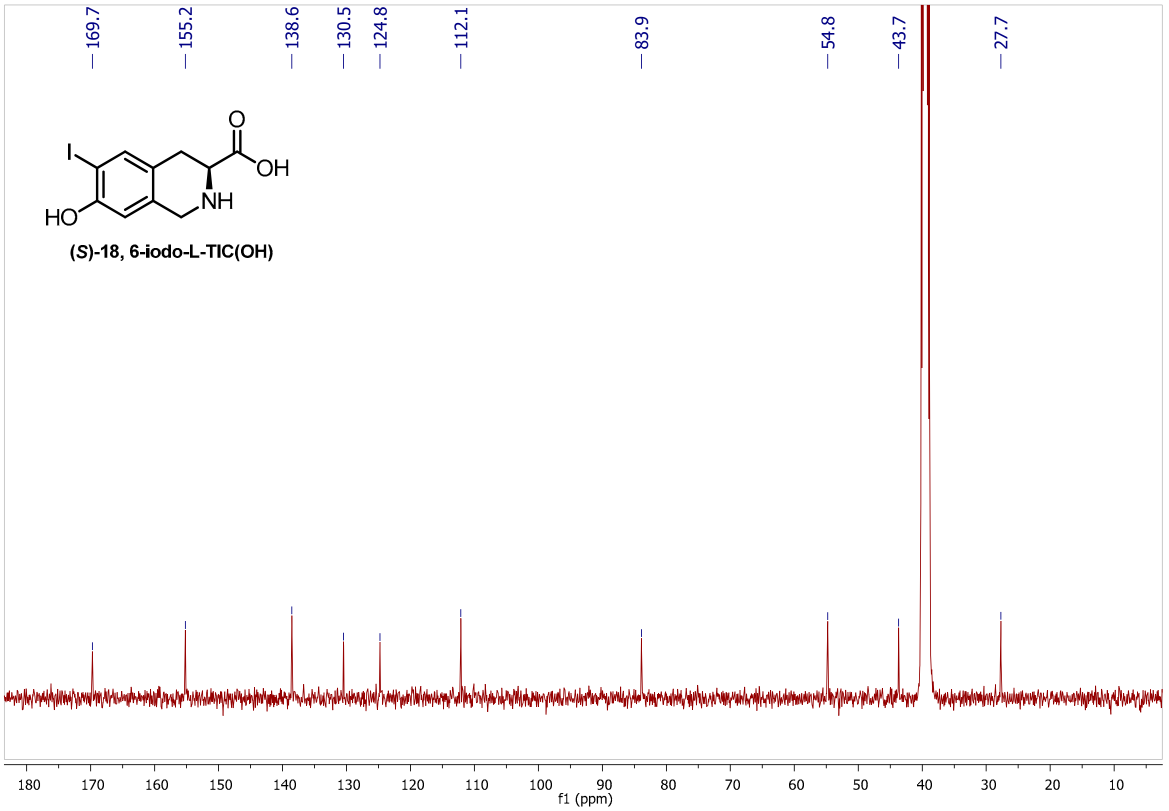

^{1}H (top) and ^{13}C (down) NMR spectra of compound ***(R)*-18 (6-iodo-D-TIC(OH))** in DMSO-*d*6.

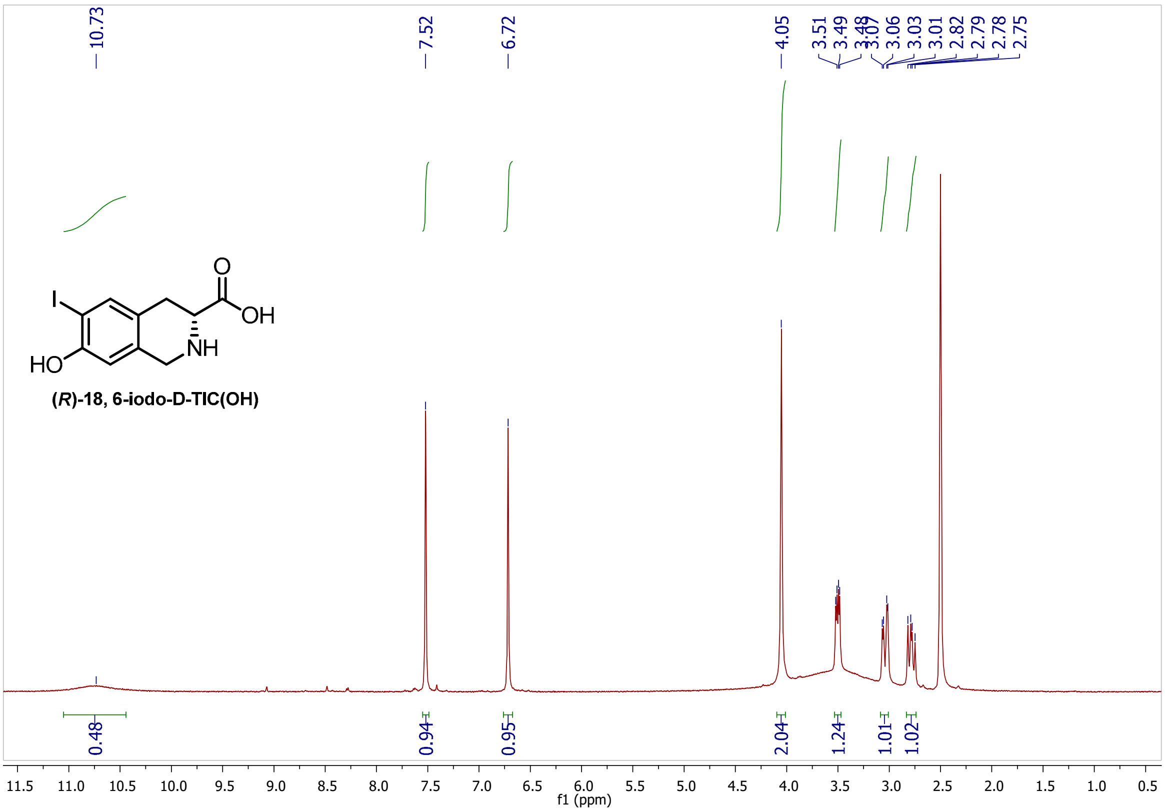

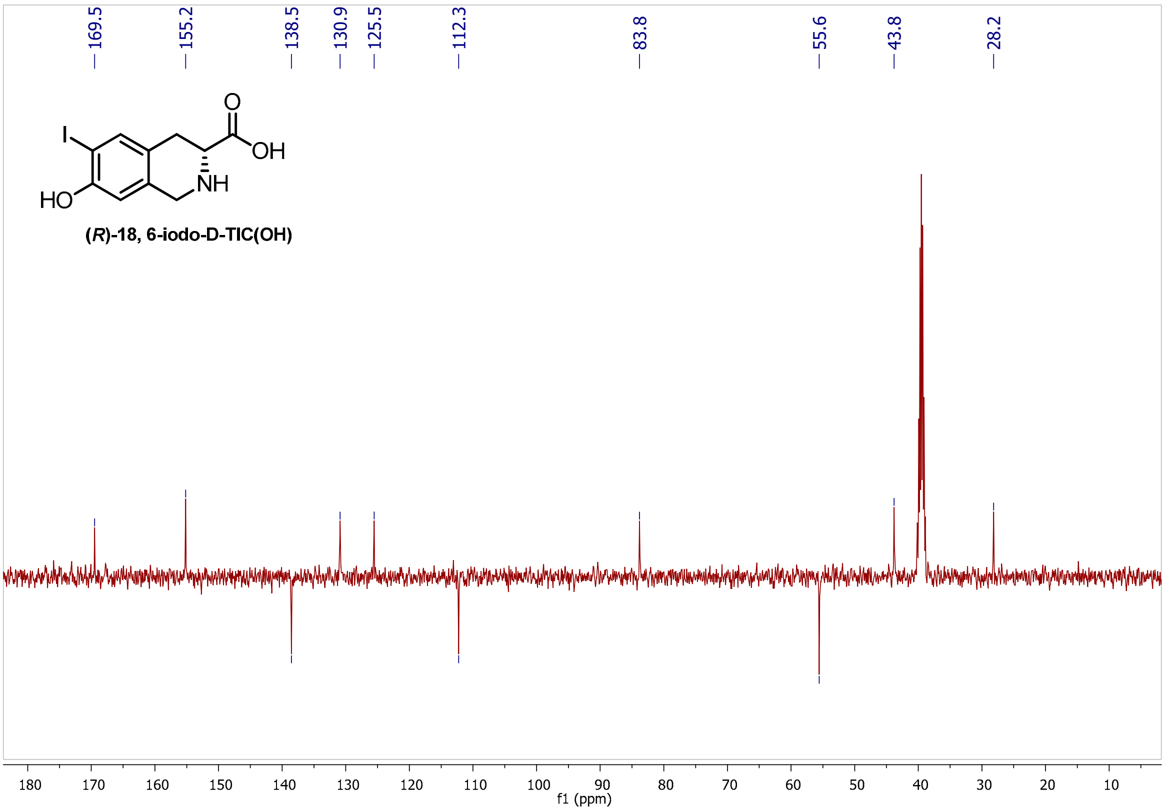

^{1}H (top) and ^{13}C (down) NMR spectra of compound **(*S*)-19** in CD_3OD.

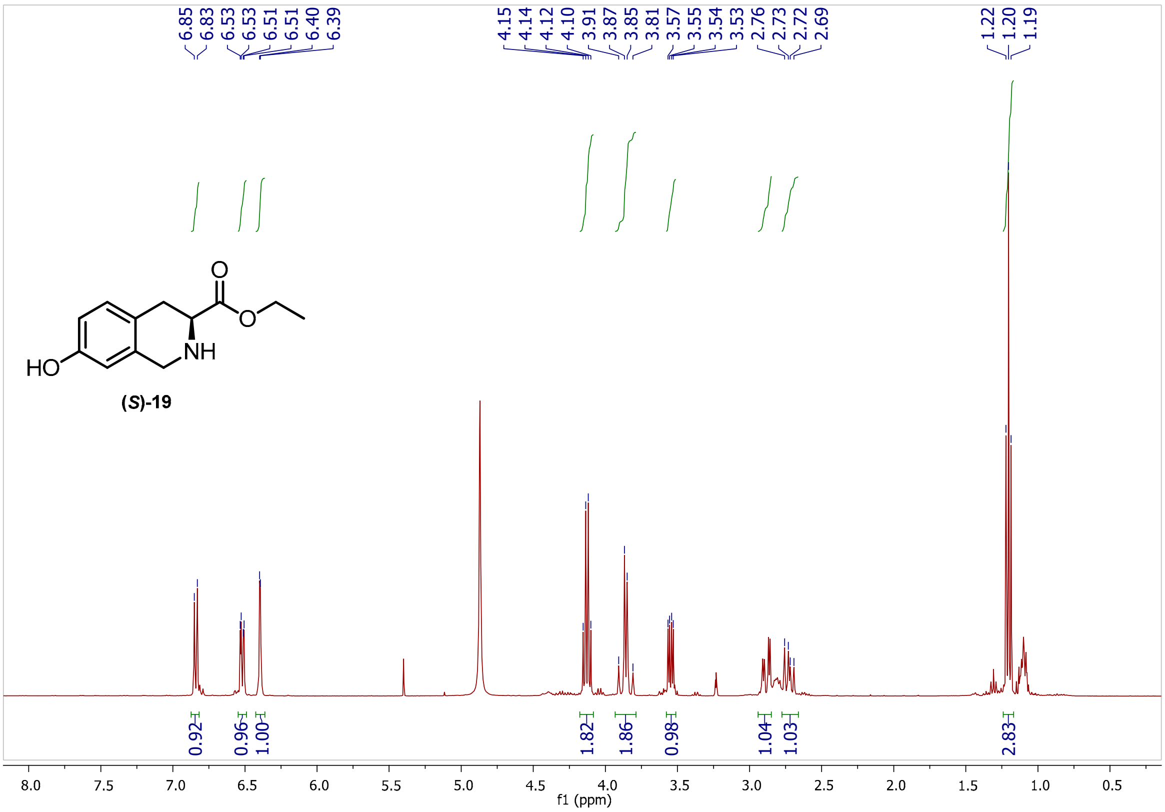

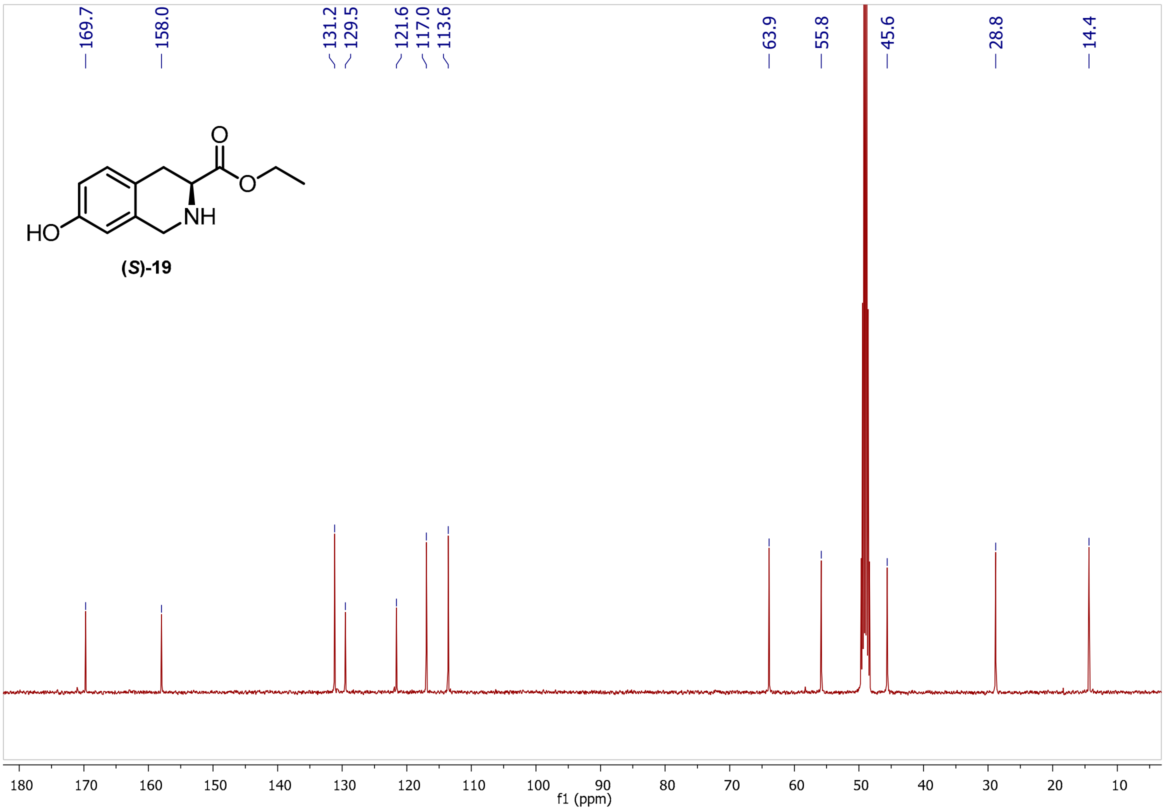

^{1}H (top) and ^{13}C (down) NMR spectra of compound **(*R*)-19** in CD_3OD.

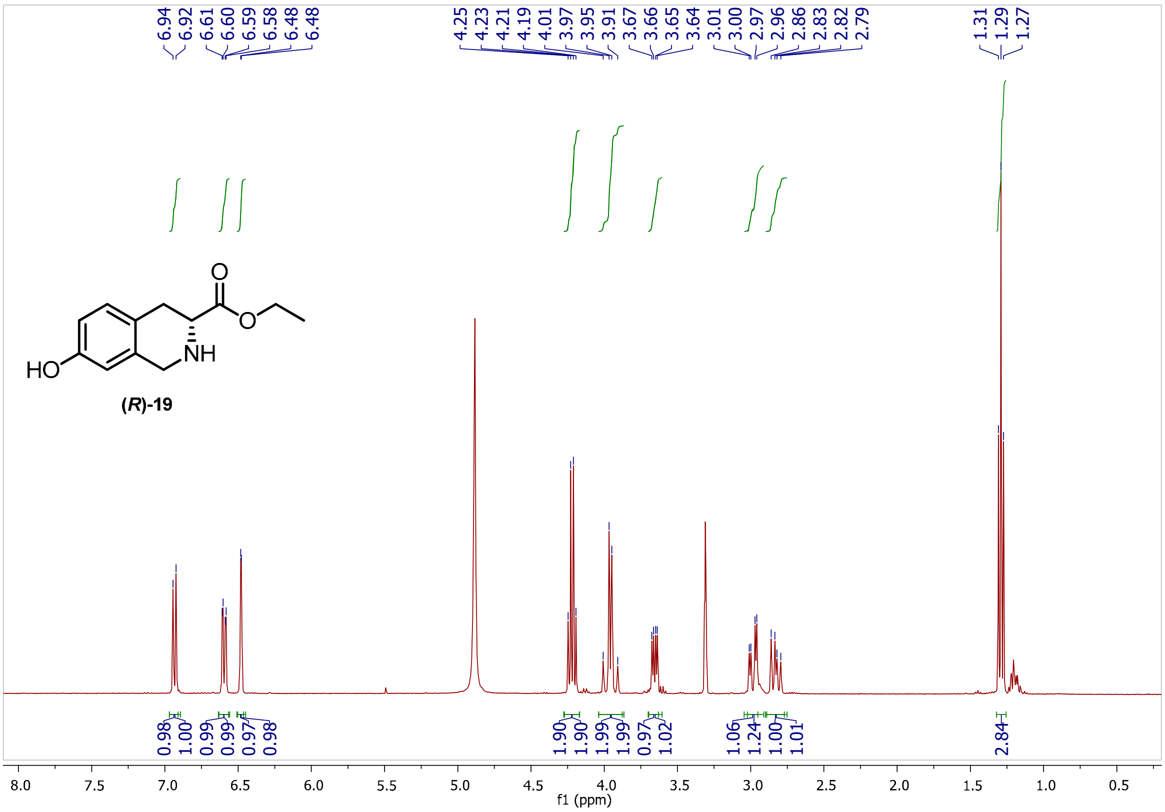

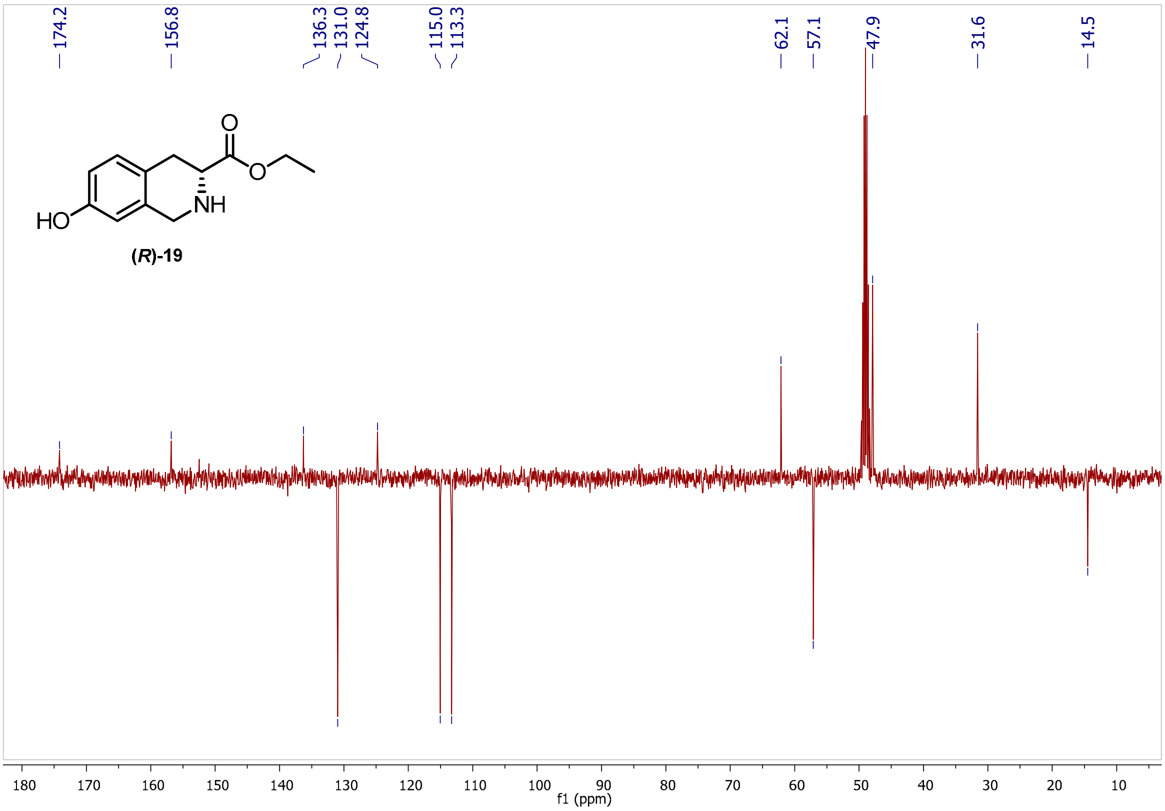

^{1}H (top) and ^{13}C (down) NMR spectra of compound ***(S)*-20** in CD_3OD.

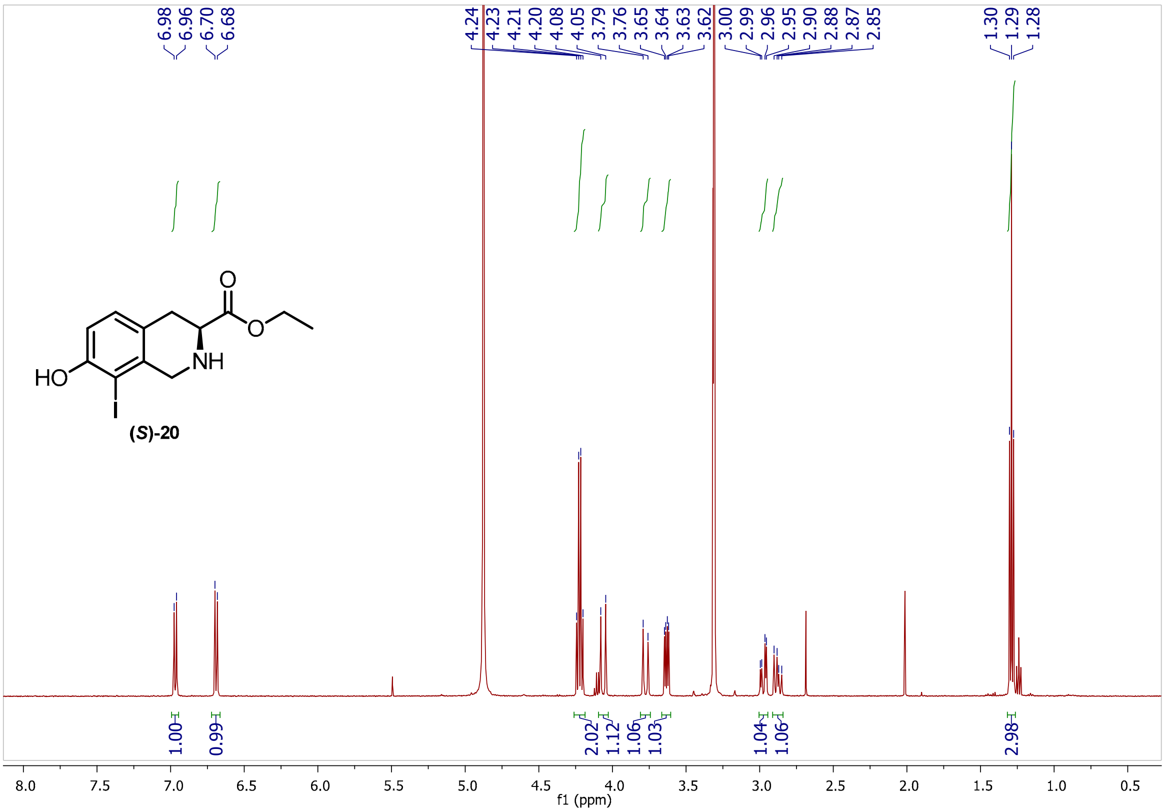

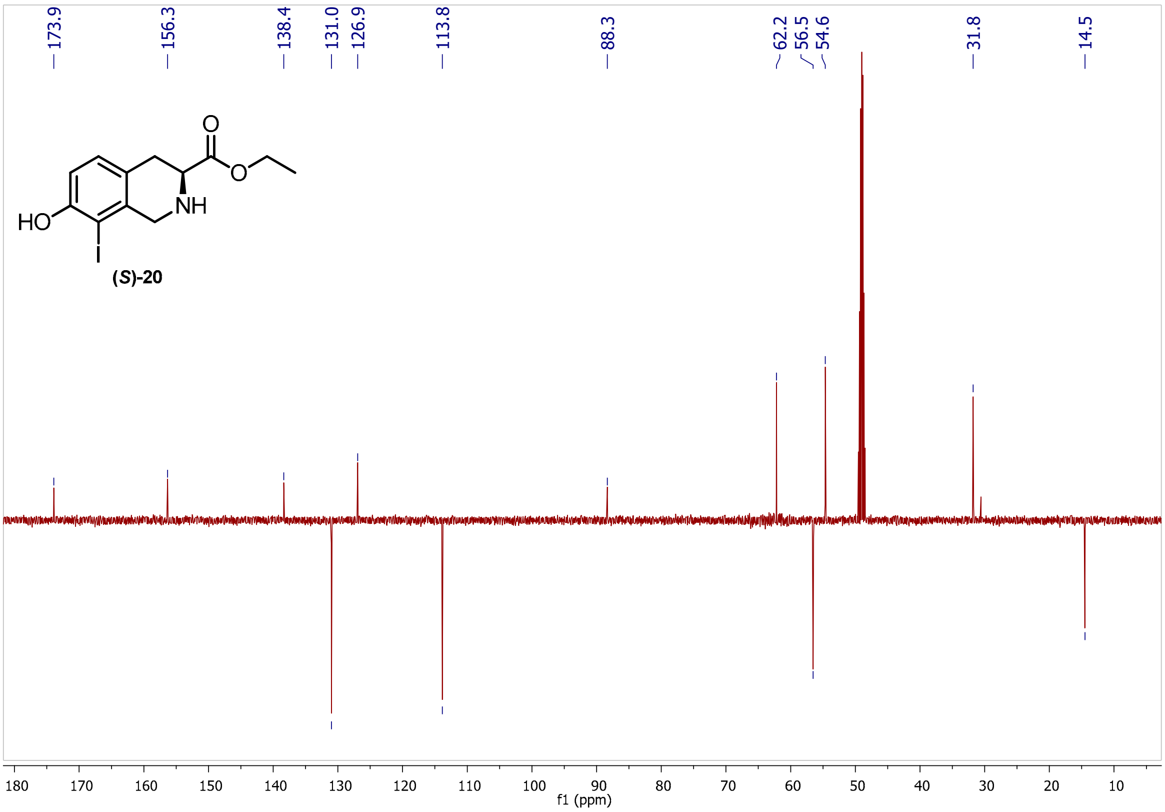

^{1}H (top) and ^{13}C (down) NMR spectra of compound **(*R*)-20** in CD_3OD.

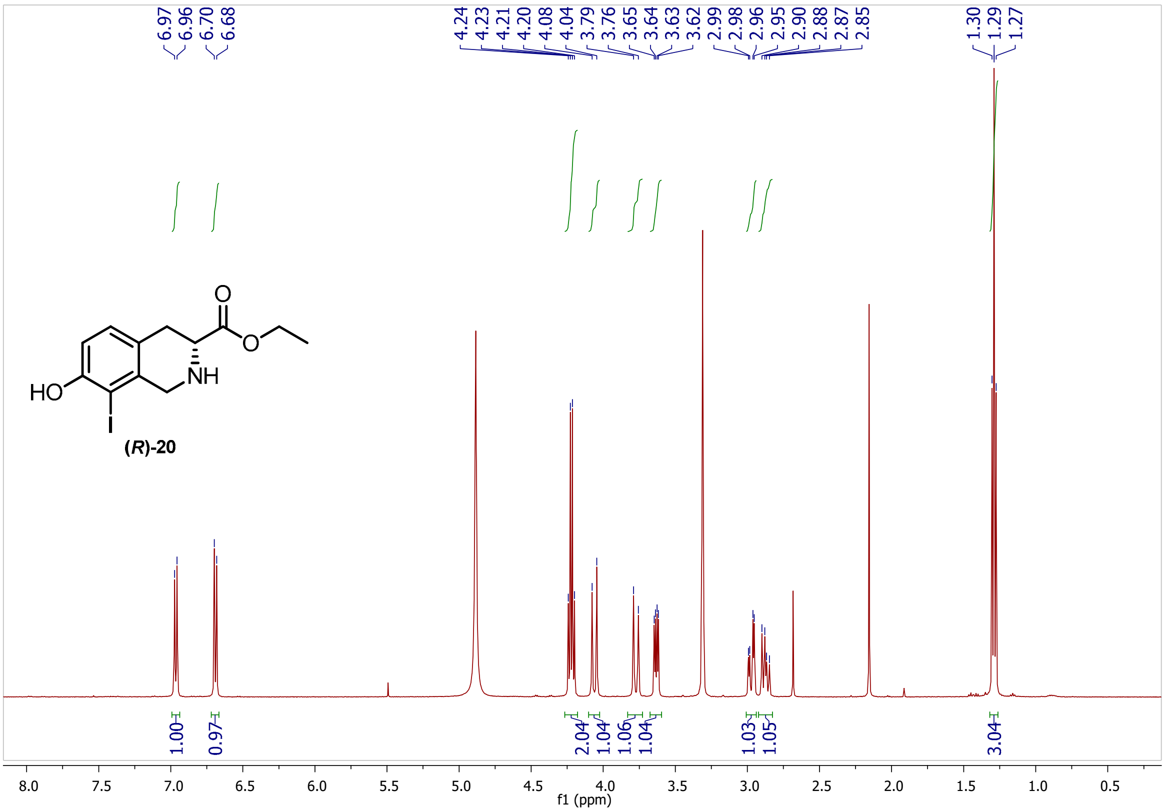

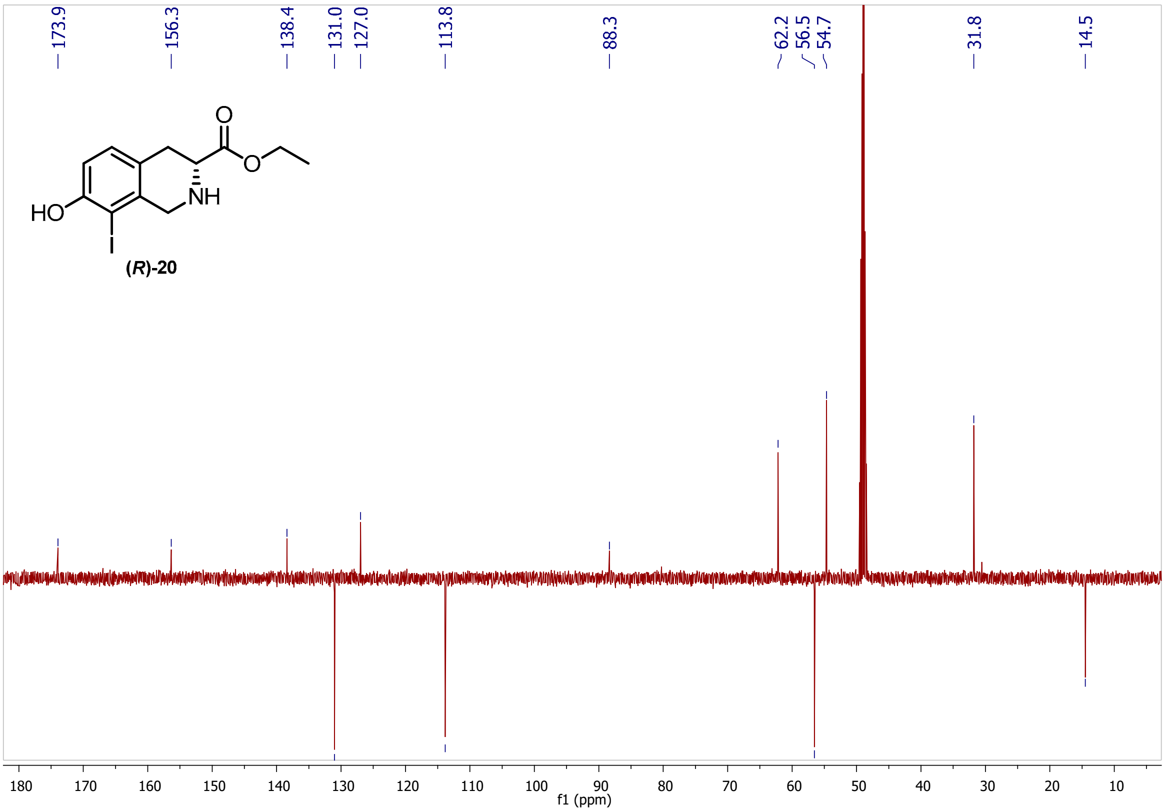

^{1}H (top) NMR spectra of compound **(*S*)-21 (8-iodo-L-TIC(OH))** in CD_3OD and ^{13}C (down) NMR spectra of compound **(*S*)-21 (8-iodo-L-TIC(OH))** in DMSO-d_6.

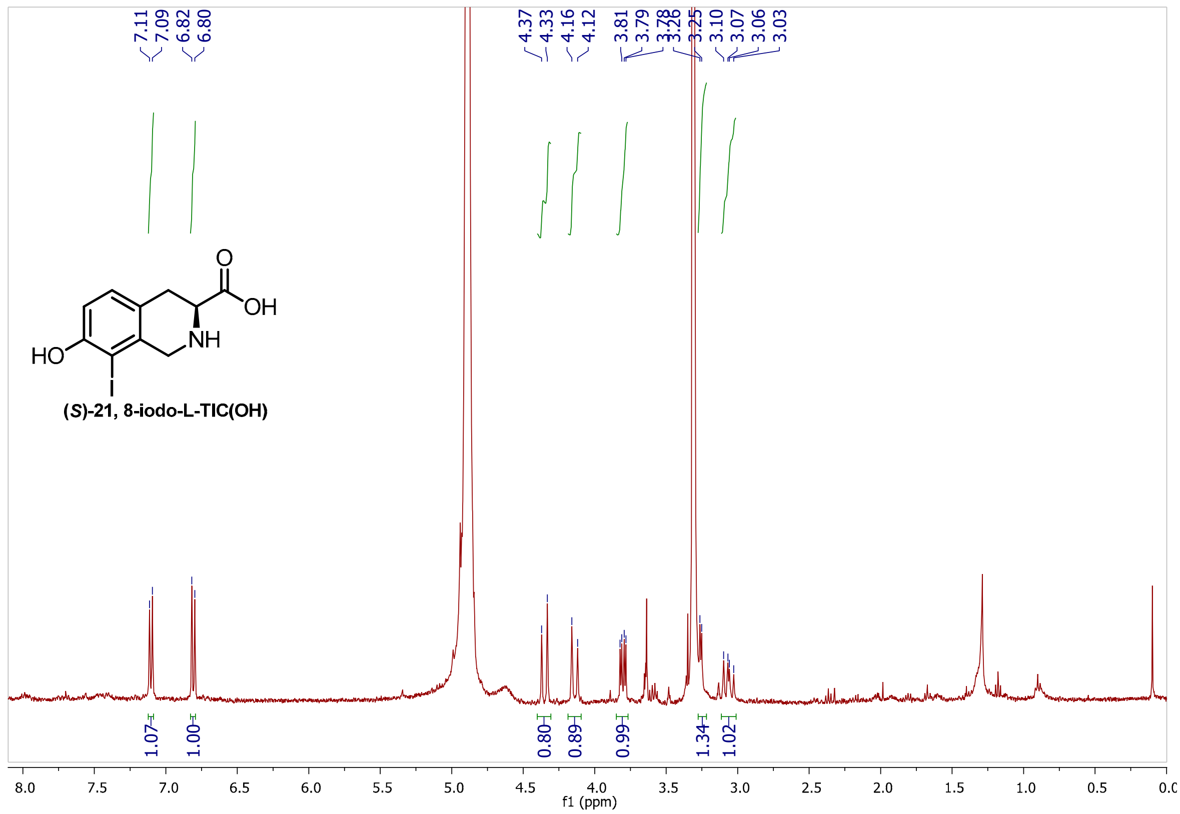

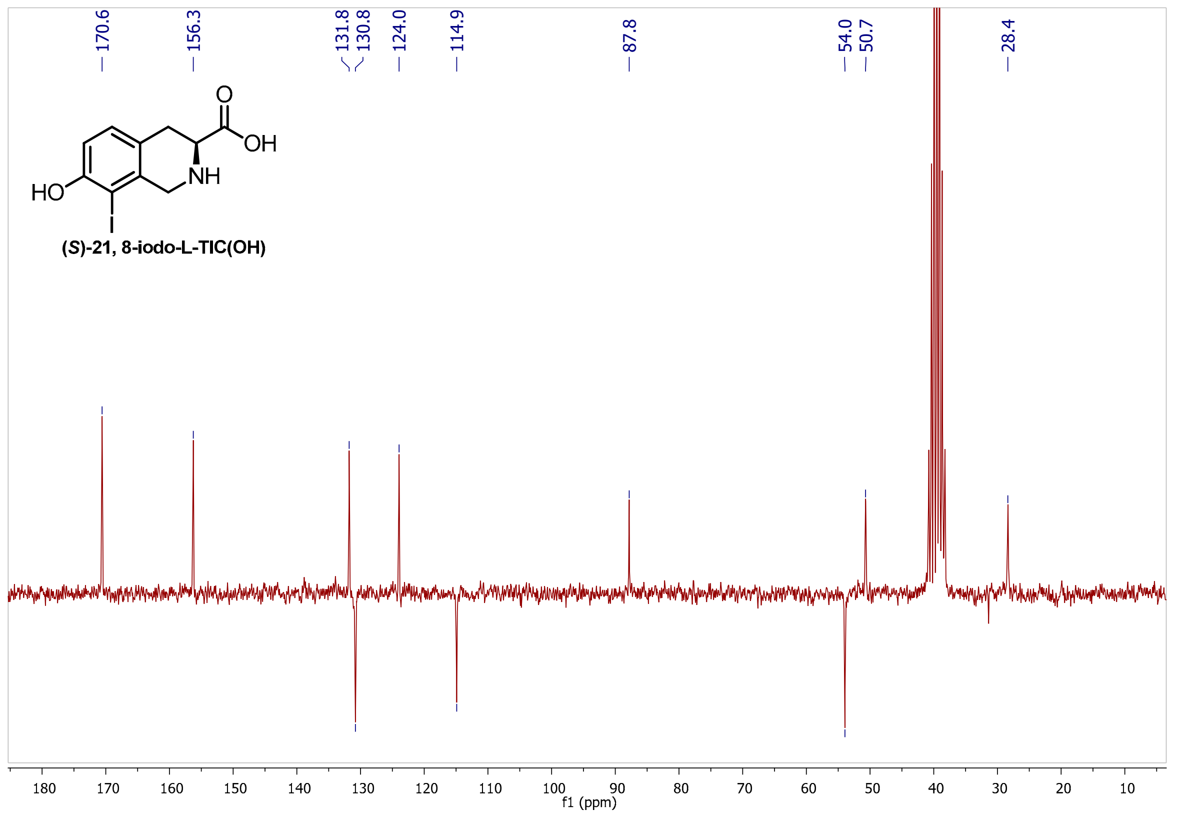

^{1}H (top) NMR spectra of compound **(*R*)-21 (8-iodo-D-TIC(OH))** in D_2O and ^{13}C (down) NMR spectra of compound **(*R*)-21 (8-iodo-D-TIC(OH))** in DMSO-d_6.

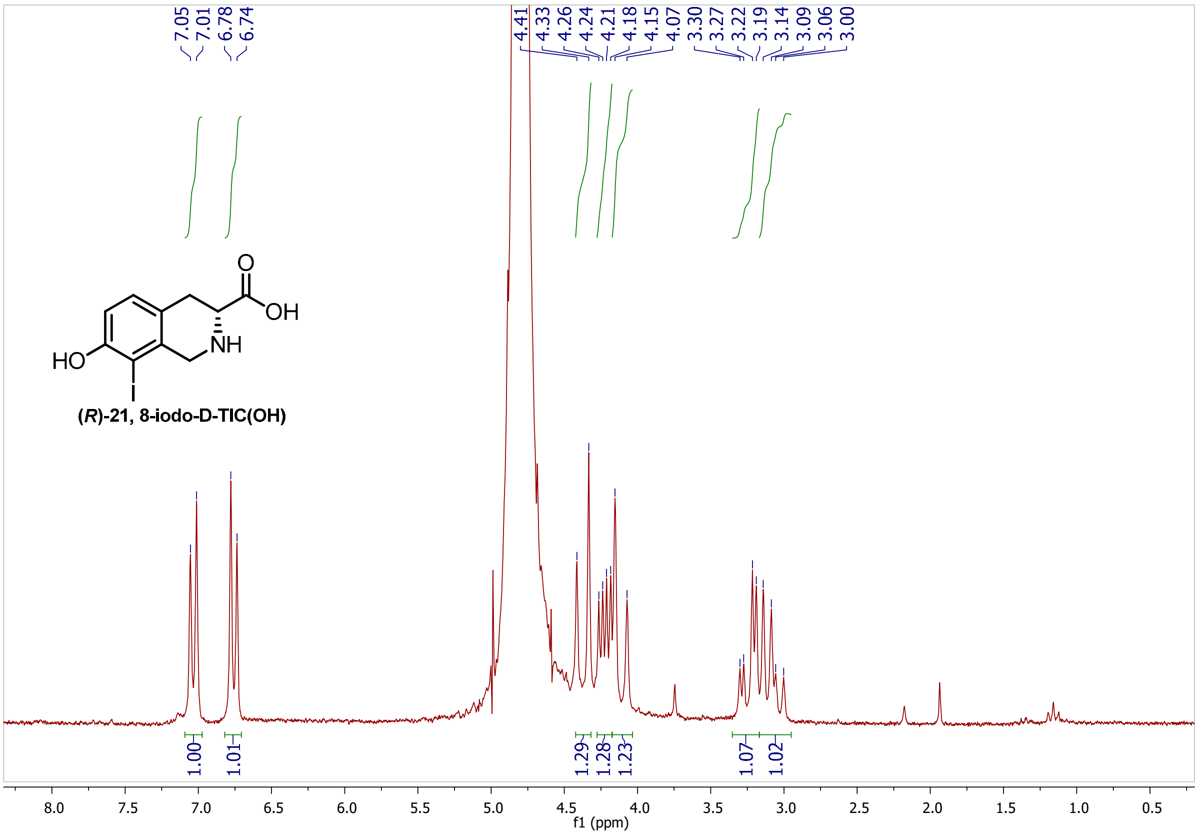

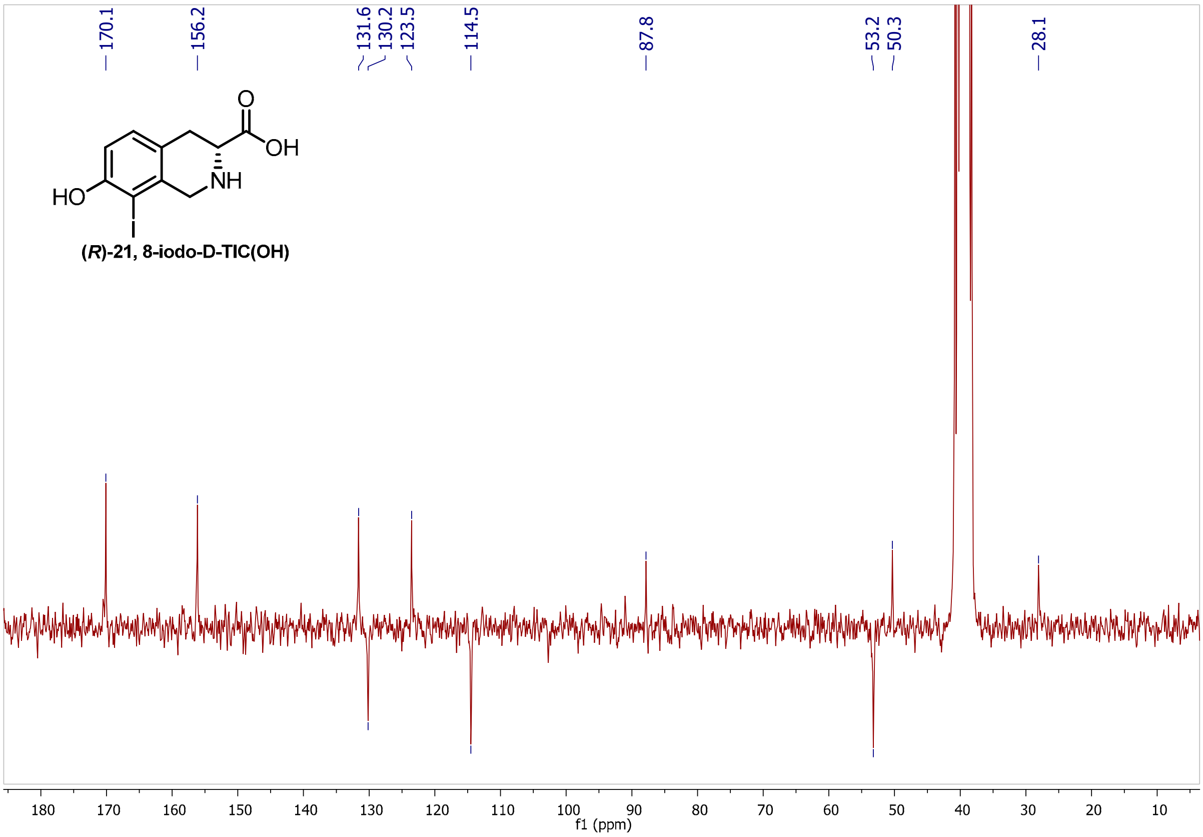

^1H (top) and ^{13}C (down) NMR spectra of compound **(*S*)-22** in $CDCl_3$.

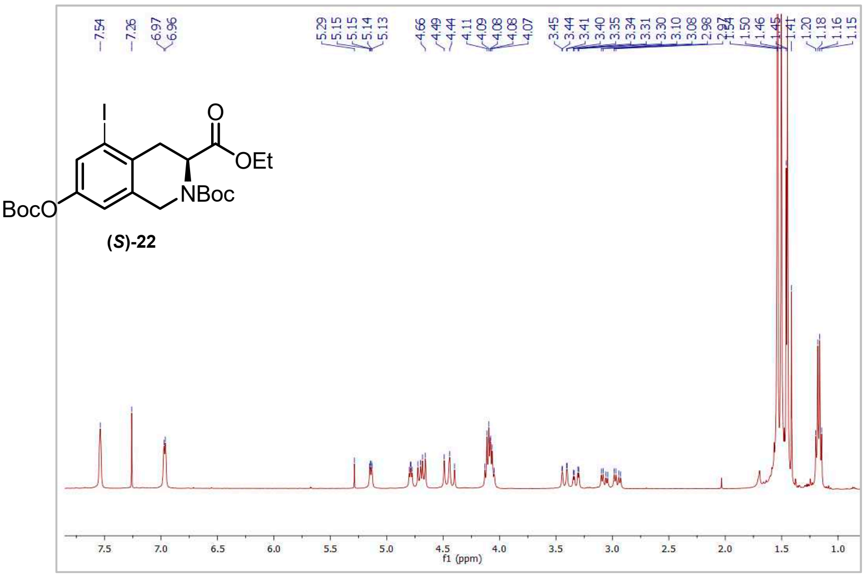

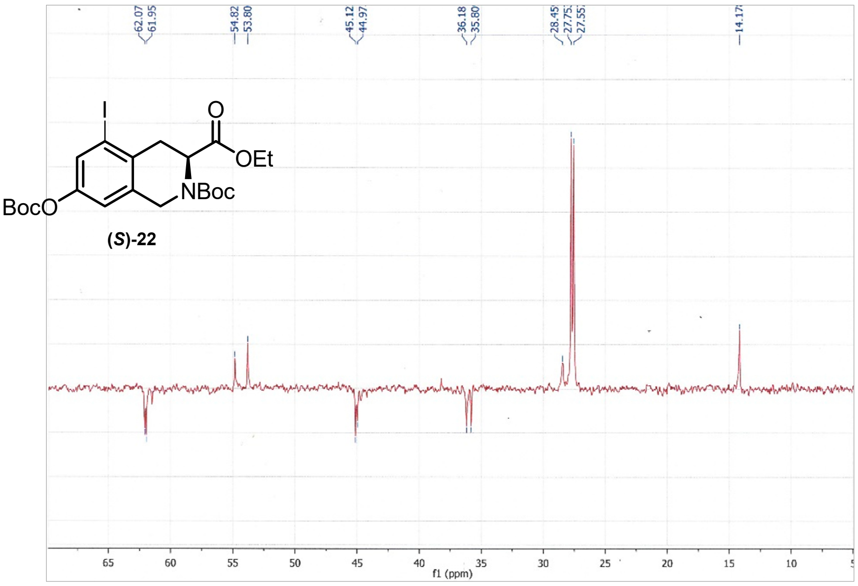

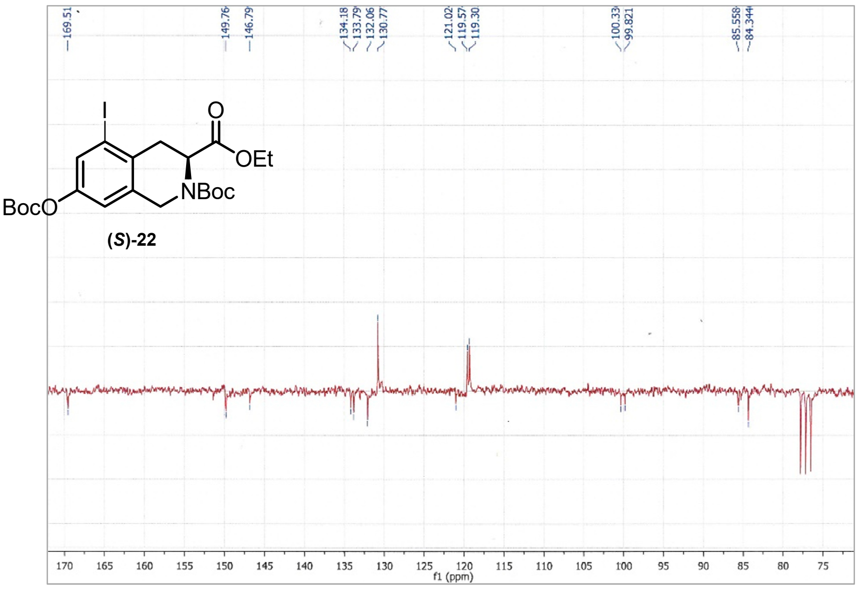

^{1}H (top) and ^{13}C (down) NMR spectra of compound ***(S)*-23** in $CDCl_3$.

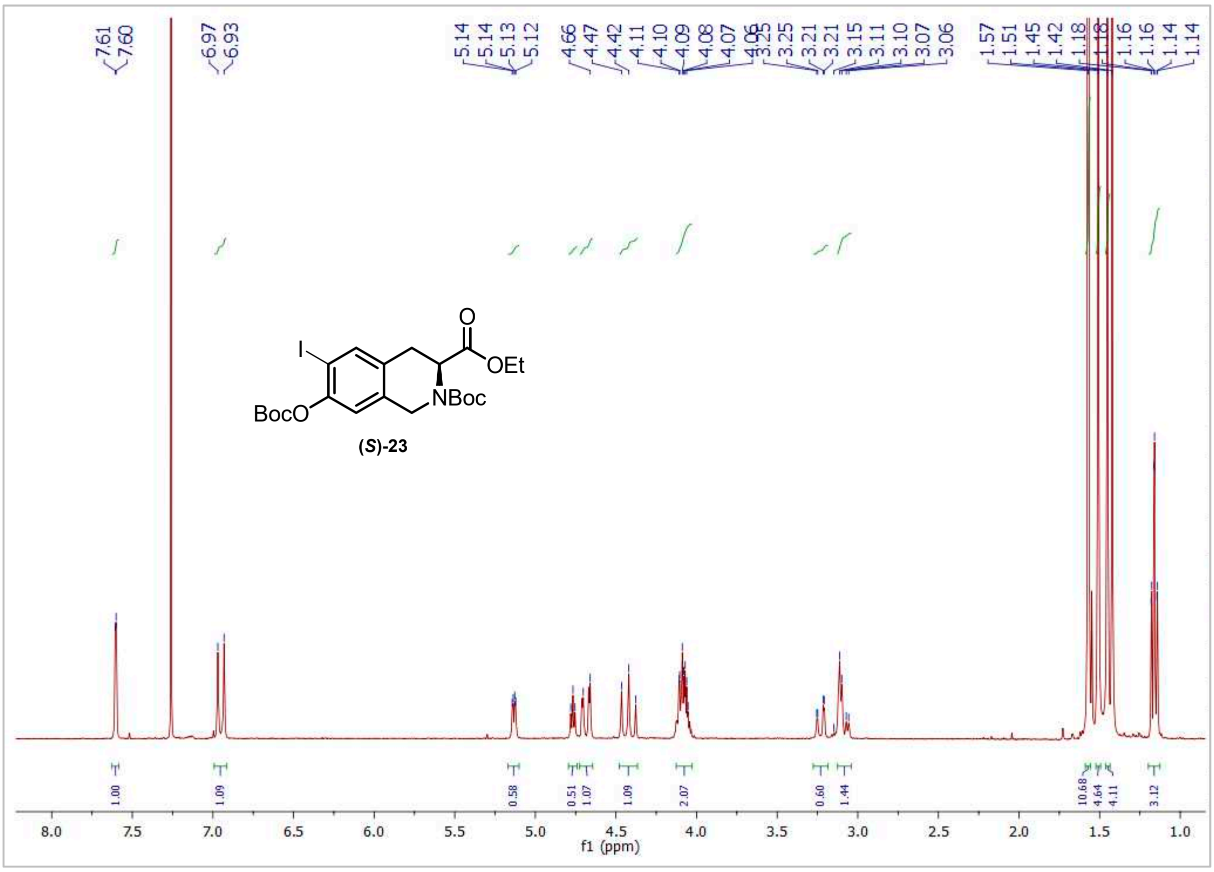

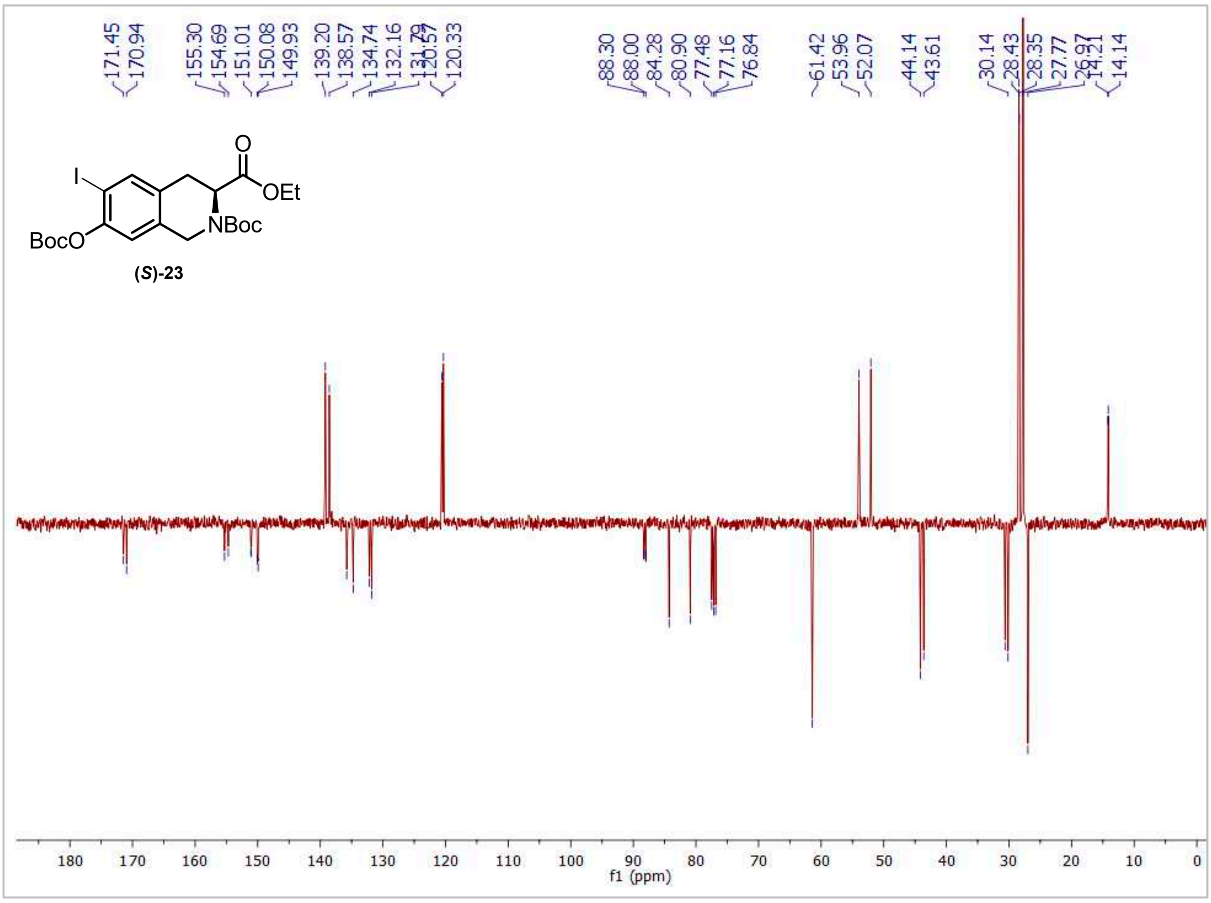

^{1}H (top) and ^{13}C (down) NMR spectra of compound **(*S*)-24** in CDCl3.

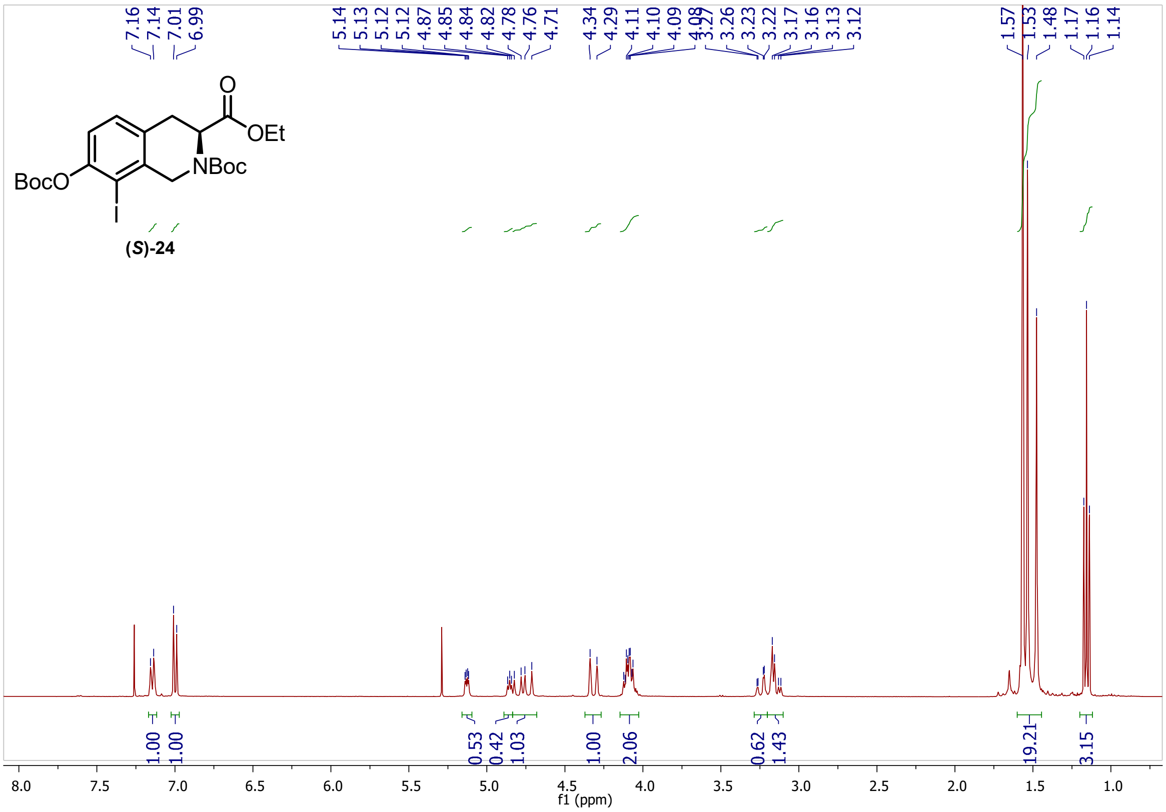

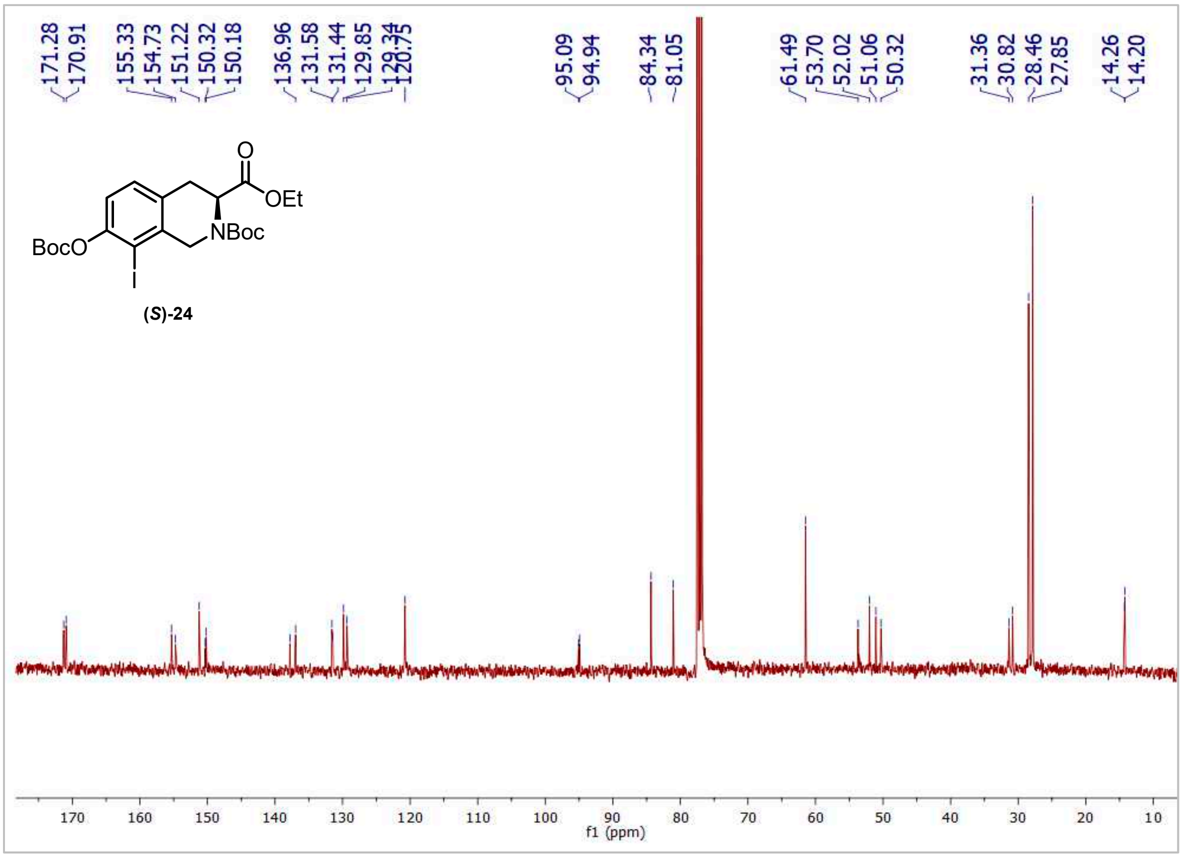

^{1}H (top) and ^{13}C (down) NMR spectra of compound **(*S*)-25** in $CDCl_3$.

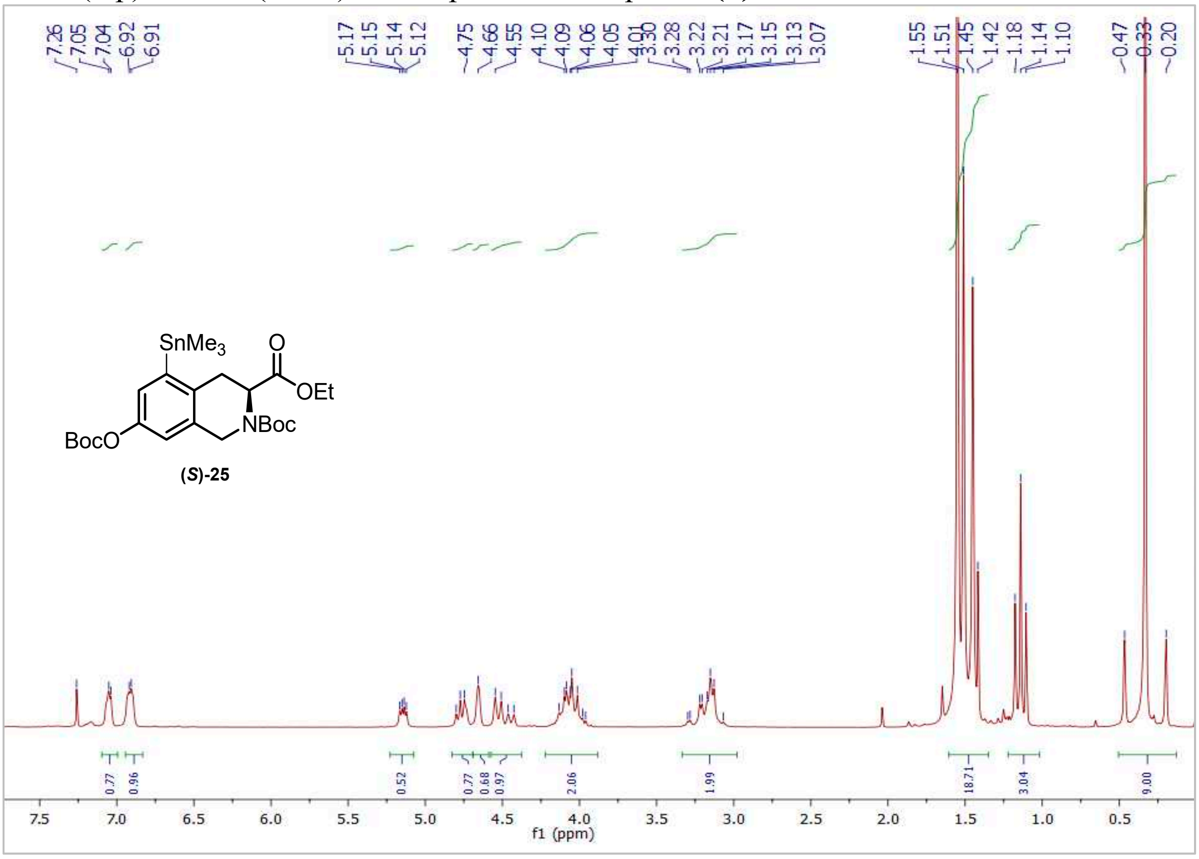

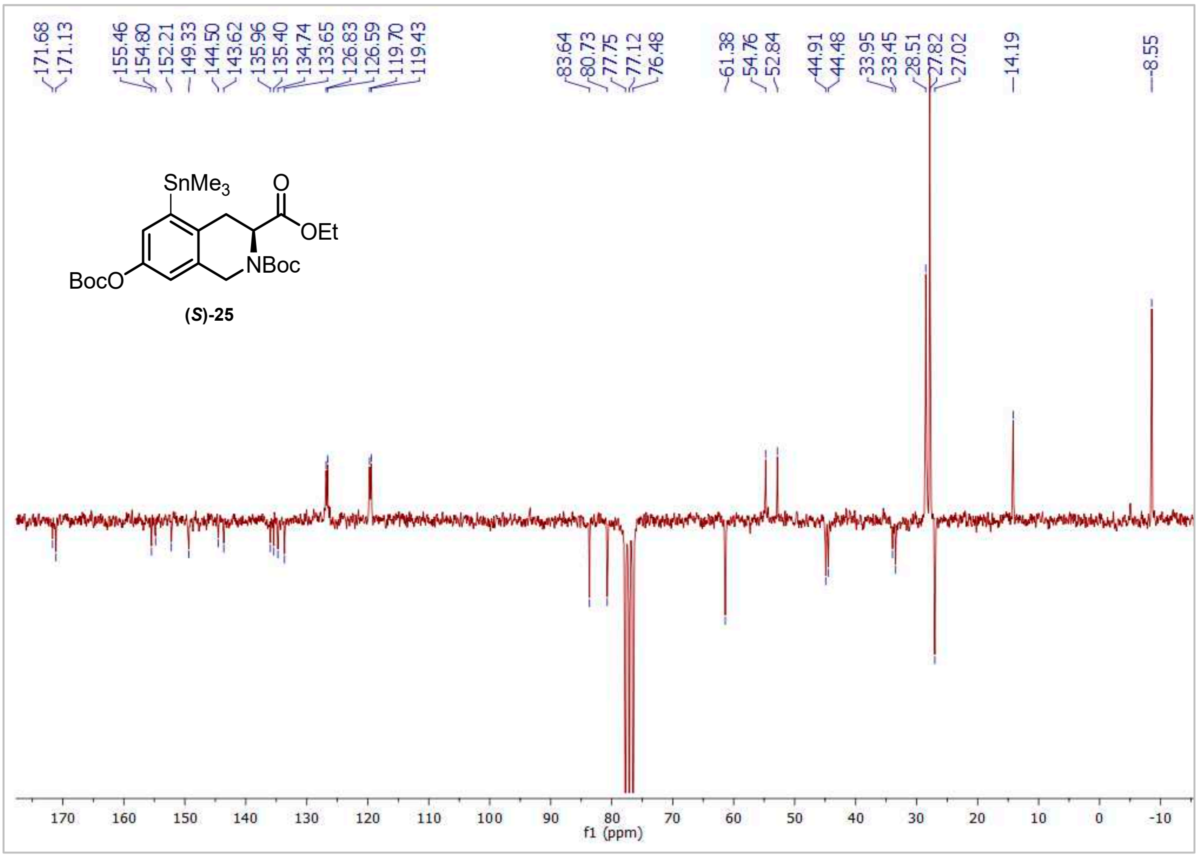

^{1}H (top) and ^{13}C (down) NMR spectra of compound **(*S*)-26** in $CDCl_3$.

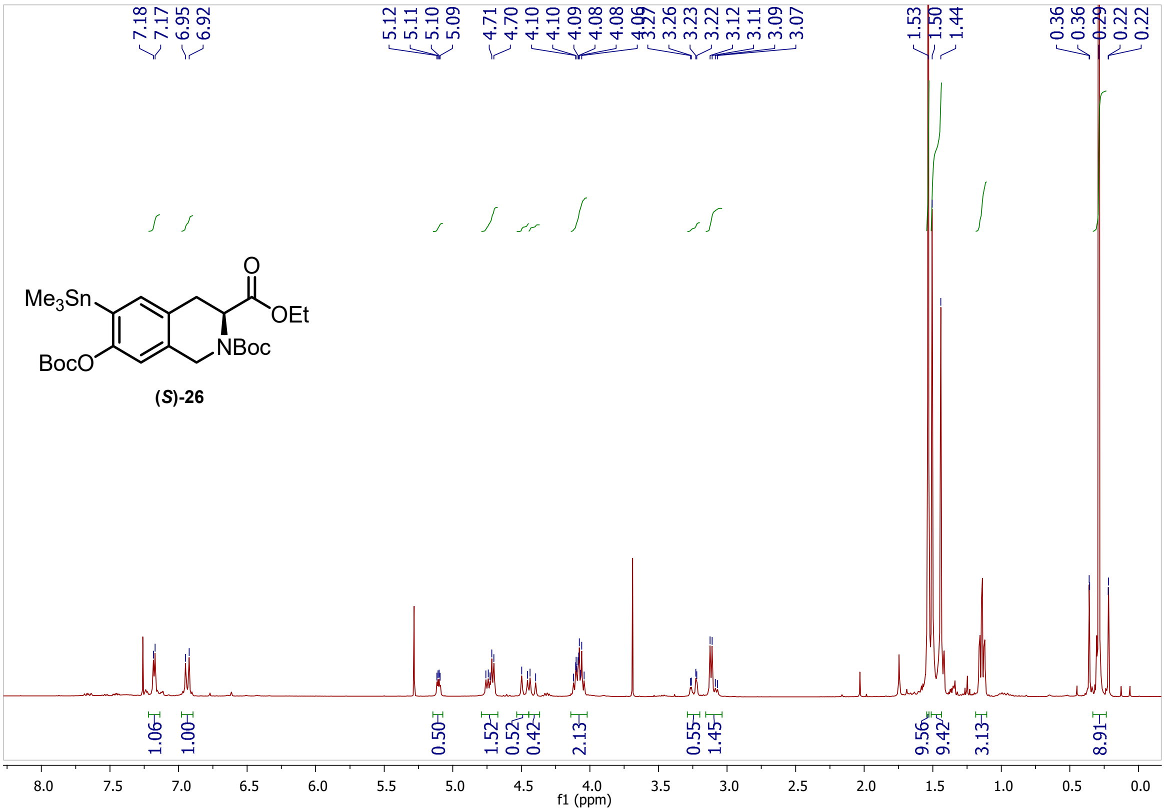

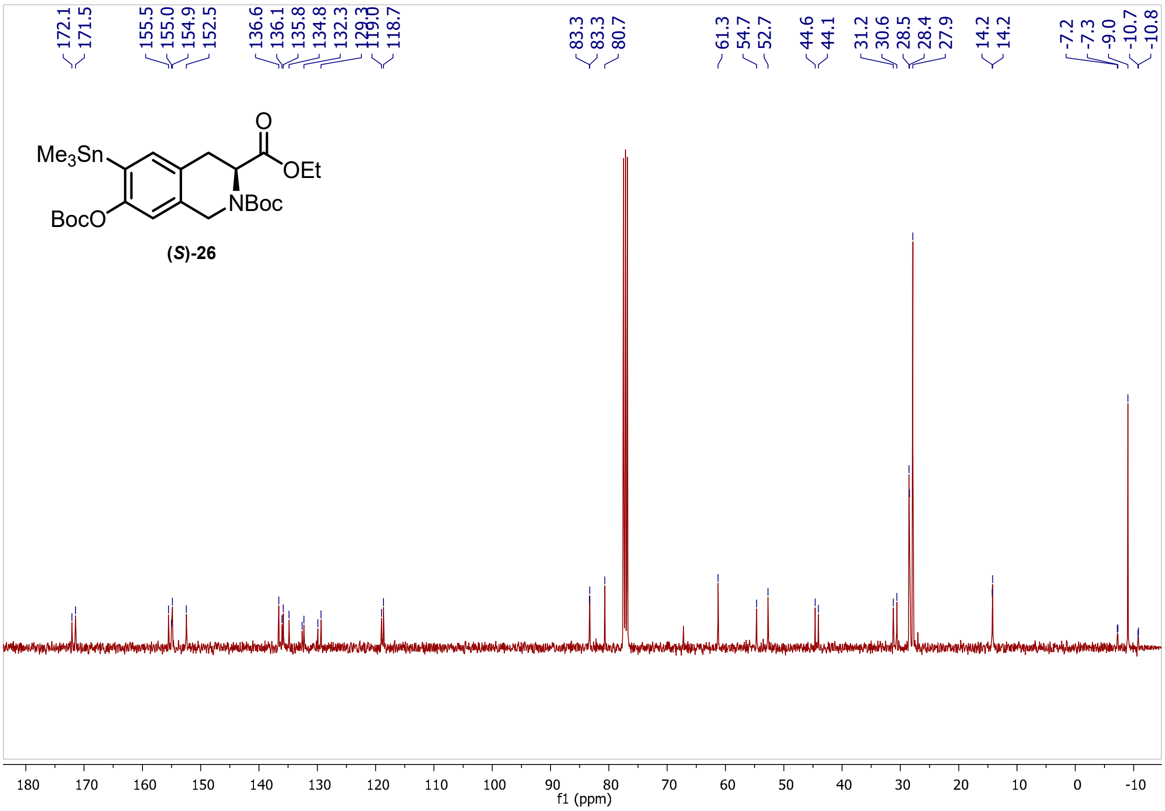

^{1}H (top) and ^{13}C (down) NMR spectra of compound **(*S*)-27** in CDCl3.

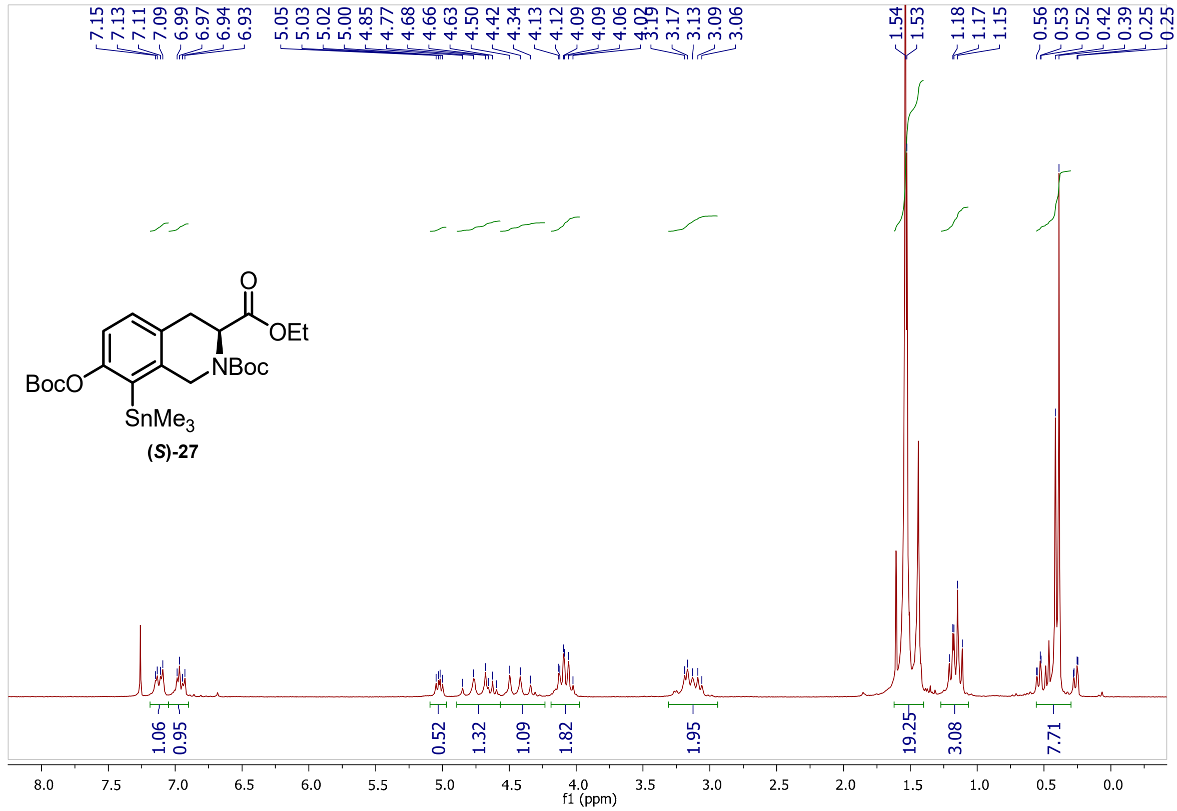

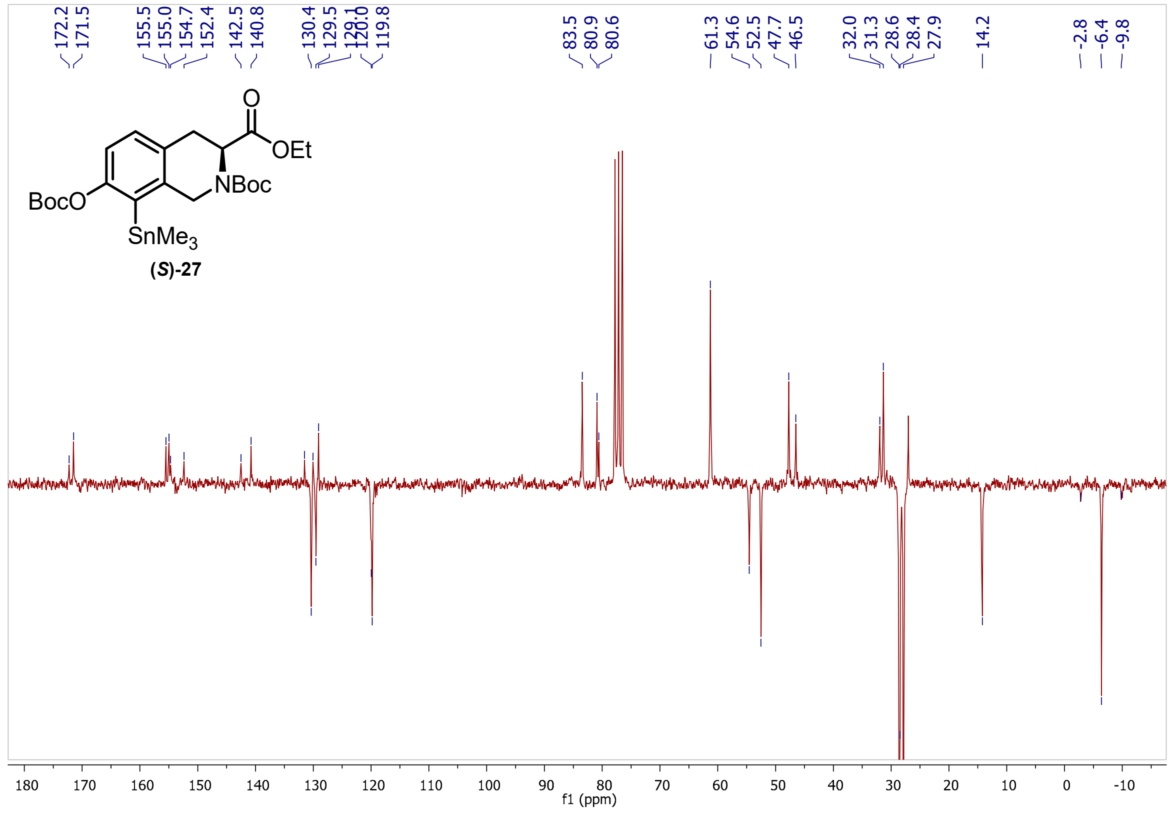

^{1}H (top) and ^{13}C (down) NMR spectra of compound ***(S)*-28** in $CDCl_3$.

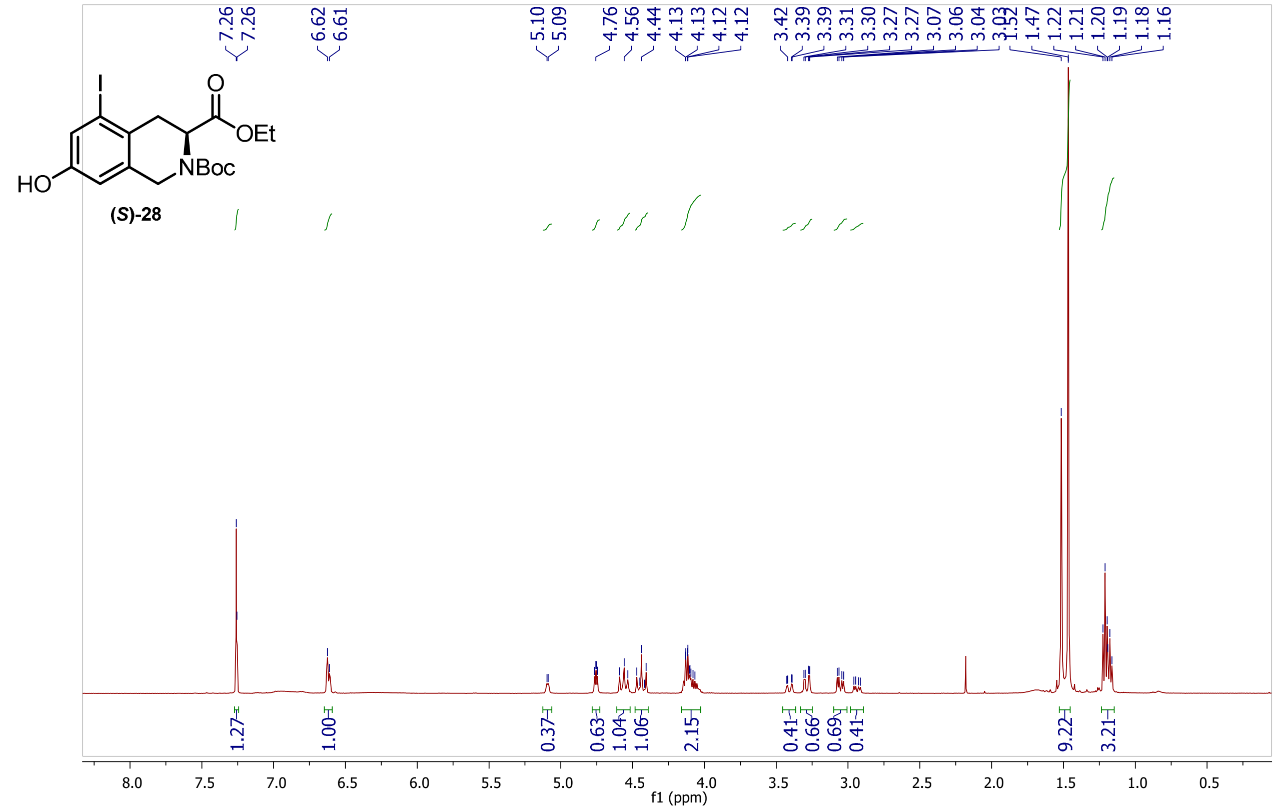

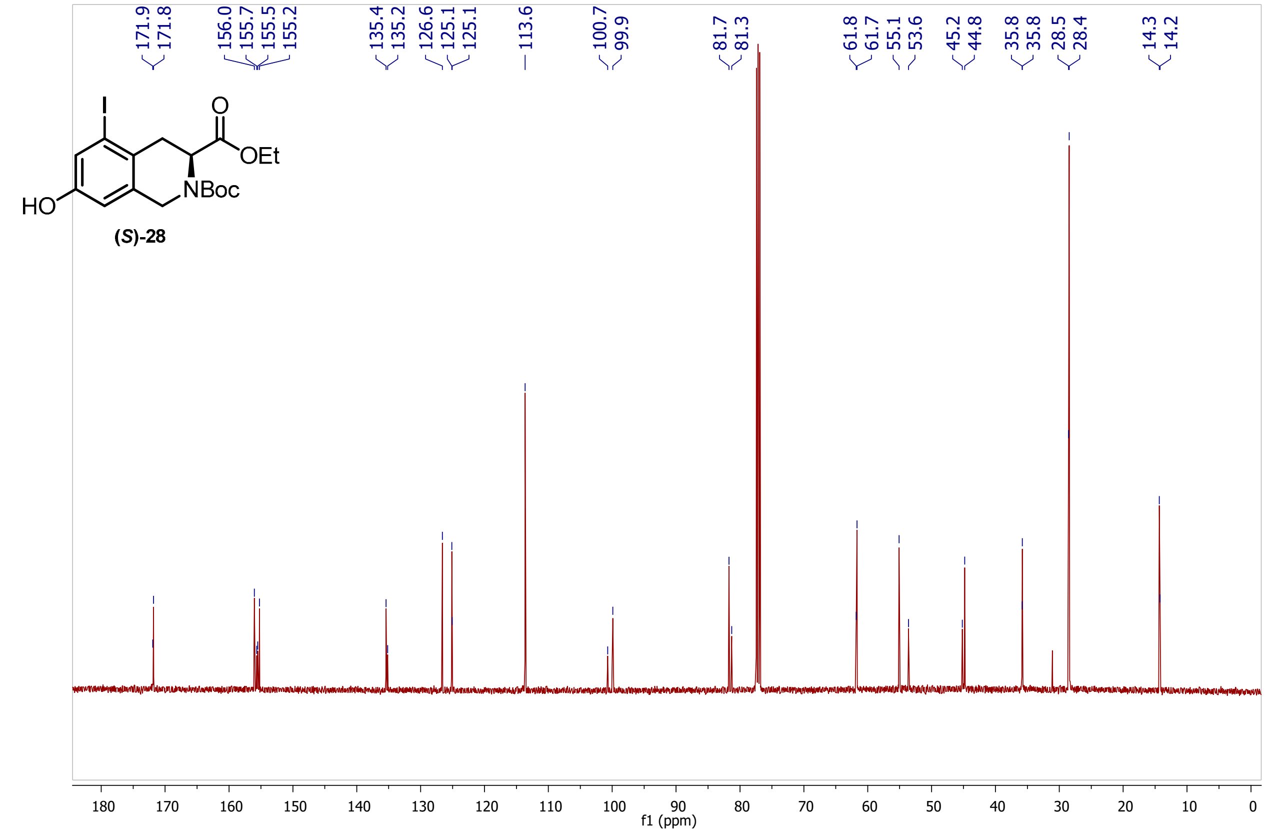

^{1}H (top) and ^{13}C (down) NMR spectra of compound **(*R*)-28** in CDCl₃.

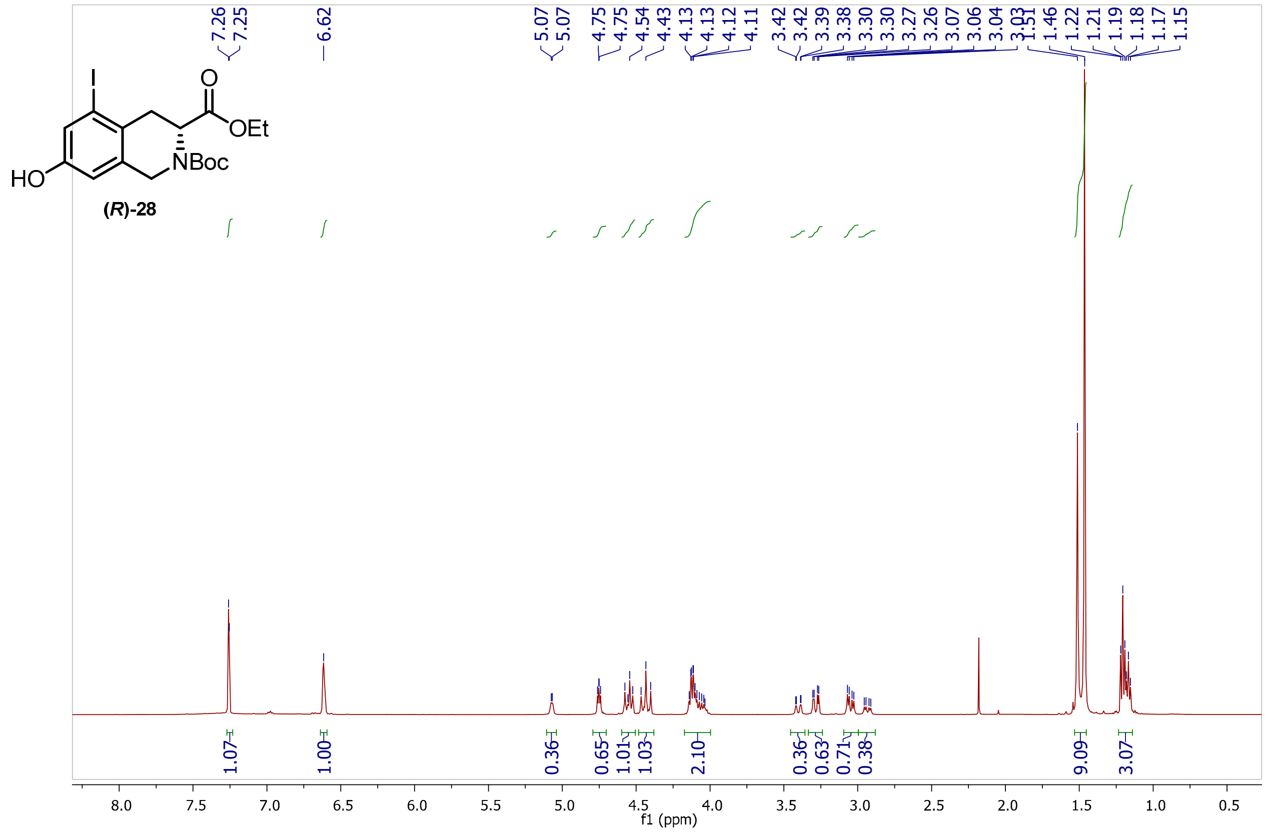

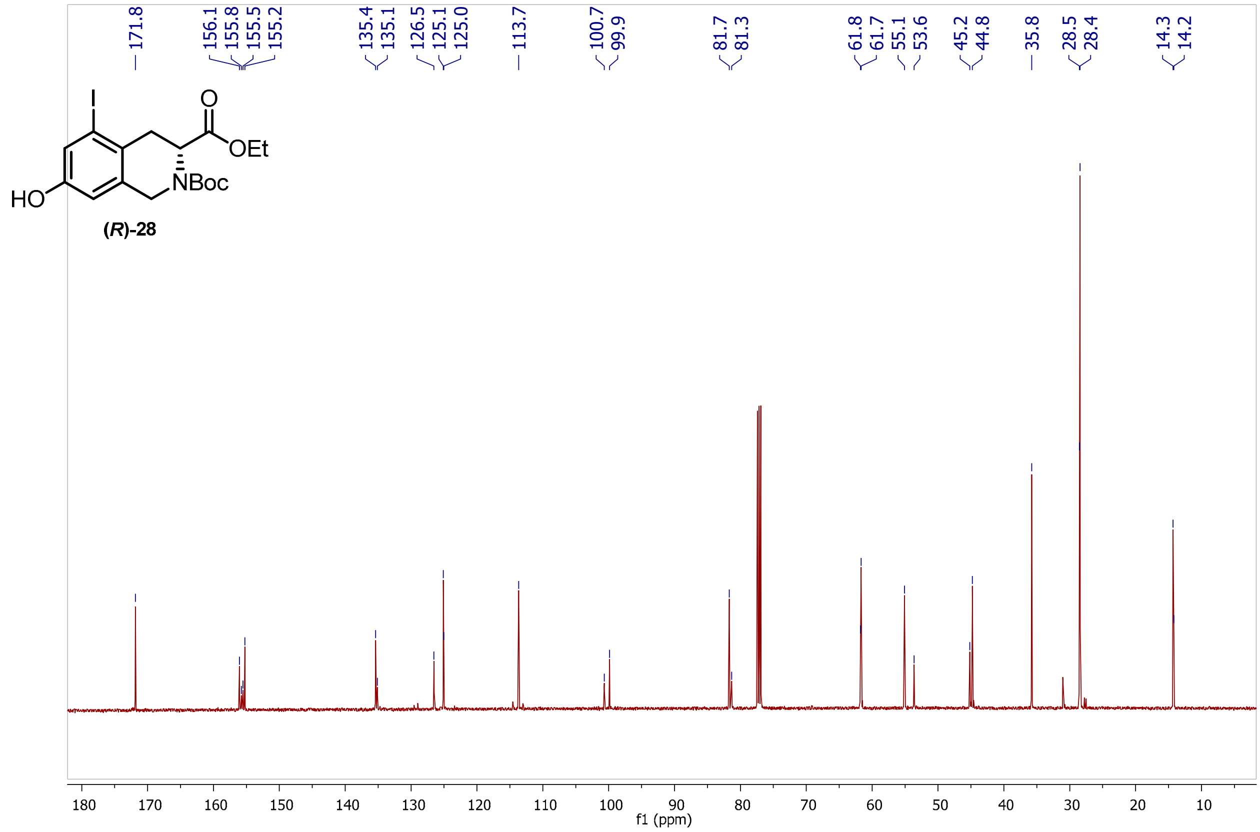

^{1}H (top) and ^{13}C (down) NMR spectra of compound ***(S)*-29** in $CDCl_3$.

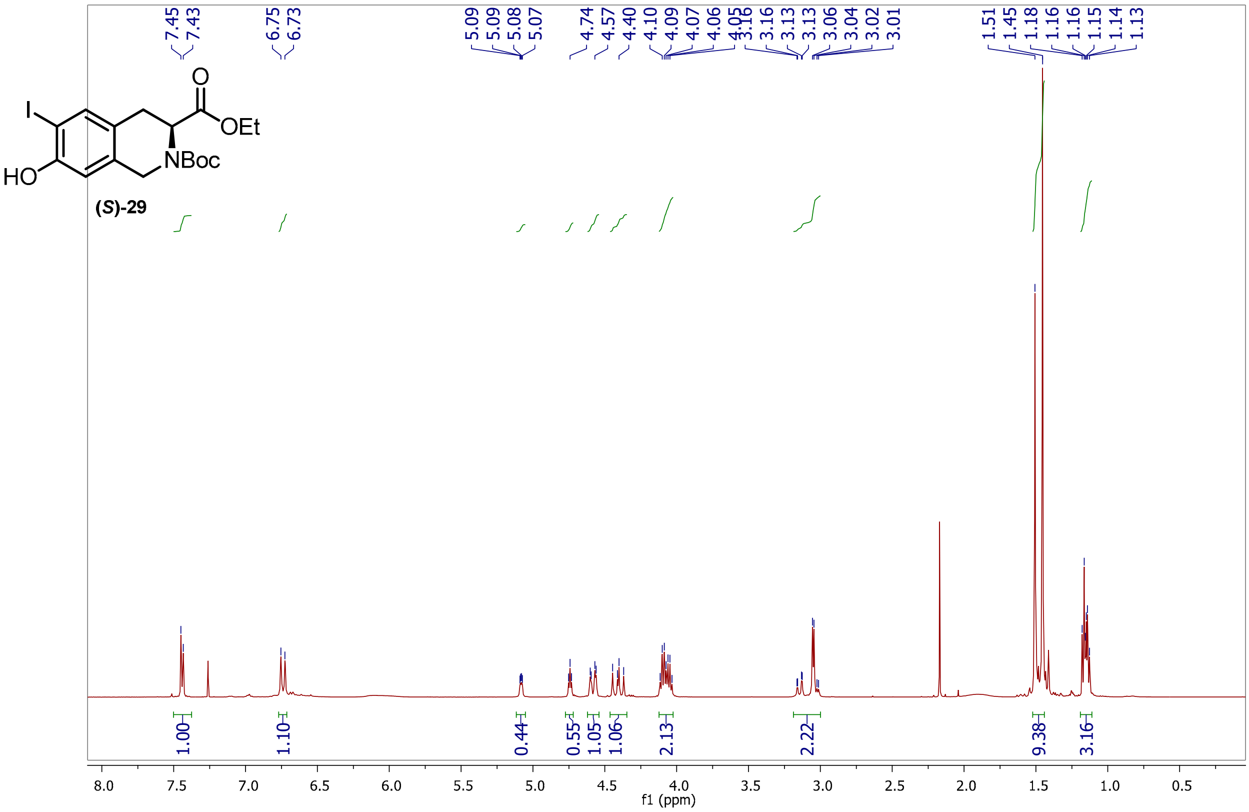

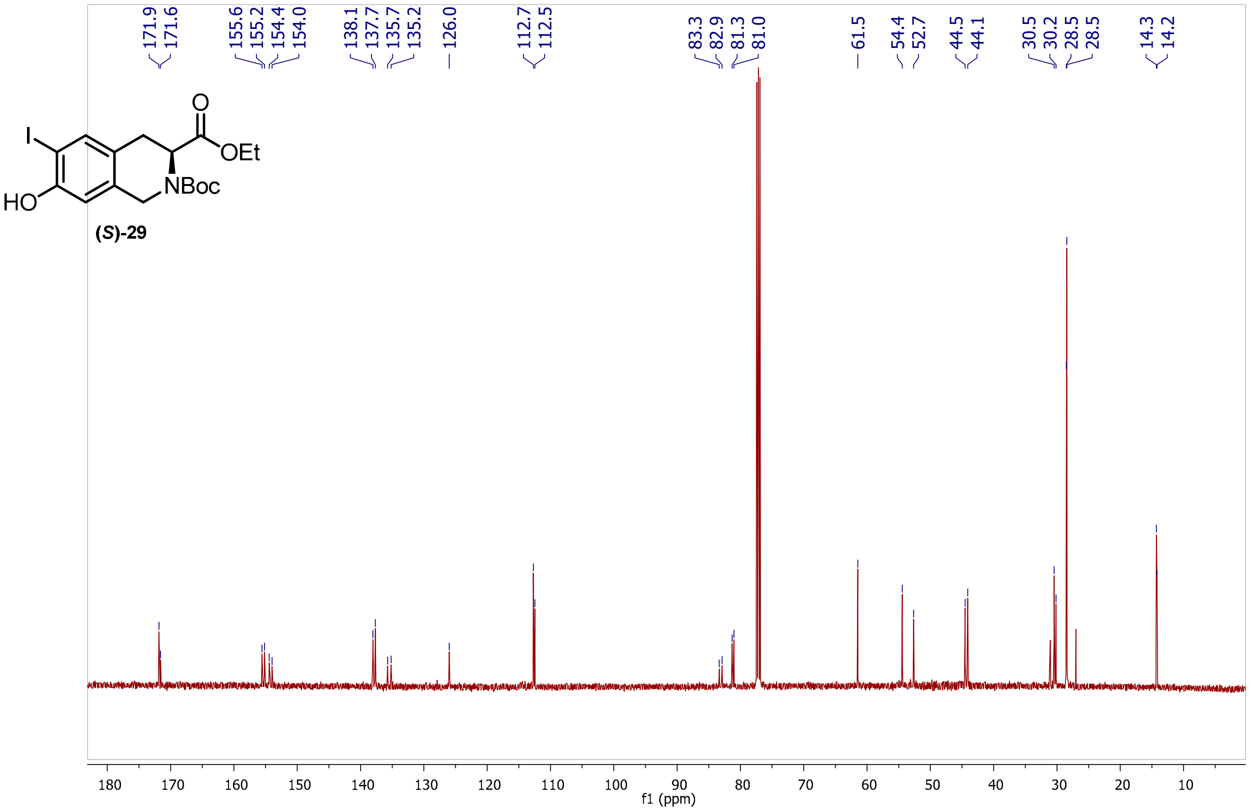

^{1}H (top) and ^{13}C (down) NMR spectra of compound ***(R)*-29** in $CDCl_3$.

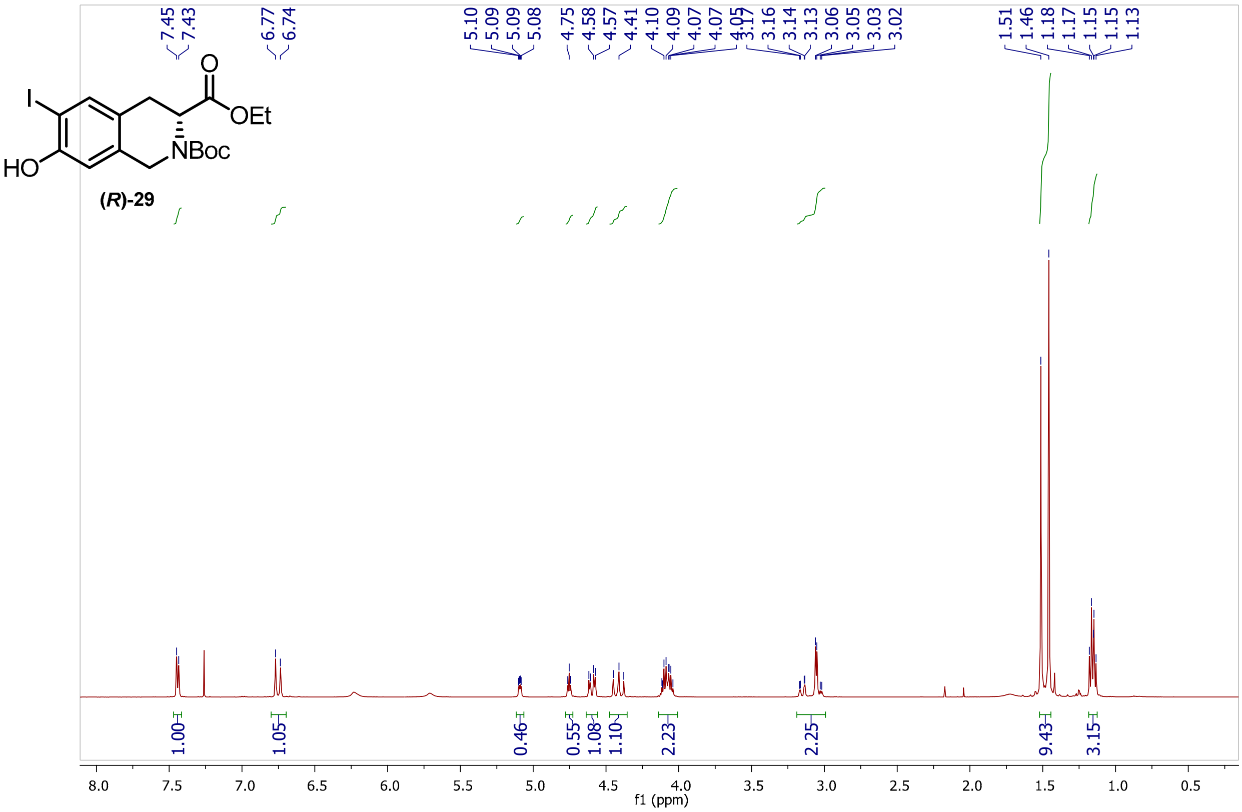

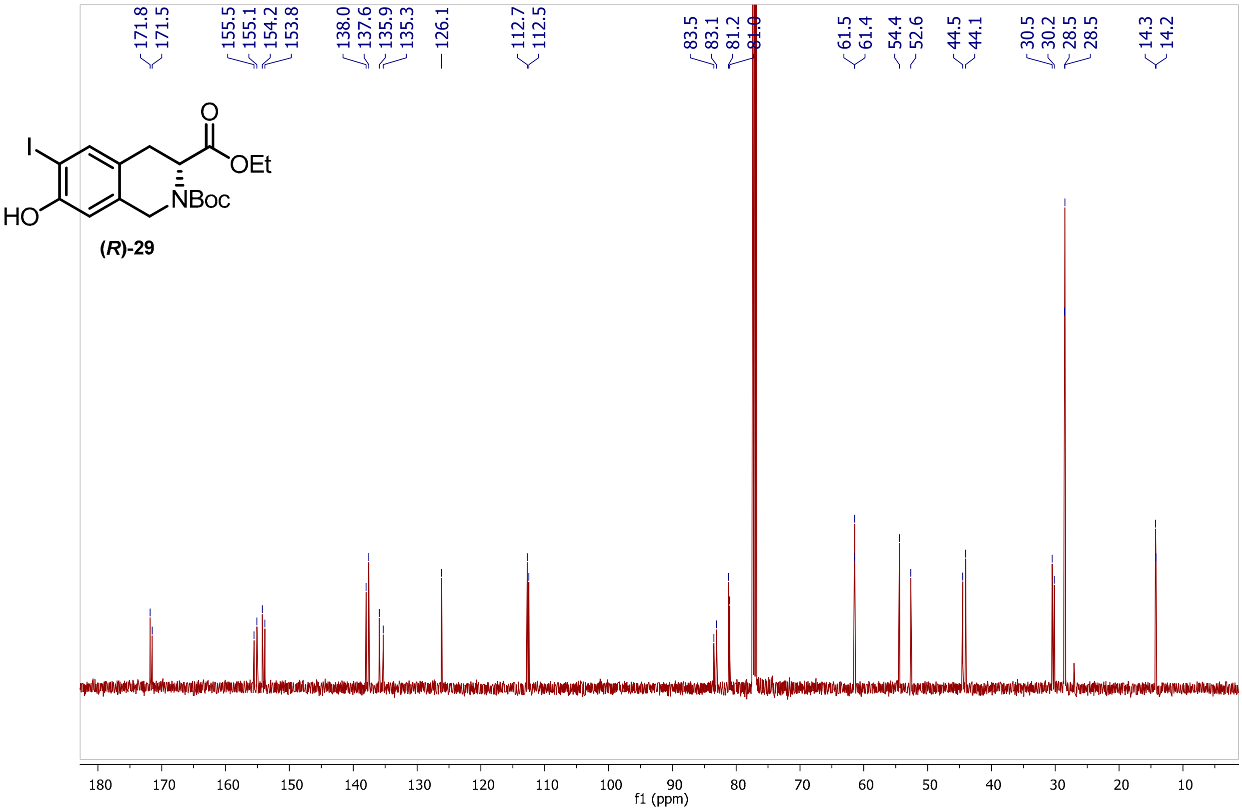

^{1}H (top) and ^{13}C (down) NMR spectra of compound **(*S*)-30** in $CDCl_3$.

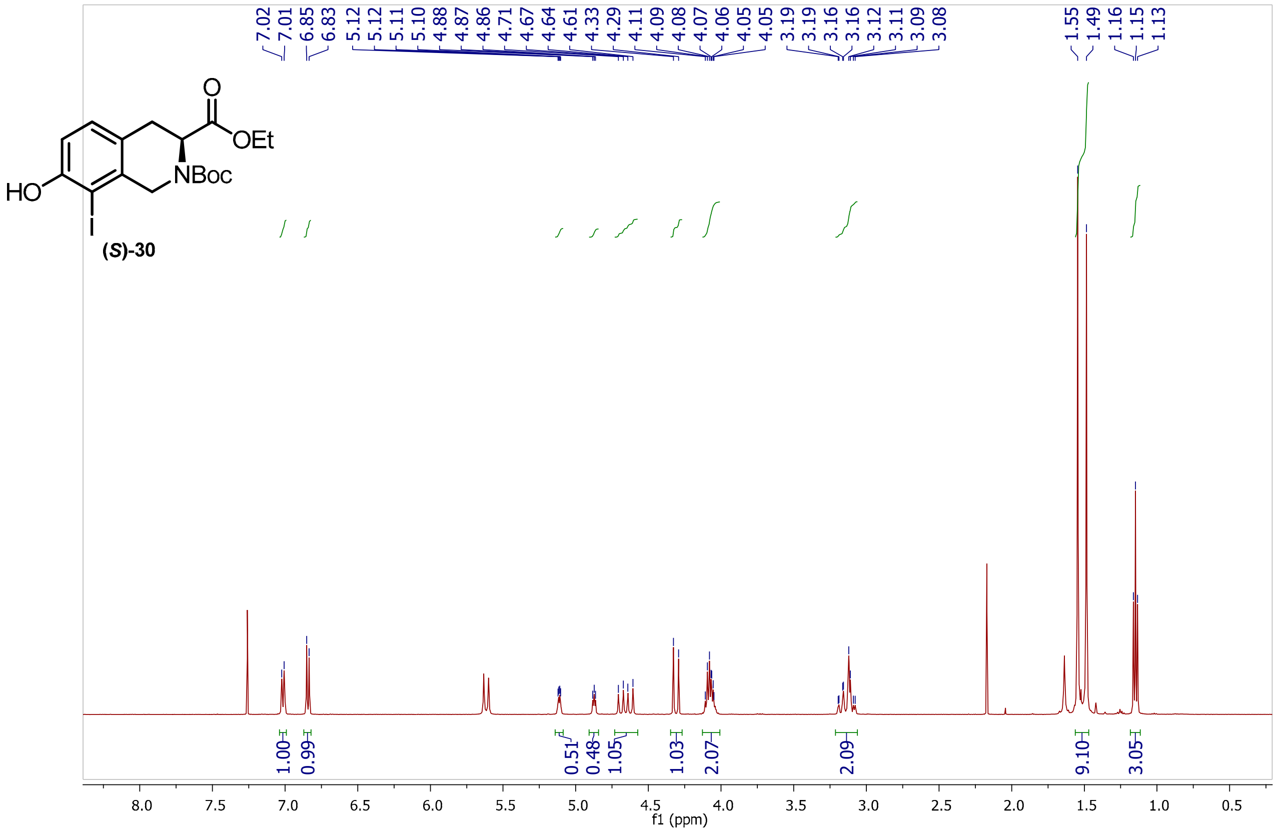

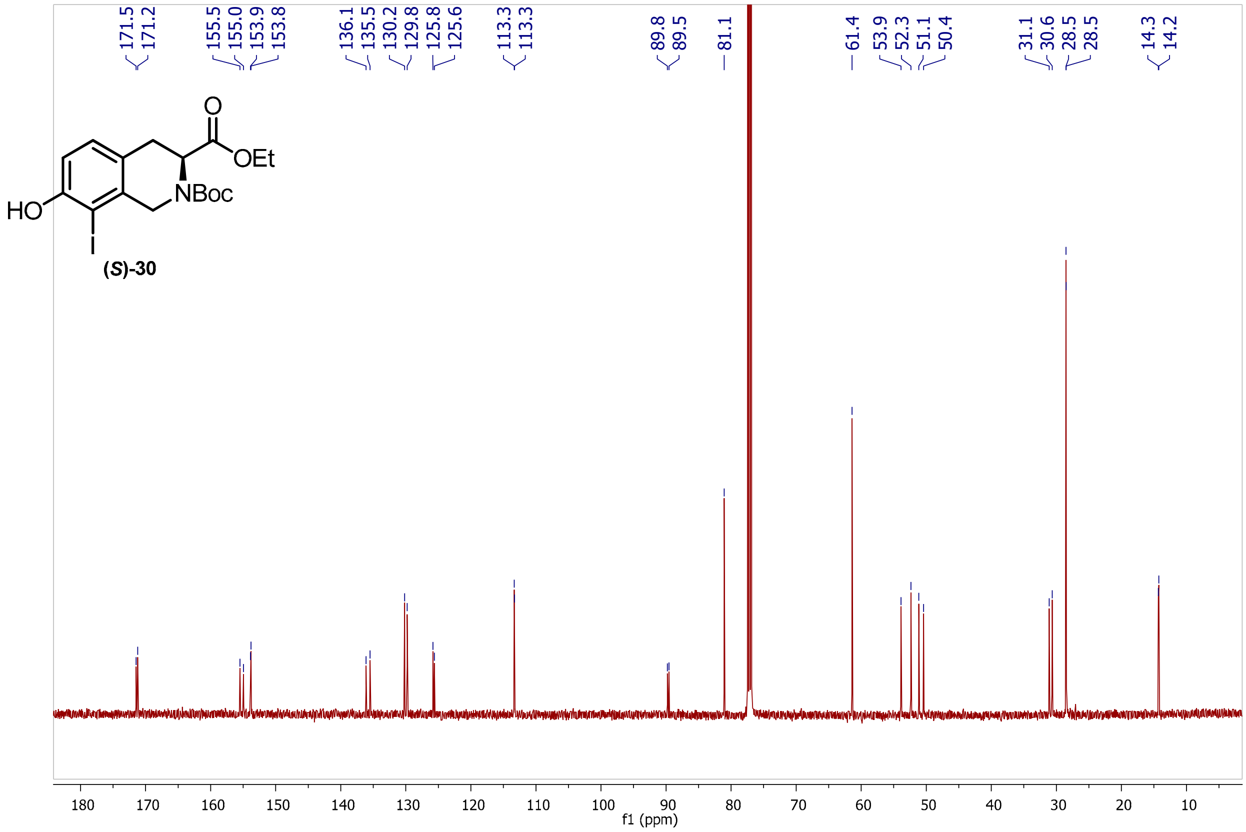

^{1}H (top) and ^{13}C (down) NMR spectra of compound **(*R*)-30** in $CDCl_3$.

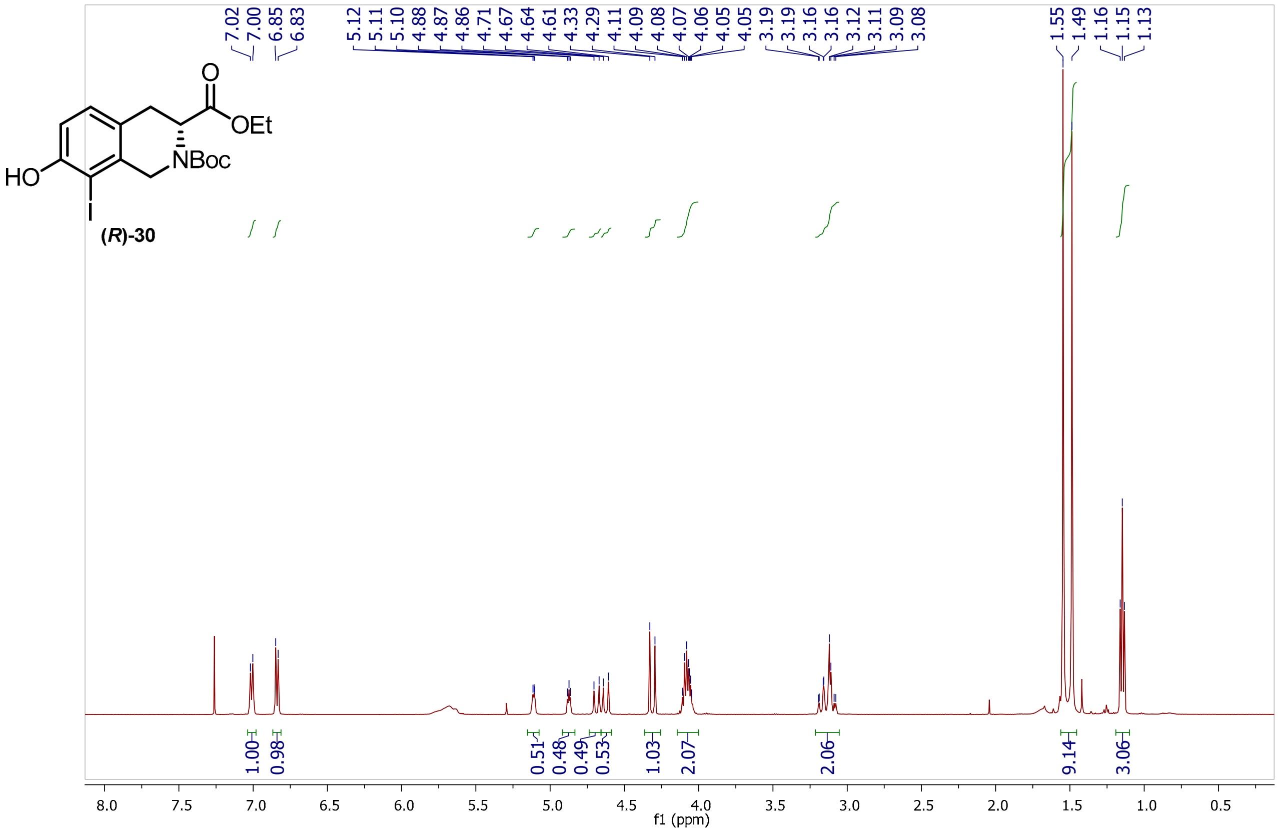

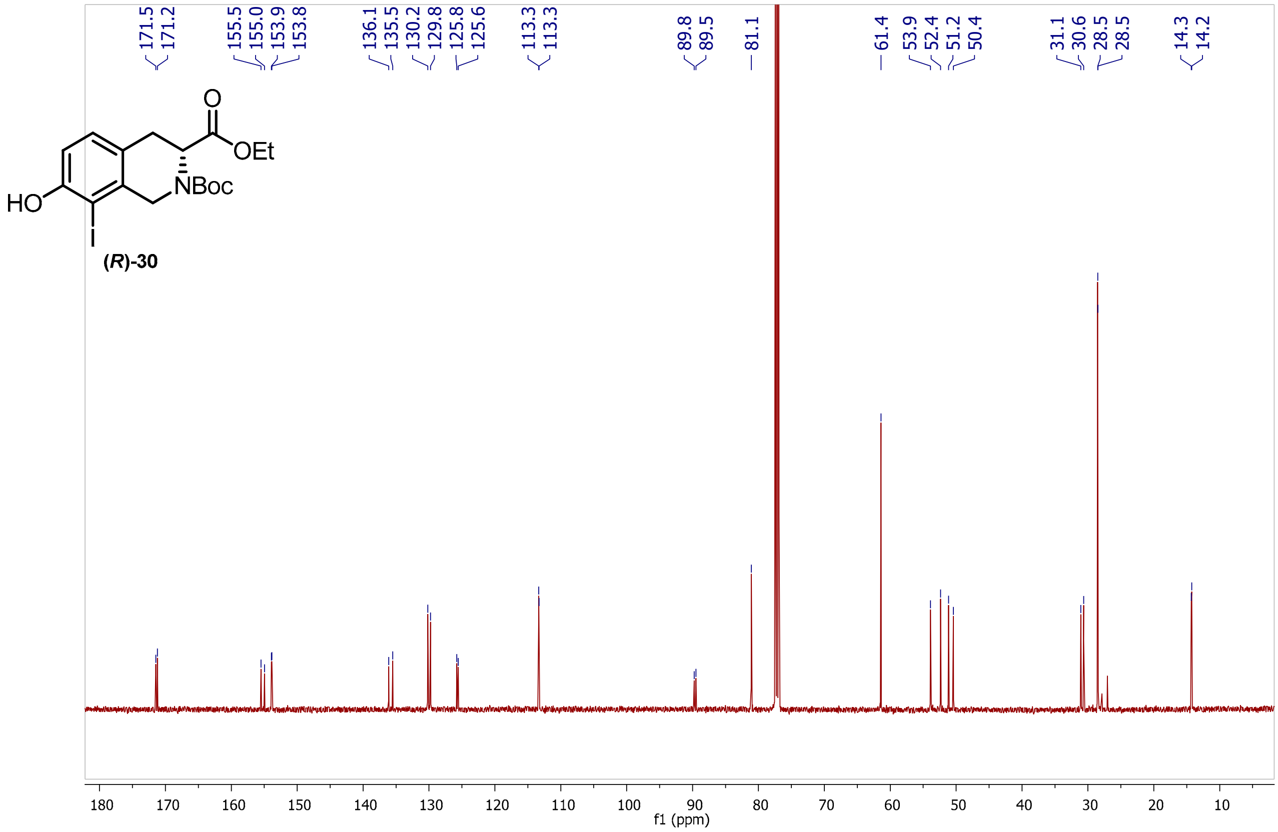

^{1}H (top) and ^{13}C (down) NMR spectra of compound **(*S*)-31** in $CDCl_3$.

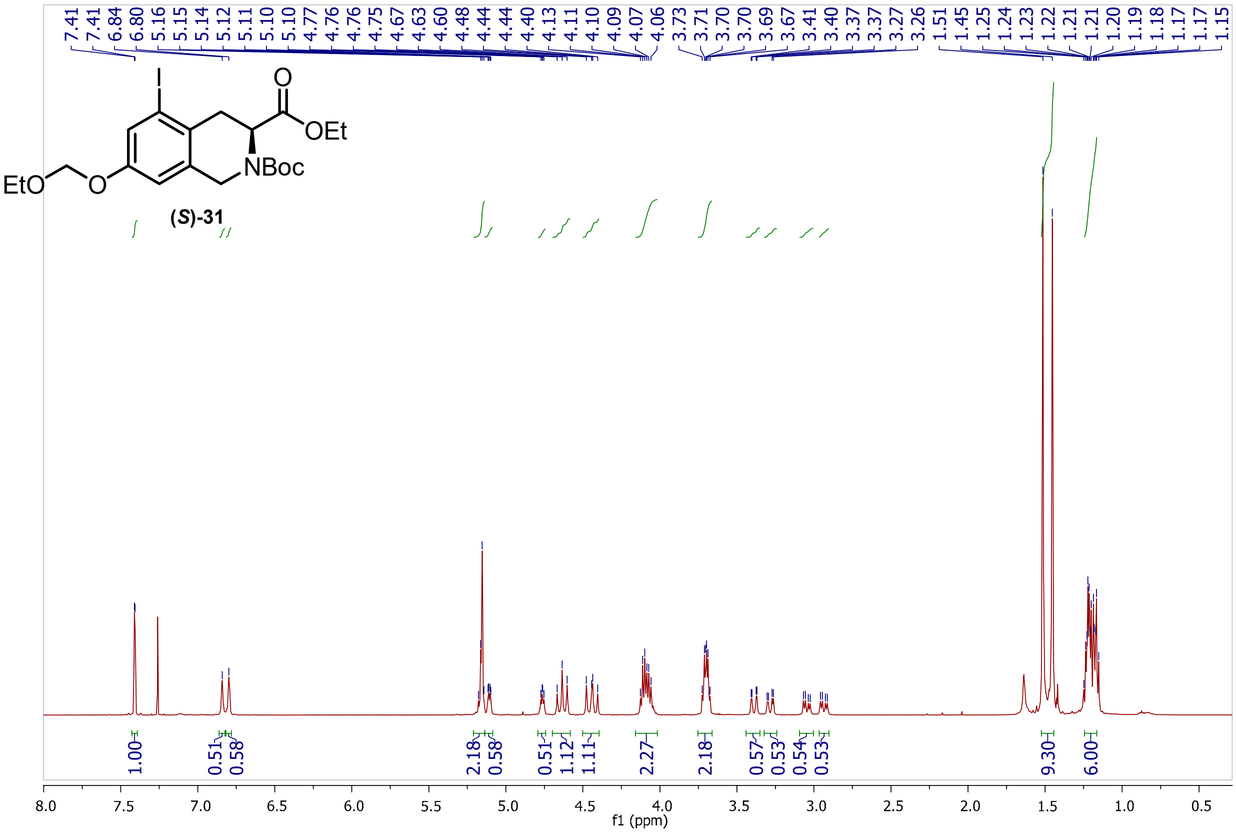

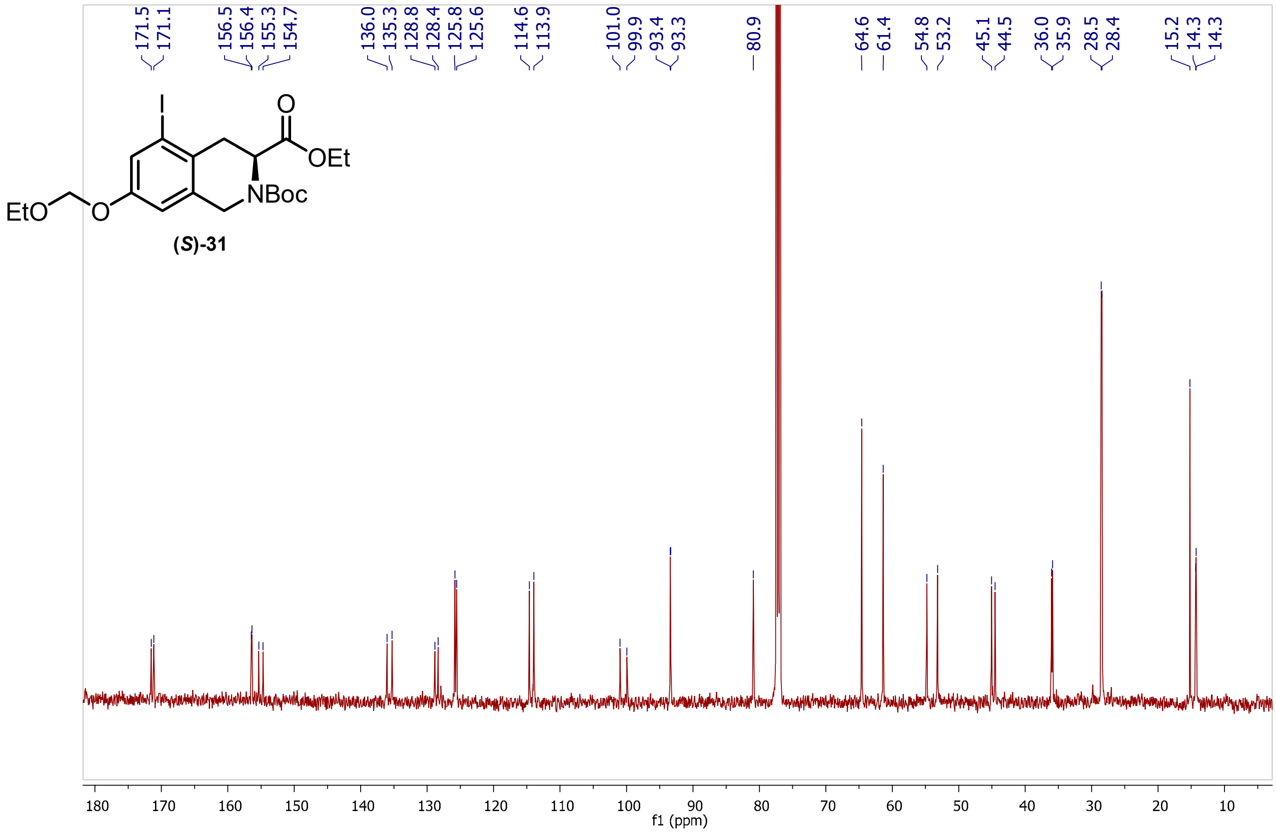

^{1}H (top) and ^{13}C (down) NMR spectra of compound **(*R*)-31** in $CDCl_3$.

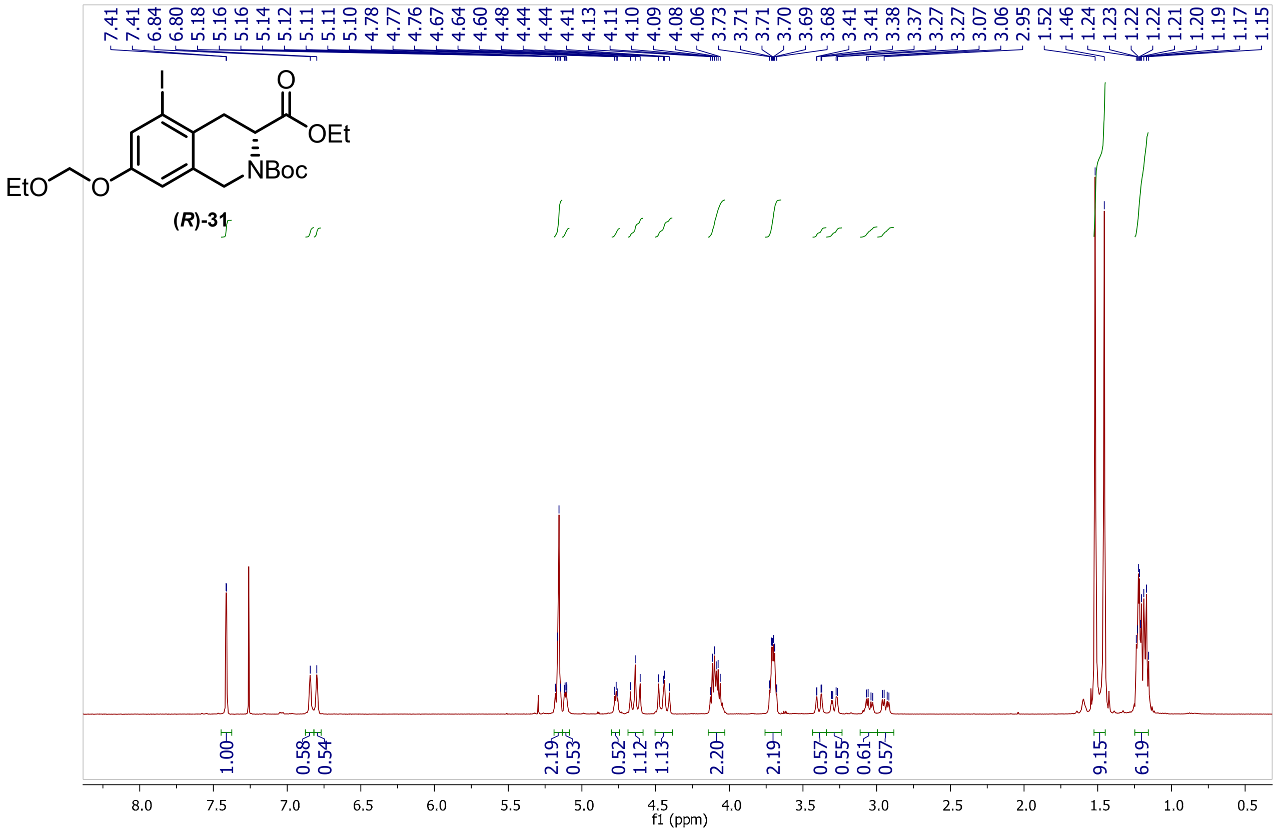

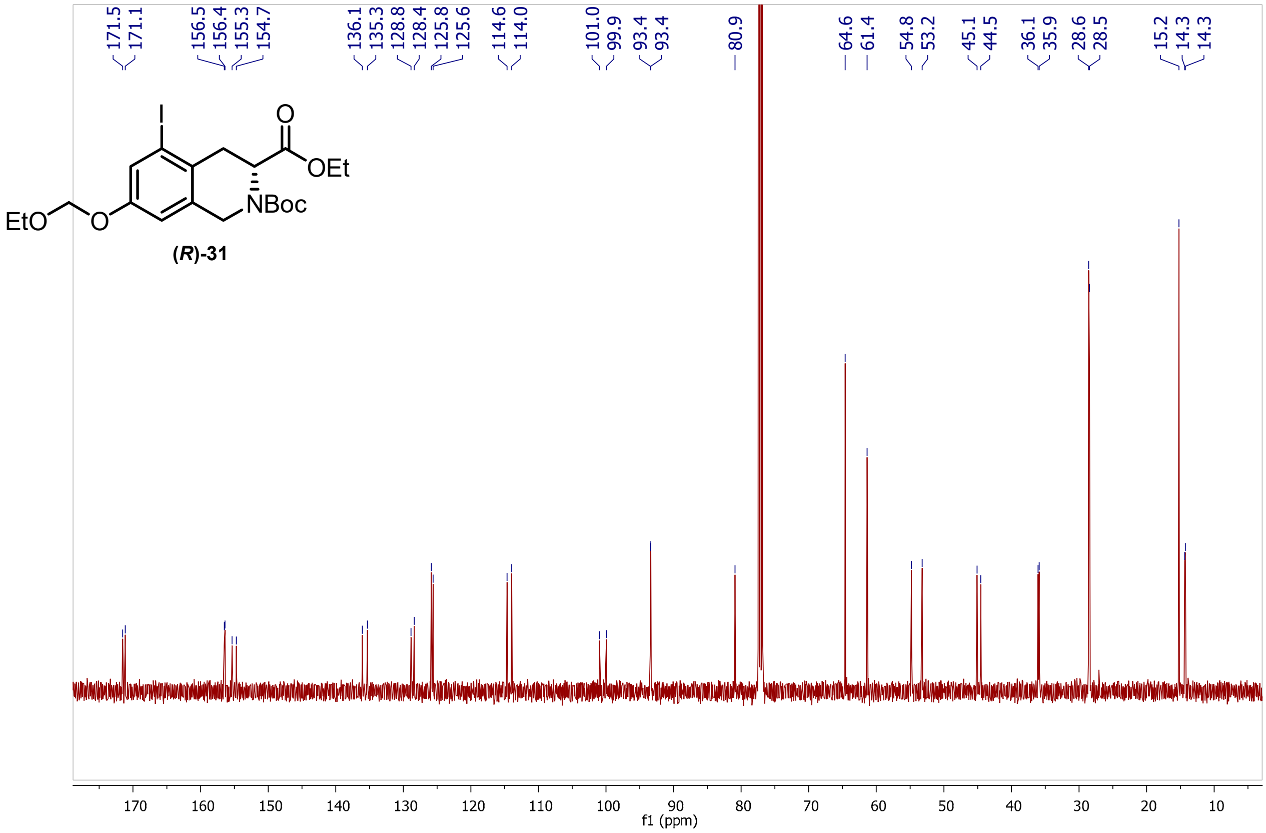

^{1}H (top) and ^{13}C (down) NMR spectra of compound **(*S*)-32** in CDCl₃.

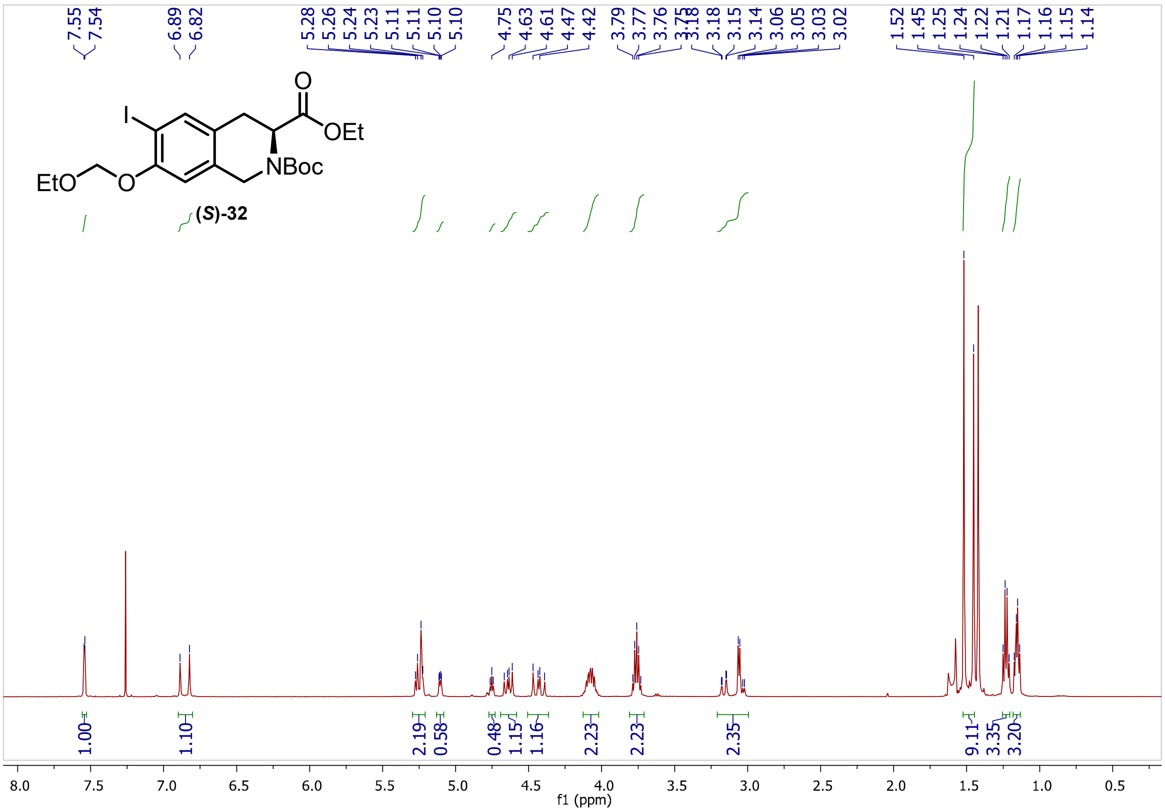

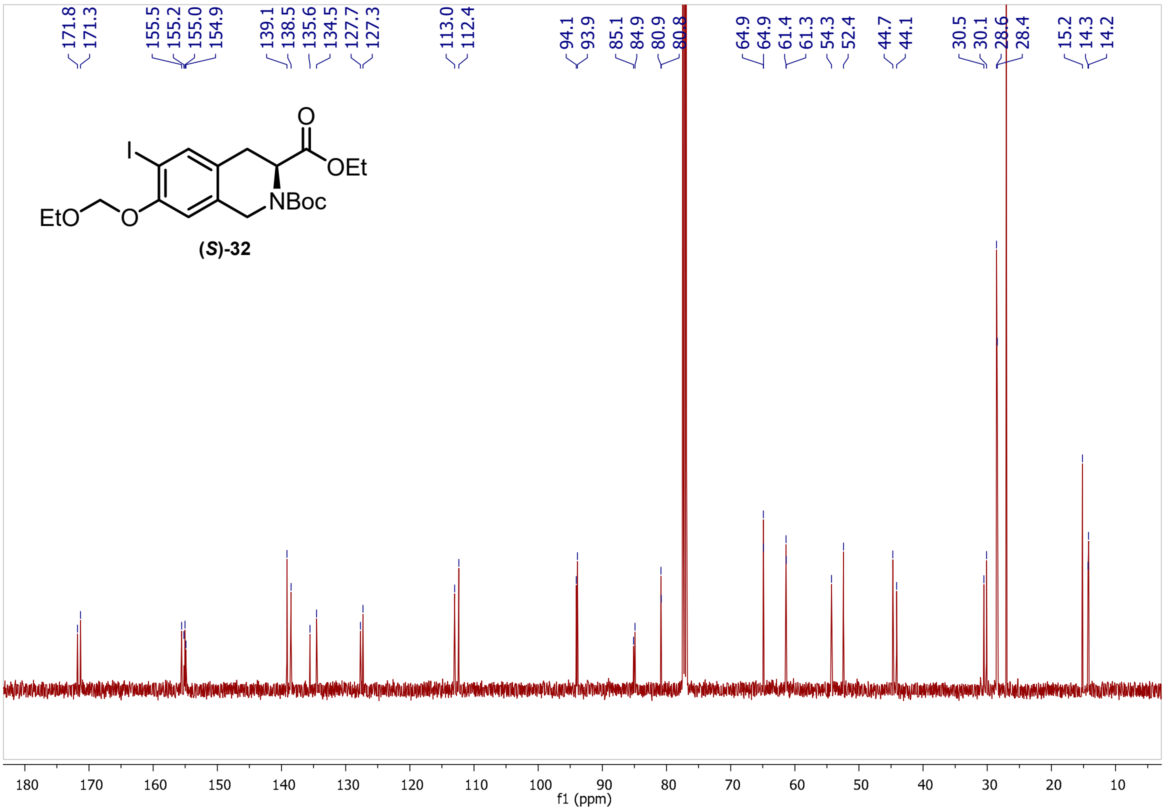

^{1}H (top) and ^{13}C (down) NMR spectra of compound **(*R*)-32** in $CDCl_3$.

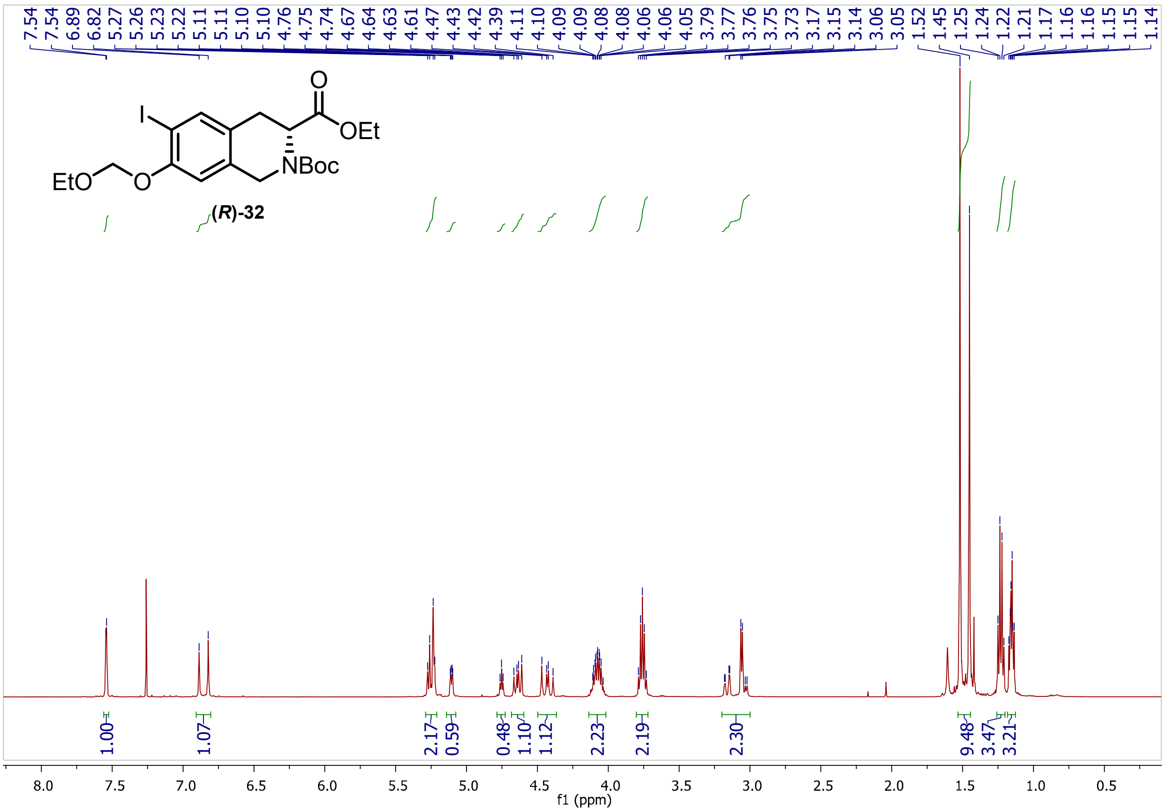

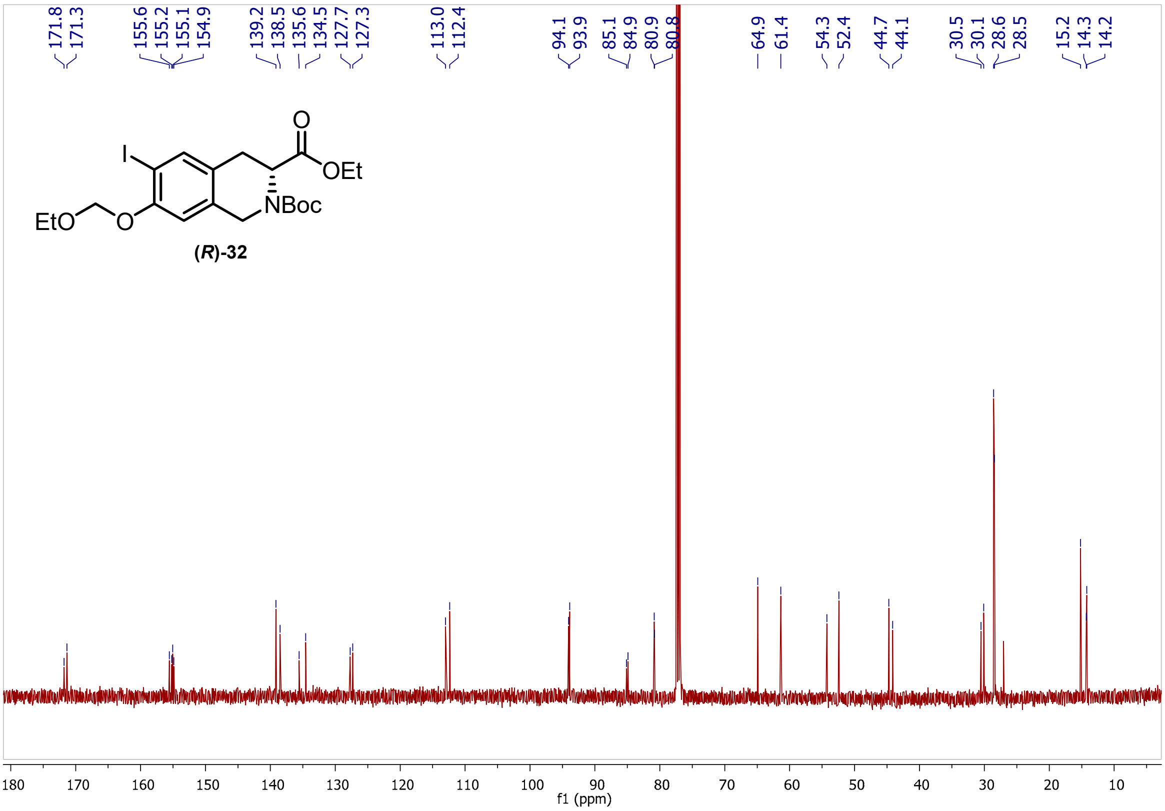

^{1}H (top) and ^{13}C (down) NMR spectra of compound **(*S*)-33** in $CDCl_3$.

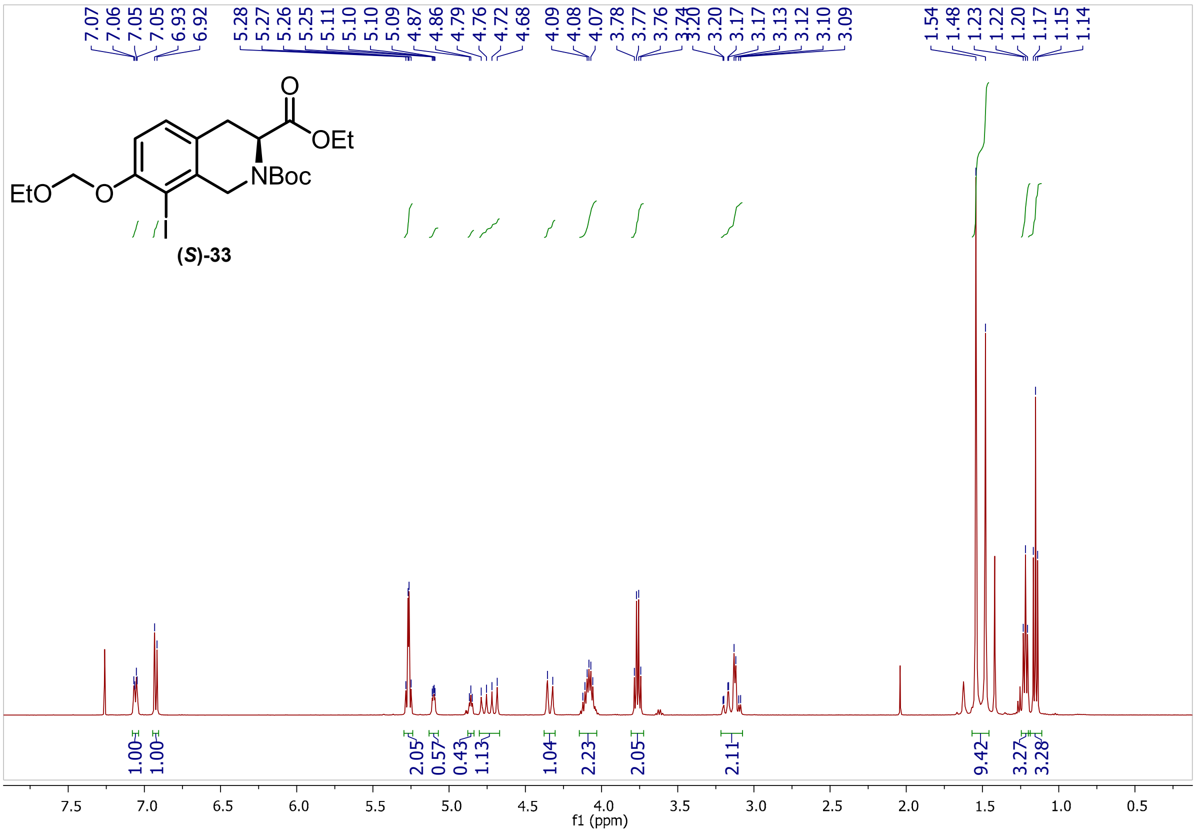

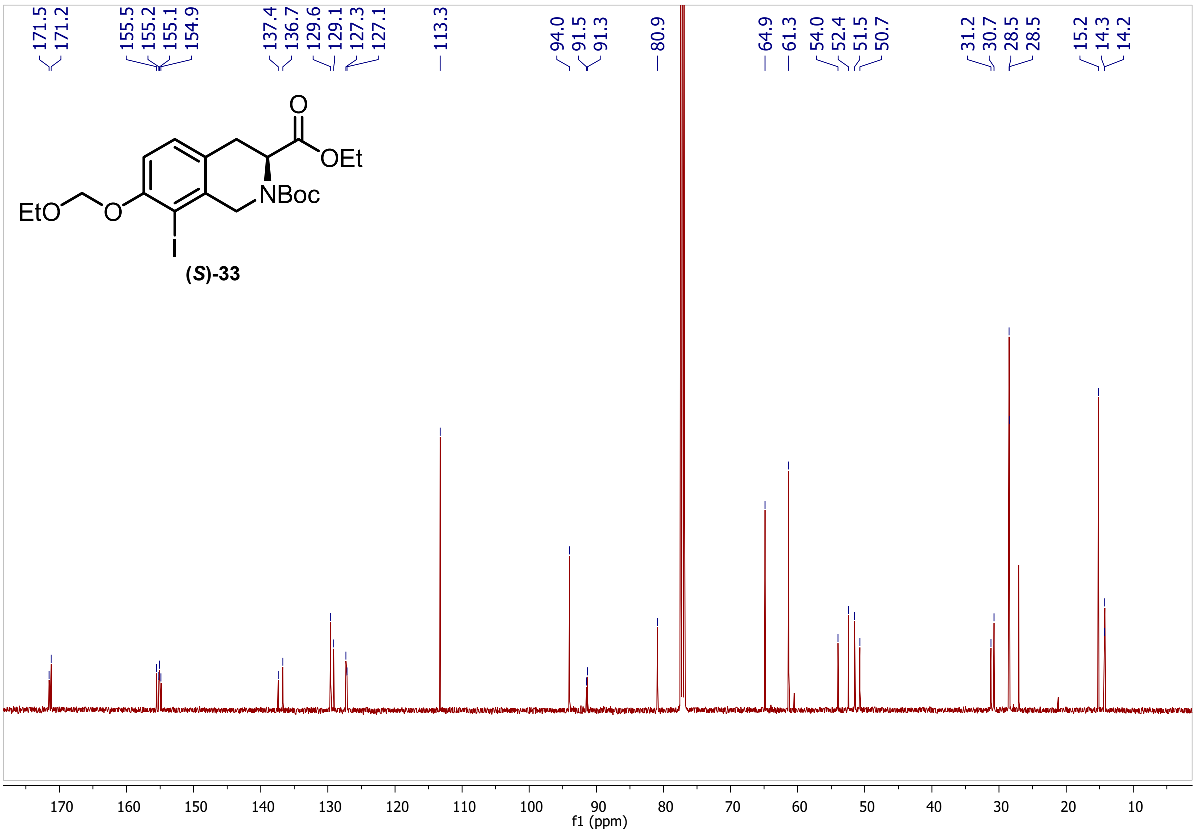

^{1}H (top) and ^{13}C (down) NMR spectra of compound ***(R)*-33** in $CDCl_3$.

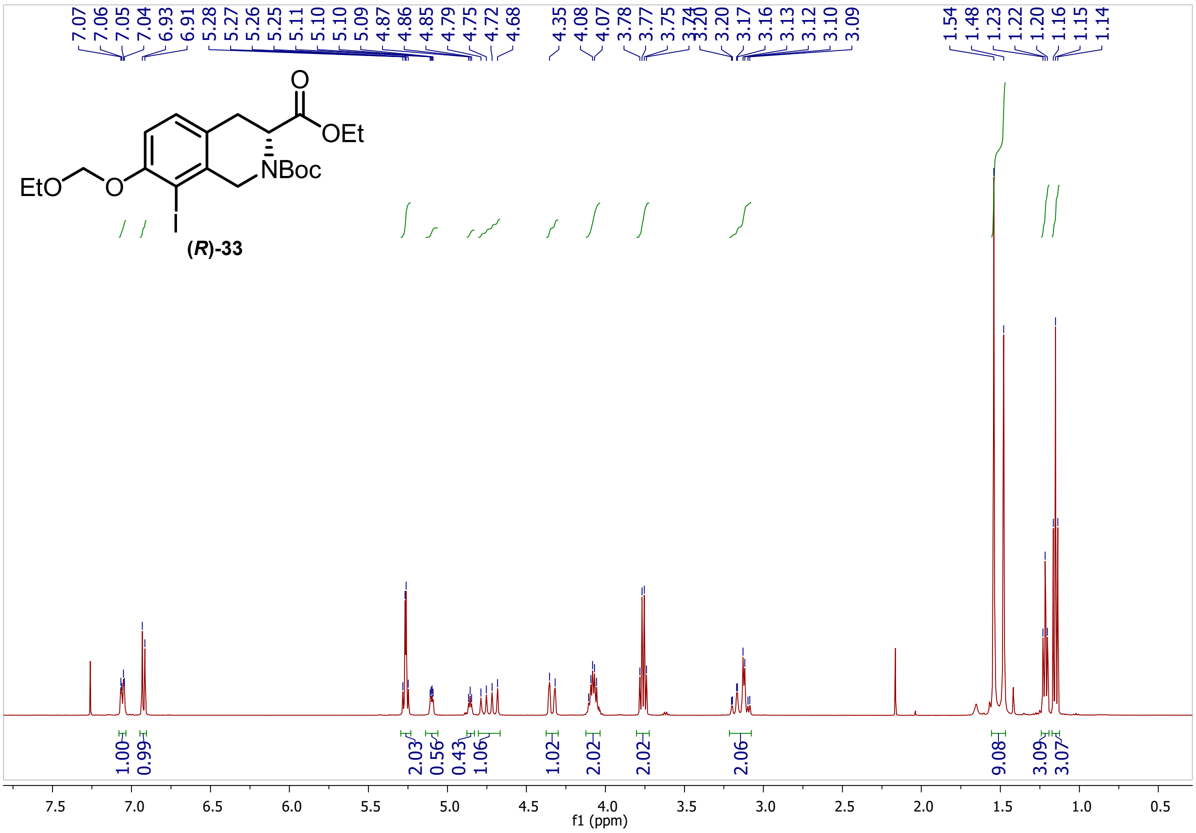

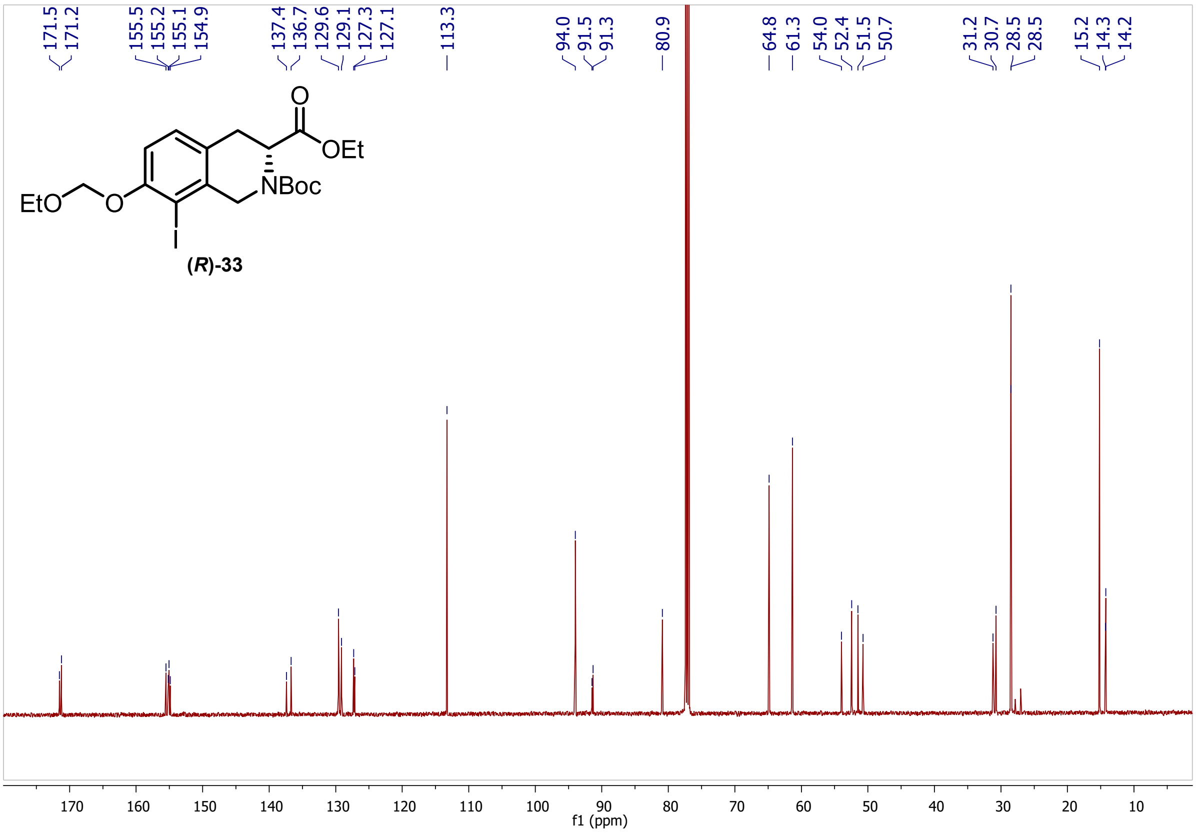

^{1}H (top) and ^{13}C (down) NMR spectra of compound **(*S*)-34** in $CDCl_3$.

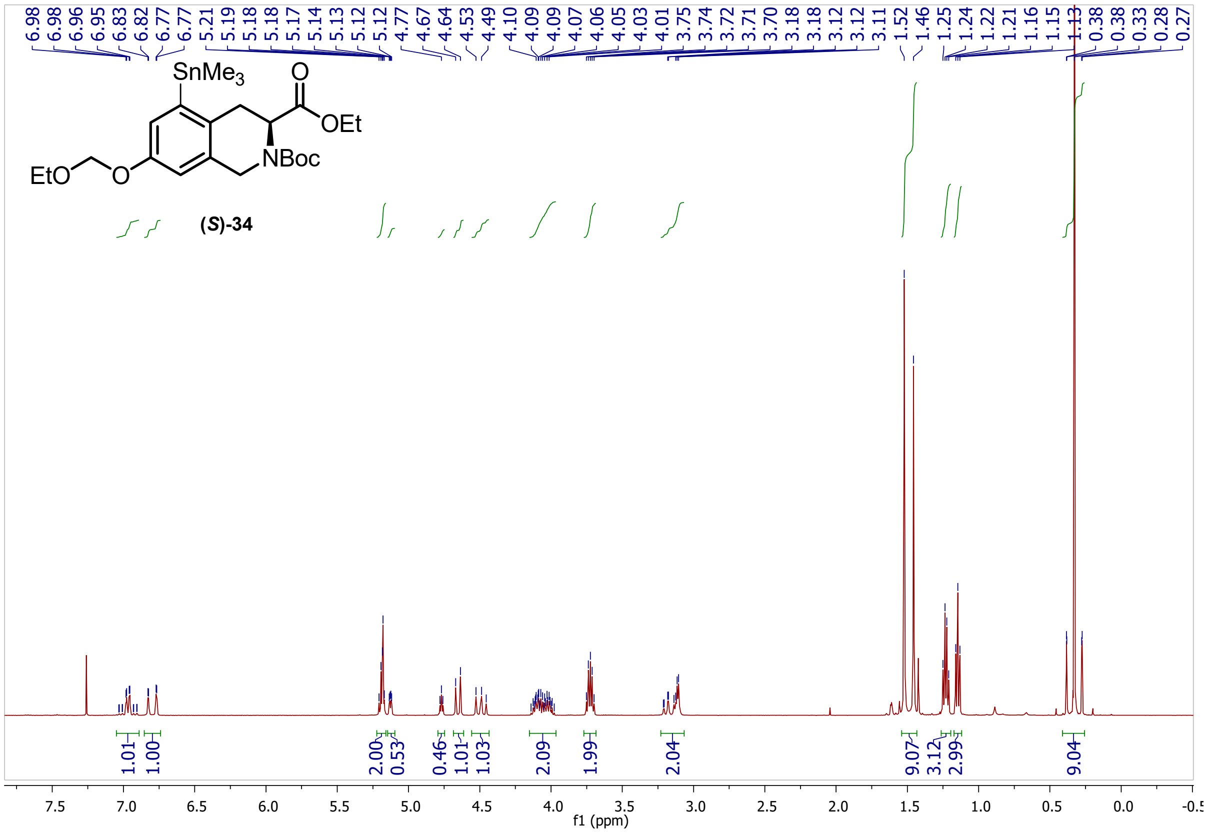

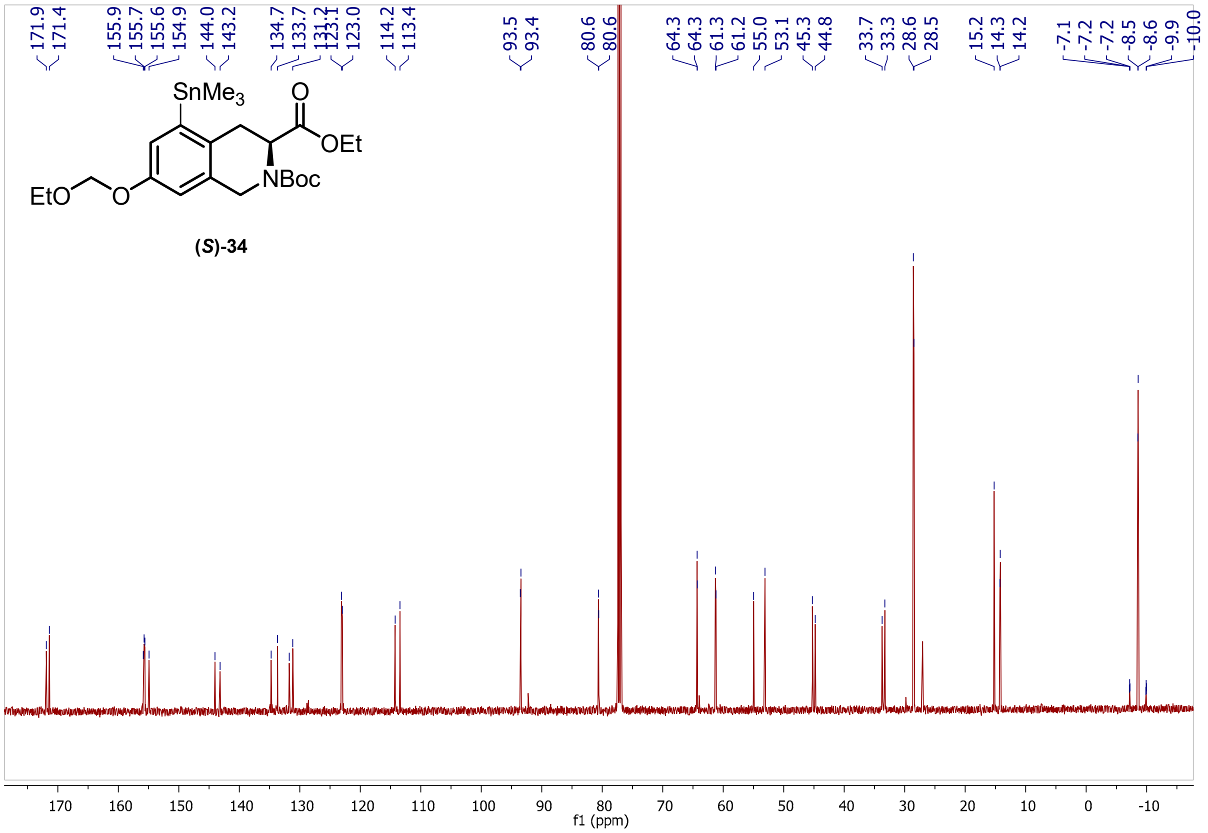

^{1}H (top) and ^{13}C (down) NMR spectra of compound **(*R*)-34** in CDCl3.

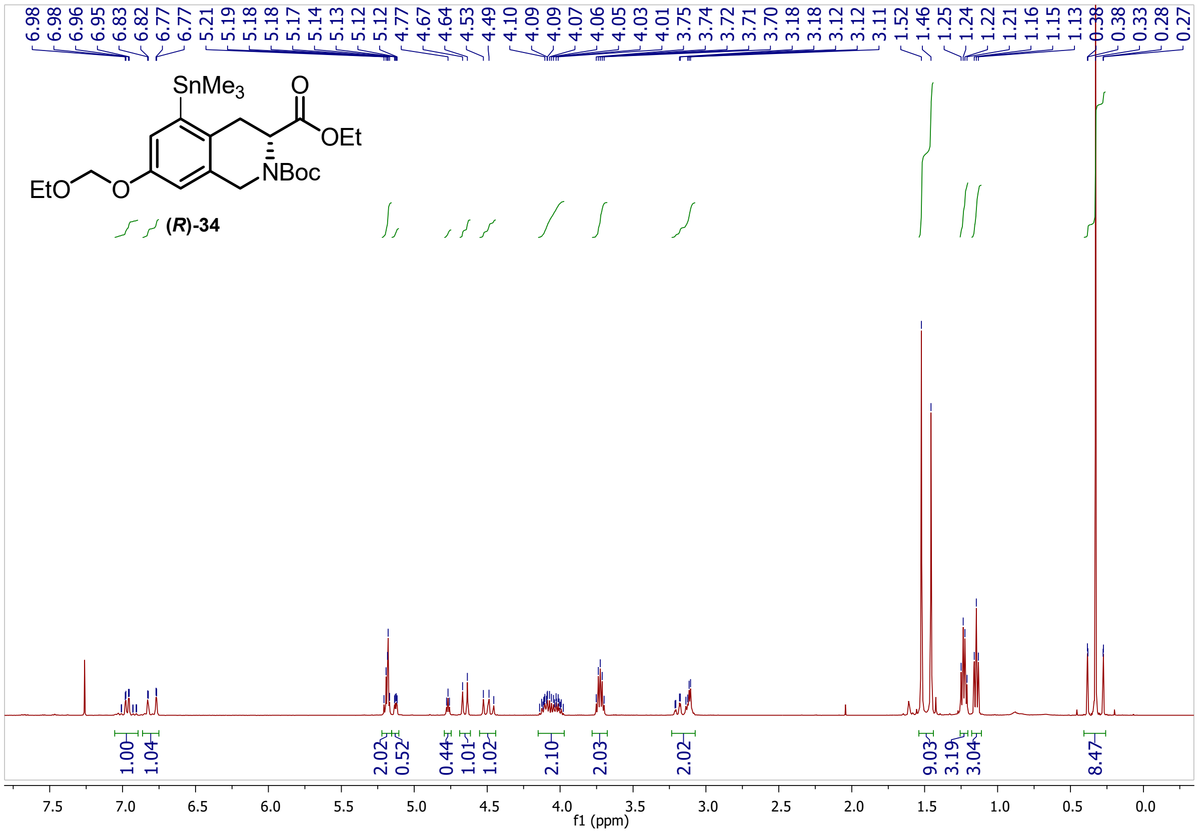

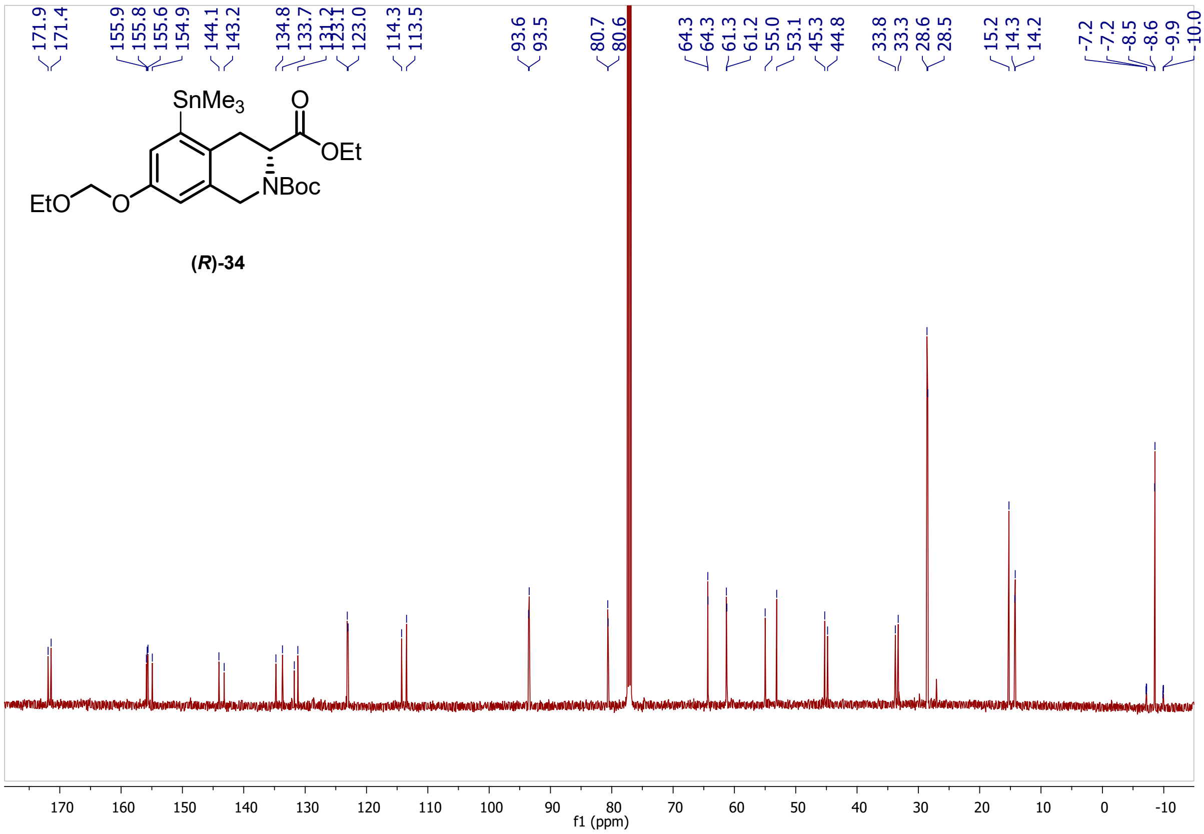

^{1}H (top) and ^{13}C (down) NMR spectra of compound **(*S*)-35** in $CDCl_3$.

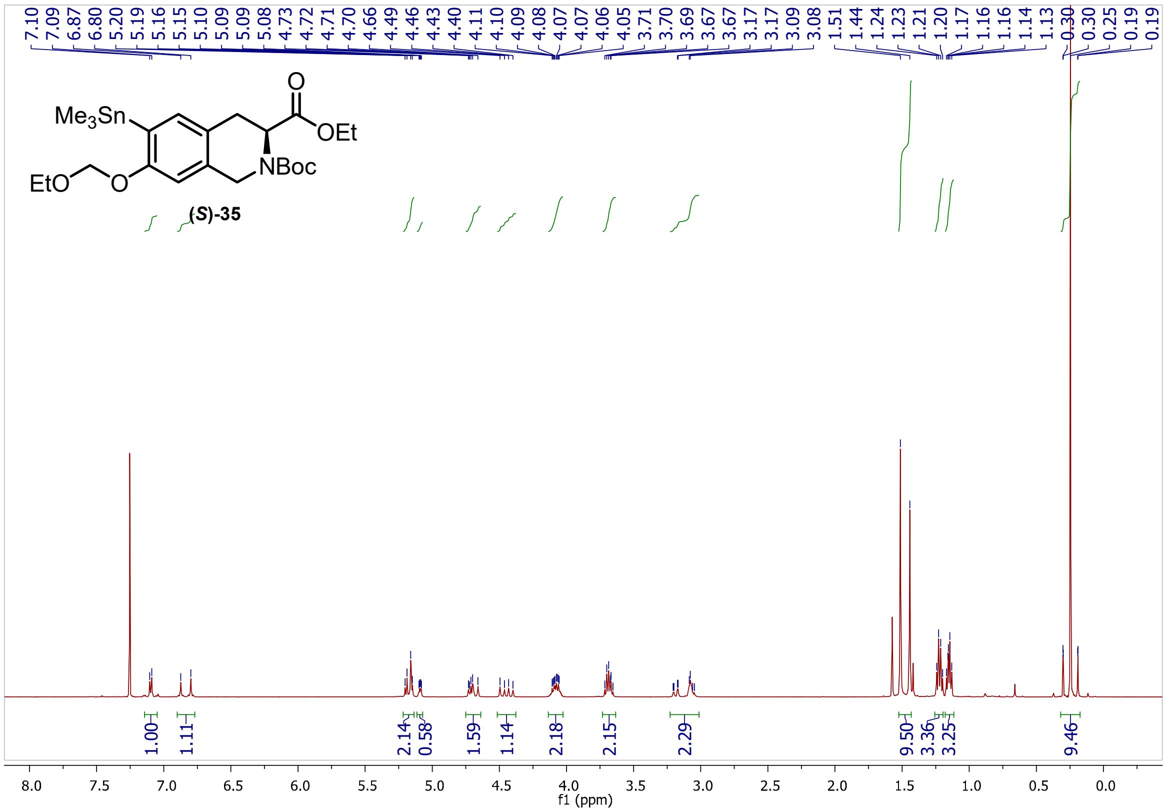

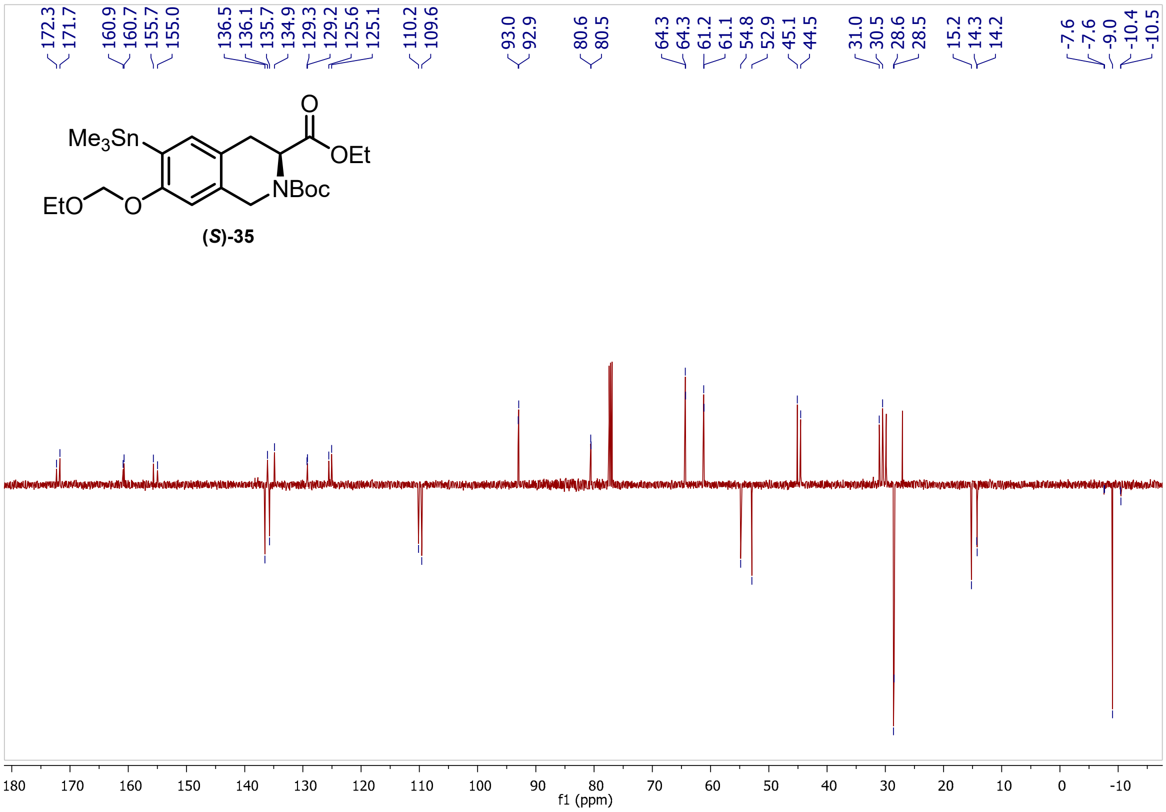

^{1}H (top) and ^{13}C (down) NMR spectra of compound ***(R)*-35** in $CDCl_3$.

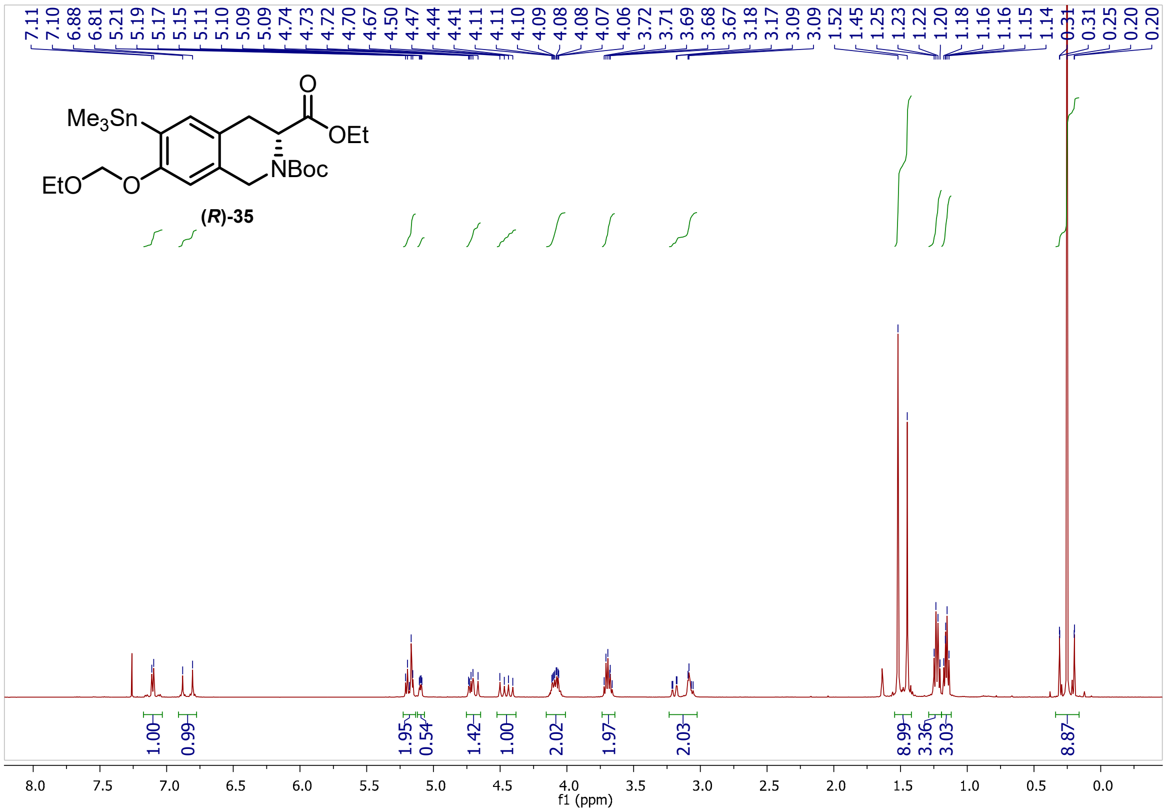

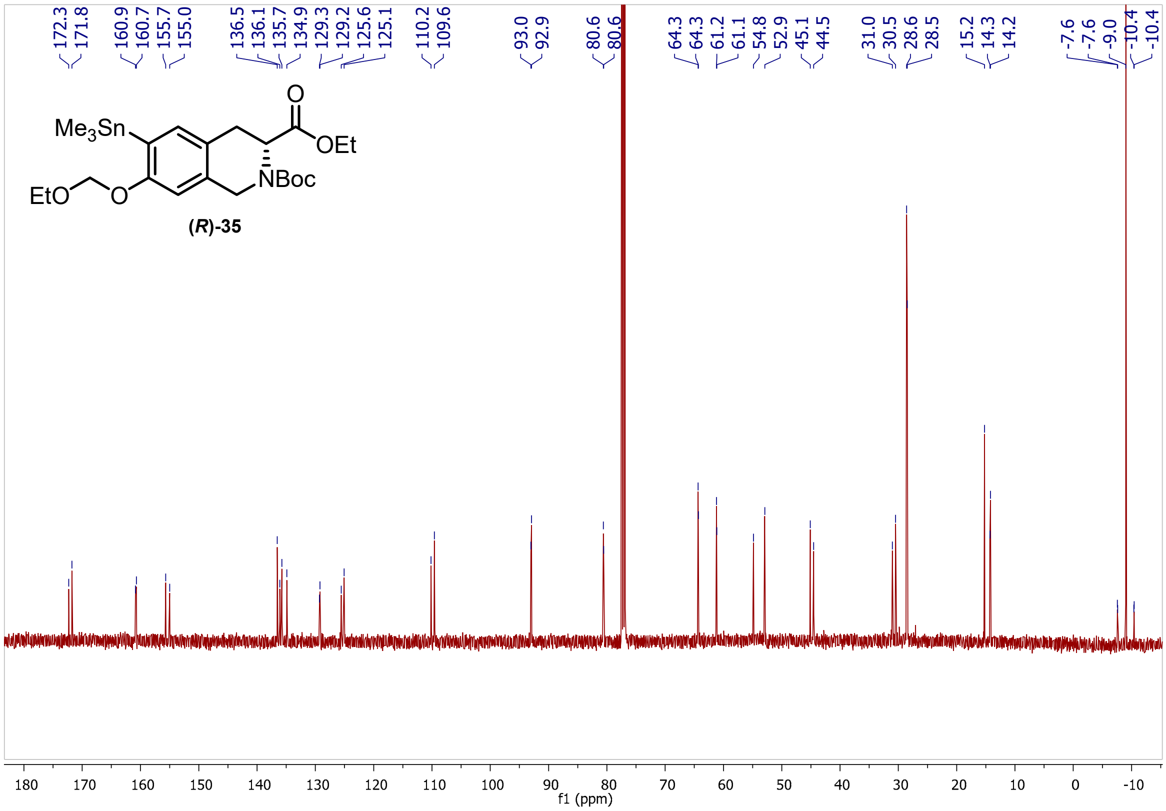

^{1}H (top) and ^{13}C (down) NMR spectra of compound ***(S)*-36** in $CDCl_3$.

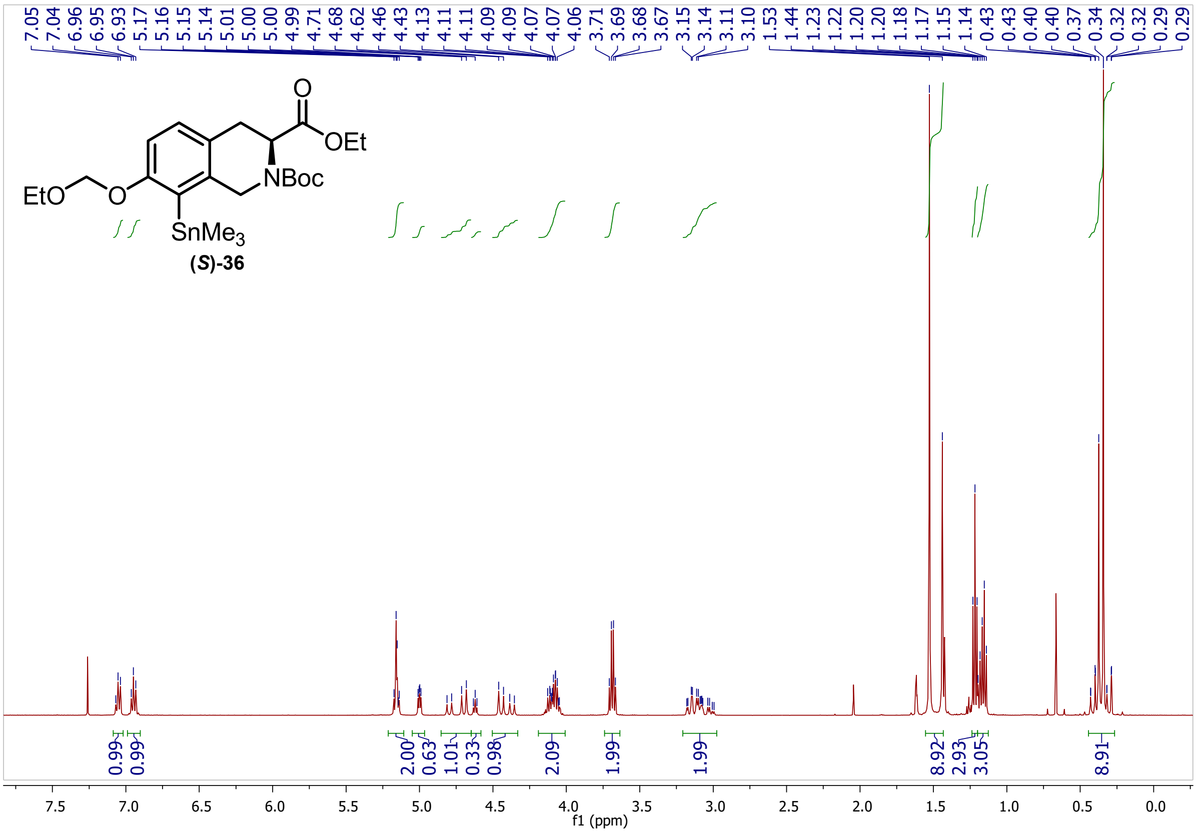

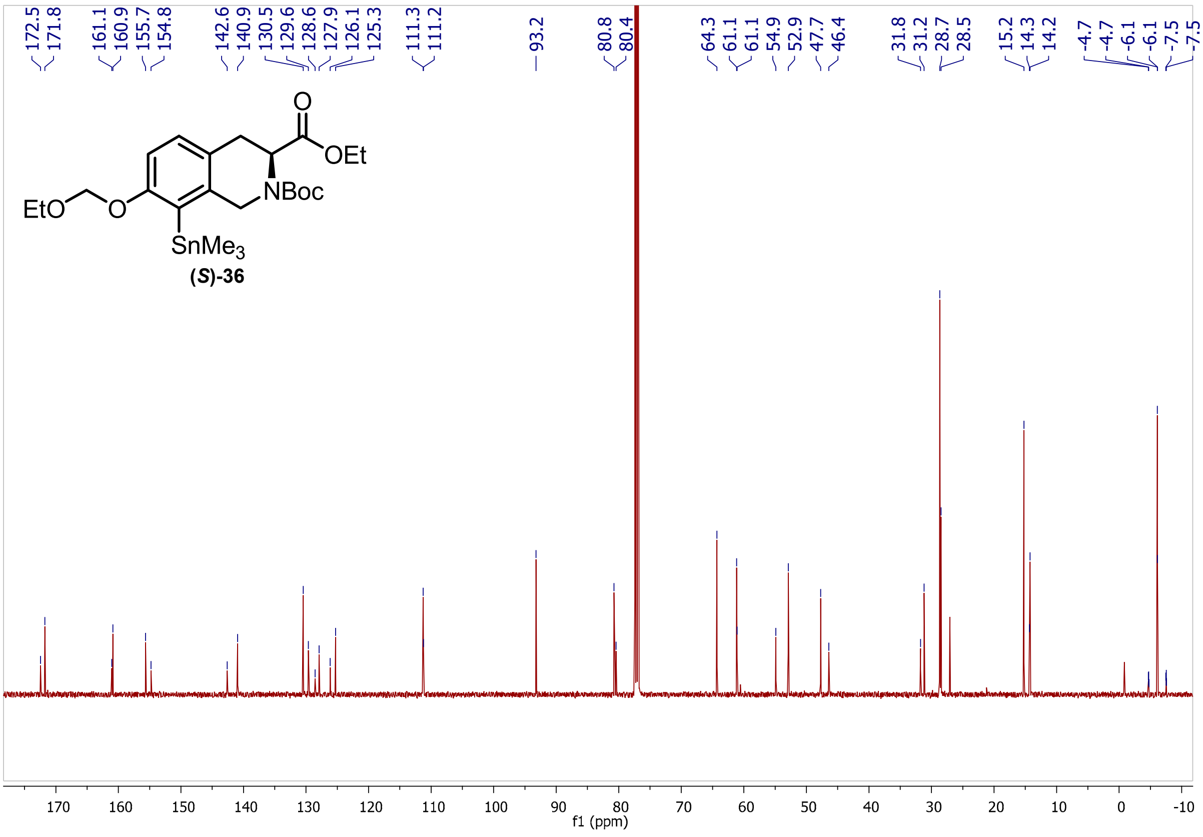

^{1}H (top) and ^{13}C (down) NMR spectra of compound **(*R*)-36** in $CDCl_3$.

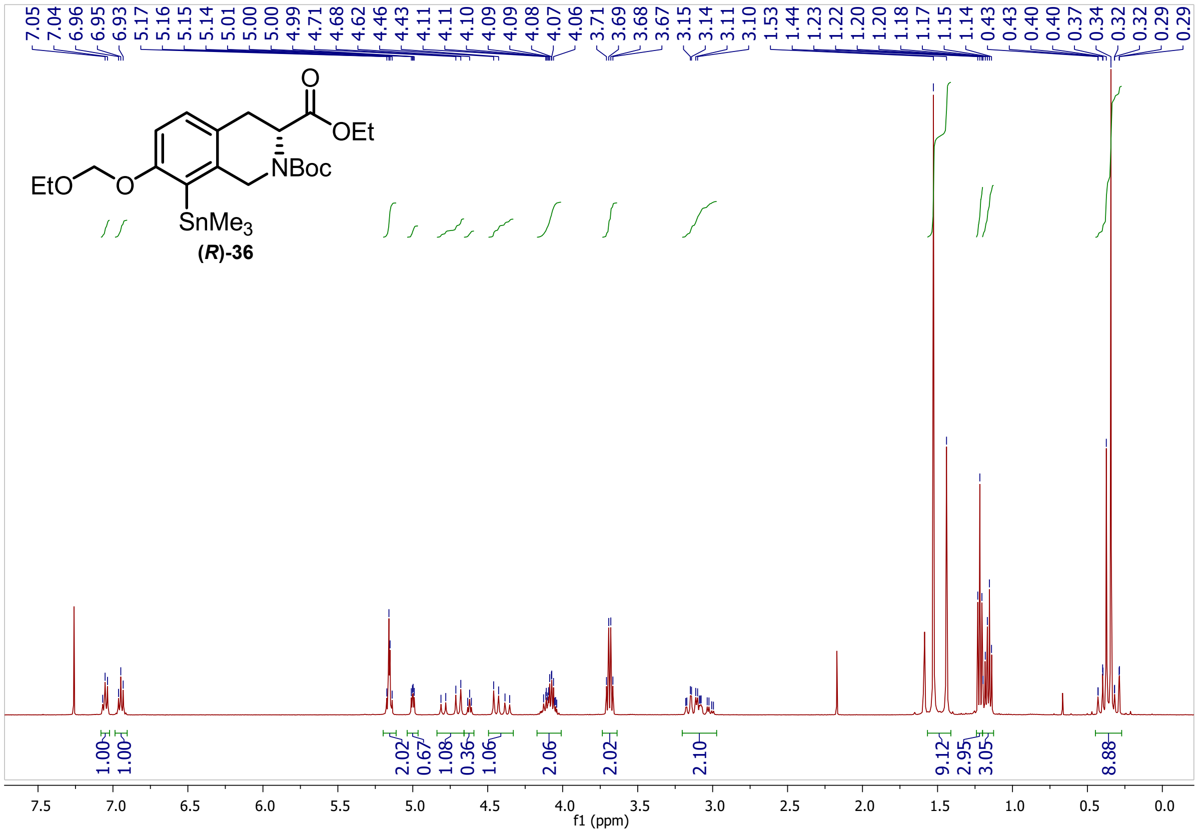

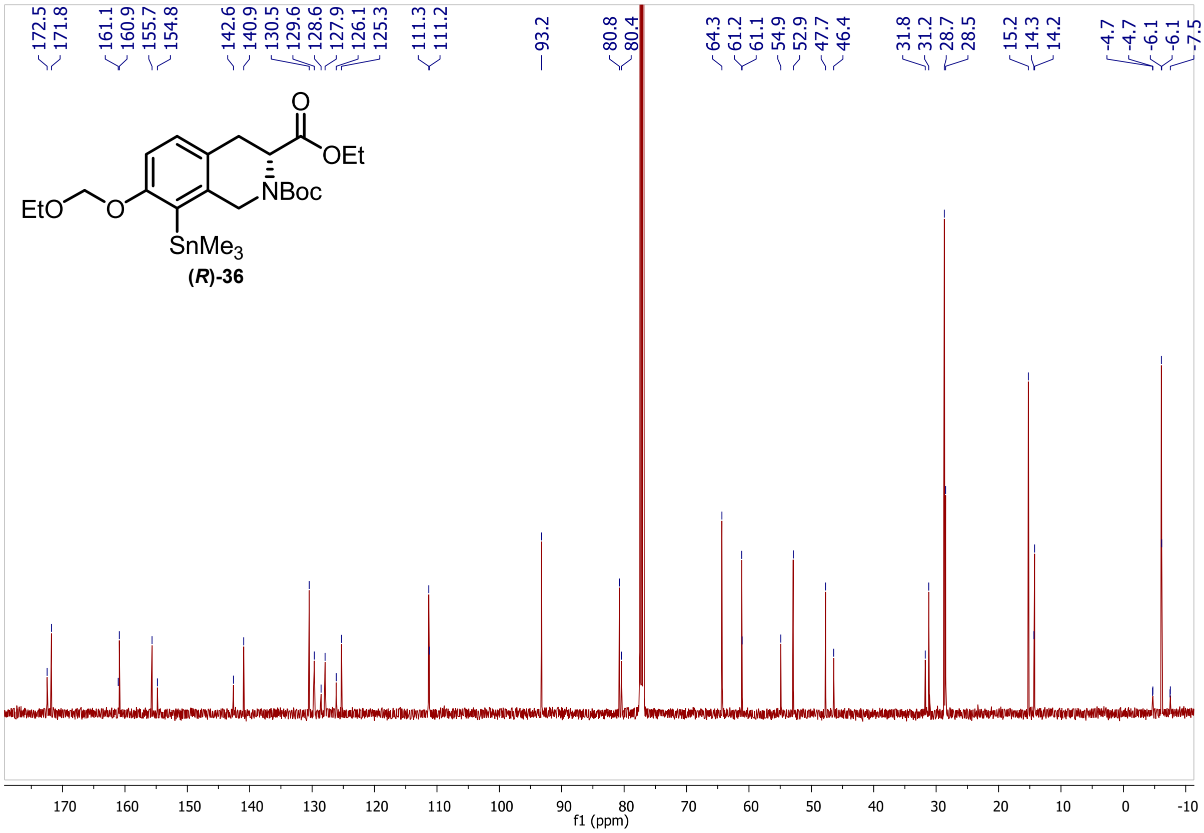

^{1}H (top), ^{13}C (middle) and ^{19}F (down) NMR spectra of compound **(*S*)-37** (**5-fluoro-L-TIC(OH).TFA**) in CD_3OD.

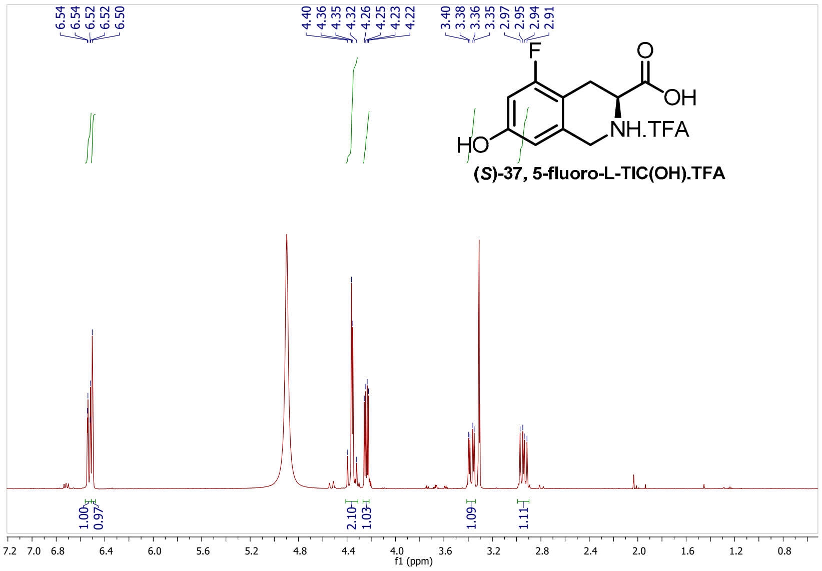

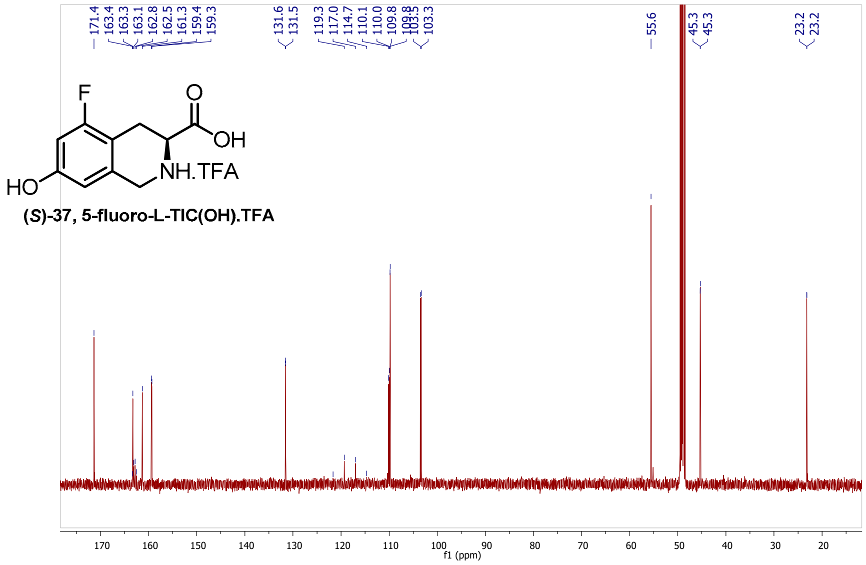

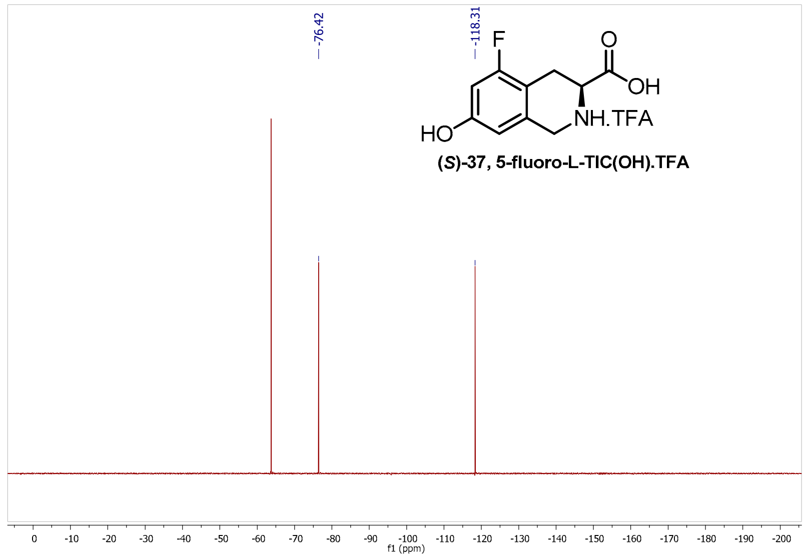

^{1}H (top), ^{13}C (middle) and ^{19}F (down) NMR spectra of compound **(*R*)-37** (**5-fluoro-D-TIC(OH).TFA**) in CD_3OD.

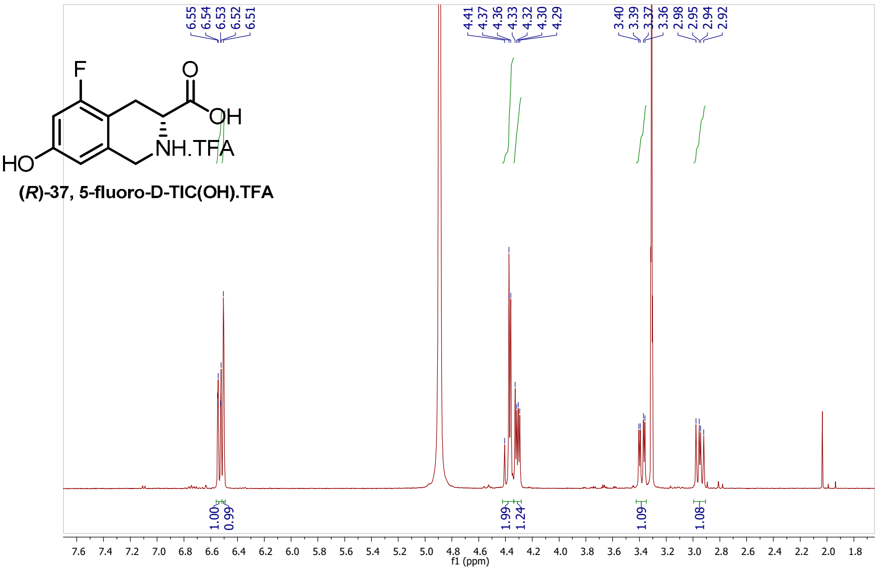

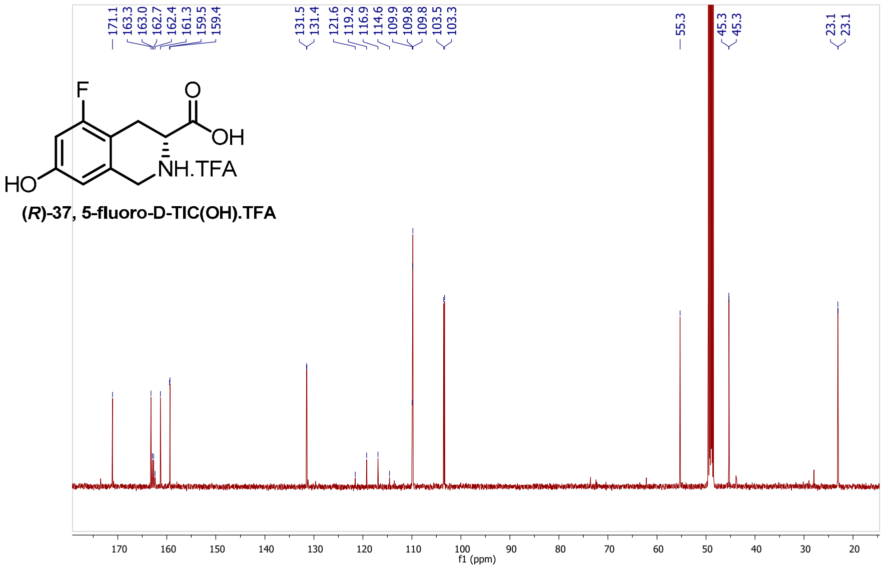

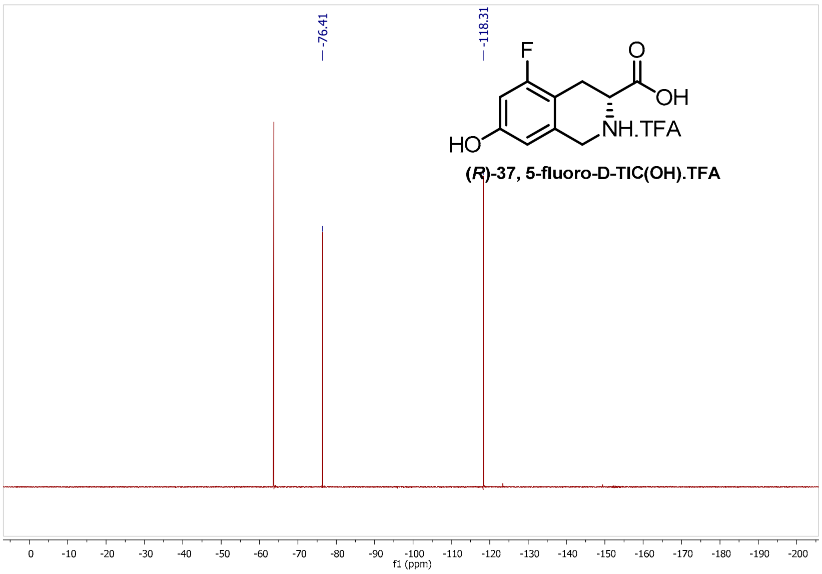

^{1}H (top), ^{13}C (middle) and ^{19}F (down) NMR spectra of compound **(*S*)-38** (**6-fluoro-L-TIC(OH).TFA)** in CD_3OD.

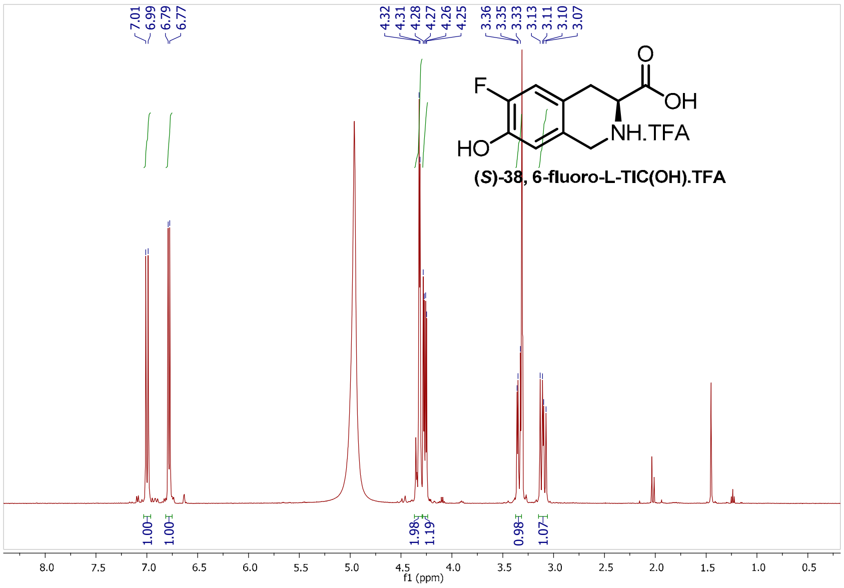

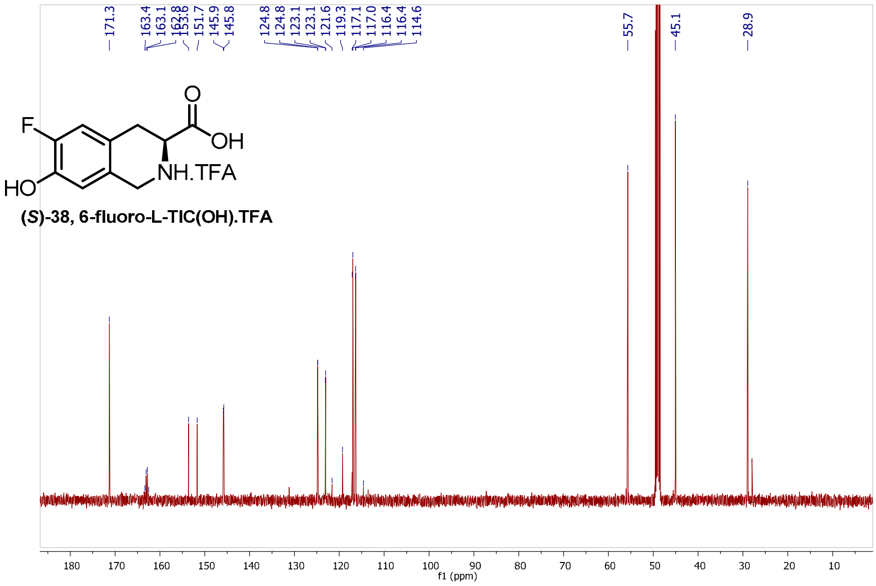

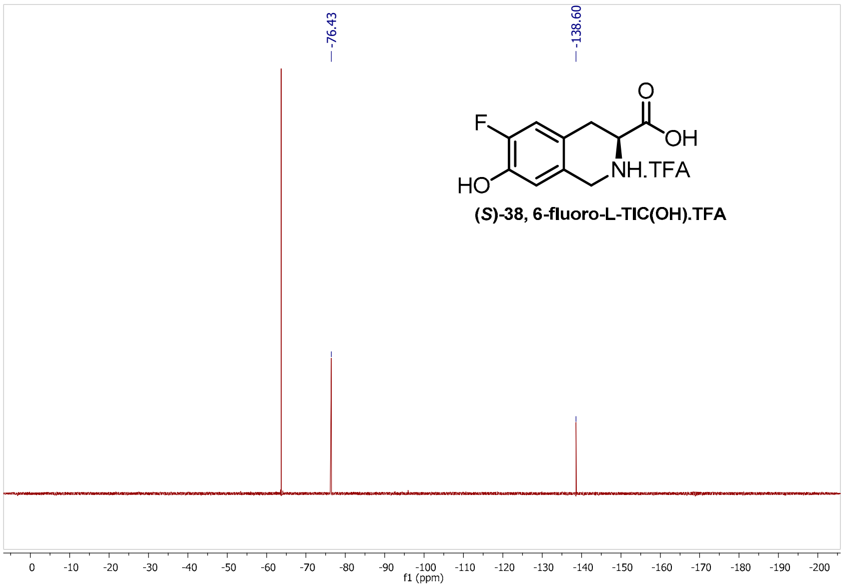

^{1}H (top), ^{13}C (middle) and ^{19}F (down) NMR spectra of compound **(*R*)-38** (**6-fluoro-D-TIC(OH).TFA**) in CD_3OD.

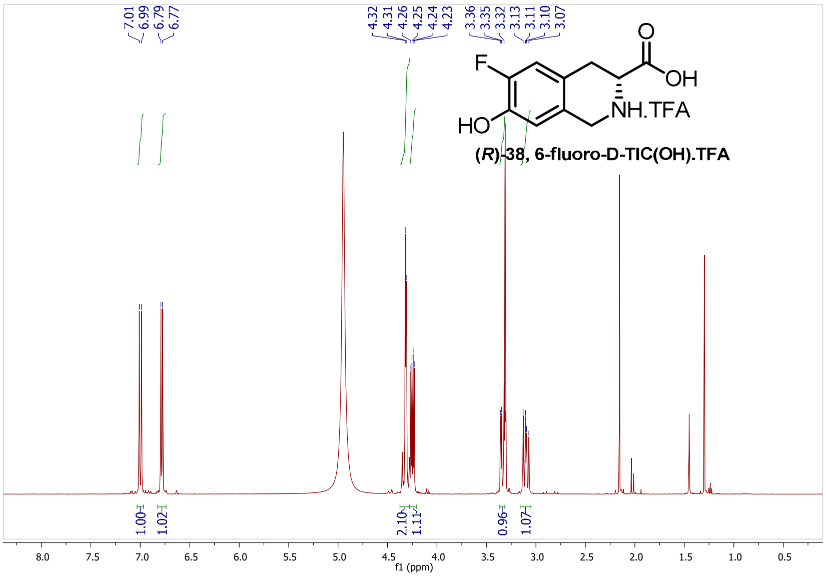

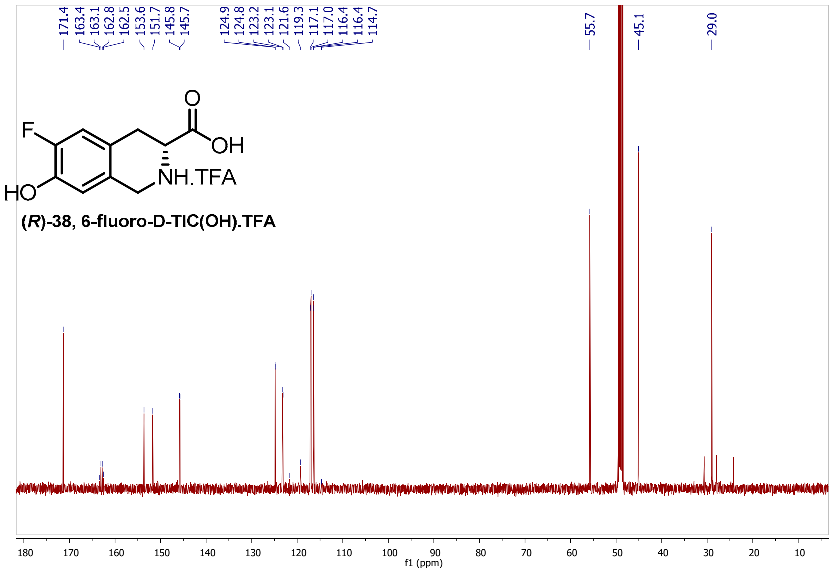

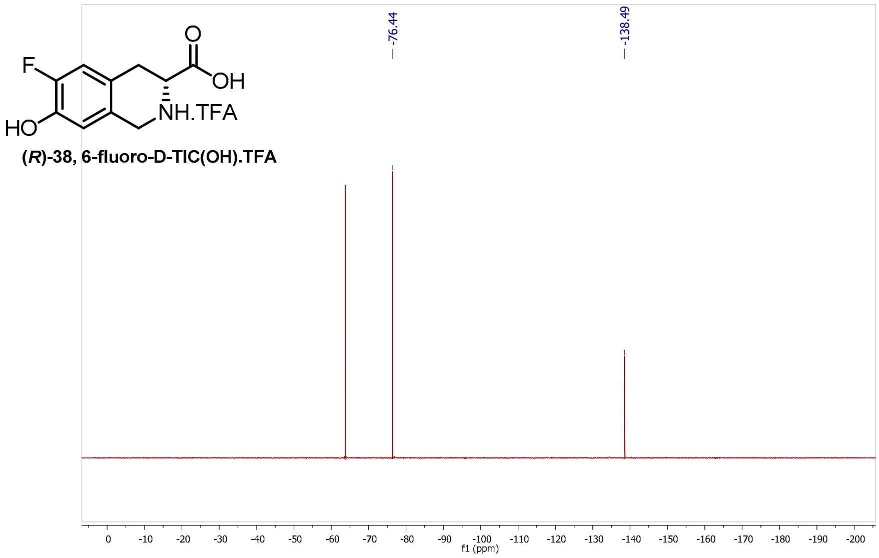

^{1}H (top), ^{13}C (middle) and ^{19}F (down) NMR spectra of compound **(*S*)-39** (**8-fluoro-L-TIC(OH).TFA**) in CD3OD.

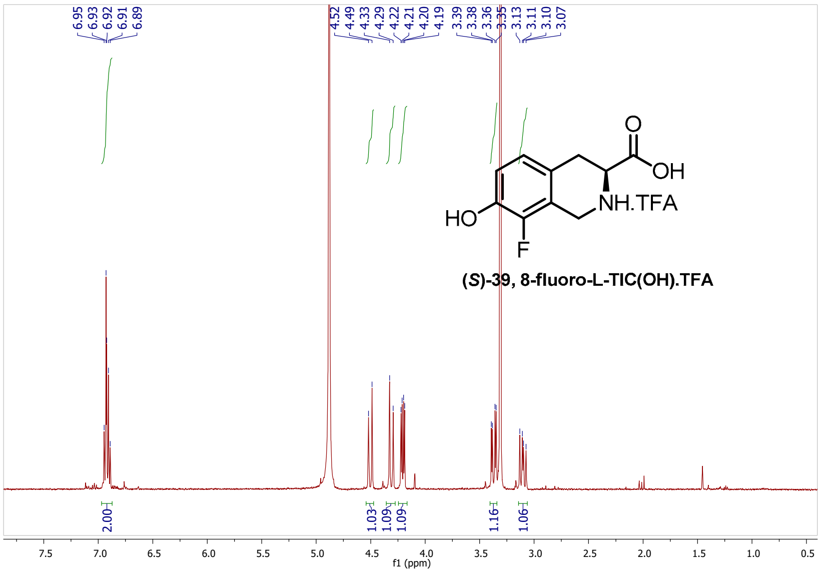

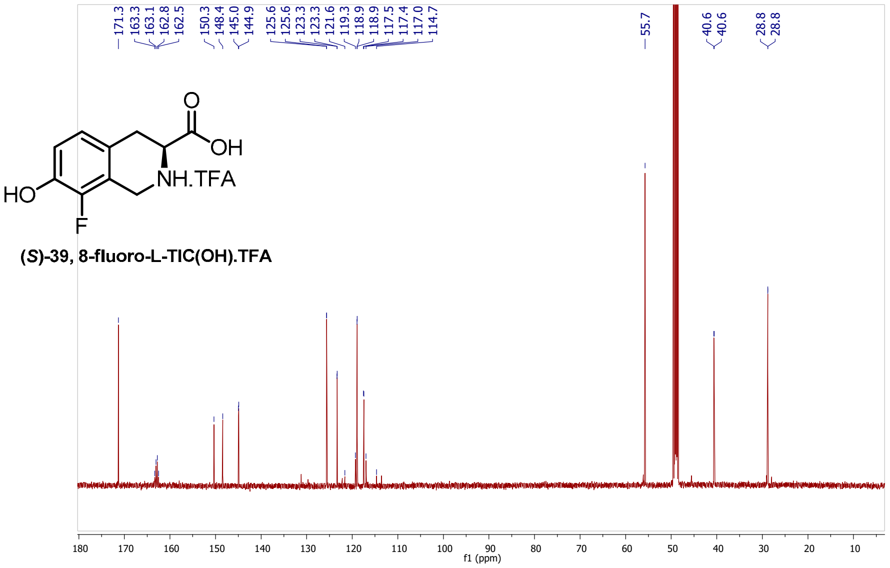

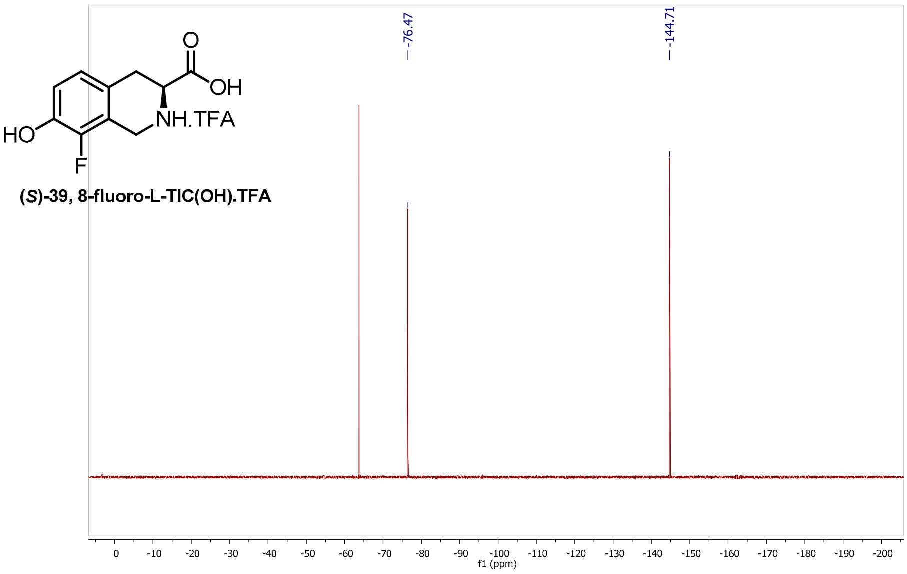

^{1}H (top), ^{13}C (middle) and ^{19}F (down) NMR spectra of compound ***(R)*-39** (**8-fluoro-D-TIC(OH).TFA**) in CD_3OD.

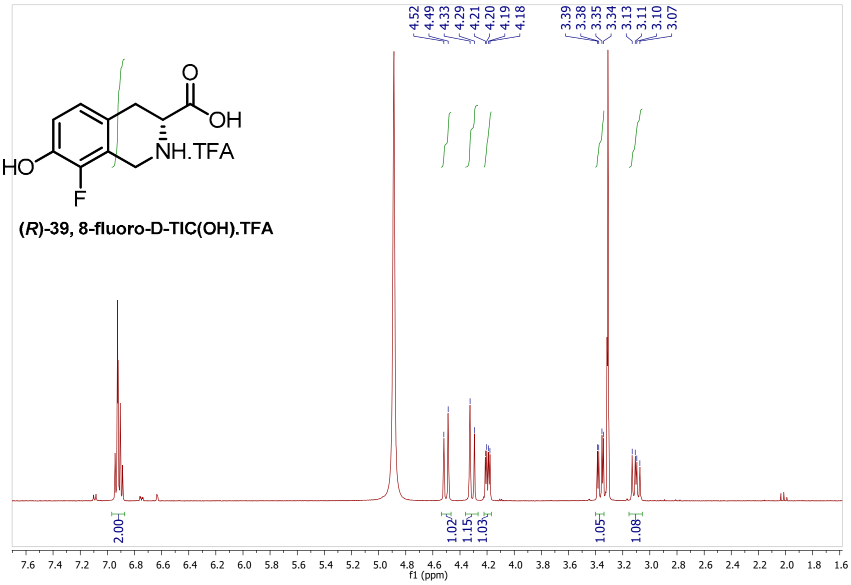

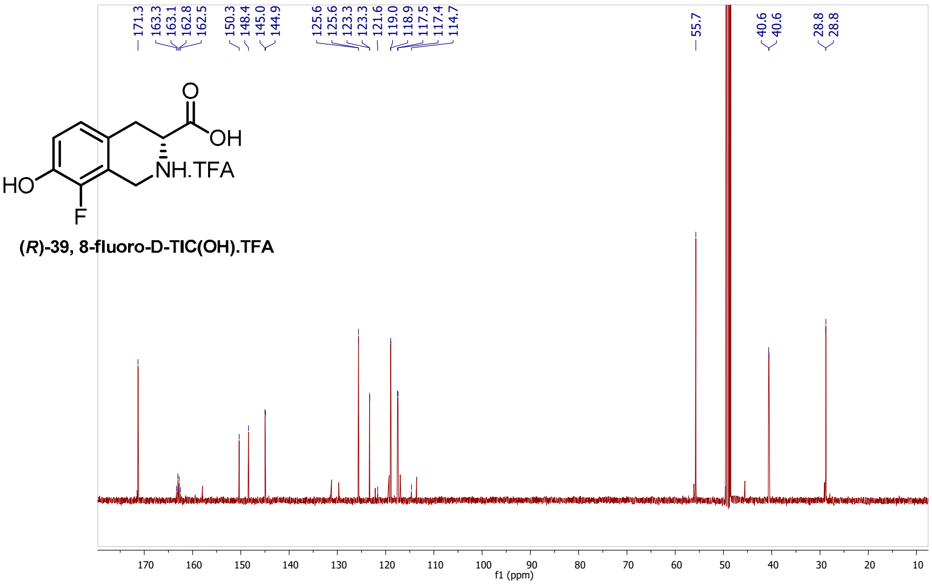

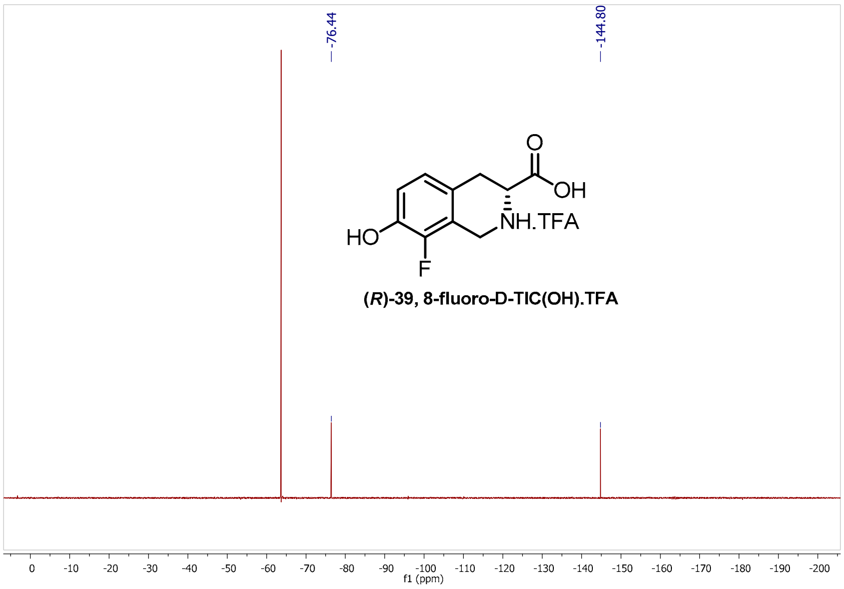

4. References

(1) Armarego, W. L. F.; Chai, C. L. L. *Purification of Laboratory Chemicals*, 6. ed.; Elsevier, Butterworth-Heinemann: Amsterdam, 2009.

(2) Chelopo, M. P.; Pawar, S. A.; Sokhela, M. K.; Govender, T.; Kruger, H. G.; Maguire, G. E. M. Anticancer Activity of Ruthenium(II) Arene Complexes Bearing 1,2,3,4-Tetrahydroisoquinoline Amino Alcohol Ligands. *European Journal of Medicinal Chemistry* **2013**, *66*, 407–414. https://doi.org/10.1016/j.ejmech.2013.05.048.

(3) Flynn, D. L.; Petillo, P. A. Modulateurs D'enzymes Et Traitements. WO2006071940A2, July 6, 2006.

(4) Farge, D.; Jossin, A.; Ponsinet, G.; Reisdorf, D. Thiazolo{8 3,4-B{9 Isoquinoline Derivatives and Pharmaceutical Compositions Containing Them. US4064247 (A), December 20, 1977.

(5) Harling, J. D.; Thompson, M. Novel Compounds. US2004142964A1, July 22, 2004.

(6) Ohta, M.; Takahashi, K.; Kasai, M.; Shoji, Y.; Kunishiro, K.; Miike, T.; Kanda, M.; Mukai, C.; Shirahase, H. Novel Tetrahydroisoquinoline Derivatives with Inhibitory Activities against Acyl-CoA: Cholesterol Acyltransferase and Lipid Peroxidation. *Chem. Pharm. Bull.* **2010**, *58* (8), 1066–1076. https://doi.org/10.1248/cpb.58.1066.

(7) Brown, M. L.; Kellar, K. J.; Levin, E. D.; Paige, M. a; Rezvani, A. H.; Xiao, Y.; Yenugonda, V. M. Ligands Nicotiniques Substitués Par Un Phényle, Et Leurs Procédés D'utilisation. WO2013071067A1, May 16, 2013.

(8) Jiang, J.; Zhang, J.; Chen, S.; Sun, L. Compounds for Inflammation and Immune-Related Uses. US2010130522 (A1), May 27, 2010.

(9) Moreno-Mañas, M.; Trepat, E.; Sebastián, R. M.; Vallribera, A. Asymmetric Synthesis of Quaternary α-Amino Acids Using d-Ribonolactone Acetonide as Chiral Auxiliary. *Tetrahedron: Asymmetry* **1999**, *10* (21), 4211–4224. https://doi.org/10.1016/S0957-4166(99)00457-7.

(10) Azukizawa, S.; Kasai, M.; Takahashi, K.; Miike, T.; Kunishiro, K.; Kanda, M.; Mukai, C.; Shirahase, H. Synthesis and Biological Evaluation of (S)-1,2,3,4-Tetrahydroisoquinoline-3-Carboxylic Acids: A Novel Series of PPAR.GAMMA. Agonists. *Chem. Pharm. Bull.* **2008**, *56* (3), 335–345. https://doi.org/10.1248/cpb.56.335.

(11) Makaravage, K. J.; Brooks, A. F.; Mossine, A. V.; Sanford, M. S.; Scott, P. J. H. Copper-Mediated Radiofluorination of Arylstannanes with [^{18} F]KF. *Org. Lett.* **2016**, *18* (20), 5440–5443. https://doi.org/10.1021/acs.orglett.6b02911.
